# Spin-orbit coupling induced semi-metallic state in the 1/3 hole-doped hyper-kagome Na_3_Ir_3_O_8_

**DOI:** 10.1038/srep06818

**Published:** 2014-10-29

**Authors:** Tomohiro Takayama, Alexander Yaresko, Akiyo Matsumoto, Jürgen Nuss, Kenji Ishii, Masahiro Yoshida, Junichiro Mizuki, Hidenori Takagi

**Affiliations:** 1 Max Planck Institute for Solid State Research, Heisenbergstrasse 1, 70569 Stuttgart, Germany; 2 Department of Physics and Department of Advanced Materials, University of Tokyo, 7-3-1 Hongo, Bunkyo-ku, Tokyo 113-0033, Japan; 3 SPring-8, Japan Atomic Energy Agency, Sayo, Hyogo 679-5148, Japan; 4 School of Science and Technology, Kwansei Gakuin University, Sanda, Hyogo 669-1337, Japan; 5 RIKEN Advanced Science Institute, Wako 351-0195, Japan

## Abstract

The complex iridium oxide Na_3_Ir_3_O_8_ with a B-site ordered spinel structure was synthesized in single crystalline form, where the chiral hyper-kagome lattice of Ir ions, as observed in the spin-liquid candidate Na_4_Ir_3_O_8_, was identified. The average valence of Ir is 4.33+ and, therefore, Na_3_Ir_3_O_8_ can be viewed as a doped analogue of the hyper-kagome spin liquid with Ir^4+^. The transport measurements, combined with the electronic structure calculations, indicate that the ground state of Na_3_Ir_3_O_8_ is a low carrier density semi-metal. We argue that the semi-metallic state is produced by a competition of the molecular orbital splitting of *t*_2*g*_ orbitals on Ir_3_ triangles with strong spin-orbit coupling inherent to heavy Ir ions.

The realization of quantum spin liquid is a long-sought dream in condensed matter physics, where exotic phenomena such as spinon Fermi surface, fractional excitations or unconventional superconductivity upon doping are anticipated^1^. The most promising arena for spin liquid is in geometrically frustrated lattices based on a triangular motif. Antiferromagnetically interacting spins on such a frustrated lattice cannot simultaneously satisfy all magnetic bonds and the interplay with quantum effect may give rise to a quantum liquid state of spins. To date experimental efforts have yielded several candidates, such as transition metal oxides with a kagome lattice^1^,^2^ and organic Mott insulators with a triangular lattice^1^,^3^.

A prime candidate in these recent discoveries is Na_4_Ir_3_O_8_^4^. It is one of the very first candidates for a three-dimensional quantum spin liquid, where Ir^4+^ ions with a localized *S* = 1/2 moment (or most likely close to *J_eff_* = 1/2 moment, see below) form a corner-sharing network of triangles in three-dimensions, called a “hyper-kagome” lattice. All the Ir sites and the Ir-Ir bonds are equivalent, rendering the hyper-kagome lattice magnetically frustrated. Indeed, Na_4_Ir_3_O_8_ exhibits no magnetic ordering down to 2 K despite the strong antiferromagnetic interaction inferred from the Curie-Weiss temperature *θ*_W_ ~ -650 K. This discovery triggered intensive experimental and theoretical surveys on this compound, including proposals for the presence of a spinon Fermi surface^5^.

In tandem with the discovery of Na_4_Ir_3_O_8_, complex Ir^4+^ oxides have recently been recognized as a novel playground for physics of strong spin-orbit coupling (SOC). SOC of Ir^4+^ is as large as *λ*_SO_ ~ 0.6 eV, reflecting the heavy atomic mass. *λ*_SO_ is significant even when compared with the other parameters dominating the electronic states, such as the inter-site hopping *t*, the Coulomb repulsion *U* and the crystal field splitting Δ. The interplay of the large SOC with the other parameters leads to the formation of unprecedented electronic phases. In the layered perovskite Sr_2_IrO_4_, for example, a half filled *J_eff_* = 1/2 state is formed by strong SOC, which is a superposition of three *t*_2*g*_ orbitals 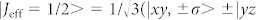, 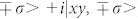) and gives rise to a spin-orbital Mott insulator^6^. The magnetic coupling of *J_eff_* = 1/2 moments in such spin-orbital Mott insulators can be distinct from those of the spin-dominant moments in 3*d* oxides, as exemplified by the possible Kitaev spin-liquid proposed for honeycomb iridates^7^. Such unique magnetic couplings in iridates may make the mystery of the possible spin-liquid state of Na_4_Ir_3_O_8_ even more intriguing^8^.

The insulating state of Na_4_Ir_3_O_8_ is marginally stabilized by a modest *U* with the help of strong SOC. Such weak Mottness, implying the close proximity to a metallic state^9^, has been proposed to play a vital role in realizing the spin-liquid ground state here, as well as in organic triangular spin liquids^1^. It is natural to expect that Na_4_Ir_3_O_8_ might switch into a metallic state by carrier-doping or by applying pressure. Exotic superconductivity at the critical border to a spin liquid might be anticipated in analogy with the organic systems^10^. The metallic state of hyper-kagome iridate is therefore worthy of exploration.

Despite such intriguing outlooks in Na_4_Ir_3_O_8_, not much progress has been achieved in the critical investigation of the spin-liquid like state including the role of spin-orbit coupling, largely due to the lack of single crystals. During the course of attempting to grow single crystals of Na_4_Ir_3_O_8_, we obtained single crystals of Na_3_Ir_3_O_8_, a B-site ordered spinel. The crystal structure is distinct from that of Na_4_Ir_3_O_8_, a spin liquid candidate, but shares the same Ir-O hyper-kagome network. Na_3_Ir_3_O_8_ therefore can be viewed as a doped hyper-kagome spin liquid. We report here that Na_3_Ir_3_O_8_ has a semi-metallic ground state produced by a subtle competition of strong spin-orbit coupling and molecular orbital splitting of *t*_2*g*_ electrons in an Ir_3_ triangular unit.

## Results

### Crystal Structure Analysis

**Figure 1:**
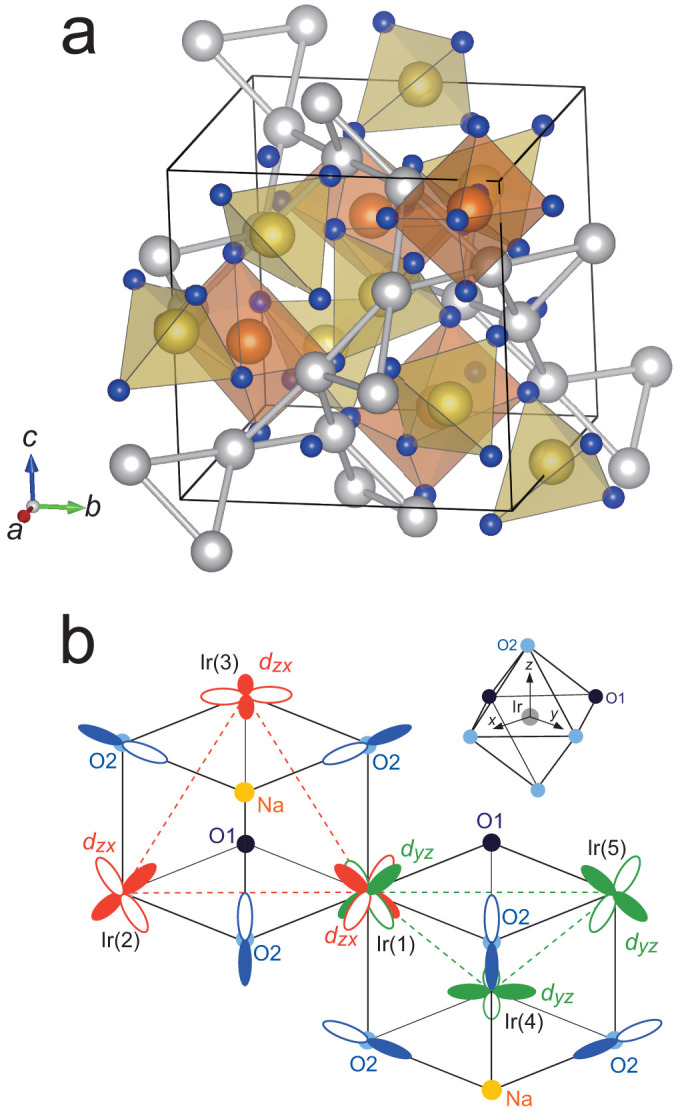
Crystal structure of Na_3_Ir_3_O_8_. (a) The unit cell of Na_3_Ir_3_O_8_ with a space group of *P*4_1_32. The orange, yellow, silver and blue spheres represent Na1, Na2, Ir and O atoms, respectively^19^. While Na1 atoms are enclosed in distorted O_6_ octahedra, Na2 atoms are coordinated tetrahedrally by oxygen atoms. The iridium atoms comprise corner-sharing triangles in three-dimensions called hyper-kagome lattice. (b) Schematic illustration of the hyper-kagome lattice showing formation of molecular orbitals on Ir_3_ triangles seen in the scalar relativistic calculation. *d_zx_* on Ir(1) has hopping paths to Ir(2) and Ir(3) via O2 *p*-orbitals, forming a localized molecular orbitals. Likewise, *d_yz_* on Ir(1) forms another molecular orbital with the like orbitals on Ir(4) and Ir(5). Note that the *t_2g_* orbitals are defined in a local frame (sketched in the inset) which is unique for each Ir site. The hopping via O1, which is likely much smaller than the one via O2^17^, is neglected here.

Single crystals of Na_3_Ir_3_O_8_ were grown by a flux method (see Methods). The obtained crystals are stable in air. Detailed refinement of structure using single crystal X-ray diffraction was performed on the crystals. Satisfactory refinement was performed with space groups *P*4_1_32 or *P*4_3_32. The obtained structural parameters are listed in Table 1. The structure shown in Fig. 1a can be viewed as an ordered spinel, an intimately related but distinct structure to that of Na_4_Ir_3_O_8_. Rewriting the chemical formula of 1/2 Na_3_Ir_3_O_8_ as Na(Na_1/4_, Ir_3/4_)_2_O_4_, in correspondence with that of spinel AB_2_O_4_, is useful to understand the structure. Na2 in Table 1 corresponds to the tetrahedral A-site of spinel structure. In Na_4_Ir_3_O_8_, this site is empty and, instead, the octahedral A-site is occupied by Na_1.5_. The occupancies of the octahedral sites were found to be zero in the Na_3_Ir_3_O_8_ single crystal within the resolution of structural analysis. Na1 corresponds to 1/4 of the pyrochlore B sub-lattice. The remaining 3/4 of the pyrochlore B sub-lattice sites are occupied by Ir atoms. All the Ir sites are equivalent and form the same hyper-kagome network as in Na_4_Ir_3_O_8_. The presence of chirality in the hyper-kagome network gives rise to two possible space groups with different chiralities. The crystals under investigation contained racemic twins (see Supplementary). We did not find any signature of non-stoichiometry such as sodium deficiency in the refinement and therefore the composition of single crystal should be stoichiometric Na_3_Ir_3_O_8_.

### Transport and Magnetic Properties

**Figure 2:**
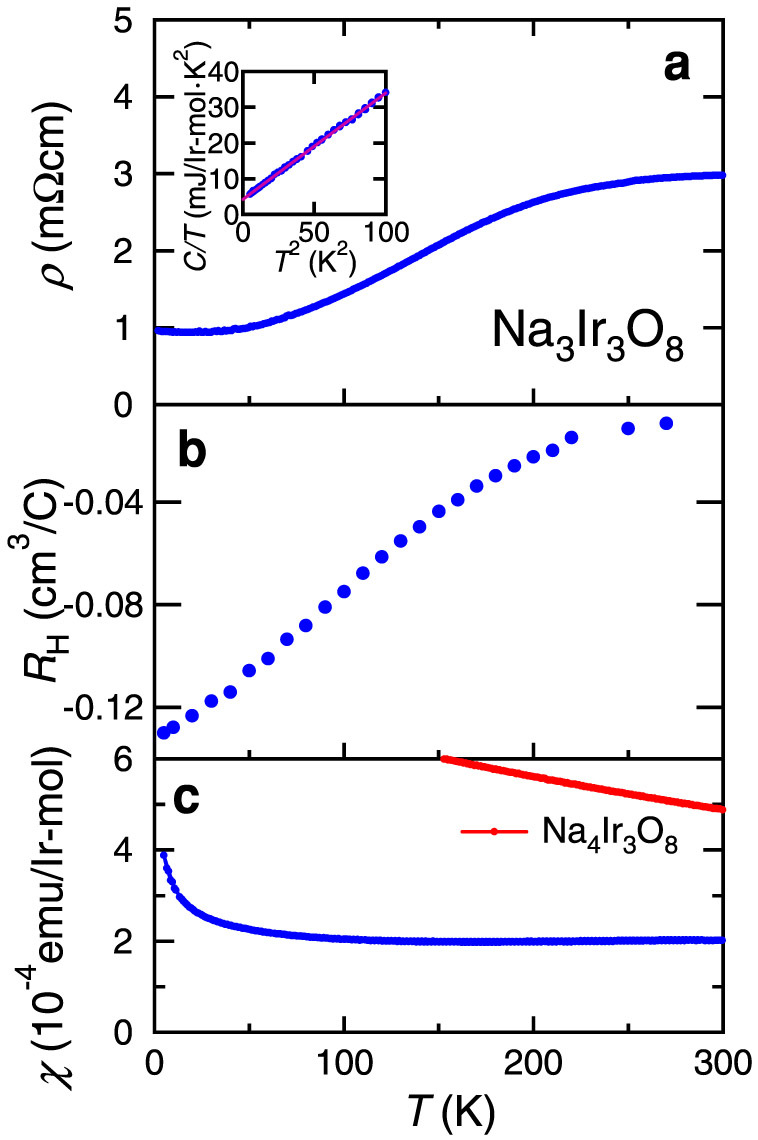
Transport, magnetic and thermodynamic properties of Na_3_Ir_3_O_8_. (a) Resistivity, (b) Hall coefficient and (c) Magnetic susceptibility of Na_3_Ir_3_O_8_ single crystal. The red dots in (c) represent magnetic susceptibility of Na_4_Ir_3_O_8_ polycrystalline sample for comparison. The inset in (a) shows specific heat *C* divided by temperature *T* of Na_3_Ir_3_O_8_. The solid line is a fit based on the conventional model *C*/*T* = *γ* + *βT*^2^.

The composition of Na_3_Ir_3_O_8_ corresponds to Ir valence of 4.33+, not 4+. Considering that Na_4_Ir_3_O_8_ is an Ir^4+^ (5*d*^5^) Mott insulator, Na_3_Ir_3_O_8_ may be viewed as 1/3 hole-doped hyper-kagome spin liquid. Indeed, the obtained single crystals were found to show metallic behavior of resistivity as shown in Fig. 2a, in marked contrast to the spin liquid Na_4_Ir_3_O_8_. The magnitude of resistivity is relatively large as a metal, ~1 mΩcm at 5 K. The Hall coefficient of Na_3_Ir_3_O_8_ indicates that the poorly metallic behavior originates from a low carrier concentration. The Hall coefficient shown in Fig. 2b is negative and its magnitude is orders of magnitude larger than those of typical metals, indicating that a very low density of electrons dominates the transport. The carrier number estimated from the Hall constant is of the order of 10^19^ cm^−3^ at 5 K, which is too small if the picture of a simple 1/3 hole-doped Mott insulator with 2/3 electrons is to be assumed. The Hall mobility at 5 K is as large as ~100 cm^2^/V·s and, clearly, the disorder is not the dominant factor of the poorly metallic behavior. The Hall coefficient shows a rapid decrease, more than one order of magnitude, with increasing temperature from 5 K to 300 K, despite the metallic behavior of resistivity. This very likely implies the coexistence of two different types of carriers at least at high temperatures, suggesting that Na_3_Ir_3_O_8_ is either a semi-metal or a very narrow gap semiconductor doped with a small number of carriers. The result of band calculation shown below indicates that the former is the case. Unexpectedly, the ground state of a doped hyper-kagome appears to be very close to a band insulator.

The low temperature specific heat of Na_3_Ir_3_O_8_ single crystal, shown in the inset of Fig. 2a, yields an electronic specific heat coefficient *γ* = 4.3 mJ/Ir-mol·K^2^. This value is pronounced especially when the vanishingly small density of carriers is considered and therefore implies the presence of heavy mass carriers. The magnetic susceptibility (Fig. 2c) is almost temperature-independent, distinct from that of magnetic Na_4_Ir_3_O_8_. The paramagnetic susceptibility is estimated to be *χ*_PM_ = 2.7 × 10^−4^ emu/Ir-mol by subtracting the contribution of core diamagnetism taken from the values for Na^+^, Ir^4+^ and O^2−^. This value gives the Wilson ratio *R*_W_ ~ 4.6 combined with *γ* = 4.3 mJ/Ir-mol·K^2^. The large *R*_W_ at a glance would mean a Stoner enhancement and close proximity to ferromagnetism. Considering the small density of carriers, we speculate that the enhanced *χ*_PM_ might be ascribed alternatively to a superposition of Van Vleck-like contribution.

### Resonant Inelastic X-ray Scattering

**Figure 3:**
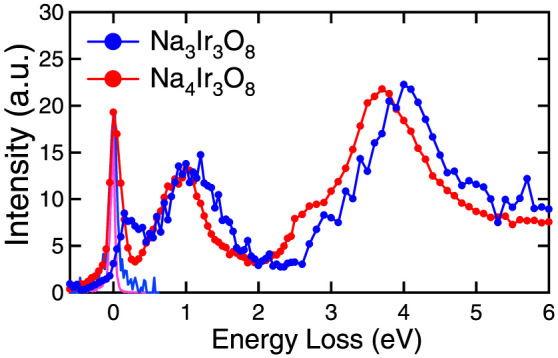
RIXS spectra of Na_3_Ir_3_O_8_ single crystal and polycrystalline Na_4_Ir_3_O_8_ at Ir *L*_3_-edge. The two spectra were normalized by the intensity of the high energy tail above 6 eV. The blue and pink solid lines around zero energy show the normalized elastic signals independently measured with *σ*-polarized incident X-ray.

To verify the 1/3 hole-doped state, we performed a resonant inelastic X-ray scattering (RIXS) at *L*_3_-edge of Ir(2*p*_3/2_ → 5*d*)^11^ on Na_3_Ir_3_O_8_ single crystals and Na_4_Ir_3_O_8_ polycrystalline samples. In the obtained RIXS spectra shown in Fig. 3, three peaks at around 0.2, 1.0 and 4.0 eV are clearly observed. All those peaks show only small dispersions, which very likely originates from the intra-atomic excitations within the *d*-orbitals of Ir. The intra-atomic character of excitations justifies the comparison of spectra for the single crystal Na_3_Ir_3_O_8_ and the polycrystalline Na_4_Ir_3_O_8_. A clear shift of peaks at 1.0 eV and 4.0 eV to lower energy and a strong suppression of the 0.2 eV peak are clearly seen in Na_4_Ir_3_O_8_. The peak at 4.0 eV can be assigned to *t*_2*g*_→ *e_g_* transitions, whereas the peaks at 1.0 eV and 0.2 eV likely originate from the excitations within the *t*_2*g*_ manifold. The shift of 4.0 eV peak can be ascribed to the increased Ir-O distance (~3%) in Na_4_Ir_3_O_8_ and hence the reduced cubic splitting 10 Dq. The change in the 1.0 eV and 0.2 eV peaks cannot be accounted for simply by the crystal field effect and very likely reflects the difference of band filling between the two compounds. This point will be justified based on the result of band calculation described below, which supports the 1/3 hole-doped state in Na_3_Ir_3_O_8_.

### Electronic Structure Calculation

**Figure 4:**
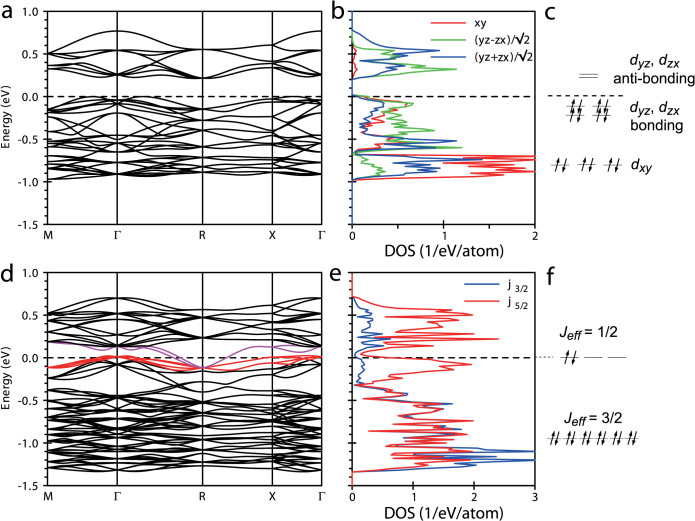
Electronic structure calculation for Na_3_Ir_3_O_8_. (a) Scalar-relativistic band structure, and (b) orbital resolved Ir 5*d* density of states (DOS). The *xy* orbital represents a *t*_2_*_g_* orbital lying in the plane which contains two larger Ir-O1 bonds. (c) Schematic energy level splitting of the *t*_2*g*_ orbitals in an Ir_3_ triangular molecule. The *d_xy_* orbitals have lower energy due to the crystal field. The *d_yz_* and *d_zx_* orbitals form bonding and anti-bonding molecular orbitals, which produce an energy gap. (d) Relativistic band structure including spin-orbit coupling (SOC). The bands which form hole and electron pockets are colored in red and magenta, respectively. (e) Ir 5*d* density of states corresponding to (d) resolved with *j* = 5/2 and 3/2 characters. (f) Local *d*-level splitting for an Ir_3_ triangle with SOC. At each Ir atom coordinated octahedrally by O atoms, SOC splits the *t*_2*g*_ orbitals into lower lying *J_eff_* = 3/2 quartet and upper *J_eff_* = 1/2 doublet. 14 electrons in an Ir_3_ triangle fills up the *J_eff_* = 3/2 states, while the *J_eff_* = 1/2 states are occupied partially.

*ab initio* electronic structure calculation using a fully relativistic LMTO code^12^ revealed that Na_3_Ir_3_O_8_ is a compensated semi-metal due to the interplay of periodic potential and SOC, which is consistent with the experimental observation described above. Figure 4 depicts the electronic states around the Fermi energy where *t*_2_*_g_* orbitals of Ir have a dominant contribution. The 5*d* electrons are accommodated into the *t*_2_*_g_* manifold due to the large *t*_2_*_g_* – *e_g_* crystal field splitting. The number of *d*-electrons per Ir atom is non-integer, 4.67 (Ir^4.33+^). In the unit formula with 3 Ir atoms, we have even number of electrons, 14( = 3 × 4.67) *t*_2_*_g_* electrons. In the scalar-relativistic calculation neglecting SOC, shown in Fig. 4a, a well-defined gap of 0.2 eV can be seen within the *t*_2*g*_ bands. 14 *t*_2*g*_ electrons fill up all the bands below the 0.2 eV gap and the system is a band insulator. A similar gap separating *t*_2_*_g_* bands and explained by strong *p*-*d* hopping was also found for Na_4_Ir_3_O_8_^13^, where, due to the higher Na content, the unoccupied *t*_2_*_g_* bands above the gap in Na_3_Ir_3_O_8_ are partially filled.

Strong SOC of Ir, in reality, splits the conduction and the valence bands substantially and a negative band gap is realized. The band structure calculated with SOC is shown in Fig. 4d. Since the crystal structure lacks inversion symmetry, SOC lifts the Kramers degeneracy everywhere except for time-reversal invariant points. In the presence of realistic SOC, a pair of unoccupied *t*_2_*_g_* bands, colored in magenta, bends down near the R point and becomes degenerate with the valence bands at the R point. This pronounced SOC effect results in the overlap of the pair of conduction band and the valence band colored in red, and creates two electron-like Fermi surfaces around the R point and four hole-like Fermi surfaces near the Γ point. Na_3_Ir_3_O_8_ is therefore a robust semi-metal, protected by the degeneracy at the R point. From the calculation, the masses of carriers are estimated; 1.4, 2.3, 3.7 and 5.6*m*_0_ for hole bands and 1.8*m*_0_ for two electron bands. The relatively heavy masses yield a large specific heat coefficient *γ*_calc_ = 2.9 mJ/Ir-mol·K^2^, though the carrier density is small. The *γ*_calc_ is close to and only 35% smaller than the experimentally observed *γ* = 4.3 mJ/Ir-mol·K^2^. The more dispersive electron bands with higher mobility should dominate the transport, again consistent with the negative sign of Hall constant experimentally observed. The recent observation of Fano resonances in the phonon spectra of Na_3_Ir_3_O_8_ provides further evidence for the semi-metallic state, where the electron-hole excitation continuum interferes with superimposed discrete phonon states^14^.

## Discussion

The effect of SOC leads to the closure of the scalar relativistic gap in Na_3_Ir_3_O_8_. An inspection of orbitally resolved densities of Ir 5*d* states reveals that the gap closure is a consequence of the competition between SOC and the level splitting associated with the formation of molecular orbitals on the Ir_3_ triangle. We consider Ir_3_ triangular “molecules” which are the basic structural unit of the hyper-kagome network. The dominant hopping process between Ir *t*_2*g*_ states in the hyper-kagome lattice is the one between the nearest neighbor Ir *d*-orbitals via oxygen *p* orbitals (*t_pd_*_π_) which is likely much larger than the direct Ir *d*-*d* hopping^15^,^16^. There are two oxygen sites, O1 and O2, in Na_3_Ir_3_O_8_, forming the IrO_6_ octahedra, and the Ir-O bond lengths for those two sites are very different (Ir-O1: 2.053 Å, Ir-O2: 1.976 or 1.978 Å). Because of the contrasting Ir-O bond lengths, the hopping via O2 is expected to be significantly stronger than via O1 as in the case of Na_4_Ir_3_O_8_^17^. Let us define for each Ir site its own local frame with the *x* and *y* axis lying in the plane of the IrO_4_ square with two more distant O1 ions (see inset of Fig. 1b). Among the three *t*_2*g*_ orbitals of Ir, the *d_xy_* orbital should be energetically stabilized by the crystal field associated with the longer Ir-O1 distance. This can be clearly recognized in the orbitally resolved density of states shown in Fig. 4b. Since *d_xy_* bands are fully filled, only *d_yz_* and *d_zx_* orbitals are responsible for the bands right above and below the 0.2 eV gap.

Both *d_yz_* and *d_zx_* have the hopping path to *t*_2*g*_ orbitals of the neighboring Ir atoms via O2 but it is spatially limited. *d_zx_* on Ir(1) can hop only to *d_zx_* on Ir(2) or Ir(3), whereas *d_yz_* only to *d_yz_* on Ir(4) or Ir(5), meaning that for each of the two orbitals the hopping paths via O2 are restricted to one of the two Ir_3_ triangles sharing common Ir(1). In the simplified picture which considers only the hopping via O2 2*p* orbitals, therefore, the *d_zx_*states form molecular orbitals on the Ir(1)Ir(2)Ir(3) triangle, namely, two degenerate bonding and one anti-bonding molecular orbitals. The *d_yz_* on Ir(1) participates in molecular orbitals on the Ir(1)Ir(4)Ir(5) triangle. Note that *d_zx_* and *d_yz_* orbitals are nearly orthogonal to each other, which means that those molecular orbitals which belong to different triangles do not interact and are almost localized. Considering the energy levels of the Ir_3_ molecule with 3 × 3 = 9 *t*_2g_ orbitals, 3 *d_xy_* orbitals have the lowest energy. The other two, *d_yz_* and *d_zx_*, orbitals form 4 bonding and 2 anti-bonding orbitals, as schematically shown in Fig. 4c. With 14 electrons per Ir_3_ triangle, 3 *d_xy_* and 4 bonding molecular orbitals are fully filled and the splitting of bonding and anti-bonding molecular orbitals gives rise to an energy gap.

SOC of heavy Ir 5*d* orbitals is very large ~0.6 eV and may reconstruct the electronic structure. In a cubic environment SOC splits three-fold degenerate *t*_2_*_g_* *d*-orbitals into 2:1 ratio of lower *J_eff_* = 3/2 (admixture of *j* = 5/2 and 3/2 characters) and higher *J_eff_* = 1/2 states (purely *j* = 5/2 character), which accommodate 12 and 6 electrons respectively for the Ir_3_ triangle, as shown in Fig. 4f. In the strong SOC limit, *J_eff_* = 3/2 states are fully filled up and *J_eff_* = 1/2 states are 1/3 filled. The splitting into *J_eff_* = 3/2 and higher *J_eff_* = 1/2 states can be seen in the relativistic calculation with SOC shown in Figs. 4d, e. The competition between the molecular orbital split and strong SOC should be responsible for suppression of the 0.2 eV gap in the scalar relativistic calculation. This is because SOC mixes all three *t_2g_* states, *d_zx_* and *d_yz_* forming two distinct molecular orbitals and *d_xy_*.

Based on the calculated electronic structure, we can reasonably assign the relevant *d*-*d* excitations to the peaks observed in the RIXS spectrum in Fig. 3. The peak structure observed around 1.0 eV corresponds to the excitations from the *J_eff_* = 3/2 to the unoccupied *J_eff_* = 1/2 bands. The peak around 0.2 eV originates from the transition between the occupied *J_eff_* = 1/2 bands with remnant molecular bonding character and the unoccupied *J_eff_* = 1/2 bands with remnant anti-bonding character, likely representing the strong inter-band transition between the pair of flat bands crossing *E*_F_. The absence of the 0.2 eV peak in the RIXS spectrum of Na_4_Ir_3_O_8_ is fully consistent with the filling of the unoccupied and flat *J_eff_* = 1/2 bands associated with the removal of 1/3 hole and opening of Mott gap in Na_4_Ir_3_O_8_.

In summary, we successfully synthesized single crystals of hyper-kagome iridate Na_3_Ir_3_O_8_. Unlike the spin-orbital Mott insulator Na_4_Ir_3_O_8_, Na_3_Ir_3_O_8_ was found to be a semi-metal. The electronic structure calculation indicates that Na_3_Ir_3_O_8_ should be a band insulator with absence of SOC. The semi-metallic state is shown to arise from a competition between the splitting of molecular orbitals on Ir_3_ triangles and strong SOC. The semi-metallic Na_3_Ir_3_O_8_ might be an intriguing platform to test the possible non-trivial topological effects, associated with the frustrated and chiral geometry of the lattice and the negative gap produced by SOC. As an example, the breaking of inversion symmetry due to the chirality, together with strong SOC, produces a sizable Rashba splitting with a Dirac dispersion at the Γ point in the hole band, which harbors non-trivial Berry phase in electron transport^18^. We therefore believe that such material inheriting strong SOC and a unique lattice topology provides a tool to substantialize novel states of matter.

## Method

### Crystal growth and characterizations

Single crystals of Na_3_Ir_3_O_8_ were grown by a flux-growth technique. The mixture of Na_2_CO_3_, IrO_2_ and NaCl with a ratio of 5:1:20 was loaded in an alumina crucible, and heated up to 1075°C in an oxygen atmosphere. It was then slowly cooled to 1000°C with a cooling rate of 2°C/hour, and subsequently furnace-cooled down to room temperature. Black and block-shaped single crystals were found in the solidified melt. The polycrystalline samples of Na_4_Ir_3_O_8_ were synthesized by a conventional solid state reaction as a reference material. The crystal structure of single crystals was analyzed by X-ray diffraction using a three circle diffractometer (Bruker AXS) equipped with SMART APEX II CCD, and Mo *Kα* radiation (see Supplementary). Transport, magnetic and thermodynamic properties were measured with Quantum Design PPMS and MPMS.

In the obtained crystals, we in fact found a very small amount of insulating crystals besides the majority of metallic ones. The X-ray diffraction indicated that the insulating crystals also had the Na_3_Ir_3_O_8_ stoichiometry, and the spectroscopic measurements showed no difference between the metallic and insulating crystals. Judging from the poor quality of the insulating crystals, we suspect that the insulating crystals include domains of the Na_4_Ir_3_O_8_ phase and there might be a temperature window where Na_4_Ir_3_O_8_ phase is stabilized during the crystal growth. However, the growth of Na_4_Ir_3_O_8_ single crystal is a future perspective and out of the scope of this article.

### Resonant inelastic X-ray scattering (RIXS)

Resonant inelastic X-ray scattering (RIXS) measurement was performed at BL11XU of SPring-8. Incident X-rays were monochromatized by a double-crystal Si(111) monochromator and a secondary Si(844) channel-cut monochromator. Horizontally scattered X-rays were analyzed by a diced and spherically-bent Si(844) crystal. The total energy resolution was 70 meV. The energy of the incident X-ray was tuned at 11.214 keV, which corresponds to the *L*_3_-edge of Ir (2*p*_3/2_ → *t*_2*g*_).

The samples used were single crystal Na_3_Ir_3_O_8_ and polycrystalline Na_4_Ir_3_O_8_. The spectra were collected at 20 K in Na_3_Ir_3_O_8_, whereas at 300 K in Na_4_Ir_3_O_8_. The difference in the measurement temperature should not change the overall spectra. The contribution of elastic scattering was evaluated by independently measuring the elastic signal with a *σ*-polarized configuration. It was almost negligible in Na_3_Ir_3_O_8_, whereas the strong quasi-elastic signal was seen around zero energy in the Na_4_Ir_3_O_8_ polycrystalline sample. This is possibly attributed to the elastic signal coming from concomitant Bragg reflections and/or to the phonon excitations inherent to the measurement at room temperature.

## Author Contributions

T.T. and H.T. conceived and designed the project. T.T. and A.M. performed synthesis and magnetic and transport measurements. J.N. analyzed the crystal structure. K.I., M.Y., T.T., A.M. and J.M. measured and analyzed the RIXS data. A.Y. conducted electronic structure calculation. T.T., A.Y. and H.T. wrote the manuscript and all authors discussed and reviewed the paper.

## Supplementary Material

Supplementary Information Supplementary information

Supplementary Information Dataset 1

## Figures and Tables

**Table 1:** Refined structural parameters of Na_3_Ir_3_O_8_. The space group is *P*4_1_32 (No. 213) and *Z* = 4, and the lattice constant is *a* = 8.9857(4) Å. *g* and *U*_iso_ denote site occupancy and isotropic displacement parameter, respectively. The final *R* indices are *R* = 0.0133 and *wR* = 0.0287

Atom	Site	*g*	*x*	*y*	*z*	*U*_iso_ (Å^2^)
Ir	12*d*	1	0.61264(1)	*x* + 1/4	5/8	0.00802(4)
Na1	4*b*	1	7/8	7/8	7/8	0.0122(5)
Na2	8*c*	1	0.2570(2)	*x*	*x*	0.0138(4)
O1	8*c*	1	0.1144(2)	*x*	*x*	0.0105(6)
O2	24*e*	1	0.1364(3)	0.9071(2)	0.9186(2)	0.0111(4)
