# Supplementary material for: Spin-orbit coupling induced semi-metallic state in the 1/3 hole-doped hyper-kagome Na3Ir3O8
Source: Sci Rep. 2014 Oct 29;4:6818. doi: 10.1038/srep06818 (PMC4212231; doi:10.1038/srep06818)
Supplement: Supplementary Information [file srep06818-s1.pdf]

Supplementary Information for

"Spin-orbit coupling induced semi-metallic state in the 1/3 hole-doped hyper-kagome $Na_3Ir_3O_8$"

Tomohiro Takayama[1,2*], Alexander Yaresko[1], Akiyo Matsumoto[2], Jürgen Nuss[1], Kenji Ishii[3], Masahiro Yoshida[3,4], Junichiro Mizuki[3,4] & Hidenori Takagi[1,2,5]

[1]Max Planck Institute for Solid State Research, Heisenbergstrasse 1, 70569 Stuttgart, Germany
[2]Department of Physics and Department of Advanced Materials, University of Tokyo, 7-3-1 Hongo, Bunkyo-ku, Tokyo 113-0033, Japan
[3]SPring-8, Japan Atomic Energy Agency, Sayo, Hyogo 679-5148, Japan
[4]School of Science and Technology, Kwansei Gakuin University, Sanda, Hyogo 669-1337, Japan
[5]RIKEN Advanced Science Institute, Wako 351-0195, Japan

Structure Determination

Diffraction data were collected at 294 K on a three circle diffractometer (Bruker AXS, Karlsruhe, Germany) equipped with SMART APEX II CCD, using Mo-*K*α radiation (λ = 0.71073 Å). The collection and reduction of data were carried out with the BRUKER SUITE software package [S1]. The intensities were corrected for absorption effects applying a multi-scan method with SADABS [S2]. The structure was solved by Direct Methods and refined by full matrix least-squares fitting with the SHELXTL software package [S3]. The structure was refined as a two-component inversion twin with twin volume fractions close to racemic twinning (0.51(5)/0.49). Experimental details of data collection and crystallographic data are given in Table S1 and S2.

Supplementary Table S1: Crystal data and structure refinement data of $Na_3Ir_3O_8$.

	$Na_3Ir_3O_8$
Temperature /K	294(2)
Formula weight	773.57
Space group (no.), Z	$P4_132$ (213), 4
Lattice constants /Å	a = 8.9857(4)
V /Å^3, ρ_{xray} /g cm^{-3}	725.5(1), 7.082
Crystal size /mm^{-3}	0.08×0.06×0.04
Diffractometer	SMART APEX II, Bruker AXS
X-ray radiation, λ/Å	0.71073
Absorption correction	Multi-scan, SADABS[S2]
2θ range /°	6.4-72.6
Index range	$-14 \leq h \leq 14$
	$-14 \leq k \leq 14$
	$-14 \leq l \leq 14$
Reflection collected	13526
Data, R_{int}	593, 0.045
No. of parameters	24
Transmission:t_{max}, t_{min}	0.166, 0.073
$R1[F^2 > 2\sigma(F^2)]$	0.012
$wR(F^2)$	0.029
Absolute structure parameter	0.51(5)
$\Delta\rho_{max}$, $\Delta\rho_{min}$ /e Å^{-3}	0.893, –0.606
Deposition no.[S4]	CSD-426992

Supplementary Table S2. Atomic coordinates and displacement parameters U_{ij}/Å^2×10^4 for $Na_3Ir_3O_8$ at 294 K.

Atom	site	*x*	*y*	*z*
Ir	12*d*	0.61264(2)	*x*+ 1/4	5/8
Na1	4*b*	7/8	7/8	7/8
Na2	8*c*	0.2570(2)	*x*	*x*
O1	8*c*	0.1144(3)	*x*	*x*
O2	24*e*	0.1364(3)	0.9071(3)	0.9186(3)

Atom	U_{11}	U_{22}	U_{33}	U_{12}	U_{13}	U_{23}	U_{eq}
Ir	78.56(6)	U_{11}	83.5(8)	4.1(5)	-2.3(4)	-U_{13}	80.2(5)
Na1	122(6)	U_{11}	U_{11}	-18(8)	U_{12}	U_{12}	122(6)
Na2	138(5)	U_{11}	U_{11}	-2(6)	U_{12}	U_{12}	138(5)
O1	105(7)	U_{11}	U_{11}	2(9)	U_{12}	U_{12}	105(7)
O2	146(12)	103(11)	84(10)	-2(9)	13(9)	-1(8)	111(5)

Supplementary References:

[S1] *Bruker Suite*, Version 2008/3, Bruker AXS Inc., Madison USA **2008**.

[S2] G. M. Sheldrick, SADABS — *Bruker AXS area detector scaling and absorption*, Version 2008/1, University of Gottingen, Germany **2008**.

[S3] G. M. Sheldrick, *Acta Crystallogr., Sect. A* **2008**, *64*, 112-122.

[S4] Further details may be obtained from Fachinformationszentrum Karlsruhe, 76344 Eggenstein-Leopoldshafen, Germany (fax: (+49)-7247-808-666; e-mail: crysdata(at)fiz-karlsruhe.de,http://www.fiz-karlsruhe.de/request for deposited data.html) on quoting the CSD number (426992).
