# Supplementary material for: Spin-orbit coupling induced semi-metallic state in the 1/3 hole-doped hyper-kagome Na3Ir3O8
Source: Sci Rep. 2014 Oct 29;4:6818. doi: 10.1038/srep06818 (PMC4212231; doi:10.1038/srep06818)
Supplement: Supplementary Information — Dataset 1 [file srep06818-s2.doc]

data_publication_text

_ccdc_journal_manuscript_code 'Scientific Reports'

_publ_contact_author_name 'Tomohiro Takayama'

_publ_contact_author_address

;Max Planck Institute for Solid State Research

Heisenbergstrasse 1

70569 Stuttgart

Germany

;

_publ_contact_author_email t.takayama@fkf.mpg.de

_publ_contact_author_phone '+49(0)711 6891502'

_publ_contact_author_fax '+49(0)711 6891501'

loop_

_publ_author_name

_publ_author_address

'Takayama, Tomohiro'

;Max Planck Institute for Solid State Research

Heisenbergstrasse 1

70569 Stuttgart

Germany

;

'Matsumoto, Akiyo'

;Department of Physics of Advanced Materials

University of Tokyo

7-3-1 Hongo, Bunkyo-ku

113-0033 Tokyo

Japan

;

'Nuss, J\"urgen'

;Max Planck Institute for Solid State Research

Heisenbergstrasse 1

70569 Stuttgart

Germany

;

'Yaresko, Alexander'

;Max Planck Institute for Solid State Research

Heisenbergstrasse 1

70569 Stuttgart

Germany

;

'Ishii, Kenji'

;SPring-8

Japan Atomic Agency

Sayo

669-1337 Hyogo

Japan

;

'Yoshita, Masahiro'

;SPring-8

Japan Atomic Agency

Sayo

669-1337 Hyogo

Japan

;

'Mizuki, Junichiro'

;SPring-8

Japan Atomic Agency

Sayo

669-1337 Hyogo

Japan

;

'Takagi, Hidenori'

;Max Planck Institute for Solid State Research

Heisenbergstrasse 1

70569 Stuttgart

Germany

;

data_tak4_0m

_audit_creation_method SHELXL-2013

_chemical_name_systematic ?

_chemical_name_common ?

_chemical_formula_moiety 'Ir3 O8, 3(Na)'

_chemical_formula_sum 'Ir3 Na3 O8'

_chemical_melting_point ?

_exptl_crystal_description block

_exptl_crystal_colour black

_diffrn_ambient_temperature 294(2)

_chemical_formula_weight 773.57

loop_

_atom_type_symbol

_atom_type_description

_atom_type_scat_dispersion_real

_atom_type_scat_dispersion_imag

_atom_type_scat_source

O O 0.0106 0.0060 'International Tables Vol C Tables 4.2.6.8 and 6.1.1.4'

Na Na 0.0362 0.0249 'International Tables Vol C Tables 4.2.6.8 and 6.1.1.4'

Ir Ir -1.4442 7.9887 'International Tables Vol C Tables 4.2.6.8 and 6.1.1.4'

_space_group_crystal_system cubic

_space_group_IT_number 213

_space_group_name_H-M_alt 'P 41 3 2'

_space_group_name_Hall 'P 4bd 2ab 3'

_shelx_space_group_comment

;

The symmetry employed for this shelxl refinement is uniquely defined

by the following loop, which should always be used as a source of

symmetry information in preference to the above space-group names.

They are only intended as comments.

;

loop_

_space_group_symop_operation_xyz

'x, y, z'

'-x+1/2, -y, z+1/2'

'-x, y+1/2, -z+1/2'

'x+1/2, -y+1/2, -z'

'z, x, y'

'z+1/2, -x+1/2, -y'

'-z+1/2, -x, y+1/2'

'-z, x+1/2, -y+1/2'

'y, z, x'

'-y, z+1/2, -x+1/2'

'y+1/2, -z+1/2, -x'

'-y+1/2, -z, x+1/2'

'y+3/4, x+1/4, -z+1/4'

'-y+3/4, -x+3/4, -z+3/4'

'y+1/4, -x+1/4, z+3/4'

'-y+1/4, x+3/4, z+1/4'

'x+3/4, z+1/4, -y+1/4'

'-x+1/4, z+3/4, y+1/4'

'-x+3/4, -z+3/4, -y+3/4'

'x+1/4, -z+1/4, y+3/4'

'z+3/4, y+1/4, -x+1/4'

'z+1/4, -y+1/4, x+3/4'

'-z+1/4, y+3/4, x+1/4'

'-z+3/4, -y+3/4, -x+3/4'

_cell_length_a 8.9857(4)

_cell_length_b 8.9857(4)

_cell_length_c 8.9857(4)

_cell_angle_alpha 90

_cell_angle_beta 90

_cell_angle_gamma 90

_cell_volume 725.53(10)

_cell_formula_units_Z 4

_cell_measurement_temperature 294(2)

_cell_measurement_reflns_used 6073

_cell_measurement_theta_min 3.2

_cell_measurement_theta_max 36.3

_exptl_crystal_density_meas ?

_exptl_crystal_density_method ?

_exptl_crystal_density_diffrn 7.082

_exptl_crystal_F_000 1312

_exptl_transmission_factor_min ?

_exptl_transmission_factor_max ?

_exptl_crystal_size_max 0.080

_exptl_crystal_size_mid 0.060

_exptl_crystal_size_min 0.040

_exptl_absorpt_coefficient_mu 55.081

_shelx_estimated_absorpt_T_min 0.096

_shelx_estimated_absorpt_T_max 0.217

_exptl_absorpt_correction_type multi-scan

_exptl_absorpt_correction_T_min 0.073

_exptl_absorpt_correction_T_max 0.166

_exptl_absorpt_process_details

;

semi empirical absorption correction with SADABS,

G.M. Sheldrick, SADABS, Version 2007/4, University of G\"ottingen. Germany.

;

_exptl_special_details

;

?

;

_diffrn_radiation_probe x-ray

_diffrn_radiation_type MoK\a

_diffrn_radiation_wavelength 0.71073

_diffrn_source 'fine-focus sealed tube'

_diffrn_measurement_device_type 'SMART APEX II, Bruker AXS'

_diffrn_measurement_method \wscan

_diffrn_detector_area_resol_mean ?

_diffrn_reflns_number 13526

_diffrn_reflns_av_unetI/netI 0.0142

_diffrn_reflns_av_R_equivalents 0.0453

_diffrn_reflns_limit_h_min -14

_diffrn_reflns_limit_h_max 14

_diffrn_reflns_limit_k_min -14

_diffrn_reflns_limit_k_max 14

_diffrn_reflns_limit_l_min -14

_diffrn_reflns_limit_l_max 14

_diffrn_reflns_theta_min 3.206

_diffrn_reflns_theta_max 36.292

_diffrn_reflns_theta_full 25.242

_diffrn_measured_fraction_theta_max 1.000

_diffrn_measured_fraction_theta_full 0.994

_diffrn_reflns_Laue_measured_fraction_max 1.000

_diffrn_reflns_Laue_measured_fraction_full 0.994

_diffrn_reflns_point_group_measured_fraction_max 1.000

_diffrn_reflns_point_group_measured_fraction_full 0.996

_reflns_number_total 593

_reflns_number_gt 577

_reflns_threshold_expression 'I > 2\s(I)'

_reflns_Friedel_coverage 0.497

_reflns_Friedel_fraction_max 1.000

_reflns_Friedel_fraction_full 1.000

_reflns_special_details

;

Reflections were merged by SHELXL according to the crystal

class for the calculation of statistics and refinement.

_reflns_Friedel_fraction is defined as the number of unique

Friedel pairs measured divided by the number that would be

possible theoretically, ignoring centric projections and

systematic absences.

;

_computing_data_collection 'Bruker Suite (Bruker AXS)'

_computing_cell_refinement 'Bruker Suite (Bruker AXS)'

_computing_data_reduction 'Bruker Suite (Bruker AXS)'

_computing_structure_solution 'SHELXL-2013 (Sheldrick, 2013)'

_computing_structure_refinement 'SHELXL-2013 (Sheldrick, 2013)'

_computing_molecular_graphics ?

_computing_publication_material ?

_refine_special_details

;

Refined as a 2-component inversion twin.

;

_refine_ls_structure_factor_coef Fsqd

_refine_ls_matrix_type full

_refine_ls_weighting_scheme calc

_refine_ls_weighting_details

;

w=1/[\s^2^(Fo^2^)+(0.0105P)^2^+1.1837P]

where P=(Fo^2^+2Fc^2^)/3

;

_atom_sites_solution_primary direct

_atom_sites_solution_secondary difmap

_refine_ls_extinction_method none

_refine_ls_extinction_coef .

_refine_ls_abs_structure_details

;

Refined as an inversion twin.

;

_refine_ls_abs_structure_Flack 0.51(5)

_symmetry_cell_setting cubic

_symmetry_space_group_name_h-m 'P 41 3 2'

_symmetry_int_tables_number 213

_chemical_absolute_configuration syn

_refine_ls_number_reflns 593

_refine_ls_number_parameters 24

_refine_ls_number_restraints 0

_refine_ls_R_factor_all 0.0133

_refine_ls_R_factor_gt 0.0124

_refine_ls_wR_factor_ref 0.0287

_refine_ls_wR_factor_gt 0.0285

_refine_ls_goodness_of_fit_ref 1.193

_refine_ls_restrained_S_all 1.193

_refine_ls_shift/su_max 0.001

_refine_ls_shift/su_mean 0.000

loop_

_atom_site_label

_atom_site_type_symbol

_atom_site_fract_x

_atom_site_fract_y

_atom_site_fract_z

_atom_site_U_iso_or_equiv

_atom_site_adp_type

_atom_site_occupancy

_atom_site_site_symmetry_order

_atom_site_calc_flag

_atom_site_refinement_flags_posn

_atom_site_refinement_flags_adp

_atom_site_refinement_flags_occupancy

_atom_site_disorder_assembly

_atom_site_disorder_group

Ir Ir 0.61264(2) 0.86264(2) 0.6250 0.00802(5) Uani 1 2 d S T P . .

Na1 Na 0.8750 0.8750 0.8750 0.0122(6) Uani 1 6 d S T P . .

Na2 Na 0.2570(2) 0.2570(2) 0.2570(2) 0.0138(5) Uani 1 3 d S T P . .

O1 O 0.1144(3) 0.1144(3) 0.1144(3) 0.0105(7) Uani 1 3 d S T P . .

O2 O 0.1364(3) 0.9071(3) 0.9186(3) 0.0111(5) Uani 1 1 d . . . . .

loop_

_atom_site_aniso_label

_atom_site_aniso_U_11

_atom_site_aniso_U_22

_atom_site_aniso_U_33

_atom_site_aniso_U_23

_atom_site_aniso_U_13

_atom_site_aniso_U_12

Ir 0.00785(6) 0.00785(6) 0.00835(8) 0.00023(4) -0.00023(4) 0.00041(5)

Na1 0.0122(6) 0.0122(6) 0.0122(6) -0.0018(8) -0.0018(8) -0.0018(8)

Na2 0.0138(5) 0.0138(5) 0.0138(5) -0.0002(6) -0.0002(6) -0.0002(6)

O1 0.0105(7) 0.0105(7) 0.0105(7) 0.0002(9) 0.0002(9) 0.0002(9)

O2 0.0147(12) 0.0103(11) 0.0084(10) -0.0001(8) 0.0013(10) -0.0002(9)

_geom_special_details

;

All esds (except the esd in the dihedral angle between two l.s. planes)

are estimated using the full covariance matrix. The cell esds are taken

into account individually in the estimation of esds in distances, angles

and torsion angles; correlations between esds in cell parameters are only

used when they are defined by crystal symmetry. An approximate (isotropic)

treatment of cell esds is used for estimating esds involving l.s. planes.

;

loop_

_geom_bond_atom_site_label_1

_geom_bond_atom_site_label_2

_geom_bond_distance

_geom_bond_site_symmetry_2

_geom_bond_publ_flag

Ir O2 1.976(3) 12_675 ?

Ir O2 1.976(3) 24_665 ?

Ir O2 1.978(3) 7_664 ?

Ir O2 1.978(3) 19_566 ?

Ir O1 2.053(2) 2_565 ?

Ir O1 2.053(2) 14 ?

Ir Ir 3.10138(16) 11_466 ?

Ir Ir 3.10138(15) 5 ?

Ir Ir 3.10138(15) 9 ?

Ir Ir 3.10138(17) 8_656 ?

Ir Na1 3.25830(17) . ?

Ir Na1 3.25830(18) 2_674 ?

Na1 O2 2.399(3) 24_665 ?

Na1 O2 2.399(3) 14_656 ?

Na1 O2 2.399(3) 9_556 ?

Na1 O2 2.399(3) 5_565 ?

Na1 O2 2.399(3) 1_655 ?

Na1 O2 2.399(3) 19_566 ?

Na1 Ir 3.25829(17) 10_756 ?

Na1 Ir 3.25829(17) 2_675 ?

Na1 Ir 3.25829(17) 6_567 ?

Na1 Ir 3.25829(17) 9 ?

Na1 Ir 3.25829(17) 5 ?

Na2 O1 2.220(6) . ?

Na2 O2 2.280(3) 10_645 ?

Na2 O2 2.280(3) 6_456 ?

Na2 O2 2.280(3) 2_564 ?

Na2 Na1 3.6692(16) 3_646 ?

Na2 Na1 3.6692(16) 2_664 ?

Na2 Na1 3.6692(16) 4_466 ?

Na2 Na2 3.672(6) 14 ?

Na2 Ir 3.688(3) 6_456 ?

Na2 Ir 3.688(3) 10_645 ?

Na2 Ir 3.688(3) 2_564 ?

Na2 Na2 3.965(2) 16_545 ?

O1 Ir 2.053(2) 2_564 ?

O1 Ir 2.053(2) 10_645 ?

O1 Ir 2.053(2) 6_456 ?

O2 Ir 1.976(3) 6_467 ?

O2 Ir 1.978(3) 10_656 ?

O2 Na2 2.280(3) 2_565 ?

O2 Na1 2.399(3) 1_455 ?

loop_

_geom_angle_atom_site_label_1

_geom_angle_atom_site_label_2

_geom_angle_atom_site_label_3

_geom_angle

_geom_angle_site_symmetry_1

_geom_angle_site_symmetry_3

_geom_angle_publ_flag

O2 Ir O2 100.42(16) 12_675 24_665 ?

O2 Ir O2 91.99(17) 12_675 7_664 ?

O2 Ir O2 95.16(11) 24_665 7_664 ?

O2 Ir O2 95.16(11) 12_675 19_566 ?

O2 Ir O2 91.99(17) 24_665 19_566 ?

O2 Ir O2 168.83(17) 7_664 19_566 ?

O2 Ir O1 79.24(11) 12_675 2_565 ?

O2 Ir O1 174.32(12) 24_665 2_565 ?

O2 Ir O1 79.20(11) 7_664 2_565 ?

O2 Ir O1 93.69(12) 19_566 2_565 ?

O2 Ir O1 174.32(12) 12_675 14 ?

O2 Ir O1 79.24(11) 24_665 14 ?

O2 Ir O1 93.69(12) 7_664 14 ?

O2 Ir O1 79.20(11) 19_566 14 ?

O1 Ir O1 101.66(17) 2_565 14 ?

O2 Ir Ir 86.29(8) 12_675 11_466 ?

O2 Ir Ir 133.46(8) 24_665 11_466 ?

O2 Ir Ir 38.33(8) 7_664 11_466 ?

O2 Ir Ir 133.60(8) 19_566 11_466 ?

O1 Ir Ir 40.93(7) 2_565 11_466 ?

O1 Ir Ir 98.07(11) 14 11_466 ?

O2 Ir Ir 133.46(8) 12_675 5 ?

O2 Ir Ir 86.29(8) 24_665 5 ?

O2 Ir Ir 133.60(8) 7_664 5 ?

O2 Ir Ir 38.33(8) 19_566 5 ?

O1 Ir Ir 98.07(11) 2_565 5 ?

O1 Ir Ir 40.93(7) 14 5 ?

Ir Ir Ir 121.732(2) 11_466 5 ?

O2 Ir Ir 138.72(8) 12_675 9 ?

O2 Ir Ir 38.38(8) 24_665 9 ?

O2 Ir Ir 94.02(8) 7_664 9 ?

O2 Ir Ir 86.26(8) 19_566 9 ?

O1 Ir Ir 141.95(7) 2_565 9 ?

O1 Ir Ir 40.93(7) 14 9 ?

Ir Ir Ir 121.645(2) 11_466 9 ?

Ir Ir Ir 60.0 5 9 ?

O2 Ir Ir 38.38(8) 12_675 8_656 ?

O2 Ir Ir 138.72(8) 24_665 8_656 ?

O2 Ir Ir 86.26(8) 7_664 8_656 ?

O2 Ir Ir 94.02(8) 19_566 8_656 ?

O1 Ir Ir 40.93(7) 2_565 8_656 ?

O1 Ir Ir 141.95(7) 14 8_656 ?

Ir Ir Ir 60.0 11_466 8_656 ?

Ir Ir Ir 121.645(2) 5 8_656 ?

Ir Ir Ir 177.098(3) 9 8_656 ?

O2 Ir Na1 89.72(8) 12_675 . ?

O2 Ir Na1 47.10(8) 24_665 . ?

O2 Ir Na1 141.72(8) 7_664 . ?

O2 Ir Na1 47.11(9) 19_566 . ?

O1 Ir Na1 138.39(6) 2_565 . ?

O1 Ir Na1 85.93(11) 14 . ?

Ir Ir Na1 175.994(5) 11_466 . ?

Ir Ir Na1 61.581(2) 5 . ?

Ir Ir Na1 61.581(2) 9 . ?

Ir Ir Na1 116.666(4) 8_656 . ?

O2 Ir Na1 47.10(8) 12_675 2_674 ?

O2 Ir Na1 89.72(8) 24_665 2_674 ?

O2 Ir Na1 47.11(9) 7_664 2_674 ?

O2 Ir Na1 141.72(8) 19_566 2_674 ?

O1 Ir Na1 85.93(11) 2_565 2_674 ?

O1 Ir Na1 138.39(6) 14 2_674 ?

Ir Ir Na1 61.581(2) 11_466 2_674 ?

Ir Ir Na1 175.994(5) 5 2_674 ?

Ir Ir Na1 116.666(4) 9 2_674 ?

Ir Ir Na1 61.581(2) 8_656 2_674 ?

Na1 Ir Na1 115.215(5) . 2_674 ?

O2 Na1 O2 72.72(10) 24_665 14_656 ?

O2 Na1 O2 176.50(13) 24_665 9_556 ?

O2 Na1 O2 109.52(13) 14_656 9_556 ?

O2 Na1 O2 105.18(13) 24_665 5_565 ?

O2 Na1 O2 176.50(13) 14_656 5_565 ?

O2 Na1 O2 72.72(10) 9_556 5_565 ?

O2 Na1 O2 109.52(13) 24_665 1_655 ?

O2 Na1 O2 105.18(13) 14_656 1_655 ?

O2 Na1 O2 72.72(10) 9_556 1_655 ?

O2 Na1 O2 72.72(10) 5_565 1_655 ?

O2 Na1 O2 72.72(10) 24_665 19_566 ?

O2 Na1 O2 72.72(10) 14_656 19_566 ?

O2 Na1 O2 105.18(13) 9_556 19_566 ?

O2 Na1 O2 109.52(13) 5_565 19_566 ?

O2 Na1 O2 176.50(13) 1_655 19_566 ?

O2 Na1 Ir 103.55(6) 24_665 10_756 ?

O2 Na1 Ir 140.14(6) 14_656 10_756 ?

O2 Na1 Ir 76.54(6) 9_556 10_756 ?

O2 Na1 Ir 37.12(7) 5_565 10_756 ?

O2 Na1 Ir 37.17(6) 1_655 10_756 ?

O2 Na1 Ir 145.61(6) 19_566 10_756 ?

O2 Na1 Ir 140.14(6) 24_665 2_675 ?

O2 Na1 Ir 145.61(6) 14_656 2_675 ?

O2 Na1 Ir 37.12(6) 9_556 2_675 ?

O2 Na1 Ir 37.17(6) 5_565 2_675 ?

O2 Na1 Ir 76.54(6) 1_655 2_675 ?

O2 Na1 Ir 103.55(6) 19_566 2_675 ?

Ir Na1 Ir 56.839(4) 10_756 2_675 ?

O2 Na1 Ir 145.61(7) 24_665 6_567 ?

O2 Na1 Ir 103.55(6) 14_656 6_567 ?

O2 Na1 Ir 37.17(6) 9_556 6_567 ?

O2 Na1 Ir 76.54(6) 5_565 6_567 ?

O2 Na1 Ir 37.12(6) 1_655 6_567 ?

O2 Na1 Ir 140.14(6) 19_566 6_567 ?

Ir Na1 Ir 56.839(4) 10_756 6_567 ?

Ir Na1 Ir 56.839(4) 2_675 6_567 ?

O2 Na1 Ir 37.17(6) 24_665 9 ?

O2 Na1 Ir 37.12(6) 14_656 9 ?

O2 Na1 Ir 145.61(6) 9_556 9 ?

O2 Na1 Ir 140.14(6) 5_565 9 ?

O2 Na1 Ir 103.55(6) 1_655 9 ?

O2 Na1 Ir 76.54(6) 19_566 9 ?

Ir Na1 Ir 121.645(2) 10_756 9 ?

Ir Na1 Ir 177.238(3) 2_675 9 ?

Ir Na1 Ir 124.785(5) 6_567 9 ?

O2 Na1 Ir 76.54(6) 24_665 5 ?

O2 Na1 Ir 37.17(6) 14_656 5 ?

O2 Na1 Ir 103.55(6) 9_556 5 ?

O2 Na1 Ir 145.61(7) 5_565 5 ?

O2 Na1 Ir 140.14(6) 1_655 5 ?

O2 Na1 Ir 37.12(6) 19_566 5 ?

Ir Na1 Ir 177.238(3) 10_756 5 ?

Ir Na1 Ir 124.785(5) 2_675 5 ?

Ir Na1 Ir 121.645(2) 6_567 5 ?

Ir Na1 Ir 56.839(4) 9 5 ?

O2 Na1 Ir 37.12(7) 24_665 . ?

O2 Na1 Ir 76.54(6) 14_656 . ?

O2 Na1 Ir 140.14(6) 9_556 . ?

O2 Na1 Ir 103.55(6) 5_565 . ?

O2 Na1 Ir 145.61(6) 1_655 . ?

O2 Na1 Ir 37.17(6) 19_566 . ?

Ir Na1 Ir 124.785(5) 10_756 . ?

Ir Na1 Ir 121.645(2) 2_675 . ?

Ir Na1 Ir 177.238(3) 6_567 . ?

Ir Na1 Ir 56.839(4) 9 . ?

Ir Na1 Ir 56.839(4) 5 . ?

O1 Na2 O2 103.68(11) . 10_645 ?

O1 Na2 O2 103.68(11) . 6_456 ?

O2 Na2 O2 114.59(8) 10_645 6_456 ?

O1 Na2 O2 103.68(11) . 2_564 ?

O2 Na2 O2 114.59(8) 10_645 2_564 ?

O2 Na2 O2 114.59(8) 6_456 2_564 ?

O1 Na2 Na1 120.02(4) . 3_646 ?

O2 Na2 Na1 39.52(8) 10_645 3_646 ?

O2 Na2 Na1 131.87(13) 6_456 3_646 ?

O2 Na2 Na1 75.45(8) 2_564 3_646 ?

O1 Na2 Na1 120.02(4) . 2_664 ?

O2 Na2 Na1 131.87(13) 10_645 2_664 ?

O2 Na2 Na1 75.45(8) 6_456 2_664 ?

O2 Na2 Na1 39.52(8) 2_564 2_664 ?

Na1 Na2 Na1 97.15(6) 3_646 2_664 ?

O1 Na2 Na1 120.02(4) . 4_466 ?

O2 Na2 Na1 75.45(8) 10_645 4_466 ?

O2 Na2 Na1 39.52(8) 6_456 4_466 ?

O2 Na2 Na1 131.87(13) 2_564 4_466 ?

Na1 Na2 Na1 97.15(6) 3_646 4_466 ?

Na1 Na2 Na1 97.15(6) 2_664 4_466 ?

O1 Na2 Na2 180.0(3) . 14 ?

O2 Na2 Na2 76.32(11) 10_645 14 ?

O2 Na2 Na2 76.32(11) 6_456 14 ?

O2 Na2 Na2 76.32(11) 2_564 14 ?

Na1 Na2 Na2 59.98(4) 3_646 14 ?

Na1 Na2 Na2 59.98(4) 2_664 14 ?

Na1 Na2 Na2 59.98(4) 4_466 14 ?

O1 Na2 Ir 29.05(2) . 6_456 ?

O2 Na2 Ir 119.20(11) 10_645 6_456 ?

O2 Na2 Ir 113.23(10) 6_456 6_456 ?

O2 Na2 Ir 74.82(9) 2_564 6_456 ?

Na1 Na2 Ir 114.77(3) 3_646 6_456 ?

Na1 Na2 Ir 94.75(2) 2_664 6_456 ?

Na1 Na2 Ir 144.13(6) 4_466 6_456 ?

Na2 Na2 Ir 150.95(2) 14 6_456 ?

O1 Na2 Ir 29.05(2) . 10_645 ?

O2 Na2 Ir 113.23(10) 10_645 10_645 ?

O2 Na2 Ir 74.82(9) 6_456 10_645 ?

O2 Na2 Ir 119.20(11) 2_564 10_645 ?

Na1 Na2 Ir 144.13(6) 3_646 10_645 ?

Na1 Na2 Ir 114.77(3) 2_664 10_645 ?

Na1 Na2 Ir 94.75(2) 4_466 10_645 ?

Na2 Na2 Ir 150.95(2) 14 10_645 ?

Ir Na2 Ir 49.73(4) 6_456 10_645 ?

O1 Na2 Ir 29.05(2) . 2_564 ?

O2 Na2 Ir 74.82(9) 10_645 2_564 ?

O2 Na2 Ir 119.20(11) 6_456 2_564 ?

O2 Na2 Ir 113.23(10) 2_564 2_564 ?

Na1 Na2 Ir 94.75(2) 3_646 2_564 ?

Na1 Na2 Ir 144.13(6) 2_664 2_564 ?

Na1 Na2 Ir 114.77(3) 4_466 2_564 ?

Na2 Na2 Ir 150.95(2) 14 2_564 ?

Ir Na2 Ir 49.73(4) 6_456 2_564 ?

Ir Na2 Ir 49.73(4) 10_645 2_564 ?

O1 Na2 Na2 69.79(2) . 16_545 ?

O2 Na2 Na2 74.68(10) 10_645 16_545 ?

O2 Na2 Na2 170.16(8) 6_456 16_545 ?

O2 Na2 Na2 61.58(7) 2_564 16_545 ?

Na1 Na2 Na2 57.29(4) 3_646 16_545 ?

Na1 Na2 Na2 101.09(3) 2_664 16_545 ?

Na1 Na2 Na2 150.060(16) 4_466 16_545 ?

Na2 Na2 Na2 110.21(2) 14 16_545 ?

Ir Na2 Na2 57.477(8) 6_456 16_545 ?

Ir Na2 Na2 98.83(5) 10_645 16_545 ?

Ir Na2 Na2 58.53(3) 2_564 16_545 ?

Ir O1 Ir 98.13(15) 2_564 10_645 ?

Ir O1 Ir 98.13(15) 2_564 6_456 ?

Ir O1 Ir 98.13(15) 10_645 6_456 ?

Ir O1 Na2 119.27(11) 2_564 . ?

Ir O1 Na2 119.27(11) 10_645 . ?

Ir O1 Na2 119.27(11) 6_456 . ?

Ir O2 Ir 103.29(12) 6_467 10_656 ?

Ir O2 Na2 126.67(14) 6_467 2_565 ?

Ir O2 Na2 123.24(15) 10_656 2_565 ?

Ir O2 Na1 95.78(11) 6_467 1_455 ?

Ir O2 Na1 95.73(11) 10_656 1_455 ?

Na2 O2 Na1 103.27(12) 2_565 1_455 ?

_refine_diff_density_max 0.893

_refine_diff_density_min -0.606

_refine_diff_density_rms 0.185

_shelxl_version_number 2013-2

_shelx_res_file

;

TITL tak4_0m in P4(1)32 Na3Ir3O8 metallic at 293K

CELL 0.71073 8.9857 8.9857 8.9857 90.000 90.000 90.000

ZERR 4.00 0.0004 0.0004 0.0004 0.000 0.000 0.000

LATT -1

SYMM 0.5-X, -Y, 0.5+Z

SYMM -X, 0.5+Y, 0.5-Z

SYMM 0.5+X, 0.5-Y, -Z

SYMM Z, X, Y

SYMM 0.5+Z, 0.5-X, -Y

SYMM 0.5-Z, -X, 0.5+Y

SYMM -Z, 0.5+X, 0.5-Y

SYMM Y, Z, X

SYMM -Y, 0.5+Z, 0.5-X

SYMM 0.5+Y, 0.5-Z, -X

SYMM 0.5-Y, -Z, 0.5+X

SYMM 0.75+Y, 0.25+X, 0.25-Z

SYMM 0.75-Y, 0.75-X, 0.75-Z

SYMM 0.25+Y, 0.25-X, 0.75+Z

SYMM 0.25-Y, 0.75+X, 0.25+Z

SYMM 0.75+X, 0.25+Z, 0.25-Y

SYMM 0.25-X, 0.75+Z, 0.25+Y

SYMM 0.75-X, 0.75-Z, 0.75-Y

SYMM 0.25+X, 0.25-Z, 0.75+Y

SYMM 0.75+Z, 0.25+Y, 0.25-X

SYMM 0.25+Z, 0.25-Y, 0.75+X

SYMM 0.25-Z, 0.75+Y, 0.25+X

SYMM 0.75-Z, 0.75-Y, 0.75-X

SFAC O NA IR

UNIT 32 12 12

TEMP 20.670

SIZE 0.04 0.06 0.08

ACTA

TWIN

L.S. 4

BOND

FMAP 2

PLAN 20

WGHT 0.010500 1.183700

BASF 0.51245

FVAR 0.12118

IR 3 0.612640 0.862640 0.625000 10.50000 0.00785 0.00785 =

0.00835 0.00023 -0.00023 0.00041

NA1 2 0.875000 0.875000 0.875000 10.16667 0.01222 0.01222 =

0.01222 -0.00183 -0.00183 -0.00183

NA2 2 0.257042 0.257042 0.257042 10.33333 0.01382 0.01382 =

0.01382 -0.00019 -0.00019 -0.00019

O1 1 0.114391 0.114391 0.114391 10.33333 0.01049 0.01049 =

0.01049 0.00020 0.00020 0.00020

O2 1 0.136444 0.907057 0.918585 11.00000 0.01467 0.01026 =

0.00844 -0.00007 0.00133 -0.00018

HKLF 4

REM tak4_0m in P4(1)32 Na3Ir3O8 metallic at 293K

REM R1 = 0.0124 for 577 Fo > 4sig(Fo) and 0.0133 for all 593 data

REM 24 parameters refined using 0 restraints

END

WGHT 0.0090 1.3263

REM Highest difference peak 0.893, deepest hole -0.606, 1-sigma level 0.185

Q1 1 0.5604 0.9092 0.5681 11.00000 0.05 0.89

Q2 1 0.6582 0.9152 0.6818 11.00000 0.05 0.85

Q3 1 0.9042 0.8247 0.9077 11.00000 0.05 0.84

Q4 1 0.8184 0.8750 0.9316 10.50000 0.05 0.76

Q5 1 0.5773 0.8078 0.5856 11.00000 0.05 0.75

Q6 1 0.6381 0.8045 0.5715 11.00000 0.05 0.67

Q7 1 -0.0021 0.9979 0.9979 10.33333 0.05 0.59

Q8 1 0.1289 0.0852 0.0177 11.00000 0.05 0.52

Q9 1 0.1378 1.0027 0.9566 11.00000 0.05 0.52

Q10 1 0.0875 0.1268 0.0166 11.00000 0.05 0.49

Q11 1 0.1816 0.8992 1.0078 11.00000 0.05 0.45

Q12 1 0.0412 0.9215 0.9162 11.00000 0.05 0.42

Q13 1 0.0949 0.9131 0.9747 11.00000 0.05 0.42

Q14 1 0.7644 0.8734 0.8926 11.00000 0.05 0.42

Q15 1 0.1618 0.1618 0.1618 10.33333 0.05 0.42

Q16 1 0.0792 0.9460 0.8741 11.00000 0.05 0.41

Q17 1 0.2423 0.8384 0.9925 11.00000 0.05 0.40

Q18 1 0.0583 0.8889 0.8838 11.00000 0.05 0.40

Q19 1 0.1408 0.8711 0.8460 11.00000 0.05 0.40

Q20 1 0.0772 0.8616 0.9284 11.00000 0.05 0.39

;

_shelx_res_checksum 30633

_shelx_hkl_file

;

0 -1 0 0.42957 0.56925 1

1 0 0 1.94644 0.37951 1

0 1 0 -0.2192 0.3503 3

-1 0 0 1.45794 0.55611 2

0 0 -1 0.92918 0.52265 2

0 0 1 0.53858 0.26365 3

0 1 0 0.20009 0.48919 2

1 0 0 0.77406 0.44818 3

0 1 -1 542.229 22.526 3

0 1 1 523.813 22.633 3

-1 -1 0 513.156 23.117 1

1 0 1 516.743 22.664 3

1 1 0 520.363 22.884 3

1 0 -1 539.219 22.906 1

1 0 1 518.911 22.853 1

1 0 -1 509.107 24.486 2

0 -1 -1 524.562 23.000 1

0 -1 1 498.832 23.110 1

1 0 -1 540.456 22.457 3

0 -1 -1 492.878 24.471 2

0 1 -1 474.308 24.434 2

1 -1 0 515.417 22.901 1

1 1 -1 2996.87 126.90 3

1 -1 1 2894.27 129.03 1

1 -1 1 2903.63 126.77 3

1 1 -1 3037.86 137.07 2

-1 -1 -1 2800.29 136.84 2

-1 -1 -1 2943.10 129.34 1

-1 1 1 3004.26 127.35 3

1 -1 -1 3024.07 125.92 1

-1 1 -1 2839.41 133.74 2

1 -1 -1 2780.45 133.62 2

1 1 1 2826.55 124.08 3

0 -2 0 -3.2403 1.8179 1

2 0 0 -1.9628 1.2229 1

2 0 0 3.95250 1.63144 3

0 2 0 -1.3205 1.5859 2

0 2 0 -3.1006 1.2910 3

0 0 2 -2.3135 1.1071 3

-2 0 0 4.97391 1.79488 2

0 0 -2 3.32661 1.84301 2

-2 1 0 482.496 23.707 2

-2 -1 0 556.932 23.122 2

2 1 0 482.656 21.989 1

-1 0 -2 473.721 23.603 2

0 2 -1 501.074 21.514 3

2 -1 0 468.244 21.429 3

2 1 0 475.505 22.005 3

0 2 1 463.880 21.692 3

1 0 -2 535.843 21.415 3

1 0 2 483.802 21.599 3

0 -2 1 439.132 22.284 1

0 -2 -1 465.052 23.281 2

0 2 -1 476.796 23.437 2

1 0 -2 515.260 23.726 2

2 -1 0 475.753 21.802 1

1 0 -2 510.869 21.732 1

0 1 -2 483.949 24.250 2

-1 2 0 482.029 21.866 3

0 1 -2 488.739 22.056 3

0 -1 -2 519.683 24.322 2

0 -1 -2 510.452 22.350 1

0 1 2 550.563 22.247 3

1 2 0 527.627 22.634 3

2 0 -1 528.678 22.056 3

-1 -2 0 505.303 23.101 1

2 0 -1 489.245 24.048 2

1 -2 0 478.271 22.580 1

2 0 -1 483.405 22.482 1

2 0 1 509.447 22.434 1

-2 0 -1 487.896 24.136 2

-1 2 0 496.901 24.339 2

0 -1 2 505.454 22.550 1

-1 -1 -2 102.008 5.858 2

-1 1 2 110.219 4.848 3

2 -1 1 97.3013 5.0251 3

1 -1 2 95.9780 4.9804 3

1 1 -2 112.169 5.082 3

-2 -1 -1 96.4099 5.4662 2

2 -1 1 99.1127 4.9678 1

-2 1 1 100.986 6.242 2

-1 -2 -1 97.3143 5.3842 2

2 1 -1 99.9584 5.3925 2

1 2 -1 103.381 5.284 3

2 1 -1 99.4873 5.2525 1

-1 2 1 102.417 6.105 2

-1 -1 -2 106.450 5.361 1

1 1 -2 112.996 5.923 2

1 -2 1 99.5941 5.2128 1

-1 -2 -1 115.506 5.981 1

1 -1 2 100.039 4.748 1

1 1 -2 104.642 5.114 1

1 2 -1 103.462 5.396 2

2 1 -1 101.815 5.270 3

2 -1 -1 102.943 5.102 1

2 1 1 97.2749 5.1964 1

-1 2 -1 104.856 4.870 3

1 -1 -2 114.752 5.000 1

-1 -2 1 99.2192 5.7417 1

1 -1 -2 106.884 6.026 2

1 -2 -1 111.204 5.289 1

2 1 1 85.0452 5.5349 3

-1 1 -2 91.1989 5.9627 2

-1 2 -1 104.877 6.072 2

1 1 2 104.419 5.371 3

-2 1 -1 100.622 6.091 2

2 -1 -1 100.685 4.609 3

1 2 1 102.640 5.630 3

0 2 -2 62.6482 4.8697 2

2 -2 0 67.6476 4.1396 1

-2 -2 0 58.3871 4.5446 1

0 -2 -2 65.3815 4.3385 1

0 -2 2 62.3498 4.6315 1

0 2 2 78.5881 4.4065 3

2 0 -2 66.9352 3.8515 3

2 0 2 61.4740 4.3125 3

2 2 0 71.0879 4.8176 3

2 0 -2 61.5367 4.7387 2

2 0 -2 63.3594 4.0531 1

0 2 -2 62.6645 4.0169 3

-2 0 -2 57.1329 4.8387 2

-2 2 0 72.8204 5.2524 2

0 -2 -2 63.3638 4.7748 2

2 0 2 67.6498 3.9028 1

-2 -2 -1 74.4658 4.0803 2

-1 2 2 73.8068 3.8590 3

1 2 -2 75.3086 4.0634 3

2 1 -2 71.0782 3.9302 3

-1 -2 -2 68.6624 4.6446 1

2 2 -1 72.7429 4.3256 3

-1 -2 -2 75.0860 4.5742 2

2 1 -2 74.0200 4.6028 2

2 -1 2 75.5003 3.6856 1

-2 -2 -1 73.7227 4.5993 1

2 -2 1 72.9312 3.6666 3

-2 2 1 82.9369 5.4453 2

2 -1 2 74.0835 3.9838 3

-2 -1 -2 71.6918 4.5817 2

2 -2 1 69.3716 3.8989 1

1 2 -2 81.4235 4.6499 2

-2 -1 -2 69.3203 4.0732 1

1 -2 2 74.9074 3.9585 1

2 1 -2 71.5980 4.0203 1

-1 2 -2 73.7803 3.9064 3

2 -1 -2 72.7848 3.9781 1

2 -2 -1 74.9241 4.1174 1

2 1 2 80.6270 4.5768 3

1 2 2 76.4443 4.6104 3

-2 1 -2 73.1821 5.0375 2

-2 -2 1 68.1607 4.4589 1

2 2 1 65.3219 4.6369 3

-1 2 -2 74.0427 5.1136 2

-1 -2 2 81.2724 4.6851 1

1 -2 -2 82.2006 4.1487 1

-2 2 -1 77.1276 5.2690 2

1 -2 -2 66.3277 4.7809 2

2 -1 -2 80.9625 3.6472 3

2 -1 -2 77.3661 4.9413 2

-2 2 2 5970.81 254.24 3

2 -2 2 5570.55 254.07 3

2 2 -2 6215.72 254.44 3

2 -2 2 5821.73 258.49 1

2 2 -2 6383.75 274.37 2

-2 -2 -2 5796.51 259.14 1

-2 -2 -2 5617.27 274.07 2

-2 2 -2 5347.66 259.17 2

2 -2 -2 5409.08 258.98 2

-2 -2 2 5712.62 244.31 1

2 2 2 5577.13 240.55 3

2 -2 -2 5812.68 243.99 1

0 0 3 2.34512 0.76040 3

0 0 -3 0.79888 1.23564 2

0 3 0 1.16526 1.12333 2

-3 0 0 3.86009 1.33269 2

3 0 0 2.12814 0.85132 1

0 -3 0 3.36726 1.52580 1

3 0 0 0.32825 1.07298 3

0 3 0 1.01141 0.87340 3

0 3 1 56.8226 3.8262 3

0 3 -1 60.2476 3.6178 3

1 0 -3 64.5107 3.2741 1

1 0 -3 69.7193 4.6591 2

0 3 -1 60.4002 4.2392 2

0 3 1 57.9548 4.1864 2

3 -1 0 58.1951 3.5218 1

3 1 0 54.7703 4.1202 3

-3 1 0 82.4217 4.8822 2

-1 0 -3 61.0944 4.4659 2

1 0 3 65.2536 3.6810 3

0 -3 -1 63.5850 4.3155 1

0 -3 1 52.1220 4.3052 1

1 0 -3 65.4513 3.4148 3

-3 -1 0 64.0754 3.8747 2

3 1 0 63.1873 3.7324 1

3 -1 0 63.8483 3.6793 3

0 3 -2 239.039 10.811 3

3 2 0 222.705 11.566 3

2 0 -3 251.759 10.583 3

2 0 3 213.474 10.441 1

0 3 -2 218.243 12.127 2

2 0 -3 241.955 12.366 2

0 -3 2 203.418 11.535 1

3 -2 0 230.162 10.901 1

2 0 3 231.401 11.093 3

2 0 -3 240.454 10.820 1

-3 -2 0 238.765 11.392 1

-3 2 0 236.571 12.602 2

-2 0 -3 229.467 12.349 2

0 -3 -2 214.267 11.854 2

0 -3 -2 248.073 11.461 1

0 3 2 224.807 11.119 3

3 0 1 56.9854 4.0907 3

-3 0 1 57.6352 4.5370 2

0 1 -3 66.2848 4.5911 2

0 -1 -3 62.3386 3.2278 1

0 1 -3 61.4827 3.5166 3

1 3 0 64.3401 4.1300 3

0 -1 -3 64.5451 4.4894 2

-1 3 0 61.3877 3.3554 3

-1 3 0 60.9596 4.4447 2

-1 -3 0 51.1917 4.3825 1

0 1 3 67.4303 3.6942 3

-3 0 -1 65.0064 4.3789 2

3 0 -1 65.2520 3.6202 3

1 -3 0 60.5788 3.9938 1

1 3 0 74.3136 3.7908 2

3 0 -1 62.9863 3.6971 1

3 0 1 68.0281 3.6556 1

1 1 -3 2998.23 121.63 3

-1 -1 -3 2874.93 131.65 2

1 3 -1 2658.48 131.10 2

-1 -3 -1 2543.99 130.94 2

3 -1 1 2766.73 123.64 1

3 1 -1 2823.32 123.92 1

-1 1 3 3087.77 121.56 3

3 -1 1 2622.01 121.74 3

-1 3 1 2848.75 131.85 2

-3 1 1 3036.88 132.16 2

1 -3 1 2769.47 124.02 1

-1 -3 -1 2889.20 124.66 1

3 1 -1 2925.85 121.96 3

1 1 -3 2805.51 123.63 1

1 1 -3 2877.61 131.73 2

-3 -1 -1 2768.95 131.19 2

-1 -1 -3 2632.18 123.71 1

1 3 -1 2827.86 121.91 3

1 -1 3 2757.97 121.63 3

1 -3 -1 2822.83 126.74 1

-1 3 -1 2921.55 134.63 2

1 3 1 2804.14 124.85 3

-1 1 -3 2679.03 134.63 2

3 -1 -1 2759.55 126.44 1

-3 -1 1 3242.39 134.42 2

-1 -3 1 2880.24 127.29 1

1 1 3 2982.94 124.65 3

-3 1 -1 2935.08 134.63 2

1 -1 -3 3057.07 126.27 1

3 1 1 2857.80 126.57 1

1 -1 -3 2861.25 134.67 2

3 -1 -1 2960.77 124.11 3

-1 3 -1 2897.30 124.15 3

3 1 1 2497.04 124.85 3

2 1 -3 265.537 13.396 2

-3 -2 -1 258.746 12.460 2

3 2 -1 250.210 12.238 1

2 1 -3 253.405 11.874 1

-1 3 2 242.165 11.774 3

-2 -1 -3 242.047 13.156 2

-3 2 1 271.117 14.108 2

3 2 -1 254.195 12.263 3

-1 -3 -2 244.165 12.752 2

-3 -2 -1 249.230 12.349 1

2 1 -3 260.758 11.675 3

1 3 -2 238.171 12.880 2

3 -2 1 252.808 11.592 3

-2 -1 -3 271.510 12.056 1

-1 -3 -2 242.619 12.734 1

2 -1 3 248.250 11.402 1

1 3 -2 266.471 11.995 3

1 -3 2 245.737 12.160 1

3 -2 1 252.529 11.844 1

2 -1 3 237.939 11.835 3

-2 1 3 270.760 11.451 3

-1 3 -2 256.575 11.511 3

2 -1 -3 249.147 13.248 2

-2 1 -3 221.071 13.234 2

2 -1 -3 269.243 11.686 1

2 1 3 250.870 12.108 3

3 2 1 226.592 12.433 3

3 -2 -1 243.808 11.799 1

1 -3 -2 234.435 12.960 2

1 3 2 254.566 12.324 3

-1 -3 2 256.702 12.697 1

-1 3 -2 245.557 13.334 2

-3 2 -1 250.512 13.486 2

1 -3 -2 280.068 12.137 1

0 2 -3 228.194 12.469 2

0 2 -3 238.763 10.892 3

2 -3 0 239.640 11.261 1

3 0 -2 234.094 11.100 1

3 0 2 247.290 10.968 1

-2 3 0 225.569 12.567 2

-2 3 0 234.622 10.341 3

-2 -3 0 252.161 12.037 1

0 -2 -3 227.870 12.321 2

3 0 -2 231.704 12.179 2

0 -2 -3 242.041 11.018 1

0 -2 3 194.072 11.159 1

3 0 -2 237.963 10.668 3

3 0 2 219.807 11.300 3

0 2 3 248.874 11.143 3

2 3 0 246.906 11.692 3

-3 0 -2 216.747 12.243 2

3 -1 2 246.320 11.693 1

-3 -1 -2 230.407 12.883 2

1 -2 3 252.287 11.619 1

3 1 -2 256.142 12.881 2

3 1 -2 261.813 12.105 1

-2 3 1 256.475 13.845 2

-1 -2 -3 256.110 13.182 2

-2 -3 -1 226.732 12.205 2

2 3 -1 247.798 12.211 3

3 -1 2 245.221 11.926 3

3 1 -2 281.657 11.909 3

1 2 -3 258.126 13.309 2

-1 2 3 279.976 11.793 3

1 2 -3 259.769 11.778 3

-1 -2 -3 253.784 12.272 1

2 -3 1 244.358 12.027 1

2 -3 -1 256.073 12.072 1

3 -1 -2 255.540 13.350 2

3 1 2 243.512 11.897 1

1 -2 -3 251.052 13.337 2

3 1 2 239.444 12.484 3

-2 3 -1 243.607 13.503 2

-1 2 -3 262.815 11.725 3

-1 2 -3 226.369 13.385 2

3 -1 -2 255.439 11.901 1

-2 -3 1 240.987 12.676 1

1 2 3 255.592 12.177 3

-1 -2 3 275.052 12.344 1

1 -2 -3 278.896 11.852 1

-2 3 -1 253.453 11.345 3

3 -1 -2 253.999 11.221 3

2 3 1 247.284 12.531 3

-3 1 -2 235.587 13.375 2

-2 -2 -3 35.8262 3.0209 1

2 -2 3 33.8287 2.5658 3

-2 3 2 31.5166 2.2921 3

-2 -2 -3 33.1539 3.0433 2

-2 -3 -2 39.7650 3.4569 1

-3 -2 -2 31.9882 2.8911 1

-2 2 3 38.9325 2.3010 3

2 -2 3 32.4741 2.1411 1

3 2 -2 34.5669 2.8661 1

2 3 -2 38.0750 2.9876 3

-3 2 2 36.3899 3.8702 2

-2 -3 -2 32.3491 2.7687 2

-3 -2 -2 37.1754 2.9877 2

3 -2 2 33.4101 2.3935 1

2 -3 2 36.5322 2.6934 1

-2 3 2 33.7494 3.6178 2

3 2 -2 35.2412 2.8572 3

2 2 -3 34.6454 3.3152 2

2 3 -2 40.3875 2.9994 2

3 2 -2 36.4805 2.7452 2

2 2 -3 34.4094 2.5747 3

3 -2 2 33.4384 2.4978 3

-2 3 -2 38.3315 3.7948 2

3 -2 -2 32.8901 2.6255 1

-2 2 -3 37.6716 3.6852 2

-2 -3 2 38.0475 3.3274 1

3 2 2 32.1585 3.3616 3

2 -2 -3 39.0812 3.6510 2

-2 3 -2 37.3786 2.4414 3

2 2 3 39.0543 3.3655 3

2 -3 -2 39.7674 2.9034 1

2 3 2 31.1994 3.2655 3

-3 2 -2 37.8877 3.7574 2

-2 -2 3 34.3159 2.9012 1

2 -2 -3 37.7375 2.5877 1

0 -3 -3 2.74374 1.29557 1

0 3 3 4.80167 1.39231 3

3 0 -3 4.71729 0.88336 3

3 0 3 3.01299 1.30818 3

3 3 0 3.00159 1.61738 3

-3 3 0 6.93199 2.23049 2

0 3 -3 1.73101 0.95027 3

-3 -3 0 2.02125 1.45768 1

3 0 -3 5.74010 1.17426 1

3 0 3 5.40193 0.99342 1

0 -3 -3 6.17160 1.64188 2

0 3 -3 3.19618 1.62015 2

3 0 -3 4.92937 1.58941 2

0 -3 3 2.71142 1.75660 1

3 -3 0 4.17551 1.15364 1

-3 0 -3 6.44180 1.82098 2

3 -3 1 1101.39 49.41 1

1 3 -3 1130.09 48.74 3

3 -1 3 1065.84 48.84 3

-3 -1 -3 1084.89 49.40 1

-3 -1 -3 1092.49 52.91 2

3 -3 1 1109.83 48.18 3

3 1 -3 1112.85 52.83 2

-1 3 3 1114.44 48.63 3

-1 -3 -3 1079.16 52.69 2

-1 -3 -3 1120.26 50.08 1

1 -3 3 1072.93 49.40 1

1 3 -3 1135.22 53.00 2

3 1 -3 1144.28 48.53 3

-3 3 1 1159.13 53.79 2

3 -1 3 1092.40 49.00 1

-3 -3 -1 1094.09 50.19 1

3 1 -3 1112.65 49.48 1

3 3 -1 1108.62 49.18 3

3 -1 -3 1074.39 52.72 2

1 3 3 1104.59 48.91 3

1 -3 -3 1170.95 49.18 1

-3 1 -3 1019.02 52.80 2

3 3 1 1055.76 49.21 3

-1 -3 3 1181.10 49.88 1

3 -1 -3 1136.15 47.78 3

-1 3 -3 1127.90 48.22 3

3 -1 -3 1116.06 49.00 1

3 1 3 1058.96 48.90 3

-1 3 -3 1051.64 52.93 2

-3 3 -1 1085.24 53.08 2

1 -3 -3 1058.81 52.63 2

3 -3 -1 1113.94 49.17 1

-3 -3 1 1103.85 49.79 1

3 3 -2 390.190 17.635 3

-3 2 3 373.886 16.523 3

-3 3 2 372.773 19.943 2

3 -2 3 368.097 17.188 3

-3 -3 -2 357.370 18.204 2

3 -3 2 357.479 16.827 3

-3 -2 -3 365.329 17.781 1

-2 -3 -3 374.883 18.169 1

-2 3 3 368.370 16.828 3

2 -3 3 361.733 17.023 3

2 3 -3 388.072 17.392 3

-3 -2 -3 366.024 18.753 2

3 -3 2 362.332 17.242 1

-3 -3 -2 375.697 18.221 1

3 2 -3 383.836 17.272 3

3 2 -3 367.928 17.564 1

2 3 -3 404.684 18.935 2

2 -3 3 378.760 17.173 1

3 2 -3 381.075 18.773 2

3 3 -2 388.831 18.090 2

-2 -3 -3 381.849 18.714 2

3 -2 3 360.453 16.921 1

2 3 3 365.285 17.706 3

-3 2 -3 350.505 19.213 2

-3 3 -2 383.409 19.434 2

-2 -3 3 360.959 17.686 1

3 -2 -3 375.363 16.979 1

-2 3 -3 360.669 19.325 2

2 -3 -3 351.152 18.862 2

2 -3 -3 385.955 17.078 1

3 -2 -3 366.973 18.957 2

-3 -3 2 361.923 17.670 1

3 2 3 352.327 17.656 3

3 -3 -2 350.808 17.021 1

3 3 2 368.564 17.913 3

-3 -3 -3 1316.29 57.92 1

3 3 -3 1371.60 60.77 2

3 3 -3 1292.07 56.52 3

3 -3 3 1264.70 56.83 1

-3 3 3 1248.43 55.63 3

3 -3 3 1255.11 56.10 3

-3 -3 -3 1231.56 60.61 2

-3 -3 3 1265.25 56.63 1

3 -3 -3 1322.86 56.08 1

3 3 3 1212.45 56.19 3

-3 3 -3 1200.25 60.64 2

3 -3 -3 1263.45 60.39 2

0 0 4 9999.99 412.67 3

4 0 0 9193.71 412.54 3

0 4 0 9834.59 444.44 2

0 -4 0 9327.76 420.16 1

4 0 0 9969.88 419.40 1

0 0 -4 9178.20 444.78 2

0 4 0 9642.95 412.21 3

-4 -1 0 252.511 12.412 2

4 -1 0 226.781 11.561 1

4 1 0 249.224 11.809 1

-4 1 0 251.422 13.313 2

-1 0 -4 232.898 13.123 2

0 4 -1 245.748 12.772 2

0 4 1 241.475 12.811 2

1 0 -4 248.462 13.260 2

0 -4 1 225.586 12.583 1

1 0 4 247.000 11.606 3

4 -1 0 233.794 11.674 3

-1 0 4 257.055 11.337 3

0 4 -1 236.748 11.518 3

0 4 1 240.155 11.805 3

4 1 0 228.128 12.218 3

0 -4 -1 243.417 12.455 1

1 0 -4 257.317 11.170 1

-2 0 -4 2.20637 1.88376 2

0 4 2 3.11458 1.57946 3

-4 2 0 4.67610 2.23947 2

0 4 -2 -1.6622 1.1074 3

2 0 4 -0.4848 1.2679 3

-4 -2 0 3.75873 1.19961 2

2 0 -4 -0.9447 1.5600 2

4 -2 0 -0.1022 1.2169 3

4 -2 0 -0.7408 1.0689 1

2 0 -4 1.33517 1.04387 1

4 2 0 -1.7291 1.6263 3

0 -4 2 -4.2740 1.7155 1

0 -4 -2 -0.6385 1.5491 1

0 4 -2 0.64678 1.66647 2

4 2 0 -2.2319 1.2303 1

0 -4 -2 0.39062 1.34450 2

4 -3 0 110.666 6.055 1

-3 0 -4 109.340 7.347 2

3 0 4 113.918 6.425 3

3 0 -4 112.228 5.602 3

-4 -3 0 108.080 6.715 1

4 3 0 101.492 6.880 3

0 -4 -3 103.087 6.762 2

0 -4 -3 113.541 6.680 1

0 -4 3 97.8845 6.8319 1

-4 3 0 111.278 7.728 2

3 0 -4 117.710 7.297 2

0 4 -3 114.680 6.000 3

0 4 3 105.809 6.430 3

3 0 -4 115.407 5.965 1

0 4 -3 101.887 7.175 2

4 0 1 241.692 11.612 1

-4 0 1 252.098 13.276 2

1 -4 0 247.320 12.271 1

-1 4 0 219.129 11.181 3

0 1 4 251.953 11.527 3

-1 -4 0 219.554 12.676 1

-1 4 0 247.430 12.973 2

1 4 0 266.397 12.308 2

0 1 -4 237.236 13.212 2

4 0 -1 238.128 11.693 1

4 0 -1 239.453 11.531 3

0 -1 -4 245.830 13.181 2

4 0 1 216.966 12.032 3

-4 0 -1 256.982 12.920 2

0 -1 4 242.274 11.421 3

0 -1 -4 244.889 11.017 1

1 4 0 261.658 11.966 3

4 -1 1 248.039 12.533 1

1 -1 4 273.466 12.555 3

-4 -1 -1 258.943 13.564 2

1 1 -4 270.650 14.347 2

4 1 -1 270.515 12.884 1

1 -4 1 257.418 13.219 1

-1 4 1 276.188 14.378 2

-1 -1 -4 265.307 14.213 2

-1 1 4 282.696 12.379 3

1 4 -1 259.407 13.421 2

-4 1 1 278.740 14.667 2

4 1 -1 253.271 12.825 3

1 1 -4 272.352 12.319 1

1 1 -4 281.972 12.436 3

-1 4 1 272.616 12.477 3

4 -1 1 247.710 12.832 3

-1 -4 -1 267.791 13.838 1

1 4 -1 261.994 12.770 3

1 4 1 245.366 12.961 3

-1 4 -1 251.257 14.105 2

4 -1 -1 255.207 12.600 1

1 1 4 273.455 12.752 3

-4 1 -1 272.968 14.265 2

-1 -4 1 252.985 13.720 1

1 -4 -1 268.103 13.175 1

4 1 1 243.940 13.257 3

4 -1 -1 278.439 12.122 3

-1 1 -4 247.872 14.238 2

4 1 1 268.114 12.763 1

1 4 1 271.320 13.481 2

1 -1 -4 265.868 14.272 2

-4 -1 1 289.624 13.857 2

-1 4 -1 266.802 12.260 3

1 -1 -4 276.679 12.182 1

-1 4 2 273.641 13.419 3

2 1 -4 317.952 15.510 2

-4 2 1 302.330 15.887 2

-4 -2 -1 279.086 13.964 1

4 2 -1 282.744 13.874 1

2 1 -4 300.395 13.282 3

-1 4 2 285.747 15.328 2

1 4 -2 277.594 14.640 2

-2 -1 -4 277.209 13.383 1

-4 -2 -1 290.280 14.238 2

4 -2 1 283.862 13.464 1

1 4 -2 289.173 13.630 3

-1 -4 -2 265.075 14.322 2

4 2 -1 286.857 14.001 3

2 1 -4 282.853 13.381 1

-1 -4 -2 304.576 14.783 1

4 -2 1 272.848 13.329 3

2 -1 4 287.280 13.567 3

1 -4 2 285.113 14.119 1

-2 1 4 297.826 13.094 3

-2 -1 -4 278.361 15.128 2

4 -2 -1 289.989 13.745 1

-1 4 -2 278.520 13.337 3

2 -1 -4 281.368 15.436 2

4 2 1 266.035 14.492 3

1 4 2 273.824 14.147 3

2 1 4 295.302 13.997 3

-2 1 -4 273.636 15.538 2

-1 4 -2 280.790 15.512 2

1 -4 -2 296.817 14.229 1

-4 -2 1 337.721 14.463 2

-4 2 -1 283.045 15.668 2

-1 -4 2 284.907 14.897 1

2 -1 -4 299.915 13.456 1

-4 -3 -1 44.7689 4.0050 1

-1 -4 -3 41.6188 3.5606 2

1 -4 3 40.7240 3.6270 1

-3 -1 -4 42.9145 4.0309 2

3 -1 4 45.5660 2.6460 1

1 4 -3 46.1251 4.1147 2

3 1 -4 42.7306 3.0347 3

4 -3 1 46.2596 3.2959 1

3 1 -4 45.7051 4.2141 2

-3 -1 -4 41.0696 3.2554 1

-1 -4 -3 39.8642 3.9356 1

3 1 -4 42.6648 3.1595 1

4 -3 1 46.2793 2.9437 3

-1 4 3 45.0493 3.4479 3

1 4 -3 46.2847 3.3463 3

-4 3 1 53.4434 4.9780 2

3 -1 4 43.0810 3.5233 3

4 3 -1 45.4933 3.8917 3

-1 4 -3 47.8911 3.2158 3

-1 4 -3 45.6202 4.4728 2

3 -1 -4 39.6136 3.0370 1

-4 3 -1 45.7531 4.5966 2

1 4 3 42.9938 3.8366 3

-3 1 -4 45.2080 4.4132 2

1 -4 -3 47.1489 3.6387 1

3 1 4 43.8050 3.7762 3

-4 -3 1 45.4344 3.9551 1

-1 -4 3 41.7315 4.0928 1

4 3 1 46.9877 4.2171 3

4 -3 -1 45.3030 3.3181 1

1 -4 -3 40.6663 3.8976 2

3 -1 -4 44.1229 4.2121 2

4 0 -2 5.51574 1.47734 1

2 4 0 -1.8739 1.3819 3

4 0 -2 -1.1765 0.9495 3

-2 -4 0 -0.2576 1.8962 1

0 2 -4 -0.1779 1.7510 2

2 -4 0 -2.0523 1.3072 1

-2 4 0 -0.5357 0.7936 3

0 2 -4 5.54661 1.36598 3

-4 0 -2 0.00524 1.72010 2

0 -2 -4 2.79822 1.05652 1

4 0 2 0.04801 1.08179 1

4 0 2 2.60767 1.85741 3

-2 4 0 7.45966 2.29053 2

0 2 4 -0.8970 1.2251 3

0 -2 -4 1.27286 1.75437 2

-4 0 2 0.99127 1.99522 2

0 -2 4 -0.7051 1.3477 1

-2 -4 -1 297.783 15.053 1

4 -1 2 284.782 13.740 1

4 1 -2 320.109 13.920 3

2 4 -1 306.118 14.326 3

1 -2 4 287.376 13.738 3

-1 -2 -4 291.696 15.478 2

1 2 -4 304.513 15.756 2

-1 -2 -4 276.129 13.765 1

-4 -1 -2 290.452 15.164 2

4 1 -2 298.816 14.160 1

2 -4 1 285.880 14.227 1

1 2 -4 303.634 13.751 3

-4 1 2 297.695 16.190 2

4 -1 2 274.469 14.046 3

-2 4 1 279.233 15.851 2

-2 4 1 280.214 13.167 3

-1 2 4 336.829 13.856 3

-1 -2 4 303.508 13.941 1

4 -1 -2 296.618 12.912 3

1 2 4 295.260 13.901 3

2 4 1 264.531 14.221 3

-2 -4 1 288.610 14.776 1

2 -4 -1 285.123 13.977 1

4 -1 -2 288.869 13.715 1

1 -2 -4 282.934 15.344 2

-4 -1 2 291.261 15.147 2

-2 4 -1 275.933 15.488 2

-4 1 -2 301.996 15.584 2

1 -2 -4 300.632 13.399 1

-1 2 -4 263.726 15.485 2

-2 4 -1 290.985 13.049 3

4 1 2 252.800 14.282 3

4 1 2 308.349 13.820 1

2 -2 4 44.8930 2.5474 1

-2 -2 -4 43.6112 4.1323 2

4 2 -2 59.5213 3.8735 3

2 -2 4 42.4723 3.3397 3

4 2 -2 37.9681 3.4400 1

4 -2 2 43.3789 3.3499 3

-2 2 4 44.9688 3.0427 3

-4 -2 -2 42.5310 3.6776 2

-2 4 2 39.4553 4.4639 2

-2 -2 -4 42.2824 3.7324 1

-2 -4 -2 32.4032 3.1654 2

-2 -4 -2 39.8866 4.1586 1

2 -4 2 36.8876 3.4293 1

2 4 -2 45.1790 3.6865 3

4 -2 2 37.6400 3.0319 1

-2 4 2 39.0848 3.0189 3

-4 2 2 56.2818 5.4177 2

2 2 -4 42.1094 3.2628 3

-4 -2 -2 38.0219 3.6459 1

2 4 -2 54.0175 3.6760 2

2 2 -4 58.4123 4.5710 2

2 4 2 58.3273 4.4601 3

-4 2 -2 38.7303 4.4475 2

4 -2 -2 41.5152 3.2761 1

2 -2 -4 43.2142 3.1058 1

2 -2 -4 36.9290 3.9856 2

-2 4 -2 41.4172 4.4777 2

-2 -2 4 37.6428 3.3783 1

-2 -4 2 39.6760 4.1146 1

4 2 2 60.0163 4.8236 3

-2 2 -4 45.0917 4.5636 2

-2 4 -2 39.1676 2.9537 3

2 -4 -2 50.7085 3.7686 1

2 2 4 39.1976 3.6994 3

-4 -3 -2 190.205 10.285 1

-3 -2 -4 175.102 10.492 2

-2 4 3 177.845 9.122 3

4 3 -2 190.719 9.822 3

-4 3 2 193.394 11.870 2

3 -2 4 189.747 9.525 3

-4 -3 -2 178.526 9.739 2

-3 2 4 193.485 8.834 3

-3 -2 -4 176.190 9.731 1

4 -3 2 185.267 9.285 1

2 -4 3 189.334 9.483 1

3 2 -4 206.347 10.878 2

2 4 -3 189.289 9.647 3

-2 -4 -3 181.572 10.478 1

3 2 -4 178.823 9.489 1

4 -3 2 186.257 9.180 3

2 4 -3 227.143 10.607 2

3 2 -4 188.092 9.330 3

-2 -4 -3 178.391 10.056 2

3 -2 4 181.183 8.766 1

3 -2 -4 180.192 10.969 2

-3 2 -4 180.507 11.289 2

-4 3 -2 195.451 11.505 2

-2 4 -3 184.674 9.109 3

-4 -3 2 190.055 10.127 1

-2 -4 3 181.181 10.435 1

4 -3 -2 179.361 9.450 1

-2 4 -3 188.765 11.453 2

2 -4 -3 191.767 9.638 1

4 3 2 182.265 10.604 3

2 4 3 191.089 10.345 3

3 2 4 179.772 10.069 3

2 -4 -3 186.528 10.866 2

3 -2 -4 193.030 9.302 1

0 3 4 122.396 6.365 3

4 0 -3 103.070 6.915 2

-3 -4 0 109.729 7.053 1

0 3 -4 105.868 7.401 2

-4 0 -3 96.3817 7.1086 2

4 0 -3 109.278 6.073 1

0 3 -4 115.508 5.993 3

-3 4 0 106.472 7.550 2

0 -3 -4 122.158 6.171 1

3 -4 0 108.629 6.241 1

3 4 0 106.494 6.772 3

0 -3 -4 111.211 7.054 2

4 0 3 105.792 6.567 3

4 0 3 118.164 5.883 1

0 -3 4 86.6690 6.3160 1

4 0 -3 112.916 5.616 3

3 -4 1 46.0066 2.6819 3

1 3 -4 50.7222 4.4793 2

4 -1 3 46.2103 3.0020 1

-1 -3 -4 44.9728 4.0407 2

4 1 -3 48.7999 3.2258 3

-3 4 1 39.8375 4.4983 2

-4 -1 -3 44.0234 4.0098 2

1 -3 4 45.9226 3.1565 1

1 3 -4 46.3319 3.2641 3

3 -4 1 47.7016 3.5361 1

-1 -3 -4 37.7140 3.4316 1

-1 3 4 45.7493 3.3771 3

4 1 -3 44.1746 3.8545 2

3 4 -1 43.6845 3.8184 3

4 1 -3 45.2174 3.3992 1

-3 -4 -1 47.9083 4.2287 1

4 -1 3 42.4948 3.6297 3

1 -3 -4 47.9638 4.1868 2

4 1 3 44.6512 3.9869 3

3 -4 -1 41.4727 3.4276 1

1 3 4 40.7391 3.6693 3

4 -1 -3 44.9518 4.3104 2

-3 -4 1 46.3132 4.2275 1

-3 4 -1 45.5265 4.5273 2

-1 3 -4 42.4768 4.3981 2

4 -1 -3 48.4169 2.6652 3

4 1 3 45.6027 3.2517 1

4 -1 -3 37.9794 3.1763 1

3 4 1 43.3134 4.0826 3

-4 1 -3 47.4609 4.4326 2

1 -3 -4 50.0725 3.2768 1

-1 3 -4 41.2923 3.1688 3

-1 -3 4 52.5155 4.1797 1

4 2 -3 209.812 9.663 3

-3 -4 -2 183.097 9.617 2

-4 2 3 185.199 8.537 3

-4 -2 -3 185.021 9.905 1

3 4 -2 196.901 9.951 3

2 -3 4 190.584 9.014 1

4 2 -3 194.213 10.219 2

3 -4 2 191.968 9.575 1

-2 -3 -4 175.305 10.422 2

4 -2 3 168.773 9.056 1

4 2 -3 185.716 9.676 1

-2 -3 -4 175.311 10.056 1

4 -2 3 182.072 9.574 3

-3 -4 -2 190.607 10.553 1

2 -3 4 185.693 9.424 3

2 3 -4 199.297 9.532 3

3 -4 2 194.899 9.058 3

-3 4 2 179.496 11.663 2

2 3 -4 203.790 10.932 2

-2 3 4 194.000 9.192 3

-4 -2 -3 175.698 10.356 2

-4 2 3 197.819 11.840 2

-2 -3 4 184.690 9.973 1

2 -3 -4 193.363 9.265 1

2 3 4 173.380 9.928 3

-3 4 -2 192.701 11.352 2

-2 3 -4 174.171 11.154 2

2 -3 -4 170.527 10.687 2

-3 -4 2 190.330 10.434 1

4 -2 -3 194.021 9.315 1

4 2 3 179.517 10.354 3

-4 2 -3 180.144 11.218 2

3 4 2 177.768 10.241 3

3 -4 -2 181.293 9.472 1

3 3 -4 7.75755 1.69003 3

3 4 -3 7.82545 1.81746 3

-3 -3 -4 10.1121 2.2398 1

4 3 -3 11.8127 1.9706 3

3 -3 4 10.3924 1.3025 1

3 4 -3 6.40850 1.74900 2

-3 -3 -4 5.61725 1.86427 2

3 3 -4 8.86540 2.15900 2

4 -3 3 7.89928 1.40283 1

-3 4 3 7.98384 2.84812 2

-4 3 3 12.1240 3.2706 2

-3 -4 -3 10.0753 1.8797 2

4 -3 3 4.42929 1.48728 3

4 3 -3 8.11091 1.61095 2

3 -3 4 12.6963 1.8362 3

3 -4 3 8.67576 1.61662 1

-4 -3 -3 6.51769 2.07403 1

3 -4 3 12.5855 1.6176 3

-3 -4 -3 7.51828 2.25466 1

-4 -3 -3 6.36401 1.78877 2

-3 3 4 11.2711 1.4398 3

3 -3 -4 12.8143 2.5954 2

3 -4 -3 10.8089 1.8121 1

-4 -3 3 3.78503 1.85390 1

-3 3 -4 10.7277 2.9034 2

-3 -4 3 9.32504 2.52675 1

3 -3 -4 6.31624 1.47521 1

-3 -3 4 5.77504 1.89943 1

3 3 4 9.53352 2.47436 3

4 -3 -3 7.64866 1.62617 1

-4 3 -3 7.64276 2.82135 2

4 3 3 6.50717 2.45412 3

3 4 3 11.0121 2.5837 3

-3 4 -3 9.46552 2.74639 2

4 0 -4 7733.38 335.97 1

-4 0 -4 7372.63 356.71 2

0 -4 4 7443.64 336.46 1

4 0 4 8030.21 335.68 1

-4 -4 0 7842.29 337.08 1

0 -4 -4 7736.11 336.34 1

0 4 -4 8144.06 330.33 3

0 4 4 7778.60 330.72 3

4 0 -4 7814.94 329.78 3

4 0 4 7487.13 330.74 3

4 4 0 7501.66 331.21 3

4 0 -4 7829.60 356.72 2

0 -4 -4 7478.74 356.32 2

0 4 -4 7229.23 356.88 2

4 -4 0 7632.73 336.08 1

-4 4 0 7776.76 357.19 2

-1 -4 -4 340.412 17.240 2

4 -1 4 311.012 15.991 3

4 1 -4 337.591 15.370 3

4 -4 1 322.151 15.849 1

-1 4 4 335.941 15.781 3

1 4 -4 333.115 15.677 3

4 1 -4 335.182 15.853 1

-4 -1 -4 315.390 17.535 2

4 -4 1 350.277 15.013 3

4 4 -1 347.189 16.388 3

-4 4 1 328.245 18.390 2

4 -1 4 341.254 15.317 1

-4 -4 -1 362.262 17.077 1

-4 -1 -4 324.739 15.737 1

1 4 -4 342.469 17.750 2

-1 -4 -4 308.565 16.291 1

4 1 -4 316.940 17.273 2

1 -4 4 304.504 15.803 1

4 -1 -4 304.680 17.181 2

1 -4 -4 343.029 15.535 1

-1 -4 4 304.991 16.268 1

-4 4 -1 314.272 17.702 2

-4 1 -4 329.982 17.559 2

4 -1 -4 328.081 15.315 1

4 -1 -4 330.175 14.495 3

-1 4 -4 334.639 15.148 3

4 4 1 292.467 16.202 3

-1 4 -4 304.456 17.560 2

-4 -4 1 331.493 16.468 1

4 -4 -1 322.510 15.502 1

1 -4 -4 304.775 16.940 2

1 4 4 328.453 15.981 3

4 1 4 306.053 15.842 3

2 -4 4 15.0209 1.9392 1

4 2 -4 11.6483 1.9655 3

-4 4 2 13.9594 3.6079 2

4 -4 2 12.0452 1.7347 3

2 4 -4 11.4092 2.6648 2

-4 -4 -2 12.9569 1.7530 2

-4 -2 -4 18.3096 2.7518 2

4 -2 4 11.0120 1.5453 1

4 4 -2 21.5468 2.7000 3

4 -2 4 9.95360 2.09635 3

2 4 -4 16.8762 2.3028 3

-4 2 4 15.1218 1.4798 3

-2 4 4 13.7200 1.9864 3

2 -4 4 21.6471 2.2786 3

-4 -4 -2 9.35256 2.63203 1

4 2 -4 7.43532 2.16856 2

-2 -4 -4 19.4868 2.5821 2

-2 -4 -4 11.7065 2.7933 1

-4 -2 -4 28.2286 2.9543 1

4 2 -4 9.70189 1.96031 1

4 -4 2 15.5980 2.0930 1

2 4 4 12.0406 2.6502 3

-4 4 -2 16.6756 3.4707 2

4 -2 -4 21.9841 2.2852 1

-4 2 -4 19.4104 3.5767 2

4 2 4 14.3472 2.8689 3

4 -2 -4 11.0866 2.7691 2

4 -4 -2 12.8432 2.1392 1

-4 -4 2 11.6973 2.7316 1

2 -4 -4 19.3355 3.0778 2

-2 4 -4 16.7930 3.4401 2

-2 -4 4 18.1961 3.0956 1

2 -4 -4 13.5270 2.0945 1

4 4 2 17.4613 3.1921 3

3 4 -4 112.405 6.789 3

4 -4 3 111.031 6.406 1

4 4 -3 121.974 6.499 2

4 -3 4 108.761 6.016 1

4 4 -3 120.897 7.118 3

-4 4 3 120.917 9.225 2

-3 -4 -4 114.459 7.235 2

-4 -4 -3 106.164 6.729 2

-4 -3 -4 108.180 7.297 2

3 4 -4 127.428 7.603 2

-3 4 4 111.431 6.081 3

-4 -4 -3 131.885 7.900 1

3 -4 4 108.139 6.238 1

4 3 -4 108.277 7.108 2

3 -4 4 126.041 6.694 3

-3 -4 -4 114.160 7.596 1

-4 3 4 114.924 5.829 3

-4 -3 -4 116.069 7.287 1

4 -4 3 115.212 6.441 3

4 -3 4 121.435 6.785 3

4 3 -4 122.486 6.804 3

4 -4 -3 106.735 6.535 1

-4 -3 4 122.285 7.266 1

4 4 3 101.292 7.575 3

-3 4 -4 116.723 8.689 2

3 -4 -4 99.0535 7.7532 2

-4 3 -4 119.897 8.746 2

4 -3 -4 108.214 8.013 2

4 3 4 119.769 7.868 3

-4 -4 3 107.175 7.521 1

3 4 4 116.671 7.728 3

-4 4 -3 128.221 8.878 2

4 -3 -4 116.395 6.548 1

-3 -4 4 115.274 7.561 1

3 -4 -4 114.914 6.578 1

4 -4 4 4187.09 181.38 3

-4 -4 -4 4131.07 195.34 2

-4 4 4 4090.90 197.68 2

4 4 -4 4274.13 181.77 3

4 4 -4 4289.87 195.30 2

-4 -4 -4 4120.01 185.22 1

4 -4 4 4264.76 184.03 1

-4 4 -4 4101.01 194.31 2

-4 -4 4 4158.37 182.40 1

4 -4 -4 4115.71 181.53 1

0 5 0 0.28193 1.33225 2

0 5 0 -0.5547 1.0793 3

-5 0 0 1.43846 1.71190 2

0 0 -5 -0.4972 1.5693 2

5 0 0 0.72388 1.10045 1

0 0 5 1.80297 1.04120 3

0 -5 0 0.16763 1.86488 1

5 0 0 0.11345 1.39485 3

1 0 5 805.124 36.642 3

-1 0 5 851.713 36.419 3

-5 1 0 839.778 40.325 2

0 -5 1 768.717 38.191 1

-5 -1 0 842.058 39.428 2

5 1 0 777.794 37.331 3

0 5 -1 796.563 39.730 2

0 5 1 850.577 39.841 2

1 0 -5 823.823 40.423 2

-1 0 -5 810.538 40.315 2

5 -1 0 809.953 37.083 1

5 1 0 826.264 37.281 1

0 5 -1 831.013 36.614 3

0 5 1 836.235 36.817 3

1 0 -5 861.145 36.484 1

0 -5 -1 817.681 38.174 1

0 5 2 218.696 12.556 2

0 -5 -2 217.664 12.093 1

5 2 0 234.844 12.254 3

0 5 2 228.589 11.580 3

2 0 5 217.629 11.362 3

-2 0 5 236.088 10.869 3

-2 0 -5 239.370 13.091 2

0 -5 2 216.917 12.459 1

2 0 -5 224.252 10.775 1

0 5 -2 227.607 12.631 2

-5 2 0 223.661 13.163 2

-5 -2 0 226.597 11.577 2

5 2 0 238.159 11.652 1

0 5 -2 219.386 11.098 3

2 0 -5 242.594 13.128 2

5 -2 0 227.331 11.212 1

0 -5 -3 266.578 14.854 2

3 0 -5 300.421 13.295 3

0 -5 -3 289.528 14.733 1

5 3 0 284.429 14.839 3

0 5 -3 266.590 15.508 2

0 -5 3 267.822 14.950 1

3 0 5 276.133 14.111 3

-5 -3 0 292.631 14.655 1

-5 3 0 273.717 16.117 2

-3 0 -5 289.240 15.844 2

3 0 -5 296.155 13.576 1

0 5 3 277.561 14.078 3

5 -3 0 290.462 13.831 1

3 0 -5 285.002 15.732 2

0 5 -3 293.479 13.729 3

0 5 -4 227.711 13.909 2

4 0 -5 241.085 13.691 2

4 0 5 249.961 12.592 3

0 5 -4 245.475 11.937 3

0 5 4 230.843 12.387 3

4 0 -5 242.604 11.370 3

-4 0 -5 237.933 13.922 2

5 4 0 223.731 12.883 3

0 -5 -4 230.184 13.118 2

0 -5 4 231.822 12.928 1

0 -5 -4 245.708 12.669 1

-5 4 0 238.620 14.342 2

-4 0 -5 244.818 11.488 1

5 -4 0 236.650 12.081 1

4 0 -5 252.617 11.918 1

-5 -4 0 237.307 13.095 1

-5 0 -1 856.863 40.604 2

1 -5 0 806.740 38.513 1

0 -1 -5 801.472 40.926 2

-5 0 1 892.861 40.873 2

0 1 5 845.485 37.231 3

0 -1 5 841.863 37.161 3

-1 5 0 894.223 40.787 2

1 5 0 925.355 40.107 2

5 0 1 850.174 37.804 1

0 -1 -5 863.693 37.002 1

0 1 -5 807.712 41.075 2

5 0 -1 799.816 37.813 1

1 5 0 846.243 37.532 3

-1 5 0 812.617 36.929 3

5 0 -1 834.297 37.287 3

-1 -5 0 765.731 38.926 1

5 0 1 785.721 37.931 3

1 5 -1 2237.86 96.84 3

-1 5 1 2190.45 104.77 2

-1 -5 -1 2216.49 99.50 1

-1 1 5 2334.75 96.51 3

-5 1 1 2311.44 105.21 2

1 1 -5 2235.82 105.14 2

-5 -1 -1 2253.63 104.23 2

5 -1 1 2054.84 97.04 3

5 1 -1 2218.24 96.97 3

5 -1 1 2210.03 98.13 1

1 5 -1 2099.70 104.08 2

1 -5 1 2181.61 98.96 1

-1 -1 -5 2214.49 104.88 2

5 1 -1 2224.57 98.43 1

1 -1 5 2201.95 96.62 3

-1 5 1 2342.99 96.50 3

1 1 -5 2250.28 96.45 3

-1 -5 1 2172.88 100.92 1

1 5 1 2201.70 98.54 3

5 -1 -1 2311.40 97.68 3

1 -1 -5 2295.81 99.10 1

5 -1 -1 2199.04 99.67 1

-1 5 -1 2269.21 106.39 2

1 -5 -1 2160.29 100.38 1

1 1 5 2341.54 98.32 3

1 -1 -5 2277.07 106.67 2

-5 -1 1 2461.05 105.94 2

-1 5 -1 2147.56 97.72 3

-5 1 -1 2304.37 106.43 2

1 5 1 2280.01 105.69 2

-1 1 -5 2213.67 106.70 2

5 1 1 2308.09 99.82 1

-1 -1 5 2284.91 99.26 1

5 1 1 2103.78 98.94 3

-1 5 2 5.34957 1.62996 3

1 -5 2 3.14969 1.87060 1

1 5 -2 1.49956 1.34130 3

2 1 -5 2.90591 2.01631 2

1 5 -2 3.21011 1.62067 2

5 -2 1 0.80653 1.46142 3

-5 2 1 2.96513 2.37390 2

-2 1 5 3.76035 1.09358 3

2 1 -5 3.27381 1.07689 1

-2 -1 -5 1.29549 1.70459 2

5 -2 1 2.09961 1.18296 1

5 2 -1 1.97448 1.33737 1

2 -1 5 3.22034 1.36023 3

-5 -2 -1 1.75095 1.35618 2

5 2 -1 -0.2794 1.3715 3

-1 5 2 6.20918 2.18644 2

-1 -5 -2 2.33321 1.28880 2

2 1 -5 2.60689 1.04193 3

-1 -5 -2 3.42416 2.22011 1

2 1 5 2.01956 1.40262 3

-1 -5 2 1.76920 2.22663 1

2 -1 -5 2.97011 1.84276 2

5 -2 -1 2.32057 1.22724 1

-1 5 -2 1.24233 1.94382 2

1 5 2 2.82582 1.67150 3

5 2 1 2.16933 1.85768 3

-5 2 -1 0.17041 2.05061 2

-2 1 -5 1.30279 1.91150 2

-5 -2 1 4.96005 1.50412 2

-1 5 -2 4.48492 1.26486 3

2 -1 -5 3.26857 1.00966 1

1 -5 -2 5.86838 1.83655 1

5 2 1 1.86206 1.48510 1

1 5 -3 1259.24 54.77 3

3 -1 5 1199.23 54.86 3

3 1 -5 1289.22 59.89 2

5 -3 1 1168.76 54.31 3

5 3 -1 1234.20 55.29 3

-3 -1 -5 1313.88 55.19 1

-1 5 3 1229.98 54.67 3

-5 3 1 1225.15 60.28 2

-3 -1 -5 1179.08 59.57 2

3 1 -5 1308.33 54.32 3

-3 1 5 1277.28 54.11 3

-5 -3 -1 1260.05 58.17 2

-5 -3 -1 1288.81 56.29 1

1 5 -3 1198.88 59.25 2

-1 -5 -3 1162.62 58.70 2

5 -3 1 1192.32 55.30 1

3 1 -5 1245.28 55.22 1

1 -5 3 1210.93 55.97 1

-1 -5 -3 1273.02 56.52 1

3 -1 -5 1254.83 53.96 1

-5 -3 1 1213.21 54.92 1

3 1 5 1218.67 53.93 3

5 3 1 1166.04 54.64 3

5 -3 -1 1204.11 54.20 1

-1 5 -3 1263.92 53.20 3

1 5 3 1246.24 54.10 3

-5 3 -1 1220.24 58.84 2

-1 5 -3 1201.93 58.77 2

1 -5 -3 1286.24 54.88 1

3 -1 -5 1237.17 58.53 2

1 -5 -3 1107.91 57.80 2

-3 1 -5 1156.85 58.66 2

-1 -5 3 1134.29 55.31 1

5 -4 1 468.095 20.181 3

1 5 -4 444.720 20.789 3

-1 5 4 426.159 20.852 3

4 1 -5 448.686 20.860 1

4 -1 5 480.398 20.201 1

4 1 -5 450.568 23.062 2

4 1 -5 459.941 20.344 3

1 5 -4 454.234 23.217 2

-5 4 1 443.917 23.845 2

1 -5 4 435.310 21.330 1

-4 -1 -5 440.348 20.803 1

-1 -5 -4 445.160 22.437 2

-4 -1 -5 422.655 23.071 2

5 4 -1 440.366 21.423 3

-1 -5 -4 413.847 21.588 1

5 -4 1 440.644 20.984 1

-5 -4 -1 425.443 21.996 1

4 -1 5 442.524 21.208 3

-4 1 -5 421.107 22.735 2

-5 -4 1 438.919 21.345 1

-1 5 -4 422.012 19.826 3

1 -5 -4 447.758 20.691 1

4 -1 -5 436.658 22.668 2

1 -5 -4 403.639 21.977 2

4 -1 -5 444.398 20.101 1

1 5 4 451.509 20.898 3

4 1 5 427.834 20.708 3

-5 4 -1 423.717 23.024 2

5 -4 -1 428.635 20.337 1

5 4 1 414.088 21.276 3

-1 -5 4 396.383 21.309 1

-1 5 -4 418.031 22.876 2

2 5 0 229.585 11.780 3

0 -2 -5 233.317 10.809 1

-2 5 0 227.251 12.980 2

5 0 2 216.209 12.114 3

-2 5 0 212.524 10.608 3

-5 0 2 233.066 13.264 2

-2 -5 0 234.359 12.700 1

5 0 2 235.242 11.322 1

0 2 5 251.891 11.529 3

2 -5 0 222.518 11.860 1

0 2 -5 243.632 11.238 3

-5 0 -2 212.403 12.596 2

5 0 -2 231.710 10.976 3

0 2 -5 239.713 13.306 2

0 -2 -5 225.012 12.928 2

5 0 -2 232.819 11.483 1

1 2 -5 3.19833 1.97571 2

5 -1 2 1.79895 1.18493 1

-1 2 5 2.04184 1.20972 3

-1 -2 -5 5.48268 2.03610 2

-5 -1 -2 4.78391 1.84526 2

-2 -5 -1 5.10410 2.33286 1

1 -2 5 0.38110 1.05125 3

-2 5 1 3.16543 0.98018 3

2 -5 1 2.62388 1.64913 1

-5 1 2 1.12640 2.31814 2

1 2 -5 3.02339 1.16347 3

5 1 -2 1.87350 1.28196 1

5 -1 2 1.68576 1.58798 3

-1 -2 -5 2.50725 1.00109 1

-2 5 1 1.07094 1.95510 2

5 1 -2 8.35358 1.61897 3

2 5 -1 2.50165 1.50500 3

5 -1 -2 3.53067 0.87988 3

-2 5 -1 2.99933 1.04577 3

5 1 2 4.53328 2.08773 3

2 -5 -1 0.99114 1.36768 1

-1 2 -5 3.39023 2.04691 2

-1 -2 5 1.90751 1.43097 1

5 1 2 4.53156 1.44019 1

-5 -1 2 4.68531 2.13349 2

-5 1 -2 2.00860 1.97255 2

5 -1 -2 -0.8996 1.0997 1

2 5 1 1.61167 1.67429 3

-2 5 -1 4.83577 2.24214 2

1 -2 -5 7.39346 2.19793 2

1 -2 -5 3.62086 0.97533 1

-2 -5 1 2.73230 2.15771 1

2 5 1 1.63873 1.20517 2

1 2 5 1.62351 1.43445 3

-2 -5 -2 39.2153 4.3065 1

-2 5 2 43.2061 3.1448 3

-2 2 5 51.0844 3.3033 3

2 5 -2 51.9042 3.7233 2

-2 -2 -5 38.0564 3.2146 1

5 -2 2 46.4163 3.6960 3

5 -2 2 40.2628 3.2109 1

5 2 -2 38.1730 3.5235 1

-2 -5 -2 33.3050 3.0660 2

-5 2 2 42.8394 5.0293 2

-2 -2 -5 40.2267 4.0619 2

2 2 -5 42.3490 3.1773 3

-2 5 2 41.4576 4.6509 2

2 -5 2 39.8315 3.6766 1

-5 -2 -2 48.0320 3.9332 1

-5 -2 -2 41.1479 3.6985 2

2 5 -2 44.6642 3.7258 3

2 2 -5 43.6256 4.4106 2

2 -2 5 45.0929 3.4152 3

5 2 -2 41.3864 3.6101 3

5 -2 -2 46.0610 3.4961 1

2 5 2 40.3369 3.9922 3

-2 -5 2 33.3783 4.1985 1

5 2 2 40.7202 4.3672 3

-2 5 -2 37.4702 4.4400 2

-2 5 -2 39.1980 3.0126 3

2 -5 -2 43.1637 3.8116 1

-5 2 -2 47.8602 4.7157 2

2 -2 -5 42.7472 3.0398 1

-2 -2 5 41.5757 3.5155 1

-2 2 -5 44.4812 4.7152 2

2 -2 -5 47.0826 4.4528 2

2 2 5 44.2877 3.9257 3

-5 -2 2 54.3099 4.3818 2

-5 3 2 69.7063 6.5682 2

5 -3 2 69.9181 4.4899 3

-2 5 3 62.7573 4.3133 3

2 5 -3 71.3081 4.8470 3

3 2 -5 59.0836 4.4285 1

5 3 -2 71.3918 5.0197 3

-2 -5 -3 65.5950 4.6426 2

-5 -3 -2 64.4618 4.5589 2

-5 -3 -2 58.3769 5.1038 1

5 -3 2 62.6444 4.2352 1

-3 -2 -5 64.3614 4.6627 1

-3 -2 -5 61.9129 5.3444 2

2 5 -3 67.2675 5.0403 2

2 -5 3 64.5395 4.6960 1

-2 5 3 70.3090 6.2378 2

3 2 -5 70.3747 5.7308 2

3 2 -5 66.0979 4.3020 3

-3 2 5 71.0719 4.0262 3

3 -2 5 71.6749 4.7237 3

-2 -5 -3 63.7250 5.6394 1

-3 2 -5 60.4432 5.8574 2

2 -5 -3 63.1243 4.8101 1

2 5 3 66.7892 5.4893 3

3 -2 -5 63.3868 5.5803 2

-2 5 -3 68.7294 6.2132 2

5 -3 -2 62.0467 4.4188 1

-5 -3 2 66.6217 5.1594 1

3 -2 -5 64.6199 4.1606 1

5 3 2 68.3364 5.8080 3

3 2 5 59.3248 4.9795 3

-2 -5 3 62.0565 5.4804 1

-5 3 -2 64.9903 6.0221 2

-5 -3 2 84.6993 4.7593 2

-2 5 -3 56.4203 4.0652 3

4 2 -5 203.500 10.122 3

4 -2 5 199.625 9.508 1

5 4 -2 187.563 10.777 3

-4 -2 -5 201.144 11.819 2

4 2 -5 190.453 11.530 2

-2 5 4 193.243 10.173 3

-2 -5 -4 188.132 11.420 1

-2 -5 -4 199.106 11.092 2

5 -4 2 208.192 10.356 1

-5 -4 -2 197.017 11.499 1

-4 2 5 201.151 9.576 3

2 -5 4 199.930 10.445 1

-5 4 2 201.435 12.976 2

2 5 -4 202.771 10.582 3

-4 -2 -5 217.348 10.752 1

4 2 -5 203.384 10.409 1

5 -4 2 209.534 10.052 3

4 -2 5 177.051 10.485 3

2 5 -4 215.141 11.711 2

-5 -4 2 191.738 11.093 1

2 -5 -4 177.255 11.395 2

-2 5 -4 192.089 9.771 3

4 -2 -5 200.360 9.945 1

2 5 4 195.414 11.115 3

2 -5 -4 213.183 10.455 1

4 2 5 180.581 10.835 3

5 4 2 182.725 11.363 3

5 -4 -2 189.643 10.178 1

4 -2 -5 193.799 11.867 2

-4 2 -5 204.314 12.498 2

-5 4 -2 187.974 12.383 2

-2 -5 4 185.312 11.203 1

-2 5 -4 196.129 12.489 2

5 0 -3 293.039 14.443 1

0 -3 5 277.345 14.384 1

-5 0 3 295.522 16.671 2

5 0 -3 309.805 13.788 3

0 -3 -5 298.352 14.113 1

3 -5 0 280.814 14.669 1

0 3 5 315.059 14.536 3

-3 -5 0 283.496 15.493 1

-3 5 0 321.161 16.679 2

-3 5 0 287.184 13.239 3

0 -3 -5 322.876 16.201 2

-5 0 -3 301.529 16.173 2

3 5 0 296.995 14.955 3

0 3 -5 302.635 16.566 2

5 0 3 264.106 14.860 3

0 3 -5 306.389 14.223 3

5 0 3 300.550 14.230 1

-1 -3 -5 1243.06 60.52 2

1 -3 5 1228.62 55.58 3

3 -5 1 1237.16 56.72 1

-5 -1 -3 1240.65 60.37 2

-5 1 3 1327.94 61.63 2

-3 -5 -1 1249.65 57.60 1

1 3 -5 1279.27 61.01 2

3 5 -1 1257.67 56.17 3

-1 -3 -5 1274.91 56.38 1

5 -1 3 1232.21 56.13 1

5 -1 3 1245.79 56.11 3

-3 5 1 1231.09 61.07 2

1 3 -5 1243.64 55.50 3

-1 3 5 1299.98 55.62 3

1 -3 5 1275.22 55.83 1

5 1 -3 1250.09 56.62 1

5 1 -3 1327.68 55.46 3

-3 5 -1 1206.17 53.90 3

5 -1 -3 1263.82 55.61 1

-5 -1 3 1322.46 59.87 2

1 -3 -5 1268.43 55.26 1

-3 5 -1 1223.21 60.14 2

-5 1 -3 1226.38 60.00 2

5 -1 -3 1221.55 53.85 3

-1 3 -5 1185.31 60.24 2

5 1 3 1281.98 55.52 1

1 3 5 1272.75 55.17 3

5 1 3 1175.72 55.80 3

-3 -5 1 1255.18 56.80 1

3 -5 -1 1195.89 55.83 1

3 5 1 1234.90 55.68 3

1 -3 -5 1249.29 59.76 2

-1 -3 5 1254.95 55.97 1

-2 -3 -5 58.3690 4.6693 1

2 3 -5 68.4656 4.4657 3

-3 -5 -2 65.0083 4.0579 2

5 2 -3 66.9706 4.7292 1

3 5 -2 71.0422 4.9764 3

-3 -5 -2 64.3092 5.5474 1

5 -2 3 61.0339 4.1155 1

2 -3 5 63.5635 3.6123 1

3 -5 2 71.7866 4.7735 1

-5 -2 -3 64.8634 4.8525 1

5 2 -3 66.5579 4.5359 3

-5 2 3 68.6351 6.5496 2

-2 -3 -5 58.8406 5.1416 2

-5 -2 -3 62.5348 5.0315 2

2 3 -5 73.3727 5.8695 2

5 -2 3 62.5348 4.7367 3

5 2 -3 64.4844 4.6921 2

2 -3 5 69.8664 4.5384 3

-3 5 2 73.9535 6.5213 2

-2 3 5 67.3991 4.3502 3

3 5 2 68.7536 5.4325 3

5 2 3 56.9047 5.3451 3

5 -2 -3 63.5261 4.3922 1

2 -3 -5 67.6068 4.1891 1

-3 -5 2 70.2475 5.7629 1

5 -2 -3 67.5075 3.3880 3

3 -5 -2 68.9603 4.7947 1

-2 -3 5 61.9468 5.0355 1

-5 2 -3 59.1090 5.8166 2

-2 3 -5 70.1758 6.0864 2

2 3 5 63.8217 5.0099 3

2 -3 -5 60.3501 5.4229 2

-3 5 -2 61.7284 3.8395 3

-3 5 -2 62.7289 5.9071 2

-3 -5 -3 1428.69 67.10 2

-5 -3 -3 1404.44 67.45 2

5 -3 3 1378.66 63.33 1

-3 -3 -5 1389.16 67.97 2

5 -3 3 1414.20 62.78 3

-3 -5 -3 1501.87 64.98 1

3 3 -5 1507.46 68.48 2

3 5 -3 1573.27 67.28 2

3 -5 3 1457.27 62.46 3

5 3 -3 1488.33 63.17 3

-3 5 3 1330.87 69.18 2

3 -3 5 1420.64 62.88 3

3 3 -5 1436.46 62.72 3

-5 -3 -3 1333.66 64.34 1

3 5 -3 1301.56 62.95 3

3 -5 3 1411.64 63.68 1

-5 3 3 1434.68 69.73 2

-3 -3 -5 1404.81 64.12 1

-3 3 5 1505.92 62.34 3

3 -3 5 1376.90 62.82 1

5 3 3 1323.82 62.17 3

3 -3 -5 1324.21 66.43 2

3 -5 -3 1447.61 62.09 1

3 5 3 1417.02 62.02 3

-5 3 -3 1367.13 67.05 2

-3 5 -3 1363.94 67.12 2

-3 3 -5 1364.36 67.14 2

-3 -3 5 1436.02 62.26 1

5 -3 -3 1350.32 61.70 1

-3 -5 3 1318.13 62.76 1

3 -3 -5 1440.18 61.56 1

3 3 5 1382.70 61.70 3

-5 -3 3 1365.07 62.33 1

-3 5 4 224.022 13.831 2

-3 5 4 204.951 10.377 3

4 3 -5 232.651 12.346 2

5 -4 3 208.725 10.775 1

-4 -3 -5 206.099 12.144 2

-3 -5 -4 218.151 12.272 1

4 -3 5 207.918 10.124 1

-5 4 3 229.681 14.257 2

3 5 -4 240.433 12.020 2

-4 3 5 221.504 10.246 3

5 4 -3 216.495 11.452 3

-4 -3 -5 212.720 11.491 1

3 5 -4 207.358 11.195 3

3 -5 4 223.995 10.977 1

-3 -5 -4 198.721 11.460 2

4 3 -5 204.274 10.853 3

-5 -4 -3 202.298 11.258 2

-5 -4 -3 200.775 12.037 1

4 -3 5 201.488 11.083 3

3 -5 4 209.576 10.830 3

5 -4 3 200.140 10.785 3

4 -3 -5 209.843 12.759 2

-5 -4 3 214.504 11.983 1

5 -4 -3 207.234 10.951 1

4 -3 -5 226.114 10.842 1

4 3 5 197.635 11.833 3

-4 3 -5 201.786 13.191 2

5 4 3 177.163 11.999 3

-3 -5 4 203.917 12.112 1

3 -5 -4 210.513 11.114 1

-5 4 -3 208.526 13.507 2

-4 -3 5 209.010 11.368 1

3 5 4 206.770 12.012 3

-3 5 -4 216.550 13.588 2

-5 0 -4 237.725 13.949 2

0 -4 -5 249.793 12.304 1

0 -4 5 232.661 12.566 1

4 -5 0 248.541 12.551 1

5 0 -4 241.002 12.185 1

-4 5 0 240.988 14.394 2

5 0 4 240.868 12.832 3

0 4 5 238.257 12.416 3

0 4 -5 243.689 12.036 3

4 5 0 251.968 12.997 3

-4 -5 0 251.968 13.500 1

0 4 -5 232.207 14.197 2

5 0 -4 251.909 11.497 3

0 -4 -5 232.645 13.537 2

5 0 -4 228.328 13.544 2

5 0 4 260.599 11.925 1

-5 -1 -4 434.091 22.935 2

-1 -4 -5 437.340 22.629 2

-1 4 5 444.820 20.732 3

-1 -4 -5 418.582 21.092 1

1 -4 5 429.847 20.588 1

1 4 -5 466.367 23.476 2

4 -5 1 459.453 21.168 1

5 1 -4 437.559 22.652 2

5 -1 4 452.223 20.428 1

5 1 -4 429.443 20.934 1

4 -5 1 455.392 19.784 3

4 5 -1 449.056 21.302 3

-5 -1 -4 449.982 20.906 1

5 1 -4 444.588 20.307 3

-4 5 1 436.337 23.592 2

5 -1 4 429.929 21.122 3

-4 -5 -1 462.778 22.191 1

1 4 -5 438.613 20.636 3

5 -1 -4 434.294 19.515 3

-5 1 -4 442.696 23.221 2

5 -1 -4 429.600 22.912 2

5 -1 -4 452.371 20.648 1

5 1 4 436.144 21.433 3

4 5 1 432.512 21.540 3

-1 4 -5 418.468 23.344 2

1 4 5 449.208 21.063 3

1 -4 -5 426.894 22.593 2

1 -4 -5 437.506 20.532 1

4 -5 -1 436.732 20.918 1

-1 -4 5 393.213 21.251 1

-4 -5 1 422.626 21.825 1

-4 5 -1 455.774 23.536 2

-1 4 -5 471.849 20.413 3

-5 -2 -4 186.376 10.634 1

-2 4 5 191.371 10.052 3

-2 -4 -5 194.308 10.762 1

4 -5 2 200.947 10.472 1

2 4 -5 222.412 12.182 2

-5 -2 -4 188.101 11.472 2

-5 2 4 191.554 12.986 2

2 4 -5 197.051 10.321 3

-2 -4 -5 202.926 11.403 2

-4 5 2 199.034 12.782 2

5 -2 4 195.988 10.604 3

5 2 -4 211.007 10.269 3

2 -4 5 209.073 9.832 1

-4 -5 -2 215.088 11.680 1

5 2 -4 186.898 10.383 1

4 -5 2 199.486 9.673 3

5 -2 4 193.123 9.757 1

-5 2 4 197.085 9.250 3

5 2 -4 207.747 11.259 2

2 -4 5 202.211 10.355 3

4 5 -2 189.664 10.652 3

4 -5 -2 182.195 10.283 1

4 5 2 194.651 11.335 3

-5 2 -4 209.129 12.561 2

-2 -4 5 198.464 11.085 1

-4 5 -2 179.455 12.234 2

5 2 4 180.299 11.166 3

2 -4 -5 201.511 10.033 1

5 -2 -4 196.827 10.048 1

2 -4 -5 183.694 11.538 2

2 4 5 189.518 10.881 3

-2 4 -5 191.925 12.377 2

-4 -5 2 193.934 11.382 1

3 -4 5 216.504 10.214 1

5 -3 4 209.521 11.057 3

-3 -4 -5 221.039 11.709 1

3 -4 5 212.452 10.874 3

4 -5 3 213.267 10.475 3

-5 -3 -4 203.276 11.588 1

-3 4 5 207.775 10.325 3

-4 -5 -3 210.107 12.004 1

-5 -3 -4 192.714 11.651 2

5 3 -4 235.220 11.174 3

4 -5 3 212.367 10.882 1

3 4 -5 209.353 10.871 3

5 3 -4 195.692 11.346 2

-4 5 3 207.309 13.697 2

-3 -4 -5 204.237 11.808 2

-4 -5 -3 209.204 10.935 2

-5 3 4 197.219 13.801 2

3 4 -5 230.112 12.474 2

4 5 -3 200.166 11.200 3

5 -3 4 195.465 10.251 1

-5 -3 4 194.113 11.294 1

3 -4 -5 205.673 10.643 1

3 4 5 202.526 11.852 3

4 -5 -3 201.016 10.891 1

-4 5 -3 213.980 13.476 2

5 -3 -4 197.323 10.660 1

-3 -4 5 211.503 11.721 1

3 -4 -5 203.372 12.441 2

-3 4 -5 213.577 13.370 2

5 3 4 202.891 12.061 3

4 5 3 222.633 12.205 3

-4 -5 3 208.622 12.068 1

-5 3 -4 200.541 13.158 2

-5 -4 -4 284.039 15.673 1

4 5 -4 289.229 15.072 3

-5 4 4 311.444 18.544 2

4 -5 4 311.274 14.612 1

5 4 -4 292.638 15.029 3

4 -4 5 298.071 14.110 1

-5 -4 -4 294.644 15.662 2

5 -4 4 292.983 14.345 1

-4 -4 -5 326.258 15.806 1

-4 4 5 284.420 13.849 3

4 5 -4 312.886 15.347 2

4 -4 5 295.192 14.866 3

-4 -5 -4 296.444 15.434 2

4 4 -5 308.439 16.083 2

4 -5 4 286.405 14.520 3

-4 -5 -4 305.335 15.991 1

4 4 -5 302.873 14.895 3

5 4 -4 299.993 15.230 2

-4 5 4 275.704 17.858 2

5 -4 4 280.373 14.703 3

-4 -4 -5 298.030 16.007 2

-4 -5 4 294.389 15.882 1

4 -5 -4 292.942 14.775 1

-4 5 -4 301.276 17.798 2

-5 4 -4 290.351 17.592 2

4 -4 -5 282.541 16.739 2

4 4 5 269.942 15.657 3

-5 -4 4 283.743 15.528 1

5 -4 -4 302.011 14.721 1

5 4 4 290.370 16.052 3

4 -4 -5 297.580 14.586 1

-4 -4 5 290.130 15.508 1

4 5 4 271.139 15.785 3

-4 4 -5 311.833 17.868 2

-5 5 0 740.531 38.696 2

-5 0 -5 751.675 38.274 2

0 5 -5 789.661 34.486 3

0 5 5 765.140 35.016 3

5 0 -5 773.912 33.795 3

5 0 5 742.058 35.129 3

5 5 0 734.605 35.363 3

0 -5 -5 746.133 37.479 2

0 5 -5 761.551 38.610 2

5 0 -5 782.161 38.052 2

5 0 -5 779.251 34.905 1

5 0 5 777.012 34.398 1

0 -5 5 702.705 35.365 1

5 -5 0 771.388 35.144 1

-5 -5 0 790.909 36.403 1

0 -5 -5 770.270 35.329 1

-5 -1 -5 849.479 38.761 1

-1 -5 -5 833.999 39.401 1

-5 -5 -1 839.877 40.132 1

5 1 -5 817.177 38.762 1

1 5 -5 870.279 42.474 2

5 5 -1 837.890 38.988 3

5 1 -5 859.835 41.947 2

-5 -1 -5 852.199 42.197 2

5 -5 1 814.676 38.889 1

1 5 -5 879.835 38.393 3

-5 5 1 844.400 42.945 2

-1 -5 -5 867.662 41.533 2

5 -1 5 815.711 38.825 3

5 1 -5 872.486 37.872 3

1 -5 5 835.664 38.888 1

-1 5 5 859.226 38.582 3

5 -1 5 903.129 38.195 1

-1 5 -5 852.861 37.264 3

-5 5 -1 777.684 41.600 2

-1 5 -5 764.469 41.610 2

1 -5 -5 811.408 40.764 2

-5 -5 1 833.131 39.197 1

5 -1 -5 855.472 41.346 2

5 5 1 771.976 38.488 3

5 -5 -1 834.712 38.030 1

-5 1 -5 822.155 41.527 2

5 -1 -5 832.329 37.771 1

5 1 5 852.001 38.350 3

5 -1 -5 845.248 36.436 3

1 -5 -5 900.494 38.238 1

1 5 5 859.171 38.198 3

-1 -5 5 767.164 38.768 1

-2 -5 -5 73.6593 5.9904 1

5 5 -2 78.3168 5.7299 3

5 2 -5 76.3342 4.9203 3

5 -5 2 76.3962 4.7147 3

2 5 -5 82.6914 6.5447 2

5 -5 2 63.1818 5.0263 1

5 2 -5 80.1782 6.0486 2

5 -2 5 68.1923 4.3886 1

2 5 -5 82.7086 5.3016 3

-5 2 5 73.9386 4.2629 3

-5 -5 -2 77.1358 6.5122 1

2 -5 5 76.4932 5.2522 3

-5 -2 -5 71.4584 5.5209 1

-2 -5 -5 70.6799 5.6624 2

-5 5 2 74.5423 7.2130 2

-5 -2 -5 70.6763 6.2100 2

2 -5 5 71.5981 4.9692 1

-2 5 5 73.4296 5.2231 3

5 -2 5 65.9133 5.4696 3

5 2 -5 76.4001 5.1903 1

2 -5 -5 76.2388 6.2002 2

-2 5 -5 67.0132 6.8658 2

2 -5 -5 80.7428 5.3241 1

-5 5 -2 78.0195 7.1796 2

5 -5 -2 73.0327 5.2481 1

5 5 2 66.1402 6.1501 3

2 5 5 73.6167 6.1779 3

-5 2 -5 80.3641 7.1896 2

-5 -5 2 68.7828 6.2090 1

5 -2 -5 69.1863 6.3181 2

5 2 5 64.8414 5.8773 3

-2 -5 5 62.0262 5.8518 1

5 -2 -5 67.7973 4.9343 1

5 3 -5 1254.11 54.73 3

-5 5 3 1224.29 61.40 2

5 5 -3 1188.16 55.19 3

5 -3 5 1204.24 55.02 3

-3 -5 -5 1285.46 56.54 1

5 -5 3 1220.09 54.35 3

-5 -5 -3 1212.00 56.62 1

5 3 -5 1246.54 58.96 2

-5 -3 -5 1270.56 56.09 1

3 5 -5 1326.04 59.49 2

-5 3 5 1228.36 53.66 3

-3 5 5 1137.45 54.15 3

3 -5 5 1241.18 54.75 3

3 5 -5 1229.92 54.94 3

-5 -5 -3 1226.28 57.96 2

5 -5 3 1231.67 55.34 1

-3 -5 -5 1235.16 58.85 2

3 -5 5 1254.27 55.02 1

5 -3 5 1183.77 54.68 1

-5 -3 -5 1229.64 59.26 2

5 5 3 1145.54 54.22 3

5 3 5 1142.94 54.12 3

-5 -3 5 1200.80 54.08 1

-3 5 -5 1211.81 59.07 2

-5 -5 3 1184.50 54.77 1

3 -5 -5 1110.59 57.66 2

5 -3 -5 1211.16 53.47 1

5 -5 -3 1192.27 53.70 1

5 -3 -5 1165.64 58.05 2

3 -5 -5 1194.65 53.58 1

-5 3 -5 1207.96 58.87 2

3 5 5 1211.16 54.07 3

-3 -5 5 1218.66 54.74 1

-5 5 -3 1163.79 58.87 2

5 4 -5 591.119 29.248 2

5 -4 5 586.433 27.050 1

-5 4 5 548.821 32.190 2

-5 -4 -5 593.988 29.655 2

-5 -5 -4 594.205 29.003 2

-5 -5 -4 583.870 28.785 1

-4 5 5 567.207 32.045 2

-5 5 4 564.437 32.086 2

5 -4 5 580.901 27.667 3

-4 -5 -5 641.020 29.010 1

-4 -5 -5 573.195 29.312 2

5 5 -4 577.368 27.873 3

4 -5 5 609.158 27.255 1

5 4 -5 609.710 27.693 3

5 -5 4 612.211 27.524 1

4 -5 5 602.771 27.578 3

5 -5 4 574.085 27.320 3

4 5 -5 625.703 29.557 2

4 5 -5 585.539 27.750 3

-5 -4 -5 580.320 28.432 1

-4 -5 5 550.917 28.131 1

5 -4 -5 571.060 26.993 1

-5 5 -4 566.395 30.982 2

4 5 5 569.491 28.142 3

-5 4 -5 571.553 30.923 2

-5 -4 5 596.826 28.026 1

5 -5 -4 575.549 27.163 1

5 5 4 583.274 28.408 3

4 -5 -5 564.847 27.029 1

-5 -5 4 565.951 28.219 1

-4 5 -5 609.320 31.271 2

5 4 5 538.463 28.085 3

-5 5 5 620.244 34.713 2

-5 -5 -5 645.645 31.560 2

-5 -5 -5 677.088 31.030 1

5 5 -5 614.633 29.791 3

5 -5 5 637.097 29.727 3

5 -5 5 624.875 29.285 1

-5 5 -5 604.676 32.953 2

-5 -5 5 610.384 29.938 1

5 5 5 600.154 29.979 3

5 -5 -5 607.151 28.774 1

6 0 0 0.64785 1.71176 3

0 0 6 2.52150 1.42145 3

0 0 -6 8.11582 2.58832 2

0 6 0 11.2429 1.9745 3

-6 0 0 0.02595 1.98870 2

6 0 0 5.21442 1.64329 1

0 6 0 2.36391 1.85656 2

0 -6 0 3.95476 2.41653 1

0 6 -1 248.043 13.782 2

0 -6 -1 245.156 13.560 1

6 1 0 239.924 13.261 3

0 -6 1 251.790 13.812 1

1 0 -6 261.654 11.625 1

-6 -1 0 263.445 13.495 2

6 -1 0 249.141 12.457 1

6 1 0 254.049 12.650 1

0 6 1 255.889 12.490 3

1 0 6 246.627 12.461 3

6 -1 0 239.408 12.692 3

-1 0 -6 240.498 14.232 2

-1 0 6 257.874 12.208 3

0 6 -1 258.566 12.430 3

1 0 -6 225.279 14.145 2

-6 1 0 253.922 14.319 2

0 6 1 254.201 13.608 2

-6 2 0 31.5149 4.5919 2

2 0 6 43.7328 3.7666 3

-2 0 6 41.5463 3.2970 3

0 6 -2 37.6001 3.4697 3

6 2 0 38.2168 4.3311 3

6 -2 0 39.4487 3.5936 3

-6 -2 0 38.3973 3.6077 2

0 6 2 41.0386 3.7860 3

2 0 -6 42.7127 4.6190 2

-2 0 -6 52.5357 4.9524 2

0 6 -2 37.7102 4.2638 2

0 6 2 46.7022 4.3157 2

0 -6 2 37.0236 4.4977 1

2 0 -6 38.7613 2.8457 1

0 -6 -2 41.4954 4.3543 1

6 -2 0 39.0526 3.4878 1

6 2 0 42.9400 3.8296 1

0 6 3 195.778 10.837 3

3 0 6 216.732 11.102 3

6 3 0 197.609 11.632 3

0 -6 -3 215.954 11.724 1

0 -6 3 198.718 11.807 1

3 0 -6 214.958 10.168 1

-3 0 6 212.912 10.119 3

0 6 -3 208.096 10.520 3

-6 3 0 197.177 12.826 2

-3 0 -6 196.283 12.303 2

6 -3 0 196.518 10.554 1

0 6 3 206.502 12.015 2

3 0 -6 195.644 12.283 2

-6 -3 0 200.995 11.412 1

0 6 -3 187.053 12.087 2

0 6 -4 2.19256 1.69440 3

4 0 -6 -0.1565 1.1833 3

6 -4 0 4.06385 1.73020 1

0 6 -4 -2.5460 2.2651 2

-6 4 0 -2.6351 2.7650 2

-4 0 -6 1.24303 2.38420 2

0 -6 4 -2.4227 2.2983 1

4 0 -6 -1.3532 1.1909 1

0 6 4 2.84550 2.09500 3

4 0 6 5.11238 2.21283 3

4 0 -6 -2.5030 1.8328 2

0 -6 -4 1.49260 2.11274 1

6 4 0 -1.6810 1.8100 3

0 -6 -4 0.78787 1.77137 2

-6 -4 0 5.49151 2.54780 1

-6 5 0 166.609 11.372 2

0 6 -5 165.116 11.024 2

0 6 -5 152.382 8.746 3

-5 0 -6 154.783 10.737 2

0 -6 -5 162.278 9.827 2

0 6 5 158.462 9.388 3

6 -5 0 151.981 8.980 1

5 0 6 145.397 9.387 3

0 -6 5 156.716 9.732 1

-6 -5 0 176.478 10.563 1

-5 0 -6 163.262 8.295 1

5 0 -6 149.777 8.546 1

5 0 -6 149.001 7.989 3

6 5 0 148.322 9.614 3

0 -6 -5 139.444 9.353 1

5 0 -6 168.043 10.496 2

6 0 -1 243.024 12.443 3

6 0 1 258.432 13.463 3

1 6 0 278.223 13.377 2

1 6 0 265.174 12.705 3

-1 6 0 239.017 12.057 3

-1 6 0 260.929 13.972 2

-1 -6 0 242.745 14.034 1

0 -1 6 254.324 12.382 3

0 -1 -6 246.841 14.279 2

0 1 -6 228.236 14.336 2

-6 0 1 247.293 13.934 2

1 -6 0 254.774 13.598 1

6 0 -1 237.695 12.507 1

-6 0 -1 260.490 13.933 2

6 0 1 250.444 12.512 1

0 1 6 250.538 12.429 3

-1 1 6 29.4911 2.7092 3

6 -1 1 27.1077 2.6752 1

1 1 -6 26.6167 3.7495 2

-1 -6 -1 33.6619 4.1710 1

-6 -1 -1 29.0143 3.2779 2

1 -6 1 31.7961 3.7583 1

-1 6 1 34.5002 3.6912 2

6 -1 1 25.8825 3.2973 3

6 1 -1 25.3686 2.9949 3

1 6 -1 23.3410 2.9880 2

-1 -1 -6 30.5960 3.7997 2

1 6 -1 28.7428 2.9887 3

-1 6 1 36.9299 2.9798 3

-6 1 1 22.5410 3.8229 2

6 1 -1 26.0711 2.8013 1

1 -1 6 27.7369 2.7722 3

1 -6 -1 27.2736 3.5107 1

6 1 1 30.1814 3.7997 3

1 -1 -6 25.5390 3.6536 2

6 -1 -1 23.8490 2.7058 1

1 6 1 28.4885 3.0971 3

-1 6 -1 28.5821 3.5502 2

-1 1 -6 30.9129 3.9662 2

-6 1 -1 30.1150 3.6862 2

1 -1 -6 28.7751 2.1914 1

1 6 1 23.1536 2.8037 2

-6 -1 1 34.0048 3.4852 2

-1 -6 1 19.9477 3.6821 1

-1 6 -1 29.8403 2.5852 3

-1 -1 6 24.3826 2.5176 3

6 1 1 33.6428 2.9990 1

6 -1 -1 26.6272 2.4188 3

1 1 6 28.4716 2.9405 3

6 2 -1 191.333 10.869 3

1 -6 2 197.221 11.515 1

-1 -6 -2 210.602 11.777 1

-1 6 2 210.012 10.410 3

6 2 -1 194.487 10.645 1

6 -2 1 205.840 10.776 3

1 6 -2 196.169 10.458 3

-6 -2 -1 202.904 10.967 2

-6 2 1 201.582 12.521 2

-2 -1 -6 202.634 12.085 2

2 1 -6 208.311 12.356 2

1 6 -2 191.330 11.365 2

2 -1 6 196.570 10.513 3

-2 1 6 205.170 9.982 3

-1 6 2 198.893 12.065 2

6 -2 1 209.654 10.418 1

2 1 -6 204.283 9.867 1

2 1 -6 197.877 9.994 3

1 6 2 196.129 11.001 3

-6 2 -1 210.497 12.653 2

6 -2 -1 207.894 10.609 1

2 1 6 207.282 10.941 3

-1 6 -2 209.201 10.213 3

6 2 1 192.925 11.638 3

2 -1 -6 199.207 12.256 2

-6 -2 1 215.456 11.026 2

-2 1 -6 189.928 12.279 2

1 6 2 191.529 11.298 2

6 -2 -1 210.659 9.495 3

-1 -6 2 194.326 11.954 1

1 -6 -2 185.755 11.284 1

6 2 1 213.471 10.862 1

2 -1 -6 205.187 9.897 1

-1 6 -2 213.296 12.431 2

3 1 -6 141.308 7.251 1

-1 6 3 149.523 7.882 3

-1 -6 -3 123.142 7.941 2

-6 -3 -1 124.997 8.305 1

3 -1 6 133.356 7.893 3

1 6 -3 132.341 7.712 3

3 1 -6 139.803 7.228 3

6 -3 1 131.821 7.569 3

6 -3 1 131.389 7.523 1

-3 -1 -6 124.265 8.954 2

-6 3 1 125.468 9.676 2

1 6 -3 133.778 8.710 2

-3 1 6 142.415 7.170 3

-6 -3 -1 137.776 7.587 2

3 1 -6 153.278 9.504 2

1 -6 3 132.819 8.533 1

-1 6 3 129.256 9.178 2

6 3 -1 134.031 8.270 3

-1 -6 -3 126.803 8.664 1

-6 -3 1 145.532 8.511 1

3 -1 -6 138.001 7.139 1

1 6 3 136.612 8.269 3

1 -6 -3 129.502 8.295 1

-1 6 -3 139.336 9.514 2

3 1 6 138.991 8.125 3

-1 6 -3 132.372 7.337 3

-3 1 -6 129.869 9.342 2

6 3 1 126.494 8.743 3

-6 -3 1 133.079 7.154 2

6 -3 -1 123.943 7.505 1

-1 -6 3 123.256 9.000 1

3 -1 -6 130.078 9.058 2

-6 3 -1 122.382 9.534 2

-6 -4 -1 220.295 12.714 1

6 -4 1 224.237 11.543 1

-1 -6 -4 230.835 12.405 2

1 6 -4 220.832 13.222 2

4 -1 6 209.845 11.944 3

-4 1 6 233.824 10.888 3

-1 6 4 222.030 11.631 3

1 6 -4 225.265 11.570 3

1 -6 4 216.280 12.123 1

6 4 -1 221.355 12.176 3

-4 -1 -6 203.164 13.161 2

4 1 -6 235.929 13.432 2

-6 4 1 202.531 13.727 2

4 1 -6 215.602 11.249 1

-1 -6 -4 235.684 12.669 1

4 1 -6 228.575 11.013 3

4 1 6 223.687 12.094 3

1 -6 -4 203.670 12.626 2

-1 6 -4 219.097 11.255 3

1 6 4 244.443 12.429 3

-1 6 -4 221.118 13.870 2

-6 4 -1 190.757 13.662 2

-6 -4 1 213.299 12.546 1

4 -1 -6 216.622 13.290 2

-1 -6 4 203.966 12.889 1

6 -4 -1 221.999 11.576 1

1 -6 -4 230.044 12.290 1

4 -1 -6 235.206 11.305 1

6 4 1 251.558 13.193 3

-4 1 -6 224.765 13.829 2

1 6 -5 6.23822 2.11707 3

-1 6 5 5.67807 2.32660 3

-1 -6 -5 5.10966 2.43106 1

6 5 -1 7.78700 2.54045 3

1 -6 5 5.28003 2.36390 1

-5 -1 -6 8.23822 2.25839 1

6 -5 1 4.85545 2.10256 1

5 1 -6 5.90983 1.59631 3

-6 5 1 3.56062 3.45068 2

-5 -1 -6 3.67414 2.63744 2

-6 -5 -1 8.38437 3.20578 1

-5 1 6 9.91271 1.67030 3

5 -1 6 5.33199 2.49036 3

-1 -6 -5 6.84509 2.32359 2

5 1 -6 10.5910 2.9695 2

5 1 -6 5.33258 1.84883 1

1 6 -5 6.90791 2.99788 2

-1 -6 5 0.92599 2.73990 1

1 -6 -5 4.93182 2.37368 2

6 -5 -1 5.52859 2.04844 1

-6 5 -1 9.34781 3.71576 2

-1 6 -5 4.97922 3.18804 2

1 -6 -5 5.92295 2.13632 1

-6 -5 1 7.26616 3.04181 1

1 6 5 9.07793 2.80957 3

5 1 6 6.56180 2.58128 3

-1 6 -5 9.33178 2.09343 3

6 5 1 2.54183 2.71959 3

5 -1 -6 9.35319 1.97589 1

5 -1 -6 11.4951 3.1441 2

-5 1 -6 9.05187 3.42559 2

0 -2 -6 38.9424 2.6665 1

0 -2 6 41.4628 3.4001 3

2 6 0 44.4758 3.9935 3

-2 -6 0 41.6096 4.6355 1

6 0 2 37.6972 3.4782 1

0 2 6 42.9228 3.7393 3

-2 6 0 37.6185 4.3561 2

0 2 -6 39.3770 4.5928 2

-6 0 2 33.9166 4.4608 2

2 -6 0 41.8623 4.0691 1

-6 0 -2 36.9398 4.1561 2

6 0 -2 37.2064 3.2065 3

6 0 -2 39.0606 3.5671 1

-2 6 0 37.9255 2.9329 3

0 -2 -6 41.4361 4.4665 2

6 0 2 45.6566 4.4689 3

-2 -6 -1 216.005 12.103 1

-6 1 2 209.665 12.818 2

2 -6 1 187.780 11.120 1

1 2 -6 205.817 12.402 2

-2 6 1 205.244 9.822 3

-2 6 1 193.856 11.990 2

6 1 -2 202.380 10.729 1

-1 -2 -6 203.257 9.635 1

-6 -1 -2 203.840 11.602 2

1 2 -6 208.659 10.322 3

6 -1 2 198.578 10.356 1

-1 -2 -6 195.756 12.021 2

6 -1 2 202.112 11.348 3

-1 2 6 219.863 10.528 3

1 -2 6 194.607 10.484 3

6 1 -2 215.486 10.576 3

2 6 -1 214.762 10.927 3

-2 -6 1 185.870 11.891 1

2 6 1 195.186 11.111 3

6 -1 -2 201.234 9.727 3

1 -2 -6 201.472 12.213 2

6 1 2 217.649 10.792 1

6 1 2 185.559 11.636 3

-6 1 -2 201.889 12.320 2

2 -6 -1 188.517 11.166 1

-1 -2 6 225.485 10.589 1

1 -2 -6 207.631 9.917 1

-2 6 -1 203.849 12.427 2

-2 6 -1 185.535 9.885 3

-1 2 -6 201.787 12.618 2

-6 -1 2 237.634 12.276 2

2 6 1 234.550 10.896 2

6 -1 -2 204.517 10.666 1

1 2 6 207.613 10.848 3

2 6 -2 3860.09 163.37 3

2 -6 2 3604.74 166.20 1

2 -2 6 3597.05 163.04 3

2 2 -6 3695.04 162.69 3

-2 6 2 3931.48 162.43 3

-6 -2 -2 3826.83 175.54 2

6 -2 2 3644.92 165.45 1

-2 -2 -6 3710.04 176.34 2

-6 2 2 3821.63 177.27 2

2 2 -6 3906.71 176.96 2

2 6 -2 3919.02 175.42 2

6 -2 2 3576.79 163.41 3

-2 2 6 3971.37 162.75 3

6 2 -2 3786.43 163.29 3

6 2 -2 3527.60 165.90 1

-2 -6 -2 3967.85 167.22 1

-2 6 2 3655.49 176.74 2

-2 6 -2 3583.82 161.05 3

2 -2 -6 3694.38 163.76 1

-6 -2 2 4272.62 174.37 2

-2 -6 2 3766.00 165.76 1

-2 6 -2 3550.57 175.26 2

2 6 2 3671.16 173.86 2

6 -2 -2 3627.48 164.34 1

-6 2 -2 3749.64 175.39 2

6 2 2 3558.73 163.01 3

2 -6 -2 3652.25 164.96 1

2 2 6 3785.48 162.10 3

-2 2 -6 3526.66 175.50 2

-2 -2 6 3971.74 164.38 1

6 2 2 4035.66 164.69 1

2 -2 -6 3737.41 175.22 2

2 6 2 3616.32 162.34 3

6 -2 -2 3647.62 160.44 3

-2 6 3 194.991 10.043 3

2 6 -3 181.900 10.442 3

-3 2 6 213.343 9.925 3

6 -3 2 197.330 10.207 1

3 -2 6 192.443 10.568 3

3 2 -6 200.312 10.004 3

6 3 -2 196.239 10.820 1

6 3 -2 194.473 10.771 3

-6 3 2 203.490 13.087 2

-3 -2 -6 198.310 11.929 2

-2 6 3 183.163 12.336 2

2 6 -3 209.617 11.259 2

-6 -3 -2 192.138 11.093 1

3 2 -6 210.376 12.329 2

6 -3 2 200.250 10.548 3

-2 -6 -3 187.452 10.617 2

-2 -6 -3 218.965 11.995 1

2 -6 3 191.087 10.748 1

-6 -3 -2 189.322 10.706 2

-3 -2 -6 192.701 10.014 1

-2 6 -3 206.848 12.699 2

-3 2 -6 221.135 13.014 2

-6 3 -2 205.634 12.709 2

2 -6 -3 197.780 10.912 1

3 -2 -6 199.669 9.985 1

2 6 3 198.430 11.275 3

-2 6 -3 183.511 9.869 3

-3 -2 6 211.503 10.518 1

6 -3 -2 184.964 10.392 1

3 2 6 195.809 10.908 3

6 3 2 200.144 11.954 3

3 -2 -6 181.964 11.928 2

-2 -6 3 190.814 11.865 1

2 6 -4 12.0951 2.7943 2

-4 -2 -6 10.6128 2.5552 1

-6 4 2 5.87663 3.34849 2

-2 6 4 11.6734 3.5925 2

-6 -4 -2 16.5443 3.4417 1

6 -4 2 15.7744 2.4266 3

4 2 -6 10.2211 2.1718 1

-2 -6 -4 12.3011 2.3344 2

6 -4 2 12.9198 2.2662 1

-2 6 4 14.8322 2.4812 3

4 2 -6 10.2574 2.8330 2

-4 2 6 9.78093 1.69517 3

-2 -6 -4 15.5690 3.3513 1

4 -2 6 11.4814 2.6033 3

4 2 -6 14.0837 2.1301 3

-6 -4 -2 11.0810 1.8892 2

-4 -2 -6 9.15357 2.89892 2

2 6 -4 8.17971 2.23873 3

2 -6 4 11.3640 2.3971 1

6 4 -2 9.65973 2.55838 3

-4 2 -6 10.7941 3.5193 2

-2 -6 4 8.37452 3.23022 1

2 6 4 12.5804 3.0617 3

-2 6 -4 16.7997 2.3014 3

4 -2 -6 13.9742 3.2871 2

2 -6 -4 8.30119 2.48068 1

6 4 2 5.88890 3.04027 3

4 -2 -6 10.4045 2.0189 1

4 2 6 18.6365 3.1569 3

-6 -4 2 11.5706 2.9817 1

-2 6 -4 7.37341 3.53217 2

6 -4 -2 9.15089 2.21295 1

-6 4 -2 9.30799 3.49911 2

5 -2 6 139.518 8.738 3

2 6 -5 140.805 8.278 3

5 2 -6 135.300 8.039 1

2 6 -5 140.507 9.425 2

-6 -5 -2 120.373 9.156 1

5 2 -6 140.155 7.751 3

6 5 -2 131.954 8.652 3

-2 -6 -5 138.165 8.612 2

2 -6 5 142.761 8.232 1

-5 2 6 144.236 7.278 3

-2 6 5 135.834 8.163 3

6 -5 2 139.806 8.206 1

5 2 -6 143.702 9.377 2

-5 -2 -6 149.655 9.718 2

-6 5 2 126.755 10.388 2

-5 -2 -6 147.265 8.373 1

-2 -6 -5 138.098 9.048 1

2 -6 -5 144.675 8.696 1

-6 5 -2 129.764 10.519 2

5 2 6 148.326 9.398 3

6 5 2 122.960 9.478 3

-6 -5 2 153.170 9.808 1

5 -2 -6 147.864 8.140 1

-2 6 -5 138.761 7.993 3

6 -5 -2 143.885 8.463 1

-2 6 -5 144.263 10.849 2

2 -6 -5 128.310 9.409 2

-5 2 -6 143.744 10.526 2

5 -2 -6 142.313 9.943 2

-2 -6 5 133.570 9.632 1

2 6 5 136.019 9.197 3

0 3 6 210.792 11.037 3

6 0 -3 212.540 10.995 1

-6 0 3 228.163 13.330 2

0 -3 -6 207.755 10.376 1

-3 -6 0 211.651 12.286 1

0 3 -6 217.907 13.109 2

6 0 3 226.022 10.827 1

6 0 3 181.378 11.672 3

-3 6 0 201.051 9.768 3

6 0 -3 210.908 10.259 3

-3 6 0 212.449 12.807 2

3 6 0 219.033 11.495 3

3 -6 0 211.584 11.454 1

-6 0 -3 216.090 12.581 2

0 3 -6 208.228 10.733 3

0 -3 -6 215.031 12.466 2

6 1 -3 137.151 7.842 1

1 3 -6 136.032 7.567 3

6 1 -3 145.876 7.513 3

1 3 -6 129.564 9.489 2

6 -1 3 130.655 7.410 1

-1 3 6 143.813 7.782 3

-1 -3 -6 143.668 7.306 1

-3 6 1 133.860 9.319 2

-1 -3 -6 140.119 8.984 2

-6 1 3 140.894 10.241 2

-3 -6 -1 147.570 9.320 1

3 6 -1 132.930 8.135 3

-6 -1 -3 138.880 8.820 2

1 -3 6 129.953 7.752 3

3 -6 1 126.020 8.096 1

6 -1 3 133.094 8.419 3

6 -1 -3 132.605 7.665 1

-3 -6 1 128.397 8.883 1

-3 6 -1 132.760 6.729 3

3 -6 -1 128.257 8.048 1

-1 3 -6 140.313 9.690 2

-6 -1 3 148.841 9.478 2

6 1 3 121.639 8.699 3

1 -3 -6 135.065 7.099 1

6 1 3 148.850 7.749 1

3 6 1 135.600 8.478 3

1 3 6 139.313 8.029 3

1 -3 -6 128.849 8.856 2

-1 -3 6 141.156 8.001 1

-3 6 -1 123.903 9.227 2

6 -1 -3 131.360 6.674 3

-6 1 -3 134.785 9.257 2

6 2 -3 190.611 10.758 1

6 -2 3 207.842 11.078 3

-6 -2 -3 204.830 11.126 1

-2 3 6 212.824 10.436 3

6 -2 3 191.037 10.202 1

-3 6 2 200.557 12.745 2

6 2 -3 214.143 10.729 3

-6 -2 -3 195.580 11.469 2

2 3 -6 206.244 10.391 3

-6 2 3 207.778 13.583 2

3 6 -2 193.137 10.850 3

-2 -3 -6 193.382 10.265 1

2 -3 6 217.667 10.779 3

-3 -6 -2 214.153 12.078 1

2 3 -6 219.824 12.703 2

-2 -3 -6 187.445 11.809 2

3 -6 2 192.720 10.952 1

6 2 3 205.593 12.152 3

6 -2 -3 197.915 10.592 1

3 6 2 192.967 11.461 3

-3 6 -2 207.313 12.810 2

-3 6 -2 196.663 9.833 3

2 3 6 207.379 11.171 3

-3 -6 2 201.277 12.044 1

-2 3 -6 184.208 12.653 2

-6 -2 3 225.028 12.250 2

-6 2 -3 184.559 12.464 2

2 -3 -6 200.535 12.192 2

3 6 2 215.650 10.698 2

3 -6 -2 209.304 11.088 1

2 -3 -6 195.849 10.145 1

6 -2 -3 188.138 9.193 3

-2 -3 6 225.393 11.302 1

-3 -3 -6 218.593 12.693 1

3 3 -6 254.792 14.738 2

-6 3 3 263.799 16.237 2

3 -6 3 241.543 13.014 1

-3 -3 -6 255.779 14.311 2

-6 -3 -3 245.390 13.639 1

3 -3 6 241.515 12.963 3

6 -3 3 254.295 13.003 3

6 3 -3 270.262 13.228 3

-6 -3 -3 251.602 13.633 2

3 3 -6 253.786 12.617 3

6 -3 3 252.678 12.509 1

-3 -6 -3 249.289 12.992 2

3 6 -3 278.854 13.204 2

-3 -6 -3 276.050 14.391 1

6 3 -3 237.118 13.091 1

-3 3 6 265.090 12.417 3

-3 6 3 229.267 15.206 2

3 6 -3 245.617 13.057 3

-3 -3 6 252.666 12.800 1

3 -3 -6 246.595 11.922 1

-3 6 -3 216.499 14.472 2

-3 6 -3 246.916 11.600 3

-6 3 -3 230.248 14.721 2

-3 3 -6 237.932 14.864 2

-3 -6 3 224.364 13.485 1

3 3 6 245.975 13.000 3

3 -3 -6 215.965 13.904 2

3 -6 -3 232.301 12.611 1

3 6 3 254.474 13.408 3

6 3 3 226.399 13.642 3

6 -3 -3 232.610 12.216 1

-3 -6 -4 150.461 8.670 2

-3 6 4 156.518 11.213 2

4 3 -6 156.564 9.921 2

-3 -6 -4 154.829 9.935 1

6 4 -3 144.618 8.856 3

6 -4 3 149.104 8.520 3

3 -6 4 142.989 8.293 3

-4 -3 -6 145.184 8.828 1

-6 4 3 148.161 11.543 2

4 -3 6 143.780 8.805 3

3 6 -4 146.826 8.809 3

3 6 -4 181.792 9.331 2

-4 -3 -6 152.585 9.782 2

4 3 -6 144.706 8.236 3

6 -4 3 142.869 8.253 1

-4 3 6 153.138 7.925 3

-6 -4 -3 139.445 8.630 2

3 -6 4 164.423 8.740 1

-6 -4 -3 130.963 9.436 1

-6 -4 3 139.570 9.366 1

4 -3 -6 126.041 9.733 2

-3 6 -4 161.234 11.269 2

6 4 3 141.421 10.020 3

6 -4 -3 146.141 8.500 1

3 6 4 148.760 9.552 3

4 3 6 134.760 9.183 3

-4 3 -6 164.990 11.082 2

-3 -6 4 145.443 9.817 1

-4 -3 6 160.074 9.055 1

3 -6 -4 145.927 8.772 1

4 -3 -6 148.050 8.253 1

-6 4 -3 148.349 11.050 2

5 3 -6 33.1521 4.1391 2

3 -6 5 40.2869 3.6558 1

6 -5 3 38.1798 3.5828 3

3 6 -5 33.3704 3.7817 3

-6 -5 -3 37.9735 4.7383 1

3 6 -5 35.9467 4.2362 2

6 5 -3 31.2754 3.9456 3

-3 -6 -5 29.2010 3.5075 2

-5 -3 -6 39.2125 4.1217 1

3 -6 5 40.0705 3.8482 3

-6 5 3 40.3286 5.9174 2

-3 -6 -5 40.9603 4.7811 1

5 3 -6 37.8418 3.5865 3

-3 6 5 32.8292 5.6100 2

-5 -3 -6 36.1159 4.5532 2

5 -3 6 35.8918 4.1807 3

-5 3 6 37.2853 3.2390 3

6 -5 3 38.4344 3.5741 1

5 -3 -6 36.2630 4.7939 2

5 3 6 38.8657 4.7795 3

-5 -3 6 31.6360 4.0065 1

-6 -5 3 36.6902 4.8417 1

-3 -6 5 29.8693 4.6009 1

3 6 5 40.1050 4.8506 3

3 -6 -5 33.1442 3.7741 1

5 -3 -6 33.7893 3.4511 1

6 -5 -3 29.7519 3.6105 1

-5 3 -6 41.9568 5.6333 2

-3 6 -5 33.7684 5.5296 2

6 5 3 26.8061 4.5361 3

-6 5 -3 28.8982 5.3227 2

6 0 -4 5.26460 1.80316 1

-6 0 4 0.89348 2.84715 2

4 -6 0 4.01286 2.06958 1

6 0 4 -0.1031 2.1419 3

0 4 6 3.33713 1.97836 3

-4 -6 0 8.96375 2.93502 1

0 -4 -6 2.25924 2.14922 2

-6 0 -4 0.10873 2.37607 2

6 0 -4 1.64100 1.21938 3

4 6 0 7.64149 2.46247 3

6 0 4 1.05989 1.46357 1

0 -4 -6 -0.9234 1.3646 1

0 4 -6 -1.1586 2.2700 2

-4 6 0 -1.3352 2.4420 2

0 4 -6 4.51003 1.69447 3

0 -4 6 0.58155 1.86615 1

1 4 -6 241.570 11.889 3

-6 1 4 228.741 14.754 2

-1 -4 -6 223.703 13.216 2

-4 6 1 236.929 14.204 2

-1 -4 -6 235.469 11.912 1

6 -1 4 228.293 11.530 1

6 -1 4 238.669 12.709 3

1 -4 6 232.351 11.259 1

1 4 -6 228.053 14.015 2

6 1 -4 237.258 12.101 1

6 1 -4 237.947 11.482 3

4 -6 1 238.434 10.891 3

1 -4 6 237.162 12.033 3

4 -6 1 206.666 12.148 1

4 6 -1 219.131 12.348 3

-4 -6 -1 247.730 13.386 1

-6 -1 -4 225.622 13.415 2

-1 4 6 232.439 11.941 3

-6 -1 4 243.858 13.941 2

1 -4 -6 220.433 11.284 1

-1 4 -6 227.809 14.113 2

6 1 4 212.540 12.791 3

1 -4 -6 223.061 13.070 2

-4 -6 1 223.821 12.962 1

-1 -4 6 196.724 12.058 1

6 -1 -4 224.255 11.612 1

6 1 4 238.172 11.593 1

4 6 1 220.402 12.456 3

6 -1 -4 226.711 10.523 3

1 4 6 214.941 11.985 3

-4 6 -1 233.109 13.879 2

4 -6 -1 218.780 11.971 1

-6 1 -4 229.333 13.781 2

-6 -2 -4 7.22961 2.74853 2

4 -6 2 15.0252 2.6512 1

2 4 -6 19.2960 2.5488 3

6 -2 4 13.0135 2.0409 1

4 -6 2 11.5718 1.8506 3

-2 -4 -6 10.9292 2.8214 2

-6 -2 -4 14.4545 2.7437 1

-2 -4 -6 12.2474 2.5853 1

2 4 -6 8.30902 3.05924 2

-4 6 2 5.86513 3.38286 2

-2 4 6 8.50998 2.15461 3

6 2 -4 12.3674 2.2541 3

2 -4 6 14.0786 1.6648 1

4 6 -2 12.8439 2.5547 3

2 -4 6 12.5448 2.4358 3

6 -2 4 7.46469 2.43146 3

6 2 -4 16.3508 2.5763 1

-6 2 4 13.3054 4.3335 2

-4 -6 -2 10.6956 3.1881 1

-4 6 -2 8.90928 3.42765 2

2 4 6 11.2534 2.8841 3

6 -2 -4 15.2699 2.4287 1

6 -2 -4 7.91732 1.28872 3

-6 -2 4 12.0342 3.2556 2

2 -4 -6 13.4214 2.1270 1

6 2 4 11.9417 3.3312 3

2 -4 -6 13.8978 3.1413 2

4 6 2 12.9948 3.2108 3

-6 2 -4 10.8252 3.4935 2

-2 4 -6 11.0631 3.5791 2

4 -6 -2 15.2679 2.7111 1

-4 -6 2 12.2583 3.3438 1

-2 -4 6 7.53264 2.82696 1

-3 -4 -6 148.889 9.283 2

-4 -6 -3 145.198 7.892 2

6 -3 4 146.090 8.591 3

-4 6 3 138.212 10.794 2

6 3 -4 137.961 8.601 1

-6 -3 -4 138.599 9.045 2

-6 -3 -4 134.494 8.946 1

-6 3 4 143.366 11.477 2

6 -3 4 132.780 7.742 1

-3 -4 -6 140.877 8.651 1

-3 4 6 145.184 7.898 3

3 -4 6 144.720 8.420 3

4 -6 3 144.992 7.846 3

4 6 -3 153.655 8.785 3

3 4 -6 144.631 8.267 3

3 4 -6 160.700 10.005 2

4 -6 3 132.480 8.356 1

-4 -6 -3 155.751 9.798 1

6 3 -4 154.330 8.530 3

3 -4 6 145.596 7.237 1

-4 -6 3 142.243 9.928 1

-4 6 -3 141.905 10.810 2

-6 3 -4 151.906 10.970 2

-3 -4 6 143.559 9.290 1

3 -4 -6 143.703 8.147 1

3 -4 -6 141.744 9.865 2

3 4 6 133.628 9.237 3

4 6 3 156.663 9.763 3

4 -6 -3 140.669 8.745 1

-3 4 -6 141.362 10.736 2

6 -3 -4 146.877 8.376 1

6 3 4 146.820 10.000 3

-6 -3 4 155.816 9.597 2

6 4 -4 10.6597 2.6688 3

-4 6 4 13.9490 4.3540 2

4 4 -6 12.6234 2.4556 3

4 -6 4 7.34761 2.16898 3

4 4 -6 15.1984 3.2018 2

-6 4 4 9.67015 4.32043 2

-4 -6 -4 9.90726 3.39551 1

-6 -4 -4 11.1997 2.5644 2

-4 -4 -6 10.8214 2.9587 1

-4 -6 -4 11.9999 2.2141 2

-4 4 6 18.8013 2.3373 3

-6 -4 -4 5.31343 3.04021 1

4 -6 4 14.4204 2.6490 1

6 -4 4 17.7609 2.3362 1

6 -4 4 15.8717 2.7025 3

4 -4 6 15.1574 2.8306 3

4 6 -4 8.35636 2.55171 3

-4 -4 -6 11.8598 2.8656 2

-4 -4 6 15.6151 3.3373 1

-4 6 -4 13.2224 4.2679 2

-4 -6 4 10.5965 3.4839 1

4 -4 -6 14.1665 2.3531 1

-6 -4 4 10.0098 3.2150 1

4 6 4 16.3621 3.7315 3

4 -4 -6 4.06608 2.72082 2

-4 4 -6 12.2743 4.2149 2

4 4 6 13.0870 3.3399 3

6 4 4 15.4124 3.7884 3

4 -6 -4 13.0736 2.6629 1

6 -4 -4 13.5805 2.4614 1

-6 4 -4 22.0650 4.6291 2

5 -4 6 126.496 7.999 3

4 -6 5 110.841 7.358 3

-4 6 5 107.480 10.239 2

-4 -6 -5 115.177 7.546 2

-6 -5 -4 109.629 8.471 1

6 -5 4 112.539 7.034 1

6 5 -4 119.723 7.788 3

5 4 -6 114.281 7.281 3

-5 -4 -6 112.419 8.158 2

4 6 -5 122.235 7.754 3

-5 -4 -6 129.077 8.390 1

6 -5 4 121.403 7.612 3

-4 -6 -5 113.788 8.424 1

5 4 -6 110.406 7.857 2

4 -6 5 120.043 7.189 1

-6 5 4 114.503 10.609 2

-4 -6 5 119.778 8.972 1

6 -5 -4 103.935 7.325 1

5 -4 -6 111.678 9.005 2

-5 -4 6 123.751 8.426 1

4 6 5 123.935 8.907 3

4 -6 -5 116.333 7.572 1

5 4 6 114.429 8.775 3

5 -4 -6 110.739 7.236 1

-5 4 -6 121.735 10.131 2

-6 5 -4 115.436 10.011 2

-4 6 -5 112.146 10.113 2

6 5 4 98.7913 8.7613 3

-6 -5 4 124.953 8.901 1

0 -5 -6 148.665 8.875 1

0 5 6 154.131 9.367 3

6 0 -5 169.907 10.542 2

-6 0 -5 154.548 10.481 2

0 5 -6 155.001 8.797 3

-5 6 0 169.406 11.186 2

6 0 -5 160.080 8.907 1

0 5 -6 146.246 10.953 2

5 6 0 155.477 9.603 3

-5 -6 0 165.860 10.441 1

6 0 5 173.207 9.999 3

0 -5 -6 156.575 9.917 2

6 0 5 164.191 8.504 1

6 0 -5 157.265 8.050 3

5 -6 0 162.042 9.256 1

0 -5 6 142.382 9.125 1

-5 6 1 6.04648 3.37922 2

-1 -5 -6 5.77135 2.17146 1

-6 -1 -5 10.9749 3.0654 2

6 1 -5 8.73109 1.72608 3

5 -6 1 10.4598 2.4054 1

6 1 -5 10.7990 2.1900 1

6 -1 5 4.41845 2.46373 3

-6 -1 -5 8.85075 2.10029 1

6 1 -5 4.81744 2.23837 2

1 -5 6 9.79240 2.06357 1

-5 -6 -1 3.72148 2.78822 1

-1 -5 -6 6.48059 2.35571 2

6 -1 5 6.74615 1.76006 1

1 5 -6 8.27912 2.15885 3

1 5 -6 9.81549 3.23538 2

5 6 -1 5.25691 2.31932 3

-1 5 6 7.13151 2.34177 3

1 -5 -6 5.27052 2.60202 2

-5 -6 1 8.64922 3.22969 1

-1 -5 6 0.55843 2.64218 1

5 -6 -1 8.74579 2.33361 1

-5 6 -1 7.52793 3.36283 2

6 -1 -5 9.41339 2.90108 2

-1 5 -6 2.12359 2.96735 2

-6 1 -5 8.57767 3.33327 2

-1 5 -6 7.04081 2.05763 3

6 -1 -5 11.4653 2.2859 1

6 -1 -5 5.84510 1.30436 3

1 5 6 5.23842 2.58784 3

5 6 1 4.95325 2.64054 3

6 1 5 6.28888 2.77351 3

1 -5 -6 9.78224 2.19666 1

-5 6 2 129.496 10.333 2

-6 2 5 131.311 11.236 2

5 -6 2 151.964 8.569 1

5 -6 2 147.352 7.550 3

5 6 -2 148.674 8.747 3

2 -5 6 141.819 7.634 1

-6 -2 -5 138.875 8.629 1

6 2 -5 132.857 8.871 2

-6 2 5 141.968 7.107 3

-2 -5 -6 134.769 8.715 1

6 -2 5 140.995 8.762 3

6 2 -5 153.495 8.056 3

2 5 -6 139.235 8.294 3

-2 -5 -6 135.442 8.935 2

2 5 -6 166.307 10.260 2

-6 -2 -5 129.070 9.419 2

-2 5 6 137.564 8.149 3

2 -5 6 160.386 8.628 3

-5 -6 -2 139.373 9.483 1

6 -2 5 134.897 7.512 1

6 2 -5 124.609 8.150 1

5 -6 -2 142.517 8.595 1

2 -5 -6 144.943 8.142 1

2 -5 -6 144.749 9.655 2

6 -2 -5 137.909 8.139 1

-5 -6 2 146.589 9.787 1

-5 6 -2 127.240 10.405 2

-2 5 -6 126.129 10.467 2

5 6 2 155.504 9.636 3

6 2 5 134.161 9.556 3

2 5 6 136.332 9.163 3

-2 -5 6 134.225 9.319 1

-6 2 -5 136.483 10.361 2

3 5 -6 31.0439 3.6287 3

-5 6 3 31.7122 5.5268 2

5 6 -3 35.4090 4.0766 3

-6 -3 -5 36.7325 4.3067 1

6 -3 5 41.8002 4.1821 3

6 3 -5 36.2953 3.8179 2

-3 -5 -6 32.8961 4.0106 1

6 3 -5 39.6722 3.7334 3

5 -6 3 36.6409 3.3586 3

3 -5 6 36.6941 3.0123 1

3 -5 6 33.4786 3.7265 3

-6 -3 -5 38.8310 4.3299 2

6 -3 5 31.5031 2.9831 1

5 -6 3 38.1190 3.8024 1

3 5 -6 37.3005 4.5941 2

-3 5 6 35.9609 3.4877 3

-6 3 5 36.0854 6.1503 2

-5 -6 -3 39.1371 4.8137 1

-3 -5 -6 40.5401 4.1948 2

3 -5 -6 33.9267 3.5062 1

-3 5 -6 33.3935 5.5403 2

6 3 5 32.4961 4.7785 3

-6 -3 5 27.5823 3.9602 1

-5 6 -3 32.2647 5.5280 2

-5 -6 3 36.3429 4.8981 1

6 -3 -5 39.5905 3.7382 1

5 6 3 33.1979 4.7188 3

-3 -5 6 33.9960 4.5751 1

-6 3 -5 39.5425 5.6789 2

5 -6 -3 40.7064 4.0127 1

3 5 6 36.2318 4.6623 3

3 -5 -6 33.4008 4.5973 2

6 -4 5 116.906 6.797 1

-6 4 5 139.832 11.525 2

-4 -5 -6 120.480 8.107 2

4 5 -6 118.782 8.313 2

6 -4 5 121.733 7.867 3

-5 -6 -4 113.981 8.715 1

4 -5 6 121.872 6.611 1

-5 6 4 100.281 9.991 2

-6 -4 -5 111.009 8.106 2

4 -5 6 121.754 7.832 3

4 5 -6 110.517 7.328 3

-4 -5 -6 119.825 8.436 1

5 -6 4 122.464 7.375 3

5 6 -4 103.319 7.641 3

-6 -4 -5 110.810 8.208 1

5 -6 4 120.254 7.370 1

6 4 -5 113.392 7.497 3

6 4 5 99.7582 8.6521 3

4 5 6 100.667 8.470 3

-6 -4 5 127.313 8.579 1

-4 5 -6 119.140 10.370 2

-5 6 -4 110.958 9.972 2

4 -5 -6 122.417 7.535 1

-5 -6 4 104.777 8.622 1

5 -6 -4 114.241 7.575 1

4 -5 -6 115.744 9.070 2

6 -4 -5 117.897 7.487 1

5 6 4 125.347 9.111 3

-6 4 -5 117.129 10.121 2

-4 -5 6 116.774 8.631 1

-6 5 5 58.5648 7.9964 2

-5 6 5 52.0400 7.6253 2

5 6 -5 59.8033 5.4193 3

5 -5 6 55.2986 5.3521 3

5 -6 5 56.2418 4.9885 1

6 5 -5 61.3008 5.4323 3

-6 -5 -5 68.5430 6.1732 1

-5 -5 -6 76.2268 6.5394 1

6 -5 5 66.5171 5.6221 3

5 -6 5 69.8206 5.5387 3

-5 -6 -5 67.7139 6.3650 1

-5 5 6 58.0366 7.8814 2

-5 -5 -6 58.6731 5.7431 2

5 5 -6 64.3379 5.3217 3

6 -5 5 65.2416 4.8230 1

5 -5 -6 65.8528 5.3816 1

6 5 5 59.9035 6.5073 3

-5 5 -6 66.9874 7.7998 2

5 6 5 70.8821 6.8774 3

-6 -5 5 57.3516 6.2477 1

5 -6 -5 58.4748 5.3550 1

-5 -6 5 61.6542 6.4462 1

5 5 6 65.8774 6.5476 3

6 -5 -5 65.0994 5.4694 1

-6 5 -5 62.1986 7.7714 2

-5 -5 6 70.3284 6.5308 1

-5 6 -5 63.7098 7.8552 2

0 -6 -6 1.88892 2.20165 1

6 -6 0 3.95771 2.23695 1

-6 -6 0 3.78629 2.97100 1

-6 0 -6 4.84412 3.09834 2

-6 6 0 2.79059 3.52181 2

0 -6 -6 -0.6088 1.9866 2

0 6 -6 5.45291 3.33348 2

6 0 -6 -1.5015 2.2918 2

6 0 -6 -0.8832 1.6723 1

6 0 6 9.31682 2.96871 3

6 6 0 8.47944 2.69462 3

0 6 6 -2.4041 2.1830 3

6 0 -6 0.94351 1.30962 3

0 -6 6 0.60196 2.44541 1

0 6 -6 4.09968 1.92351 3

-6 6 1 277.201 17.282 2

-1 -6 -6 304.487 15.850 2

1 -6 6 249.504 14.399 1

6 6 -1 279.532 15.193 3

-6 -1 -6 261.682 16.376 2

-6 -6 -1 264.012 15.950 1

1 6 -6 302.569 14.639 3

-6 -1 -6 293.435 14.550 1

-1 -6 -6 273.195 15.035 1

6 -1 6 289.814 15.325 3

6 1 -6 290.483 13.810 3

6 1 -6 271.460 14.411 1

1 6 -6 302.242 17.204 2

6 -6 1 288.462 14.863 1

6 1 -6 295.474 16.390 2

-1 6 6 302.142 14.989 3

-6 -6 1 292.888 16.155 1

-6 1 -6 281.204 17.006 2

6 -1 -6 292.248 13.343 3

-1 -6 6 244.017 15.279 1

1 -6 -6 270.543 14.669 1

6 1 6 288.072 15.596 3

6 -6 -1 285.551 14.868 1

-6 6 -1 272.833 17.287 2

6 -1 -6 277.237 16.432 2

-1 6 -6 291.667 14.292 3

1 -6 -6 272.773 15.966 2

6 -1 -6 289.934 14.457 1

1 6 6 284.099 15.244 3

6 6 1 283.344 15.665 3

-1 6 -6 287.193 17.595 2

6 2 -6 2395.61 114.15 2

-2 6 6 2409.84 105.54 3

2 -6 6 2444.76 105.86 3

6 2 -6 2307.41 107.20 1

-6 -2 -6 2482.53 107.46 1

2 6 -6 2603.01 114.99 2

-2 -6 -6 2514.53 113.77 2

2 6 -6 2348.33 105.65 3

6 6 -2 2301.77 106.09 3

-6 6 2 2282.27 115.44 2

6 -6 2 2441.12 107.40 1

-6 2 6 2385.46 104.44 3

-6 -2 -6 2415.25 114.55 2

6 -2 6 2385.91 106.24 3

2 -6 6 2506.87 106.93 1

-6 -6 -2 2388.99 108.70 1

6 2 -6 2395.06 105.10 3

-2 -6 -6 2396.93 107.98 1

-6 -6 2 2298.18 104.47 1

6 -2 -6 2324.58 110.52 2

2 -6 -6 2341.67 103.11 1

-6 6 -2 2236.61 110.97 2

2 6 6 2313.62 102.31 3

-2 6 -6 2276.20 111.34 2

2 -6 -6 2367.82 109.91 2

6 -6 -2 2281.35 103.14 1

-6 2 -6 2397.99 111.10 2

-2 -6 6 2182.10 103.96 1

6 2 6 2346.03 102.75 3

6 6 2 2327.24 102.87 3

6 -2 -6 2328.01 102.80 1

6 -3 6 254.665 13.745 3

3 6 -6 243.997 13.323 3

-6 6 3 238.819 16.326 2

6 6 -3 233.417 13.565 3

6 3 -6 235.947 12.782 3

-6 -3 -6 256.855 13.744 1

-6 3 6 220.312 16.821 2

-3 -6 -6 267.510 14.082 2

6 3 -6 266.278 14.302 2

-3 -6 -6 259.769 14.176 1

3 -6 6 258.616 12.725 1

-6 -3 -6 247.868 14.679 2

3 6 -6 248.629 14.625 2

3 -6 6 250.627 13.409 3

6 -6 3 252.895 13.252 1

-6 -6 -3 242.292 14.541 1

-6 6 -3 242.155 16.280 2

-6 3 -6 252.073 16.292 2

6 6 3 262.452 14.908 3

3 -6 -6 250.578 15.101 2

3 6 6 238.282 14.276 3

6 3 6 238.932 14.497 3

-3 6 -6 277.245 16.851 2

6 -3 -6 248.750 13.137 1

3 -6 -6 246.315 13.275 1

-3 -6 6 256.870 14.687 1

6 -6 -3 221.830 13.240 1

-6 -6 3 253.671 14.878 1

-6 -3 6 240.921 13.781 1

-4 -6 -6 35.8722 4.8114 1

-4 -6 -6 28.8012 3.8031 2

6 6 -4 34.0964 4.1477 3

-6 4 6 36.0000 6.8343 2

-6 -4 -6 29.9625 4.3156 2

-6 6 4 29.6527 5.9583 2

4 -6 6 33.9947 4.0887 3

6 4 -6 32.2720 3.7325 3

-6 -6 -4 39.0292 5.0420 1

6 -6 4 35.3222 3.7784 1

-6 -4 -6 38.0863 4.6084 1

4 6 -6 35.2735 3.9321 3

4 -6 6 33.1117 3.3246 1

-4 6 6 35.3466 6.3536 2

6 -4 6 32.6338 4.3820 3

6 -6 4 24.9023 3.7217 3

-4 6 -6 40.0712 6.4421 2

-6 -6 4 37.0210 5.3362 1

6 -4 -6 31.5742 3.7482 1

6 -6 -4 30.6035 3.9033 1

6 4 6 27.8653 4.9100 3

-6 6 -4 32.7732 5.8725 2

6 6 4 33.2552 5.1142 3

4 -6 -6 30.0120 3.7473 1

-6 4 -6 38.9662 6.1437 2

4 6 6 30.6269 4.8986 3

-4 -6 6 31.1265 4.8198 1

-6 -4 6 36.4571 4.7800 1

6 -6 5 165.735 8.799 1

-5 -6 -6 146.835 9.213 2

6 5 -6 143.445 9.114 3

-5 -6 -6 159.219 10.182 1

-6 -6 -5 145.406 10.236 1

6 6 -5 131.066 9.161 3

-6 -5 -6 168.657 10.247 1

6 -6 5 151.412 9.317 3

5 -6 6 129.217 9.162 3

5 6 -6 138.515 9.112 3

-5 6 6 131.314 12.354 2

-6 6 5 140.030 12.728 2

6 -5 6 131.616 9.375 3

-6 -5 -6 153.383 9.702 2

-6 5 6 127.470 12.526 2

-6 -5 6 142.678 10.356 1

-5 -6 6 127.920 10.198 1

-6 5 -6 148.425 12.263 2

-6 -6 5 143.249 10.552 1

-6 6 -5 151.863 12.485 2

5 -6 -6 152.993 9.356 1

6 5 6 143.748 10.636 3

-5 6 -6 144.196 12.500 2

6 -5 -6 151.347 9.189 1

6 6 5 129.653 10.443 3

6 -6 -5 151.926 9.400 1

5 6 6 160.598 10.881 3

-6 -6 -6 1735.44 71.82 1

6 6 -6 1498.87 69.72 3

6 -6 6 1520.51 69.85 3

-6 6 6 1459.72 78.23 2

-6 -6 6 1538.98 69.03 1

-6 6 -6 1477.32 74.60 2

6 -6 -6 1470.23 67.53 1

6 6 6 1436.93 68.04 3

0 0 7 1.33820 1.50156 3

0 7 0 1.34403 1.56788 3

7 0 0 -0.5092 2.0201 3

-7 0 0 -0.1885 2.3048 2

0 0 -7 0.48987 2.17948 2

0 -7 0 -0.7235 2.2133 1

7 0 0 1.20783 1.52472 1

0 7 0 -1.4363 1.7217 2

1 0 7 2.95386 1.67047 3

-7 -1 0 -3.9837 1.8301 2

-7 1 0 -3.4910 2.5952 2

-1 0 7 1.47509 1.37694 3

0 7 -1 2.19778 1.63828 3

0 7 1 2.41294 1.58801 3

0 -7 -1 -2.7144 2.1586 1

7 -1 0 3.19256 1.60652 1

7 1 0 3.61270 1.75748 1

1 0 -7 3.11951 2.50529 2

7 -1 0 2.28812 1.98675 3

7 1 0 3.20644 2.34927 3

0 -7 1 -0.8375 2.5569 1

0 7 -1 0.81227 2.06406 2

0 7 1 1.24258 1.85339 2

-1 0 -7 -0.7009 1.9998 2

0 -7 2 56.3124 5.9527 1

2 0 -7 71.9035 4.2088 1

0 -7 -2 78.2332 6.5425 1

7 2 0 72.3176 6.1037 3

7 -2 0 69.1303 5.0171 1

7 2 0 71.1191 5.2926 1

-7 -2 0 63.3627 5.2367 2

-2 0 -7 66.9957 6.4465 2

-2 0 7 70.0102 4.7425 3

0 7 -2 72.0026 4.9797 3

0 7 2 68.3925 5.1810 3

2 0 7 66.1372 5.2261 3

7 -2 0 70.7400 4.9686 3

-7 2 0 76.0441 6.7742 2

2 0 -7 79.6537 6.6243 2

0 7 -2 59.3763 5.9528 2

0 7 2 68.5412 5.7391 2

0 7 -3 57.7957 4.5884 3

0 -7 3 51.6310 5.7251 1

3 0 -7 65.1767 4.1499 1

7 3 0 63.6604 5.7591 3

0 -7 -3 61.2438 5.7429 1

3 0 -7 63.9456 5.9876 2

0 7 3 54.0989 5.3717 2

-3 0 -7 64.6689 6.2054 2

0 7 -3 62.8556 5.9270 2

-3 0 7 58.5352 4.2825 3

-7 -3 0 58.2105 4.5047 2

0 7 3 62.5218 5.0102 3

-7 -3 0 49.5478 5.0965 1

7 -3 0 54.5055 4.6043 1

-7 3 0 57.3539 6.5570 2

3 0 7 62.3838 5.2733 3

0 7 4 32.6441 3.7092 3

4 0 -7 22.9689 3.8612 2

0 7 -4 25.6400 4.2426 2

4 0 -7 25.9729 2.6837 1

0 7 4 19.1856 3.6883 2

-4 0 7 25.3900 2.6312 3

-7 4 0 16.8267 4.3920 2

7 4 0 15.1835 3.3878 3

-4 0 -7 20.2891 3.9898 2

0 -7 -4 17.0468 3.4885 1

4 0 7 20.7979 3.3506 3

0 -7 4 12.7850 3.5102 1

0 7 -4 18.3113 2.7207 3

-7 -4 0 20.1852 3.7873 1

7 -4 0 20.7743 2.9781 1

7 5 0 55.0847 5.6424 3

7 -5 0 51.9261 4.7640 1

-7 5 0 55.2050 6.9977 2

0 7 -5 65.4392 4.9251 3

0 7 5 57.8433 5.2446 3

5 0 -7 52.9099 5.9574 2

0 7 -5 59.0983 6.5555 2

5 0 -7 58.4814 4.6380 1

-7 -5 0 55.1377 5.9570 1

-5 0 -7 57.1173 6.2095 2

0 -7 5 49.0156 5.6089 1

0 -7 -5 63.6647 5.7539 1

5 0 7 48.9352 5.2262 3

0 -7 -5 61.0234 5.4782 2

7 6 0 60.6619 5.8405 3

-7 6 0 48.7958 6.6483 2

6 0 -7 69.7400 6.5514 2

0 -7 6 59.5402 5.7649 1

0 7 6 70.5197 5.9460 3

-7 -6 0 65.8314 6.7097 1

0 7 -6 56.9125 6.7878 2

7 -6 0 56.1430 5.2151 1

6 0 -7 59.2920 4.9317 1

0 -7 -6 67.0005 5.8835 2

0 7 -6 58.1231 5.0317 3

0 -7 -6 52.7741 5.3828 1

6 0 -7 61.2178 4.3502 3

6 0 7 55.2314 5.7663 3

-6 0 -7 57.1800 6.5379 2

-7 0 1 4.93913 2.68486 2

0 1 7 4.52936 1.76173 3

0 1 -7 0.40588 2.13480 2

1 7 0 -0.8551 1.5924 3

0 -1 -7 1.72044 2.19095 2

1 -7 0 1.91240 2.31152 1

-7 0 -1 1.50326 2.15553 2

-1 7 0 -0.9019 2.1176 2

7 0 -1 2.68371 1.68602 3

7 0 -1 0.30475 1.63039 1

7 0 1 2.18999 1.74040 1

-1 7 0 -0.6164 1.3814 3

1 7 0 2.18189 1.87032 2

-1 -7 0 1.93665 2.63135 1

0 -1 7 1.78358 1.49350 3

7 0 1 1.61407 2.29373 3

7 1 -1 723.006 34.611 3

-1 7 1 758.212 37.400 2

7 1 -1 760.844 34.924 1

-1 1 7 765.838 34.206 3

1 -7 1 778.443 36.034 1

1 7 -1 769.265 34.423 3

-7 1 1 754.596 38.008 2

7 -1 1 731.428 35.049 3

-1 7 1 822.560 34.095 3

1 1 -7 730.055 38.134 2

1 7 -1 746.255 37.000 2

1 -1 7 732.940 34.397 3

-7 -1 -1 749.642 37.070 2

-1 -7 -1 768.410 36.322 1

-1 -1 -7 750.191 38.033 2

7 -1 1 735.304 34.644 1

-1 7 -1 730.064 33.679 3

-7 1 -1 730.927 37.555 2

7 1 1 737.601 35.429 3

-1 -1 7 751.287 33.940 3

1 -7 -1 704.118 35.562 1

7 1 1 733.467 34.610 1

-7 -1 1 801.688 36.994 2

1 7 1 861.130 36.658 2

1 7 1 752.379 34.307 3

7 -1 -1 743.966 33.591 3

-1 1 -7 738.475 38.065 2

1 1 7 733.925 34.299 3

7 -1 -1 696.633 34.454 1

-1 -7 1 732.586 36.206 1

-1 7 -1 793.526 37.470 2

1 -1 -7 751.097 37.976 2

-1 -7 -2 316.477 15.918 1

-7 2 1 276.294 16.688 2

7 -2 1 288.521 14.724 3

-2 -1 -7 289.458 16.356 2

2 1 -7 293.851 16.712 2

7 -2 1 275.556 14.184 1

-1 7 2 277.186 15.902 2

1 7 -2 258.498 15.468 2

7 2 -1 286.206 14.672 1

-1 7 2 293.859 14.063 3

1 7 -2 290.818 14.343 3

-2 1 7 294.036 13.960 3

7 2 -1 282.052 14.761 3

1 -7 2 282.443 15.538 1

-7 -2 -1 280.558 15.086 2

2 -1 7 285.272 14.477 3

-2 -1 7 281.238 13.837 3

7 -2 -1 276.063 13.052 3

-1 7 -2 296.606 16.421 2

2 -1 -7 301.107 13.494 1

-1 7 -2 251.337 13.692 3

1 7 2 293.549 15.373 2

2 -1 -7 288.929 16.430 2

7 2 1 281.018 15.644 3

-1 -7 2 270.225 15.789 1

1 7 2 282.065 14.601 3

7 2 1 299.647 14.646 1

-7 2 -1 280.710 16.405 2

1 -7 -2 290.856 15.404 1

-7 -2 1 288.920 14.925 2

2 1 7 272.061 14.519 3

-2 1 -7 256.013 16.406 2

7 -2 -1 304.093 14.429 1

-1 7 3 769.082 35.728 3

7 -3 1 785.359 36.194 1

1 -7 3 805.193 37.335 1

3 -1 7 757.822 36.174 3

3 1 -7 787.567 35.693 1

-7 -3 -1 805.010 37.869 2

-7 3 1 792.412 40.237 2

1 7 -3 786.114 35.910 3

-1 -7 -3 837.701 37.664 1

-7 -3 -1 775.977 37.137 1

-3 1 7 802.968 35.316 3

7 3 -1 777.468 36.705 1

7 3 -1 765.393 36.461 3

3 1 -7 824.988 40.016 2

-3 -1 -7 794.292 39.711 2

-1 7 3 760.053 39.494 2

1 7 -3 764.493 38.956 2

1 -7 -3 769.118 37.017 1

-1 7 -3 766.599 35.212 3

3 -1 -7 811.656 35.418 1

-3 1 -7 770.034 39.733 2

1 7 3 786.886 36.120 3

-1 -7 3 777.205 37.613 1

-1 7 -3 752.963 39.566 2

-7 3 -1 752.545 39.752 2

7 3 1 788.764 37.162 3

3 1 7 801.596 36.156 3

-7 -3 1 793.691 37.246 2

1 7 3 778.552 38.507 2

-3 -1 7 789.622 35.142 3

7 -3 -1 757.119 35.970 1

3 -1 -7 809.620 39.575 2

1 -7 4 -0.8320 2.1969 1

1 7 -4 3.16167 2.58508 2

4 1 -7 1.92927 1.40240 3

-4 -1 -7 4.27948 2.72769 2

-4 1 7 1.30076 1.42125 3

-7 4 1 2.99523 3.30153 2

-1 7 4 5.05062 2.23562 3

7 4 -1 3.42786 2.29479 3

-1 -7 -4 1.75435 2.45661 1

1 7 -4 4.42569 1.96521 3

-7 -4 -1 3.93923 2.75618 1

7 -4 1 4.08817 1.95670 1

4 1 -7 4.58338 2.65293 2

-1 7 4 5.72731 2.97003 2

4 -1 7 4.65964 2.40771 3

-1 -7 -4 2.32804 2.07547 2

4 1 -7 -0.0481 1.4816 1

1 -7 -4 6.50076 2.62411 1

7 -4 -1 2.46505 1.96287 1

-1 -7 4 3.94985 3.18424 1

4 1 7 2.53718 2.25222 3

7 4 1 2.37915 2.60434 3

4 -1 -7 3.86341 1.54952 1

4 -1 -7 5.09380 2.70641 2

-1 7 -4 0.19390 1.68443 3

-7 -4 1 1.82249 2.46922 1

1 7 4 -1.1109 2.0926 3

-7 4 -1 1.71858 3.20436 2

-4 1 -7 4.49158 2.73357 2

-1 7 -4 -2.6093 2.4466 2

-1 -7 -5 945.782 43.408 2

-1 7 5 896.384 40.429 3

5 1 -7 889.993 44.319 2

1 -7 5 858.705 41.282 1

5 -1 7 872.654 40.928 3

5 1 -7 894.081 40.489 1

-7 5 1 908.048 45.371 2

1 7 -5 883.923 40.329 3

-5 -1 -7 865.950 44.469 2

1 7 -5 915.578 44.556 2

-7 -5 -1 850.320 42.193 1

7 5 -1 848.182 40.938 3

5 1 -7 878.352 39.561 3

7 -5 1 874.411 40.803 1

-1 -7 -5 880.087 41.838 1

-5 1 7 893.466 39.457 3

7 -5 -1 847.505 39.951 1

5 1 7 872.981 40.275 3

7 5 1 842.490 40.758 3

-1 7 -5 829.544 43.975 2

1 7 5 940.917 40.263 3

1 -7 -5 852.541 40.548 1

-7 -5 1 860.470 41.373 1

-1 7 -5 854.219 39.094 3

5 -1 -7 880.954 43.695 2

-5 1 -7 870.685 44.083 2

5 -1 -7 885.954 39.543 1

-1 -7 5 863.484 41.297 1

-7 5 -1 803.260 44.025 2

-7 -6 -1 193.772 12.545 1

-6 1 7 201.208 10.018 3

7 -6 1 185.329 10.985 1

1 -7 6 181.119 11.023 1

-1 7 6 204.559 11.344 3

1 7 -6 212.833 13.257 2

6 -1 7 182.570 11.575 3

6 1 -7 194.638 10.103 3

-7 6 1 191.583 13.582 2

6 1 -7 191.285 10.669 1

7 6 -1 197.306 11.631 3

6 1 -7 199.321 12.567 2

1 7 -6 199.182 10.980 3

-6 -1 -7 194.873 12.854 2

-1 -7 -6 178.689 11.468 1

-1 -7 -6 191.237 11.690 2

1 7 6 210.309 11.897 3

6 -1 -7 184.989 12.621 2

-1 7 -6 192.453 10.719 3

-1 -7 6 183.895 12.078 1

6 -1 -7 186.964 10.584 1

1 -7 -6 185.187 11.311 1

6 1 7 177.217 11.715 3

1 -7 -6 204.259 12.107 2

-1 7 -6 181.068 13.500 2

7 -6 -1 202.572 11.214 1

-7 -6 1 198.021 12.595 1

-6 1 -7 201.921 13.399 2

-7 6 -1 195.070 13.559 2

7 6 1 184.669 11.899 3

-2 -7 0 63.4371 6.2188 1

2 -7 0 62.9186 5.7736 1

7 0 -2 69.4762 5.0998 1

7 0 2 65.1446 4.9670 1

0 2 7 77.5359 5.3269 3

-2 7 0 67.3930 4.4135 3

-7 0 -2 64.6320 6.0705 2

7 0 2 70.7088 6.1596 3

-2 7 0 88.6726 6.6853 2

-7 0 2 76.6023 6.6971 2

0 2 -7 69.4261 6.5910 2

0 -2 -7 70.9026 6.3537 2

2 7 0 67.5740 5.2660 3

7 0 -2 73.9726 4.6968 3

0 -2 7 72.3173 5.0919 3

7 -1 2 272.293 14.049 1

1 -2 7 277.099 14.208 3

7 1 -2 290.748 14.189 3

1 2 -7 289.197 16.680 2

2 -7 1 285.464 15.217 1

-7 1 2 273.608 16.457 2

-2 -7 -1 281.532 15.802 1

7 1 -2 298.733 14.538 1

7 -1 2 282.140 15.250 3

2 7 -1 287.385 14.401 3

-2 7 1 296.589 13.527 3

2 7 -1 257.903 14.613 2

-1 2 7 306.061 14.256 3

-1 -2 -7 272.625 16.065 2

-7 -1 -2 272.832 15.541 2

-2 7 1 302.161 16.134 2

7 1 2 303.418 14.718 1

2 7 1 287.094 14.852 3

7 -1 -2 280.605 14.607 1

1 -2 -7 279.349 16.453 2

-7 1 -2 299.288 16.709 2

1 -2 -7 286.768 13.663 1

-1 2 -7 279.136 16.968 2

-2 7 -1 324.853 16.840 2

2 -7 -1 280.164 15.495 1

-7 -1 2 275.591 15.997 2

-2 -7 1 296.432 16.261 1

1 2 7 302.851 14.867 3

-2 7 -1 265.733 13.742 3

7 1 2 291.719 16.031 3

-1 -2 7 298.047 14.423 3

2 7 1 320.686 15.086 2

7 -1 -2 286.830 13.615 3

2 2 -7 42.9945 5.1264 2

2 -2 7 39.2841 3.8606 3

-2 7 2 31.4739 4.4744 2

7 2 -2 37.8718 3.8149 3

7 2 -2 32.8117 3.6111 1

-7 -2 -2 37.0353 3.8840 2

2 2 -7 36.8527 3.2851 3

2 7 -2 41.7433 3.7867 2

7 -2 2 34.8745 4.3303 3

-2 2 7 39.9530 3.4291 3

7 -2 2 33.7631 3.2772 1

-2 7 2 42.4791 3.4499 3

2 -7 2 35.4241 4.1745 1

-7 2 2 39.1998 5.2310 2

-2 -7 -2 38.7887 4.8567 1

2 7 -2 33.4158 3.6673 3

-2 -2 -7 34.0035 4.4703 2

7 2 2 35.9574 3.7337 1

-2 7 -2 36.3735 3.2640 3

-2 2 -7 39.9374 5.1586 2

2 7 2 36.9621 4.1240 3

-2 -7 2 30.0046 4.7598 1

-7 2 -2 34.5784 4.9138 2

-2 7 -2 42.9293 4.9868 2

-2 -2 7 44.4227 3.7992 1

2 2 7 41.9407 4.0713 3

7 -2 -2 36.3358 3.5680 1

7 -2 -2 35.0512 2.4604 3

2 -7 -2 32.9814 4.1292 1

7 2 2 37.2087 4.8953 3

2 -2 -7 45.8415 3.1417 1

2 -2 -7 37.9262 4.6592 2

2 7 2 37.5768 3.6173 2

-7 -2 2 33.4472 3.7541 2

3 2 -7 383.218 20.754 2

-7 -3 -2 374.858 19.138 1

2 -7 3 352.624 18.792 1

3 2 -7 368.884 17.644 3

-7 3 2 364.843 21.198 2

-3 2 7 376.815 17.614 3

7 -3 2 366.978 18.485 3

7 -3 2 369.633 18.013 1

-2 -7 -3 408.485 19.919 1

-2 7 3 359.143 20.653 2

3 -2 7 366.717 18.408 3

7 3 -2 351.202 18.505 1

-3 -2 -7 373.643 20.377 2

7 3 -2 345.874 18.391 3

2 7 -3 375.041 18.286 3

-7 -3 -2 377.955 19.048 2

2 7 -3 379.333 19.375 2

-2 7 3 383.507 17.555 3

2 7 3 382.810 18.916 2

-2 -7 3 334.506 19.293 1

-2 7 -3 345.851 17.062 3

-3 2 -7 352.720 20.584 2

-2 7 -3 355.346 20.434 2

-3 -2 7 379.618 17.805 1

7 3 2 357.389 19.274 3

3 -2 -7 391.059 17.298 1

3 2 7 360.249 18.172 3

-7 3 -2 348.693 20.507 2

2 7 3 365.053 18.484 3

7 -3 -2 361.424 17.840 1

3 -2 -7 356.549 20.017 2

2 -7 -3 339.293 18.442 1

4 2 -7 58.7481 4.3540 3

2 -7 4 51.0449 4.9062 1

-2 7 4 53.0668 6.2836 2

-7 4 2 53.6624 6.4724 2

2 7 -4 63.7447 5.5924 2

-2 -7 -4 55.4210 4.7244 2

7 4 -2 58.5653 5.1628 3

-4 -2 -7 63.2928 6.0199 2

-4 2 7 59.2004 4.2775 3

7 -4 2 58.0283 4.7189 1

4 -2 7 47.7573 4.8949 3

4 2 -7 62.0369 5.9699 2

2 7 -4 56.2379 4.8815 3

-2 7 4 57.4793 4.8618 3

-2 -7 -4 62.4380 5.7575 1

-7 -4 -2 58.1772 5.7959 1

4 2 7 55.0063 5.2512 3

-2 -7 4 60.9325 6.3161 1

2 -7 -4 53.6246 5.1793 1

-7 -4 2 58.1398 5.6106 1

4 -2 -7 51.9017 4.2961 1

7 -4 -2 54.5496 4.8017 1

4 -2 -7 54.2508 5.9753 2

2 7 4 63.1624 5.6414 3

-2 7 -4 48.1324 6.3275 2

-7 4 -2 55.1472 6.4119 2

-4 -2 7 59.1302 4.9321 1

-4 2 -7 62.3136 6.6023 2

-2 7 -4 59.3373 4.4382 3

7 4 2 59.6346 6.2260 3

-5 2 7 155.229 8.097 3

-7 -5 -2 144.168 10.349 1

5 -2 7 146.040 9.544 3

2 7 -5 173.915 10.439 2

2 -7 5 139.108 9.162 1

5 2 -7 150.659 10.350 2

7 -5 2 154.972 8.869 1

-2 7 5 148.963 8.843 3

2 7 -5 135.580 8.759 3

-5 -2 -7 160.768 10.702 2

5 2 -7 147.665 8.758 1

-2 -7 -5 135.706 9.745 1

5 2 -7 147.411 8.314 3

-7 5 2 145.650 11.752 2

-2 -7 -5 168.403 9.480 2

7 5 -2 149.964 9.465 3

-2 7 5 149.732 11.453 2

5 2 7 138.292 9.377 3

2 -7 -5 136.208 8.993 1

7 5 2 123.007 9.768 3

5 -2 -7 159.706 8.455 1

-5 2 -7 151.753 11.200 2

7 -5 -2 143.131 8.639 1

2 7 5 167.459 9.788 3

5 -2 -7 148.458 10.486 2

-7 -5 2 130.777 10.017 1

-2 -7 5 130.046 9.990 1

-2 7 -5 139.537 8.208 3

-2 7 -5 128.420 10.844 2

-7 5 -2 132.266 10.995 2

2 -7 6 133.174 8.376 1

2 -7 6 131.788 8.623 3

-7 6 2 131.839 10.815 2

2 7 -6 125.316 8.322 3

-6 -2 -7 125.605 9.682 2

-2 -7 -6 127.304 9.121 1

-2 -7 -6 133.037 8.800 2

7 6 -2 125.033 8.740 3

-7 -6 -2 124.148 9.825 1

7 -6 2 139.650 8.524 1

6 2 -7 127.908 8.145 1

6 2 -7 130.078 9.442 2

6 -2 7 137.393 9.129 3

-6 2 7 137.882 7.316 3

2 7 -6 147.457 10.136 2

6 2 -7 140.573 7.810 3

-2 -7 6 115.891 9.677 1

2 -7 -6 128.618 8.623 1

-2 7 -6 123.040 10.864 2

2 7 6 144.272 9.542 3

6 -2 -7 134.756 10.021 2

-7 6 -2 131.253 10.735 2

-7 -6 2 119.686 9.631 1

7 -6 -2 128.206 8.354 1

2 -7 -6 119.964 9.263 2

6 2 7 117.347 9.138 3

6 -2 -7 132.070 8.001 1

7 6 2 131.467 9.779 3

-6 2 -7 146.025 11.074 2

7 0 -3 64.1460 4.1563 3

7 0 -3 60.2775 4.7607 1

-3 -7 0 61.5199 5.9679 1

-3 7 0 68.0586 6.2551 2

0 -3 -7 58.4495 3.8819 1

3 7 0 57.9493 5.1192 3

0 3 -7 69.7588 6.5400 2

7 0 3 59.2370 4.6447 1

7 0 3 65.6221 6.0065 3

-7 0 -3 58.8360 5.6772 2

0 3 7 61.2761 5.0230 3

3 -7 0 58.8434 5.3576 1

-7 0 3 53.9484 6.0875 2

0 -3 -7 58.1660 5.6034 2

-3 7 0 54.7407 3.6869 3

0 -3 7 59.4203 4.6778 3

-1 -3 -7 804.128 39.762 2

1 3 -7 787.948 35.901 3

-3 7 1 740.617 34.831 3

1 3 -7 789.414 40.387 2

-3 7 1 757.578 39.696 2

1 -3 7 817.441 36.327 3

7 -1 3 775.996 36.264 1

7 1 -3 765.434 36.669 1

-1 3 7 869.094 36.291 3

-7 1 3 778.297 40.648 2

-7 -1 -3 777.123 39.312 2

3 -7 1 755.366 37.173 1

3 7 -1 810.461 36.563 3

-1 -3 -7 813.488 35.724 1

-3 -7 -1 872.984 38.433 1

7 -1 3 785.015 37.245 3

7 1 -3 829.546 35.829 3

3 7 1 746.059 36.418 3

3 -7 -1 761.315 37.135 1

-7 1 -3 769.349 39.784 2

7 -1 -3 756.005 34.810 3

1 3 7 803.294 36.345 3

-1 -3 7 768.131 36.181 1

7 -1 -3 762.589 36.440 1

-3 7 -1 851.608 40.177 2

7 1 3 856.420 36.511 1

-3 7 -1 743.731 34.916 3

7 1 3 782.333 37.644 3

-3 -7 1 807.913 38.021 1

1 -3 -7 832.981 39.794 2

-7 -1 3 847.570 39.851 2

-1 3 -7 774.175 40.446 2

1 -3 -7 816.170 35.703 1

2 3 -7 369.198 17.974 3

3 -7 2 355.237 18.870 1

-2 -3 -7 356.215 17.507 1

-2 3 7 376.884 18.045 3

7 -2 3 392.181 19.230 3

-7 -2 -3 379.641 19.787 2

-2 -3 -7 362.403 20.262 2

-7 2 3 400.670 21.952 2

3 7 -2 389.685 18.637 3

-3 7 2 351.994 20.692 2

7 2 -3 370.167 18.603 1

-7 -2 -3 359.026 18.834 1

7 -2 3 376.427 18.072 1

7 2 -3 397.654 18.351 3

2 -3 7 356.494 18.283 3

-3 -7 -2 412.713 20.147 1

2 3 -7 395.175 21.066 2

-7 -2 3 417.088 20.079 2

-2 -3 7 415.828 18.696 1

-7 2 -3 366.626 20.780 2

-3 7 -2 341.781 17.112 3

-3 7 -2 378.862 20.798 2

3 7 2 371.999 18.935 3

3 -7 -2 360.245 18.828 1

-3 -7 2 385.259 19.905 1

7 2 3 348.384 19.536 3

2 -3 -7 373.889 17.568 1

-2 3 -7 342.861 20.981 2

2 -3 -7 352.397 20.219 2

2 3 7 374.236 18.579 3

7 -2 -3 362.066 18.225 1

3 -7 3 364.992 18.749 1

-7 -3 -3 353.319 19.016 1

-3 3 7 410.067 17.910 3

3 3 -7 399.865 20.967 2

-3 -7 -3 395.531 19.820 1

7 3 -3 354.264 18.588 1

-7 -3 -3 354.053 19.220 2

7 -3 3 349.092 17.857 1

-7 3 3 361.553 21.667 2

3 -3 7 363.929 18.442 3

3 3 -7 360.141 17.794 3

3 7 -3 369.767 18.427 3

-3 -3 -7 367.913 20.206 2

-3 7 3 363.302 21.075 2

7 -3 3 410.654 18.865 3

7 3 -3 367.364 18.438 3

-3 7 -3 389.511 21.678 2

-3 3 -7 359.629 21.651 2

7 3 3 365.518 20.112 3

3 7 3 378.896 19.216 2

3 -3 -7 385.111 20.884 2

7 -3 -3 362.361 18.563 1

-3 7 -3 357.207 17.638 3

3 -3 -7 382.747 18.101 1

-3 -7 3 373.342 20.117 1

3 3 7 393.778 19.217 3

-3 -3 7 407.020 19.139 1

3 -7 -3 351.800 19.057 1

3 7 3 383.517 19.501 3

-7 3 -3 393.435 21.748 2

4 3 -7 35.3719 3.3872 3

-4 3 7 35.9967 3.2846 3

4 3 -7 30.5111 4.4988 2

3 -7 4 32.0439 3.4323 3

-7 4 3 46.7940 6.2598 2

-3 7 4 31.1661 5.3005 2

3 -7 4 33.2485 3.8879 1

-7 -4 -3 29.1701 4.4224 1

4 -3 7 28.8271 3.8868 3

-3 -7 -4 30.5154 4.6491 1

7 -4 3 32.0133 3.8351 3

7 -4 3 28.4529 3.2693 1

3 7 -4 27.7606 3.6581 3

-3 -7 -4 35.0491 3.3508 2

7 4 -3 29.5620 3.8202 3

-4 -3 -7 24.6377 4.1318 2

-3 7 -4 28.0239 5.2194 2

-7 4 -3 29.2432 5.3472 2

3 7 4 31.6912 4.4082 3

-4 3 -7 33.7796 5.5487 2

3 -7 -4 30.0294 3.9981 1

-4 -3 7 38.3421 4.2454 1

-7 -4 3 29.6547 4.3354 1

-3 -7 4 32.6827 4.8639 1

4 3 7 32.6337 4.3684 3

7 -4 -3 31.5037 3.5721 1

4 -3 -7 23.6207 4.2365 2

4 -3 -7 36.2866 3.3719 1

7 4 3 30.8413 5.0225 3

-5 3 7 510.687 23.334 3

-3 7 5 483.048 28.060 2

3 -7 5 511.657 24.108 3

-5 -3 -7 523.112 26.762 2

-3 -7 -5 537.728 25.634 1

-3 -7 -5 530.101 25.506 2

5 3 -7 499.983 23.781 3

3 -7 5 519.719 24.364 1

-7 -5 -3 463.245 25.585 1

3 7 -5 478.548 24.302 3

7 5 -3 495.864 24.651 3

7 -5 3 516.228 24.257 1

-7 5 3 474.220 28.178 2

5 -3 7 496.589 24.776 3

5 3 -7 519.021 26.610 2

5 -3 -7 514.249 23.423 1

3 -7 -5 495.908 24.086 1

5 3 7 458.470 24.351 3

-3 -7 5 430.075 24.744 1

3 7 5 493.069 24.569 3

-7 -5 3 480.311 25.027 1

-3 7 -5 492.951 27.549 2

-5 -3 7 514.153 24.196 1

7 5 3 506.173 25.465 3

-7 5 -3 496.174 27.515 2

-5 3 -7 497.588 27.484 2

7 -5 -3 451.090 23.510 1

5 -3 -7 478.131 26.407 2

7 -6 3 273.618 14.398 1

6 3 -7 273.695 15.633 2

-6 -3 -7 271.656 16.119 2

3 7 -6 276.544 14.541 3

7 6 -3 249.978 14.658 3

6 -3 7 243.708 14.746 3

-3 7 6 262.394 17.717 2

-3 -7 -6 281.581 15.263 1

6 3 -7 253.439 13.791 3

-7 -6 -3 249.777 15.680 1

3 -7 6 316.967 14.450 1

3 -7 6 271.585 14.555 3

-3 -7 -6 290.675 15.136 2

-7 6 3 262.207 17.538 2

-7 6 -3 261.018 17.209 2

-7 -6 3 259.514 15.490 1

-6 3 -7 254.082 16.847 2

-3 -7 6 222.714 15.041 1

7 -6 -3 247.416 13.889 1

-3 7 -6 273.955 17.537 2

3 -7 -6 263.419 14.232 1

7 6 3 245.552 15.317 3

-6 -3 7 267.200 14.442 1

6 3 7 254.981 15.221 3

3 7 6 275.841 15.160 3

6 -3 -7 248.629 13.575 1

6 -3 -7 253.852 16.190 2

0 -4 7 22.5890 3.0536 3

-4 -7 0 20.5520 4.0177 1

7 0 -4 21.6909 2.2830 3

-7 0 4 28.4705 4.8941 2

-4 7 0 21.6774 4.2027 2

7 0 -4 22.9782 2.9391 1

0 4 7 18.7029 3.1147 3

4 7 0 17.1782 3.1070 3

4 -7 0 19.1449 3.3541 1

7 0 4 23.3488 2.8242 1

0 4 -7 21.3566 4.4434 2

-7 0 -4 17.3304 3.8516 2

0 -4 -7 25.3435 3.7538 2

0 -4 -7 16.6597 2.4431 1

7 0 4 26.1297 4.1009 3

7 -1 4 5.92876 1.82440 1

-1 -4 -7 4.14078 2.35638 2

-4 -7 -1 -1.0656 2.6345 1

-1 -4 -7 0.62589 1.48927 1

-7 1 4 -5.9086 2.9479 2

-7 -1 -4 8.08030 2.91474 2

7 1 -4 0.65226 1.76793 1

4 7 -1 2.00934 1.95140 3

-1 4 7 3.23263 2.06697 3

7 1 -4 1.82164 1.48657 3

1 4 -7 1.56041 1.70874 3

1 -4 7 2.89130 1.94256 3

1 4 -7 3.89481 2.65705 2

4 -7 1 0.66356 2.17342 1

7 -1 4 7.72896 2.94289 3

-4 7 1 1.69450 2.60506 2

1 -4 -7 1.89052 1.49097 1

-1 4 -7 3.93085 3.09364 2

7 -1 -4 2.40583 1.85295 1

1 4 7 1.75828 2.09748 3

-4 -7 1 6.30001 3.12253 1

7 -1 -4 2.87636 1.21145 3

-7 -1 4 1.13994 3.17034 2

-1 -4 7 0.92914 2.41581 1

-7 1 -4 9.19438 3.22014 2

4 7 1 1.47218 2.09854 3

4 -7 -1 2.42094 2.18756 1

-4 7 -1 5.98528 3.29963 2

1 -4 -7 2.12700 2.33817 2

7 1 4 2.74966 1.91457 1

7 1 4 2.34363 2.82938 3

2 4 -7 58.3871 4.6732 3

7 -2 4 58.2629 4.3548 1

7 2 -4 52.1968 4.8101 1

7 -2 4 57.6962 5.3504 3

4 7 -2 61.7197 5.1703 3

-4 -7 -2 74.8201 6.3155 1

4 -7 2 55.2096 4.9902 1

2 4 -7 53.0053 6.0596 2

4 -7 2 51.2732 4.0045 3

-2 4 7 58.6200 4.6836 3

-7 2 4 63.9942 7.2933 2

-7 -2 -4 58.8712 5.4837 2

-4 7 2 57.0380 6.1209 2

-2 -4 -7 47.4532 4.2100 1

2 -4 7 56.3163 5.0358 3

-2 -4 -7 60.9962 5.7494 2

7 2 -4 56.4775 4.5948 3

-7 -2 -4 57.5983 5.1748 1

2 -4 -7 61.1317 4.4057 1

2 -4 -7 54.4317 5.5723 2

2 4 7 65.0811 5.6024 3

-4 7 -2 51.5926 6.1539 2

-4 -7 2 43.4835 5.5648 1

7 2 4 58.6886 6.2197 3

7 -2 -4 56.4164 4.7943 1

-7 -2 4 61.8369 6.2316 2

4 -7 -2 54.4775 5.0866 1

4 7 2 52.2161 5.4273 3

-2 4 -7 59.9476 6.7784 2

-7 2 -4 60.0904 6.5561 2

-2 -4 7 69.0229 5.8273 1

7 3 -4 39.2172 3.9769 1

4 -7 3 34.4302 3.2206 3

7 3 -4 37.7159 3.8047 3

-4 -7 -3 30.5964 4.7535 1

3 4 -7 27.1591 3.2652 3

3 -4 7 36.3882 3.9596 3

-7 -3 -4 26.6881 3.8983 2

4 7 -3 29.8944 3.7302 3

-3 -4 -7 23.0768 3.2901 1

-7 3 4 28.3233 5.8978 2

3 4 -7 22.9516 4.3153 2

-7 -3 -4 42.6106 4.6860 1

-4 7 3 30.8005 5.2777 2

-3 4 7 31.0141 3.3492 3

7 -3 4 31.0772 3.1323 1

-3 -4 -7 32.3876 4.2401 2

4 -7 3 30.9164 3.8466 1

7 -3 4 37.0212 4.1961 3

4 -7 -3 32.4710 3.9968 1

-3 -4 7 38.2384 4.5389 1

-4 7 -3 31.6754 5.3373 2

-4 -7 3 31.2399 4.6840 1

3 4 7 20.1783 3.8577 3

7 -3 -4 32.9649 3.6053 1

-7 -3 4 37.4296 4.7004 2

-7 3 -4 28.3982 5.3387 2

3 -4 -7 33.2145 4.6587 2

3 -4 -7 31.9404 3.2975 1

4 7 3 29.3050 4.3506 3

7 3 4 33.3826 4.9779 3

-3 4 -7 21.2508 5.1263 2

-7 -4 -4 42.1997 5.1601 1

4 4 -7 36.5081 3.9077 3

7 -4 4 46.2761 4.7229 3

4 7 -4 42.6524 4.4234 3

7 -4 4 40.5920 3.7849 1

4 -4 7 50.2727 4.7829 3

-4 -4 -7 36.3088 4.7607 2

4 -7 4 51.0227 4.5255 1

-4 7 4 35.9889 6.1238 2

4 4 -7 45.1813 5.1822 2

-7 4 4 39.4180 6.8502 2

-4 4 7 49.1613 4.1514 3

-4 -7 -4 49.6434 5.5731 1

4 -7 4 44.8623 4.0544 3

7 4 -4 42.5974 4.4371 3

4 -7 -4 45.9673 4.7186 1

-4 -4 7 41.2042 5.0630 1

4 4 7 34.4950 4.9227 3

4 -4 -7 46.8274 4.0499 1

-7 -4 4 43.7768 5.1520 1

4 -4 -7 33.3606 5.0490 2

4 7 4 47.7490 5.3880 3

-7 4 -4 42.5546 6.3571 2

-4 7 -4 37.1985 6.0531 2

7 4 4 39.8944 5.6077 3

-4 -7 4 37.3600 5.1663 1

-4 4 -7 58.0409 7.0507 2

7 -4 -4 40.4936 4.2380 1

4 -7 5 57.9305 5.2073 1

7 5 -4 48.8337 5.0470 3

5 4 -7 52.7591 4.6632 3

4 -7 5 53.4844 4.8312 3

7 -5 4 53.9378 4.8406 1

-4 -7 -5 57.4691 5.9839 1

5 4 -7 64.6124 5.8658 2

-7 5 4 49.7518 7.3106 2

-5 -4 -7 61.1252 5.9215 2

-7 -5 -4 55.6848 6.0219 1

5 -4 7 49.6957 5.3504 3

4 7 -5 54.3556 5.0116 3

-4 7 5 56.2022 7.3205 2

5 4 7 48.1164 5.7158 3

4 7 5 55.6198 6.0342 3

7 -5 -4 49.2275 4.7882 1

-5 -4 7 60.4371 5.7949 1

-4 -7 5 48.5341 5.9573 1

7 5 4 41.9405 5.9710 3

5 -4 -7 51.4295 6.0555 2

-7 5 -4 46.3875 6.8048 2

4 -7 -5 43.8197 4.8053 1

-4 7 -5 44.0499 6.7739 2

-7 -5 4 57.6807 6.1399 1

-5 4 -7 52.2846 7.0368 2

5 -4 -7 55.0964 4.8911 1

-7 -6 -4 64.2824 6.4890 1

7 -6 4 63.6207 5.2805 1

-6 -4 -7 57.9630 6.1035 2

7 6 -4 45.6084 5.2314 3

4 -7 6 66.0479 5.6170 3

6 -4 7 57.5047 6.0108 3

-7 6 4 48.5663 7.5584 2

4 7 -6 62.1663 5.4900 3

-4 -7 -6 60.4366 6.1964 1

6 4 -7 61.5759 5.2063 3

-6 4 7 61.5489 8.7098 2

-4 7 6 58.1256 7.9551 2

-4 7 -6 68.5895 8.1295 2

7 -6 -4 49.1456 5.1482 1

-7 -6 4 54.6279 6.5466 1

-6 -4 7 62.8325 6.1369 1

-6 4 -7 55.6822 7.4381 2

4 -7 -6 58.8056 5.3946 1

6 -4 -7 58.2804 5.2168 1

4 7 6 59.0637 6.4136 3

-4 -7 6 54.3657 6.3678 1

6 4 7 45.9085 6.0985 3

-7 6 -4 51.4217 7.4001 2

7 6 4 47.6230 6.3383 3

5 -7 0 54.6698 5.1108 1

0 -5 -7 58.1700 5.4648 2

-5 7 0 51.6610 6.5075 2

0 -5 -7 56.6338 4.7003 1

0 5 -7 54.0415 4.7829 3

0 5 7 61.2155 5.3457 3

5 7 0 59.3069 5.3329 3

-7 0 -5 56.8578 6.0845 2

7 0 5 58.5538 5.9773 3

0 5 -7 53.9627 6.6270 2

-7 0 5 68.4060 7.2502 2

7 0 -5 55.3602 3.8447 3

7 0 -5 57.0077 4.8622 1

7 0 5 53.5085 4.6739 1

-5 -7 0 51.4818 5.7942 1

5 -7 1 901.499 41.391 1

-7 1 5 923.945 46.171 2

1 -5 7 876.587 40.619 3

-7 -1 -5 865.366 44.356 2

7 -1 5 935.196 41.552 3

-1 -5 -7 887.575 40.770 1

-1 5 7 850.167 40.432 3

1 5 -7 901.109 45.210 2

-7 -1 -5 903.947 40.938 1

1 5 -7 890.480 40.279 3

-5 -7 -1 872.042 42.308 1

5 7 -1 880.449 40.911 3

-5 7 1 877.822 44.928 2

7 1 -5 870.432 39.587 3

-1 -5 -7 878.410 43.778 2

7 1 -5 835.721 40.838 1

7 -1 5 946.661 40.385 1

-5 -7 1 878.463 41.732 1

5 7 1 869.336 40.545 3

-1 5 -7 867.832 44.814 2

-1 -5 7 806.336 40.394 1

1 -5 -7 943.069 40.117 1

-5 7 -1 875.827 44.345 2

-7 -1 5 865.458 44.349 2

7 -1 -5 873.659 40.223 1

7 1 5 860.449 41.178 3

1 5 7 869.139 40.339 3

7 -1 -5 870.230 38.388 3

-7 1 -5 871.027 44.154 2

5 -7 -1 867.021 40.720 1

1 -5 -7 868.618 43.328 2

-2 5 7 151.499 8.929 3

2 -5 7 156.653 9.314 3

-2 -5 -7 140.823 8.902 1

-7 -2 -5 149.558 10.258 2

5 7 -2 152.974 9.345 3

7 2 -5 142.173 8.979 1

-7 -2 -5 150.291 9.380 1

-7 2 5 150.492 12.434 2

2 5 -7 149.883 8.842 3

7 2 -5 152.897 8.610 3

-5 7 2 166.218 11.395 2

5 -7 2 143.078 9.357 1

2 5 -7 146.972 10.882 2

-2 -5 -7 150.616 9.822 2

-5 -7 -2 166.669 10.668 1

7 -2 5 164.955 9.934 3

7 -2 5 146.499 8.288 1

-5 -7 2 155.686 10.480 1

-2 -5 7 147.275 9.873 1

7 2 5 166.683 10.705 3

-2 5 -7 136.232 11.360 2

-5 7 -2 149.718 11.201 2

2 -5 -7 139.896 10.067 2

-7 2 -5 144.382 11.108 2

2 -5 -7 154.213 8.630 1

5 -7 -2 135.433 9.205 1

2 5 7 142.081 9.604 3

7 -2 -5 149.718 8.885 1

5 7 2 149.566 9.962 3

-7 -2 5 151.469 11.072 2

-3 5 7 469.614 23.728 3

-5 7 3 498.716 28.088 2

-7 3 5 490.701 29.131 2

-3 -5 -7 519.448 26.298 2

3 5 -7 499.738 24.197 3

-3 -5 -7 514.356 24.716 1

-7 -3 -5 537.546 26.539 2

3 5 -7 557.120 27.344 2

5 -7 3 508.196 24.604 1

7 3 -5 472.390 24.607 1

3 -5 7 508.692 24.563 3

7 -3 5 491.666 23.688 1

7 3 -5 508.393 24.062 3

-7 -3 -5 510.778 25.227 1

5 7 -3 483.176 24.588 3

7 -3 5 520.127 24.871 3

-5 -7 -3 546.171 26.088 1

-3 5 -7 517.155 28.571 2

3 5 7 468.212 24.969 3

7 3 5 480.382 25.698 3

-7 3 -5 506.757 27.910 2

5 7 3 547.573 25.740 3

-5 -7 3 485.453 25.634 1

3 -5 -7 487.000 26.782 2

-7 -3 5 537.926 26.712 2

-5 7 -3 508.114 28.072 2

5 -7 -3 465.496 24.531 1

-3 -5 7 488.856 25.142 1

7 -3 -5 505.069 24.308 1

3 -5 -7 516.556 24.105 1

-5 7 4 47.7136 6.9866 2

-7 -4 -5 42.0663 5.4089 1

4 -5 7 60.8569 5.4776 3

7 -4 5 57.9417 5.4307 3

-5 -7 -4 43.1937 5.6722 1

4 5 -7 58.8766 5.7986 2

5 -7 4 60.4189 5.0914 1

7 4 -5 53.0287 4.9424 3

4 5 -7 49.2980 4.7047 3

-4 -5 -7 61.3376 5.1867 1

5 7 -4 57.9411 5.1812 3

-7 -4 -5 53.9816 5.2152 2

-7 4 5 44.9455 7.5688 2

-4 -5 -7 46.5603 5.0693 2

7 -4 5 48.9455 4.4478 1

5 -7 4 53.7440 4.7080 3

4 -5 -7 44.5043 4.4197 1

4 -5 -7 45.4479 5.6142 2

-4 5 -7 46.4205 6.9284 2

-5 7 -4 37.8034 6.2988 2

-7 -4 5 56.6354 5.8325 1

5 -7 -4 42.9547 4.7061 1

7 4 5 46.4038 5.9573 3

4 5 7 49.7663 5.9012 3

7 -4 -5 49.3995 4.6953 1

-5 -7 4 55.1348 6.1653 1

5 7 4 64.2254 6.3551 3

-4 -5 7 51.9543 5.7119 1

-7 4 -5 55.7954 7.1885 2

5 -7 5 542.727 25.783 1

-5 7 5 497.637 30.454 2

5 -7 5 549.672 25.862 3

7 -5 5 534.044 25.454 1

5 5 -7 543.023 25.820 3

-7 5 5 505.446 31.028 2

-7 -5 -5 537.030 27.094 1

-5 -7 -5 555.356 27.342 1

7 5 -5 555.458 26.199 3

5 -5 7 540.614 26.427 3

5 7 -5 495.388 25.996 3

-5 -5 -7 572.520 28.037 2

7 5 5 482.616 26.716 3

5 -7 -5 515.229 25.659 1

-7 5 -5 544.352 29.961 2

-5 -7 5 510.168 26.772 1

5 5 7 492.533 26.410 3

-7 -5 5 516.437 26.759 1

7 -5 -5 520.740 25.464 1

-5 5 -7 513.293 29.747 2

5 7 5 569.899 27.024 3

5 -5 -7 508.127 25.197 1

-5 7 -5 518.206 29.864 2

-5 -5 7 549.453 26.700 1

-7 -6 -5 90.3537 8.0751 1

7 6 -5 86.8061 7.2009 3

7 -6 5 103.306 6.820 1

-7 6 5 90.8851 10.3066 2

6 5 -7 99.8387 7.1071 3

-6 -5 -7 99.0944 7.9117 2

-6 5 7 86.1824 10.3545 2

-5 7 6 90.2709 10.1197 2

6 -5 7 84.0827 7.6353 3

5 7 -6 91.3711 7.2044 3

-5 -7 -6 107.320 8.450 1

5 -7 6 91.5320 7.6433 3

-6 -5 7 81.8567 7.9043 1

-5 -7 6 96.7550 8.6480 1

-6 5 -7 108.018 10.160 2

-5 7 -6 87.7208 9.6488 2

-7 6 -5 101.938 10.086 2

6 -5 -7 84.5934 6.8859 1

7 -6 -5 87.5994 7.0643 1

-7 -6 5 95.4659 8.5043 1

5 7 6 97.9475 8.5702 3

5 -7 -6 89.5434 7.2194 1

7 6 5 86.3273 8.3198 3

6 5 7 87.0104 8.3285 3

-7 0 6 59.0988 7.4414 2

7 0 -6 60.8803 5.1466 1

-6 -7 0 58.8576 6.5120 1

-7 0 -6 61.2265 6.6172 2

7 0 6 66.0317 6.3489 3

-6 7 0 61.7462 7.1059 2

0 6 7 63.1067 5.8416 3

6 7 0 58.5924 5.5426 3

0 6 -7 63.7705 7.1617 2

7 0 -6 59.4449 4.2352 3

6 -7 0 58.2660 5.3969 1

0 6 -7 64.9596 5.2936 3

0 -6 -7 70.1565 6.1208 2

0 -6 -7 63.5991 5.4644 1

-6 -7 -1 195.979 12.625 1

-1 -6 -7 177.298 11.109 1

-1 6 7 188.326 11.306 3

6 7 -1 179.134 11.379 3

7 -1 6 186.998 11.935 3

1 6 -7 223.031 13.832 2

-7 -1 -6 209.805 11.186 1

1 -6 7 193.338 11.328 3

-7 1 6 198.761 14.529 2

7 1 -6 199.920 12.382 2

1 6 -7 208.401 11.142 3

-6 7 1 200.454 13.591 2

-1 -6 -7 209.002 12.098 2

6 -7 1 199.450 11.428 1

7 1 -6 182.233 10.884 1

-7 -1 -6 215.110 13.119 2

7 1 -6 194.014 10.188 3

7 1 6 199.402 12.230 3

1 -6 -7 189.767 10.820 1

1 -6 -7 193.081 12.011 2

7 -1 -6 189.028 9.409 3

-1 -6 7 180.900 11.500 1

1 6 7 200.607 11.633 3

7 -1 -6 184.565 10.574 1

-1 6 -7 186.241 13.585 2

6 -7 -1 198.492 11.241 1

-6 -7 1 170.684 12.042 1

-6 7 -1 166.897 12.874 2

6 7 1 192.528 11.595 3

-7 1 -6 186.506 12.884 2

-7 2 6 130.568 12.080 2

7 2 -6 146.804 9.440 2

-2 -6 -7 139.558 8.859 1

6 7 -2 138.587 8.923 3

-6 7 2 132.574 10.767 2

7 2 -6 137.720 7.964 3

2 6 -7 130.592 8.376 3

-6 -7 -2 137.594 9.974 1

2 -6 7 135.036 8.840 3

-2 6 7 137.309 8.664 3

-2 -6 -7 139.292 9.194 2

7 2 -6 122.042 8.333 1

-7 -2 -6 134.655 8.916 1

7 -2 6 141.965 9.380 3

6 -7 2 140.419 8.820 1

2 6 -7 132.585 10.149 2

-7 -2 -6 128.733 9.831 2

7 -2 -6 135.914 8.252 1

6 7 2 144.202 9.675 3

2 6 7 127.319 9.279 3

2 -6 -7 134.432 8.324 1

-7 2 -6 120.835 10.351 2

-6 -7 2 132.721 10.079 1

-2 6 -7 130.858 11.179 2

-6 7 -2 124.615 10.600 2

6 -7 -2 123.636 8.556 1

7 2 6 128.626 9.783 3

2 -6 -7 135.548 9.543 2

-2 -6 7 124.338 9.544 1

3 -6 7 264.346 14.571 3

-7 -3 -6 256.822 14.752 1

7 -3 6 275.623 15.009 3

-6 -7 -3 275.984 15.879 1

-6 7 3 237.237 16.940 2

-3 -6 -7 274.049 15.276 2

-3 -6 -7 286.445 15.000 1

6 -7 3 276.277 14.579 1

3 6 -7 304.239 16.489 2

7 3 -6 270.609 15.109 2

-7 3 6 267.549 18.830 2

6 7 -3 248.361 14.518 3

7 3 -6 266.669 13.832 3

3 6 -7 277.547 14.392 3

-7 -3 -6 257.529 15.735 2

-7 -3 6 259.020 14.800 1

6 -7 -3 238.004 14.246 1

7 3 6 252.061 15.574 3

7 -3 -6 270.296 14.102 1

-7 3 -6 291.218 17.613 2

3 6 7 270.856 15.307 3

-6 -7 3 268.711 15.814 1

-3 -6 7 242.705 15.170 1

-3 6 -7 262.945 17.613 2

-6 7 -3 244.369 17.063 2

6 7 3 279.485 15.632 3

3 -6 -7 260.618 15.793 2

3 -6 -7 271.758 14.081 1

-4 -6 -7 58.6823 5.7238 1

4 6 -7 63.4797 5.4444 3

4 -6 7 58.7568 5.8120 3

6 -7 4 64.1192 5.4370 1

7 4 -6 54.8794 5.1241 3

-6 7 4 47.4416 7.5116 2

7 -4 6 58.5649 5.9318 3

-7 -4 -6 57.5561 5.9012 2

-7 -4 -6 64.8230 6.0616 1

-7 4 6 73.0131 9.1564 2

-6 -7 -4 66.0364 6.6771 1

6 7 -4 59.7097 5.6053 3

-4 -6 -7 64.4851 5.8352 2

7 -4 -6 59.5223 5.2387 1

-6 7 -4 64.4395 7.8509 2

4 6 7 58.9198 6.5110 3

-7 4 -6 52.1331 7.3653 2

-6 -7 4 58.0873 6.6215 1

4 -6 -7 52.9610 5.1452 1

6 -7 -4 63.7203 5.6709 1

4 -6 -7 59.6524 6.4356 2

-4 -6 7 53.2940 6.2416 1

7 4 6 57.2987 6.7124 3

6 7 4 63.4028 6.7391 3

-7 -4 6 53.3742 5.9781 1

-4 6 -7 47.6083 7.3185 2

6 7 -5 97.9186 7.4526 3

5 6 -7 96.2969 7.2447 3

7 5 -6 99.5869 7.2732 3

-6 -7 -5 94.5505 8.2619 1

5 -6 7 98.9125 7.7922 3

-6 7 5 70.6862 9.4295 2

-5 -6 -7 96.5065 7.4313 2

-7 5 6 100.096 11.062 2

-5 -6 -7 103.187 8.219 1

-7 -5 -6 102.446 8.339 1

7 -5 6 103.572 7.994 3

6 -7 5 91.2133 6.9641 1

-5 6 7 86.7105 10.4293 2

6 -7 -5 82.8440 7.2196 1

-5 -6 7 96.8717 8.6580 1

5 6 7 106.771 8.755 3

5 -6 -7 83.7111 6.8982 1

6 7 5 99.5455 8.7098 3

-7 -5 6 91.6290 8.3860 1

-7 5 -6 83.7235 9.4677 2

-5 6 -7 112.081 10.451 2

-6 7 -5 110.640 10.384 2

7 -5 -6 86.8928 6.9519 1

7 5 6 86.6952 8.3156 3

-6 -7 5 85.2069 8.1685 1

6 7 -6 202.355 12.539 3

-6 -7 -6 245.740 13.811 1

-7 -6 -6 240.185 13.784 1

7 6 -6 236.976 12.887 3

6 6 -7 195.588 12.295 3

-7 6 6 228.843 17.427 2

-6 7 6 193.208 16.381 2

-6 6 7 208.601 16.988 2

6 -6 7 196.634 13.043 3

7 6 6 199.302 13.984 3

-6 -7 6 206.808 13.987 1

6 -6 -7 218.524 12.657 1

6 6 7 222.996 14.247 3

6 -7 -6 211.026 12.885 1

-6 7 -6 218.530 16.403 2

-6 -6 7 213.751 13.981 1

-7 -6 6 215.801 14.311 1

7 -6 -6 210.954 12.802 1

-6 6 -7 242.108 16.758 2

-7 6 -6 198.179 15.975 2

6 7 6 257.151 14.726 3

7 7 0 86.6801 7.2727 3

7 0 -7 87.0503 6.4750 1

0 7 -7 89.4677 8.7353 2

7 0 -7 101.792 8.317 2

-7 0 -7 96.6450 8.6207 2

7 0 7 79.8473 7.4785 3

7 0 -7 89.1716 5.5536 3

0 7 -7 89.0697 6.5968 3

0 7 7 95.8681 7.4817 3

-7 -7 0 96.1042 8.5249 1

0 -7 -7 84.5899 6.9967 1

7 -7 0 88.5746 6.9132 1

0 -7 -7 83.7111 7.2408 2

-7 7 0 86.4937 8.7840 2

-1 -7 -7 273.057 15.120 2

-1 7 7 264.381 14.403 3

1 7 -7 258.489 13.962 3

-7 -1 -7 271.385 16.284 2

7 1 -7 236.999 13.697 1

-7 -7 -1 245.053 15.646 1

-7 7 1 254.798 16.885 2

7 -7 1 258.200 14.387 1

7 1 -7 267.330 15.662 2

7 1 -7 260.862 13.044 3

7 7 -1 274.748 14.730 3

7 -1 7 264.431 15.077 3

1 7 -7 287.469 16.851 2

-1 -7 -7 242.410 14.425 1

1 7 7 269.371 14.825 3

-7 7 -1 257.981 16.757 2

-1 -7 7 229.133 14.600 1

-1 7 -7 252.701 13.656 3

-1 7 -7 264.388 17.314 2

1 -7 -7 256.516 14.276 1

7 -7 -1 281.637 14.595 1

-7 -7 1 232.415 15.332 1

7 -1 -7 257.767 13.763 1

7 -1 -7 264.540 16.010 2

7 7 1 255.001 14.824 3

7 1 7 265.612 15.400 3

1 -7 -7 265.177 15.373 2

-7 1 -7 255.218 16.664 2

2 7 -7 370.437 20.785 2

7 2 -7 344.660 18.264 1

2 -7 7 382.133 18.875 3

7 2 -7 343.859 19.824 2

7 -2 7 364.794 19.224 3

-2 -7 -7 390.641 19.754 2

-7 -2 -7 379.874 20.845 2

-7 7 2 354.535 21.624 2

7 7 -2 340.462 18.683 3

2 7 -7 355.885 18.365 3

-2 -7 -7 362.268 19.027 1

7 -7 2 368.627 18.765 1

7 2 -7 337.191 17.459 3

-7 2 7 375.714 23.249 2

-7 -7 -2 355.073 20.053 1

7 7 2 341.095 18.883 3

2 -7 -7 348.840 19.499 2

7 2 7 356.680 19.299 3

2 7 7 341.541 18.519 3

-7 2 -7 362.287 21.165 2

-7 -7 2 329.491 19.296 1

-2 7 -7 356.601 21.608 2

-7 7 -2 337.834 20.785 2

2 -7 -7 346.095 18.023 1

7 -7 -2 347.622 18.124 1

7 -2 -7 324.472 17.412 1

-2 -7 7 321.052 18.963 1

-7 3 7 227.646 18.651 2

-3 -7 -7 299.247 15.475 1

-7 7 3 249.582 17.442 2

3 7 -7 275.547 14.626 3

3 -7 7 277.073 14.983 3

7 -7 3 275.377 14.741 1

-7 -3 -7 299.869 16.489 2

-3 -7 -7 279.772 15.223 2

-7 -7 -3 248.864 15.682 1

7 -3 7 285.425 15.489 3

-3 7 7 255.313 17.793 2

7 3 -7 268.839 15.473 2

7 7 -3 258.310 14.794 3

7 3 -7 243.794 13.745 3

7 -7 -3 254.194 14.552 1

-7 7 -3 270.204 17.752 2

-7 3 -7 259.984 17.404 2

7 3 7 272.888 15.971 3

-3 7 -7 246.885 17.606 2

7 7 3 280.202 15.937 3

-3 -7 7 255.186 15.728 1

-7 -3 7 254.998 14.933 1

3 7 7 271.879 15.420 3

-7 -7 3 243.423 15.691 1

7 -3 -7 244.246 13.940 1

3 -7 -7 265.029 14.464 1

7 7 -4 102.685 7.825 3

-4 -7 -7 121.976 8.514 1

4 -7 7 98.9718 7.9316 3

-7 -7 -4 101.287 8.930 1

-7 -4 -7 112.659 8.730 2

7 -7 4 111.817 7.626 1

-7 4 7 92.9849 11.2367 2

-4 7 7 97.7475 10.5779 2

-7 7 4 114.829 10.825 2

7 4 -7 99.2584 7.1734 3

7 -4 7 100.723 8.357 3

4 7 -7 96.5022 7.4548 3

4 7 7 104.394 8.612 3

4 -7 -7 88.3862 7.3278 1

7 4 7 100.071 8.805 3

-7 4 -7 94.2384 9.8695 2

-4 -7 7 101.756 8.839 1

-7 7 -4 98.0698 9.8164 2

-7 -4 7 93.4111 8.3923 1

-7 -7 4 98.1929 8.7884 1

-4 7 -7 90.4310 9.9875 2

7 -4 -7 95.8225 7.2065 1

7 -7 -4 83.4722 7.2859 1

7 7 4 109.472 9.074 3

7 7 -5 338.458 18.999 3

-7 5 7 348.660 24.124 2

-7 7 5 326.499 22.926 2

7 -5 7 351.242 19.486 3

7 -7 5 380.799 18.670 1

7 5 -7 374.269 18.896 3

-5 -7 -7 403.624 19.827 1

5 7 -7 366.284 19.033 3

5 -7 7 354.391 19.262 3

-5 7 7 376.165 23.961 2

-7 -7 -5 370.156 20.189 1

-5 7 -7 337.863 22.545 2

-5 -7 7 345.946 19.903 1

5 -7 -7 353.607 18.823 1

7 7 5 354.588 20.163 3

-7 -7 5 349.498 20.117 1

-7 -5 7 350.576 19.890 1

-7 5 -7 367.450 22.747 2

5 7 7 346.192 19.763 3

-7 7 -5 332.134 22.297 2

7 -7 -5 358.988 18.847 1

7 5 7 351.942 20.047 3

7 -5 -7 358.865 18.572 1

-7 -7 -6 248.786 14.830 1

6 -7 7 240.342 14.268 3

6 7 -7 232.306 13.491 3

-7 6 7 239.146 18.578 2

7 -6 7 250.683 14.421 3

7 6 -7 221.889 13.302 3

-6 7 7 228.189 18.083 2

7 7 -6 207.119 13.292 3

-7 7 6 229.135 17.801 2

7 6 7 235.262 14.998 3

7 7 6 214.260 14.696 3

6 7 7 216.434 14.512 3

-7 7 -6 199.044 16.599 2

6 -7 -7 229.197 13.576 1

-6 7 -7 240.992 17.495 2

7 -7 -6 222.058 13.441 1

7 -6 -7 232.794 13.514 1

-7 6 -7 230.766 17.301 2

-7 -7 6 245.292 15.200 1

-7 -6 7 234.866 14.995 1

-6 -7 7 220.653 14.757 1

-7 7 7 77.3655 10.9450 2

7 7 -7 70.8013 6.5849 3

-7 7 -7 75.2594 10.2126 2

7 7 7 77.1606 8.2618 3

7 -7 -7 86.4623 7.5973 1

-7 -7 7 79.6860 8.3717 1

-8 0 0 3859.77 178.92 2

0 -8 0 3734.06 169.80 1

8 0 0 3772.38 168.43 1

0 0 8 3711.16 165.65 3

0 8 0 4010.92 165.55 3

8 0 0 3700.23 166.26 3

0 8 0 4185.02 178.85 2

0 0 -8 3560.59 179.69 2

0 8 1 355.403 19.121 2

0 8 -1 380.237 17.540 3

0 8 1 376.199 17.690 3

8 -1 0 364.563 17.858 3

-8 -1 0 353.155 19.076 2

-8 1 0 379.425 20.176 2

8 1 0 336.348 18.434 3

1 0 8 365.797 17.885 3

1 0 -8 350.516 20.246 2

0 8 -1 405.224 19.662 2

-1 0 -8 346.464 20.197 2

0 -8 -1 338.876 19.132 1

0 -8 1 343.634 19.109 1

8 -1 0 343.704 17.732 1

8 1 0 358.000 17.874 1

-1 0 8 347.718 17.450 3

8 2 0 0.49583 2.36567 3

2 0 8 11.3652 2.6719 3

0 8 2 12.0127 2.5106 3

-8 2 0 -4.7498 2.7665 2

-2 0 -8 1.61903 2.56101 2

0 -8 -2 6.10915 3.00625 1

-2 0 8 3.23687 1.65626 3

0 8 -2 3.97730 1.91813 3

2 0 -8 3.96070 2.81841 2

0 -8 2 -0.7871 2.9055 1

8 -2 0 -0.3616 1.7296 1

0 8 -2 1.46876 2.42011 2

0 8 2 5.56073 2.36666 2

-8 -2 0 2.39665 2.35707 2

8 2 0 4.76936 2.15237 1

-8 -3 0 161.567 10.123 1

0 8 -3 158.604 10.632 2

3 0 -8 155.267 8.125 1

3 0 8 143.734 9.430 3

8 3 0 159.231 10.155 3

0 8 3 167.425 10.432 2

-3 0 8 145.874 8.458 3

-8 3 0 147.167 11.200 2

-3 0 -8 152.867 10.932 2

3 0 -8 153.893 10.889 2

0 -8 -3 145.036 10.228 1

0 -8 3 151.165 10.370 1

0 8 3 152.343 9.224 3

8 -3 0 148.413 8.924 1

0 8 -3 161.624 8.956 3

8 -4 0 2275.55 98.97 1

0 8 4 2222.82 105.57 2

4 0 -8 2316.12 106.40 2

-8 -4 0 2265.98 100.27 1

0 -8 -4 2273.89 100.19 1

-8 4 0 2083.05 106.44 2

-4 0 -8 2212.44 106.33 2

-4 0 8 2276.55 96.79 3

8 4 0 2237.03 98.60 3

0 8 -4 2108.92 105.91 2

0 8 -4 2135.33 97.10 3

0 -8 4 2082.79 99.86 1

4 0 -8 2277.54 98.21 1

0 8 4 2237.99 97.58 3

4 0 8 2219.47 98.01 3

-5 0 8 336.025 16.446 3

0 -8 5 329.096 18.432 1

5 0 -8 334.131 16.733 1

-5 0 -8 341.504 20.010 2

0 8 5 347.853 19.580 2

8 -5 0 334.544 17.380 1

0 -8 -5 359.049 18.490 1

0 8 5 337.070 17.584 3

5 0 -8 337.968 19.613 2

0 8 -5 334.376 19.923 2

0 8 -5 337.083 17.035 3

-8 5 0 332.474 20.439 2

5 0 8 335.166 17.962 3

8 5 0 337.741 18.214 3

-8 -5 0 350.930 19.139 1

0 -8 -6 1.34795 2.65987 2

0 8 -6 7.12573 2.47794 3

0 8 -6 0.33402 3.59316 2

6 0 -8 0.63997 2.01640 1

-6 0 8 2.82115 1.82968 3

8 6 0 4.45285 2.89123 3

8 -6 0 2.13589 2.38459 1

-8 -6 0 5.25182 3.94788 1

0 -8 -6 1.76223 2.70474 1

-8 6 0 5.96131 4.01455 2

0 8 6 5.98112 2.93879 3

-6 0 -8 5.21595 3.59035 2

6 0 -8 0.39623 3.16836 2

6 0 8 7.82587 3.42631 3

0 -8 6 2.32913 3.41797 1

0 -8 -7 74.4002 6.5481 1

0 8 -7 75.1511 8.2068 2

-7 0 8 74.0845 5.3069 3

7 0 8 75.8363 7.1572 3

-7 0 -8 57.2931 7.2045 2

0 8 -7 57.4617 5.5390 3

8 -7 0 69.8370 6.2432 1

7 0 -8 61.5025 6.8718 2

8 7 0 69.8481 6.5149 3

7 0 -8 65.0743 5.6192 1

-8 -7 0 67.1867 7.4696 1

-8 7 0 68.7317 8.0885 2

0 8 7 75.0313 6.6136 3

0 -8 -7 70.4334 6.6522 2

-1 8 0 397.886 19.665 2

0 -1 8 354.717 17.661 3

-1 -8 0 326.563 19.354 1

8 0 -1 362.323 17.878 1

8 0 -1 327.183 17.376 3

8 0 1 368.924 17.877 1

0 1 8 348.146 17.700 3

0 -1 -8 323.639 19.890 2

0 1 -8 329.136 20.192 2

-8 0 -1 357.548 19.553 2

-1 8 0 400.370 17.360 3

1 -8 0 363.620 19.265 1

-8 0 1 332.499 19.875 2

1 8 0 381.209 17.786 3

1 8 0 406.861 18.991 2

8 0 1 353.806 18.835 3

-1 -8 -1 46.3018 5.4826 1

-8 1 1 54.3433 6.1656 2

-1 8 1 52.1876 4.4468 3

8 -1 1 48.6807 4.3417 1

-8 -1 -1 55.5241 5.2131 2

8 -1 1 54.2239 5.3844 3

-1 1 8 56.5050 4.6170 3

1 -8 1 51.4909 5.6894 1

-1 8 1 59.5115 5.3547 2

1 1 -8 49.7020 5.9051 2

8 1 -1 57.8751 5.0017 3

1 8 -1 63.4576 5.5030 2

1 -1 8 49.3149 4.5653 3

8 1 -1 51.0079 4.6925 1

1 8 -1 56.9319 4.6969 3

-1 -1 -8 54.5279 5.6937 2

-1 -1 8 49.7990 4.3880 3

1 -1 -8 48.3391 5.6246 2

1 8 1 66.0861 5.3307 2

-8 1 -1 59.2242 5.9320 2

-8 -1 1 48.8813 5.1876 2

8 1 1 65.4710 6.0254 3

8 1 1 56.4730 4.8513 1

1 8 1 56.9669 4.8534 3

1 1 8 53.4160 4.7501 3

-1 8 -1 62.4106 5.7506 2

-1 8 -1 59.2745 4.4620 3

8 -1 -1 50.7675 4.1653 3

-1 1 -8 43.1032 5.7735 2

8 -1 -1 59.5533 4.8052 1

-1 -8 1 44.0691 5.7589 1

1 -8 -1 53.7007 5.5843 1

2 -1 8 140.955 8.815 3

2 1 -8 141.929 10.399 2

8 2 -1 150.618 9.186 3

-2 1 8 146.379 8.266 3

-8 2 1 136.102 10.676 2

-1 -8 -2 151.559 10.024 1

-1 8 2 135.738 9.435 2

1 8 -2 142.162 9.433 2

8 -2 1 144.578 8.425 1

1 8 -2 142.758 8.480 3

-2 -1 -8 151.573 10.398 2

-8 -2 -1 135.427 8.941 2

8 -2 1 145.467 8.998 3

-1 8 2 154.745 8.293 3

8 2 -1 145.926 8.836 1

1 -8 2 145.377 9.823 1

8 2 1 141.451 9.994 3

-1 8 -2 162.158 10.562 2

8 2 1 142.453 8.718 1

-2 1 -8 130.419 10.276 2

2 1 8 136.179 8.917 3

-1 8 -2 133.307 8.078 3

1 8 2 151.320 8.792 3

-2 -1 8 145.021 8.285 3

1 -8 -2 159.386 10.000 1

1 8 2 161.206 9.156 2

-8 2 -1 138.025 10.403 2

-8 -2 1 140.634 8.767 2

8 -2 -1 148.429 8.562 1

-1 -8 2 134.829 10.080 1

2 -1 -8 142.283 10.233 2

-8 3 1 7.73069 4.03868 2

-3 1 8 11.7347 2.2106 3

1 8 -3 8.40073 2.43646 3

-8 -3 -1 9.03076 3.19919 1

-3 -1 -8 7.22536 3.29846 2

8 -3 1 13.2231 2.4934 1

-1 8 3 7.67257 2.36252 3

3 1 -8 6.20339 3.14383 2

-1 -8 -3 14.9515 3.7332 1

3 -1 8 8.03448 2.71413 3

-1 8 3 12.3137 3.4320 2

8 3 -1 10.3675 2.6861 1

8 3 -1 7.74377 2.68599 3

1 8 -3 13.2573 3.4481 2

1 -8 3 4.55192 3.13398 1

3 -1 -8 7.36987 3.21961 2

-1 -8 3 6.48031 3.70559 1

8 3 1 12.3277 3.6487 3

1 8 3 11.0343 2.9450 2

1 8 3 4.53433 2.58114 3

-1 8 -3 11.9777 3.8132 2

-1 8 -3 8.42264 2.22092 3

8 -3 -1 6.86442 2.33173 1

-8 3 -1 9.05085 3.70664 2

1 -8 -3 10.3912 3.3322 1

-3 -1 8 9.57043 2.20671 3

3 1 8 11.2345 2.8959 3

-3 1 -8 13.3002 3.9311 2

3 -1 -8 9.12568 1.81683 1

8 -4 1 290.649 14.777 1

4 1 -8 268.791 14.046 1

8 4 -1 285.723 15.404 3

-4 1 8 279.123 13.931 3

-1 8 4 311.650 14.814 3

1 8 -4 278.336 14.590 3

1 8 -4 292.077 16.497 2

-1 8 4 291.491 16.975 2

-1 -8 -4 300.421 16.261 1

1 -8 4 289.339 15.865 1

-8 -4 -1 278.306 15.968 1

-8 4 1 231.402 17.697 2

4 -1 8 274.125 15.400 3

4 1 -8 292.652 17.063 2

-4 -1 -8 262.388 16.728 2

1 -8 -4 276.946 15.502 1

1 8 4 274.040 15.920 2

-8 -4 1 264.351 15.564 1

-4 -1 8 277.539 13.853 3

-1 8 -4 267.098 16.854 2

-8 4 -1 268.281 17.088 2

-1 8 -4 276.181 13.995 3

8 4 1 271.508 15.949 3

4 1 8 272.433 14.972 3

4 -1 -8 277.857 13.729 1

1 8 4 291.376 14.995 3

-1 -8 4 234.426 15.834 1

-4 1 -8 259.417 16.870 2

4 -1 -8 291.322 16.747 2

8 -4 -1 295.664 14.640 1

5 1 -8 150.890 8.583 1

8 5 -1 149.055 9.839 3

1 8 -5 156.344 10.813 2

-1 8 5 146.133 11.082 2

1 -8 5 150.633 9.907 1

-5 -1 -8 164.299 11.241 2

8 -5 1 143.726 9.141 1

5 -1 8 147.602 9.802 3

5 1 -8 137.431 10.449 2

5 1 -8 137.954 8.198 3

-1 8 5 149.142 9.385 3

-5 1 8 147.556 8.260 3

-1 -8 -5 154.446 10.218 1

1 8 -5 148.853 9.061 3

-8 -5 -1 148.993 10.668 1

-8 5 1 148.987 11.972 2

8 -5 -1 157.068 9.588 1

5 -1 -8 161.682 11.323 2

-8 5 -1 132.665 11.424 2

5 -1 -8 167.723 8.965 1

-5 1 -8 169.908 11.965 2

1 8 5 166.058 10.766 2

1 -8 -5 147.005 10.115 1

5 1 8 172.388 10.387 3

8 5 1 156.769 10.780 3

-1 8 -5 128.306 11.306 2

-1 -8 5 124.676 10.558 1

-8 -5 1 146.391 10.768 1

1 8 5 158.348 10.047 3

-1 8 -5 162.591 9.093 3

8 6 -1 102.051 8.125 3

-1 8 6 108.403 9.644 2

1 -8 6 108.780 8.112 1

8 -6 1 112.036 7.860 1

-8 6 1 96.8056 9.7615 2

6 1 -8 115.197 6.857 3

-1 8 6 113.717 8.034 3

6 -1 8 118.872 8.621 3

1 8 -6 109.040 9.444 2

-1 -8 -6 128.381 8.423 2

1 8 -6 106.139 7.526 3

6 1 -8 119.392 9.208 2

-1 -8 -6 109.847 8.545 1

-6 1 8 109.182 6.733 3

-8 -6 -1 110.876 9.366 1

6 1 -8 101.315 7.181 1

-6 -1 -8 114.514 9.533 2

8 6 1 114.860 8.829 3

8 -6 -1 102.884 7.652 1

1 -8 -6 99.9097 8.0839 1

6 -1 -8 110.247 7.260 1

-8 6 -1 103.030 9.737 2

-6 1 -8 120.060 10.059 2

1 8 6 121.984 8.497 3

-1 8 -6 119.710 7.603 3

-8 -6 1 99.2925 9.1916 1

6 -1 -8 106.322 9.449 2

6 1 8 129.491 9.034 3

-1 8 -6 119.745 10.422 2

-1 -8 6 105.289 8.980 1

-8 -7 -1 8.32094 4.22322 1

-8 7 1 2.94966 4.27421 2

7 -1 8 7.15033 3.71702 3

1 8 -7 9.57437 2.88379 3

7 1 -8 6.93891 2.15113 3

7 1 -8 5.37258 2.60868 1

1 8 -7 2.17353 3.90828 2

-1 -8 -7 1.75079 2.92474 1

7 1 -8 4.20660 3.30875 2

8 7 -1 4.57947 3.01618 3

-1 -8 -7 7.51045 3.01752 2

8 -7 1 6.70684 3.08627 1

-7 -1 -8 9.04970 4.26145 2

-8 7 -1 3.94971 3.94292 2

-8 -7 1 -1.3697 3.4128 1

-7 1 -8 5.05807 4.13794 2

1 -8 -7 1.48851 2.79977 1

7 1 8 2.79792 3.49309 3

8 -7 -1 4.55742 2.81077 1

7 -1 -8 4.78867 2.55048 1

-1 8 -7 4.55729 2.44082 3

1 -8 -7 4.06416 3.18470 2

8 7 1 7.75479 3.27880 3

-1 -8 7 4.38708 3.54912 1

7 -1 -8 2.34953 3.31841 2

1 8 7 4.21087 3.34115 3

-1 8 -7 0.30864 4.02401 2

8 0 2 0.98406 2.77323 3

8 0 -2 -0.0176 1.7020 1

-2 -8 0 4.77923 3.25610 1

0 -2 8 1.63255 1.68448 3

-2 8 0 1.26930 1.55904 3

0 -2 -8 0.98636 2.39561 2

0 2 -8 1.74174 2.51396 2

8 0 -2 0.37791 1.52605 3

-8 0 -2 2.11662 2.61993 2

-8 0 2 4.08369 3.05602 2

-2 8 0 1.58312 2.56276 2

0 2 8 -1.3660 1.7537 3

2 -8 0 -3.8387 2.1156 1

8 0 2 7.01260 2.14906 1

2 8 0 1.07902 2.02268 3

2 -8 1 155.870 9.883 1

1 -2 8 141.746 8.720 3

1 2 -8 153.752 10.845 2

-1 -2 -8 145.913 10.271 2

2 8 -1 152.299 8.813 3

-2 8 1 160.766 8.012 3

-1 2 8 137.037 8.580 3

-8 -1 -2 140.251 9.607 2

-8 1 2 136.109 10.447 2

8 1 -2 150.031 8.594 3

-2 8 1 148.165 10.031 2

-2 -8 -1 162.929 10.532 1

8 1 -2 141.708 8.742 1

8 -1 2 157.160 10.011 3

8 -1 2 140.693 8.454 1

2 8 1 147.698 8.751 3

8 1 2 159.005 8.731 1

-1 -2 8 146.100 8.469 3

1 2 8 154.600 8.900 3

-8 1 -2 142.922 10.218 2

1 -2 -8 131.615 9.880 2

-2 8 -1 136.353 7.793 3

8 -1 -2 144.897 7.586 3

2 -8 -1 145.612 9.651 1

8 1 2 138.126 10.002 3

-8 -1 2 146.336 9.631 2

-2 8 -1 155.449 10.134 2

-1 2 -8 136.482 10.533 2

8 -1 -2 142.336 8.580 1

-2 -8 1 134.215 10.120 1

8 2 -2 12.0348 2.7046 3

-2 8 2 12.3602 2.1980 3

-8 2 2 15.3745 4.2339 2

-2 8 2 12.5996 3.2116 2

8 -2 2 16.3639 2.6037 1

8 2 -2 10.9930 2.6536 1

-2 2 8 11.2144 2.3705 3

-8 -2 -2 10.4994 2.9725 2

-2 -8 -2 7.96631 3.53003 1

2 -2 8 11.6245 2.6934 3

2 2 -8 5.64131 3.24236 2

2 -8 2 8.38571 3.03605 1

8 -2 2 4.99611 2.90056 3

-2 -2 -8 12.3640 3.3871 2

2 8 -2 11.1269 2.5715 3

8 2 2 15.3494 3.8008 3

2 -2 -8 12.9401 3.4261 2

-2 -2 8 15.3412 2.4802 3

2 8 2 7.55225 2.61893 3

2 -2 -8 11.8937 1.8238 1

8 2 2 9.66948 2.63956 1

-8 -2 2 9.32885 2.81140 2

8 -2 -2 9.93475 2.37841 1

2 -8 -2 10.7567 3.1277 1

-8 2 -2 8.73376 3.73671 2

-2 2 -8 13.0550 3.8973 2

-2 8 -2 11.4977 3.5027 2

2 2 8 14.8812 3.0662 3

-2 8 -2 9.60933 2.09032 3

-2 -8 2 8.48385 3.54455 1

-3 2 8 90.6490 6.1556 3

-2 8 3 98.2267 8.1150 2

3 -2 8 93.9348 6.9295 3

8 3 -2 87.2104 6.7504 1

8 -3 2 107.935 6.468 1

3 2 -8 97.1789 8.2394 2

-8 -3 -2 94.6494 7.4250 1

-3 -2 -8 76.4962 7.5504 2

-8 3 2 81.3406 8.3214 2

8 3 -2 85.8558 6.8231 3

2 8 -3 95.9091 6.6668 3

-2 -8 -3 113.592 8.013 1

2 -8 3 100.670 7.435 1

-2 -8 3 89.2534 7.8793 1

3 -2 -8 98.2668 7.9665 2

-2 8 -3 96.1586 8.3805 2

2 8 3 94.9058 6.9606 2

8 -3 -2 92.6648 6.4878 1

2 -8 -3 93.5191 7.3559 1

-8 3 -2 98.7625 8.8858 2

3 -2 -8 97.2722 5.7614 1

-2 8 -3 83.8697 5.8460 3

-3 2 -8 81.8115 8.0659 2

-3 -2 8 101.308 6.288 3

8 3 2 91.9299 8.0988 3

3 2 8 95.8908 7.0198 3

2 8 3 79.2060 6.9050 3

8 -4 2 13.2067 2.7536 1

-8 4 2 17.2542 5.0380 2

2 8 -4 18.6057 3.1017 3

2 -8 4 14.7670 3.4457 1

4 -2 8 15.4564 3.4331 3

-4 -2 -8 14.2949 3.7939 2

8 4 -2 19.4208 3.5076 3

-2 8 4 16.1090 4.3049 2

4 2 -8 14.6914 2.5319 3

4 2 -8 21.4482 4.1586 2

-4 2 8 16.8493 2.5780 3

-2 -8 -4 13.7072 3.6586 1

-8 -4 -2 15.7340 3.9552 1

8 -4 -2 18.8437 3.0895 1

4 -2 -8 19.2510 2.6537 1

-4 2 -8 13.2389 4.1933 2

2 -8 -4 12.9864 3.4386 1

-2 -8 4 17.3406 4.2763 1

-8 -4 2 13.5894 3.7185 1

-8 4 -2 18.1059 4.9243 2

4 -2 -8 22.7901 4.2887 2

-2 8 -4 18.3906 4.6864 2

4 2 8 17.2174 3.5234 3

-2 8 -4 20.6536 2.8067 3

8 4 2 17.1647 4.3692 3

-4 -2 8 14.1394 3.0460 1

2 8 4 21.4589 3.8075 3

2 8 4 21.4041 3.4443 2

8 5 -2 117.415 8.329 3

2 -8 5 113.897 8.332 1

-8 5 2 102.628 10.188 2

2 8 -5 117.881 7.858 3

5 2 -8 117.003 9.234 2

5 -2 8 128.747 8.792 3

-5 2 8 125.640 7.214 3

-2 8 5 98.8185 9.7722 2

5 2 -8 122.054 7.252 3

-5 -2 -8 117.895 9.502 2

-2 -8 -5 140.393 9.359 1

8 -5 2 118.259 7.859 1

-2 -8 -5 126.929 8.196 2

-8 -5 -2 112.546 9.235 1

-5 2 -8 113.294 10.078 2

-2 8 -5 104.013 7.123 3

8 -5 -2 115.003 7.725 1

8 5 2 107.656 9.055 3

5 -2 -8 125.043 9.616 2

2 8 5 132.085 8.838 3

-8 5 -2 99.8941 9.8399 2

-5 -2 8 129.495 8.057 1

-2 -8 5 99.2250 9.2485 1

-8 -5 2 112.061 9.084 1

2 -8 -5 105.960 8.220 1

-2 8 -5 104.170 9.992 2

5 -2 -8 125.014 7.356 1

5 2 8 106.629 8.308 3

-2 -8 -6 11.4377 3.6097 1

6 2 -8 7.86093 2.29513 3

2 8 -6 8.13876 2.81195 3

2 -8 6 12.4229 3.2576 1

-6 -2 -8 14.8367 4.1889 2

6 2 -8 9.23032 3.48164 2

6 -2 8 6.71203 3.35029 3

8 -6 2 18.0379 3.3897 1

-2 -8 -6 7.32232 2.88313 2

-8 6 2 15.6691 5.0780 2

8 6 -2 15.2317 3.5545 3

-8 -6 -2 5.74600 3.94711 1

-2 8 6 11.9304 4.4829 2

-6 2 -8 12.2288 4.4809 2

8 -6 -2 6.84927 2.75005 1

2 8 6 5.79502 3.23601 3

8 6 2 3.05736 3.35942 3

-2 8 -6 9.30287 4.69263 2

6 2 8 6.31603 3.43135 3

2 -8 -6 9.39822 3.26939 1

6 -2 -8 6.30515 2.43823 1

-8 -6 2 11.5588 4.2348 1

-2 8 -6 11.6932 2.8968 3

-2 -8 6 11.7947 4.3340 1

6 -2 -8 1.71078 3.25360 2

-8 6 -2 4.53478 3.95815 2

-2 -8 -7 38.7042 4.7643 2

7 2 -8 37.8832 4.3824 1

7 2 -8 38.6991 5.2002 2

-2 -8 -7 39.6347 5.2274 1

7 -2 8 43.6329 5.5604 3

2 8 -7 37.8249 4.5493 3

8 7 -2 42.4257 5.0865 3

-7 -2 -8 31.9851 5.5712 2

7 2 -8 39.1351 3.8968 3

-8 -7 -2 42.6377 6.1813 1

8 -7 2 37.3261 4.6923 1

-8 7 2 41.5617 6.8209 2

2 -8 7 33.4567 4.7502 3

-2 8 7 39.3320 6.6562 2

-7 2 -8 38.6332 6.6738 2

7 -2 -8 39.0858 5.7744 2

2 -8 -7 36.1456 4.8505 1

-2 -8 7 15.5955 5.1370 1

-2 8 -7 28.8665 6.3209 2

8 7 2 32.7182 5.3289 3

2 8 7 35.7303 5.2236 3

-8 -7 2 38.6252 6.1122 1

7 2 8 37.7169 5.8433 3

7 -2 -8 32.1314 4.0793 1

-8 7 -2 34.5322 6.3151 2

8 -7 -2 35.0183 4.5796 1

-3 8 0 164.248 10.859 2

3 -8 0 143.959 9.843 1

3 8 0 165.780 9.414 3

0 -3 -8 157.664 10.535 2

-3 -8 0 152.482 10.574 1

0 3 8 169.837 9.400 3

0 3 -8 150.146 11.324 2

8 0 3 140.071 10.407 3

-8 0 3 157.598 11.096 2

8 0 -3 160.154 8.308 3

8 0 3 155.981 8.850 1

-8 0 -3 156.835 10.691 2

8 0 -3 159.281 9.142 1

0 -3 8 137.049 8.927 3

8 -1 3 12.5325 3.7901 3

-8 1 3 5.81296 3.76400 2

-1 3 8 8.99395 2.53427 3

8 1 -3 10.5705 2.5458 1

-1 -3 -8 12.0976 3.3080 2

1 -3 8 13.9723 2.7213 3

-3 8 1 7.48949 3.19893 2

-3 -8 -1 9.59410 3.53035 1

1 3 -8 13.9391 3.9353 2

3 8 -1 11.0596 2.6479 3

8 1 -3 9.82051 2.25336 3

8 -1 3 10.0632 2.3713 1

-8 -1 -3 10.8774 3.1931 2

3 -8 1 9.99145 3.14975 1

-1 3 -8 8.05220 3.56449 2

-8 1 -3 8.35212 3.47183 2

-8 -1 3 10.3502 3.4806 2

8 1 3 4.73948 3.29491 3

3 8 1 10.9139 2.6765 3

-3 -8 1 13.2522 3.9973 1

3 -8 -1 8.18710 3.02895 1

-3 8 -1 12.7164 3.5501 2

-3 8 -1 8.00526 1.80761 3

1 3 8 9.75749 2.82492 3

8 -1 -3 10.3054 1.6076 3

1 -3 -8 10.4138 3.2619 2

8 1 3 6.48656 2.24833 1

8 -1 -3 11.1533 2.5113 1

-1 -3 8 10.6290 2.4862 3

3 8 -2 96.0969 6.7825 3

8 2 -3 102.759 6.800 3

-8 -2 -3 89.3743 7.0336 1

-8 -2 -3 99.4850 7.3226 2

-2 3 8 95.3044 6.5622 3

-3 8 2 100.160 8.135 2

-8 2 3 91.5570 8.8204 2

8 -2 3 106.714 7.960 3

-2 -3 -8 95.0523 7.7664 2

-3 -8 -2 109.740 8.384 1

8 2 -3 98.8696 6.7814 1

8 -2 3 88.9891 6.1625 1

2 3 -8 86.1090 8.2635 2

3 -8 2 86.7558 7.1812 1

2 -3 8 92.2425 6.8237 3

2 3 -8 96.1492 6.2576 3

-8 -2 3 108.745 7.523 2

-3 8 -2 105.521 8.592 2

8 -2 -3 100.851 6.654 1

3 -8 -2 91.4435 7.2879 1

3 8 2 89.8863 6.8894 3

2 -3 -8 85.8349 7.5847 2

2 3 8 84.5883 6.8516 3

8 2 3 90.9012 8.0889 3

-2 3 -8 88.2349 8.5959 2

-3 -8 2 88.9884 8.0700 1

2 -3 -8 105.962 5.867 1

-3 8 -2 89.8185 5.6552 3

-8 2 -3 81.3168 8.0137 2

8 -3 3 8.53054 3.13793 3

-3 8 3 5.77142 3.34325 2

8 3 -3 4.11463 2.45965 1

3 3 -8 5.44466 3.23690 2

3 -3 8 1.72602 2.31643 3

-8 -3 -3 5.28224 2.65821 2

-3 -3 -8 2.61168 2.69268 2

3 -8 3 4.22141 2.79683 1

8 3 -3 3.63536 2.43741 3

-8 3 3 3.56307 3.93719 2

-3 -8 -3 -1.6999 2.9159 1

8 -3 3 4.39725 2.09987 1

3 3 -8 2.85466 1.79900 3

-8 -3 -3 7.44597 3.08482 1

-3 3 8 2.69021 1.98740 3

3 8 -3 5.20947 2.32738 3

3 -8 -3 4.26920 2.61506 1

-3 -8 3 3.14279 3.34506 1

3 -3 -8 2.32713 1.68686 1

8 3 3 1.28339 3.02358 3

-8 3 -3 2.08280 3.44562 2

3 -3 -8 2.71387 2.62081 2

3 8 3 1.99979 2.51354 3

-3 8 -3 5.35135 1.93006 3

8 -3 -3 3.94146 2.24167 1

-3 3 -8 2.36794 3.41656 2

3 3 8 4.35757 2.64539 3

-3 8 -3 3.63623 3.27225 2

-8 4 3 175.483 13.245 2

-3 8 4 156.067 12.424 2

-3 -8 -4 206.912 11.887 1

8 4 -3 171.386 10.559 3

-4 -3 -8 176.266 11.674 2

4 3 -8 183.978 12.230 2

4 3 -8 176.289 9.774 3

3 -8 4 168.735 10.793 1

8 -4 3 171.999 9.913 1

-4 3 8 187.463 9.916 3

4 -3 8 161.104 10.660 3

3 8 -4 177.909 10.445 3

-8 -4 -3 173.750 11.498 1

4 -3 -8 162.733 11.741 2

8 -4 -3 174.625 10.256 1

8 4 3 160.820 11.811 3

4 -3 -8 181.332 9.679 1

-4 3 -8 180.816 13.019 2

4 3 8 177.878 11.038 3

-4 -3 8 172.565 10.759 1

-3 -8 4 165.755 11.789 1

-3 8 -4 168.376 9.416 3

-3 8 -4 166.262 12.753 2

-8 4 -3 156.280 12.869 2

-8 -4 3 189.730 11.626 1

3 -8 -4 161.909 10.720 1

3 8 4 196.015 11.424 3

8 -5 3 76.0264 5.9707 1

5 3 -8 73.8363 7.0155 2

5 3 -8 73.1931 5.6084 3

3 -8 5 78.6753 6.4583 1

-8 -5 -3 70.5137 7.2279 1

-5 -3 -8 59.7813 6.8907 2

-3 -8 -5 76.9467 6.9859 1

-8 5 3 81.7591 8.6631 2

8 5 -3 78.9071 6.5853 3

3 -8 5 84.1508 6.3278 3

3 8 -5 82.8403 6.3098 3

-3 8 5 77.1141 8.3542 2

5 -3 8 77.1610 6.8265 3

-5 3 -8 77.0942 8.3297 2

8 -5 -3 67.4360 6.1474 1

8 5 3 79.2153 7.8026 3

-3 8 -5 67.2591 8.1002 2

3 -8 -5 78.6091 6.8368 1

5 -3 -8 74.0526 7.5369 2

-8 -5 3 77.5375 7.5275 1

3 8 5 77.7438 7.0426 3

5 -3 -8 78.6286 5.7727 1

5 3 8 73.4217 6.7555 3

-3 -8 5 70.1329 7.4212 1

-5 -3 8 63.2868 6.2977 1

-8 5 -3 86.1796 8.8874 2

-3 -8 -6 98.9071 7.8949 1

3 -8 6 90.0167 7.0385 1

3 -8 6 78.5866 6.9054 3

-3 8 6 107.776 9.849 2

6 -3 8 76.9525 7.3085 3

6 3 -8 83.8003 7.6652 2

-8 -6 -3 91.8152 8.2705 1

-6 -3 -8 82.4778 8.0923 2

-8 6 3 84.5617 9.2668 2

8 -6 3 106.223 7.154 1

8 6 -3 84.6172 7.1652 3

3 8 -6 86.6735 6.9126 3

6 3 -8 89.6748 6.3860 3

-8 -6 3 93.9793 8.5547 1

8 6 3 102.452 8.828 3

6 -3 -8 93.5782 6.7830 1

8 -6 -3 88.8142 7.1555 1

3 -8 -6 77.1510 7.2426 1

-6 -3 8 101.804 7.885 1

-8 6 -3 90.1223 9.5712 2

-3 -8 6 83.5183 8.2751 1

6 -3 -8 85.6639 8.3716 2

3 8 6 98.9138 8.1665 3

6 3 8 84.1203 7.9283 3

-3 8 -6 94.0484 9.8117 2

-6 3 -8 105.173 9.858 2

8 -7 3 32.9466 4.5246 1

-3 -8 -7 33.7945 4.9956 1

3 -8 7 32.3335 4.7089 3

3 8 -7 30.6348 4.3353 3

-8 7 3 31.3918 6.3974 2

7 -3 8 33.1854 5.2607 3

7 3 -8 35.8288 4.7842 2

-3 8 7 29.5863 6.5382 2

7 3 -8 32.4028 3.8227 3

-8 -7 -3 33.8159 5.7096 1

-7 3 8 36.1709 8.1674 2

8 7 -3 35.7832 4.7774 3

-7 -3 -8 36.5067 5.5085 2

-3 8 -7 22.0306 6.2204 2

-7 3 -8 39.1550 6.8575 2

8 7 3 25.2203 5.3576 3

7 -3 -8 40.6230 4.4325 1

3 -8 -7 35.1623 4.8181 1

-8 7 -3 23.0306 6.1955 2

-7 -3 8 25.7136 4.7628 1

-3 -8 7 32.8447 5.7484 1

3 8 7 40.0317 5.4376 3

8 -7 -3 36.0407 4.6382 1

-8 -7 3 40.8559 6.4824 1

7 3 8 28.5402 5.5261 3

4 8 0 2227.31 99.86 3

-4 8 0 2320.17 108.45 2

8 0 4 2355.91 100.82 1

0 -4 8 2149.71 99.59 3

-8 0 -4 2266.58 108.08 2

8 0 -4 2247.23 101.16 1

0 4 -8 2200.18 109.16 2

0 -4 -8 2321.90 107.98 2

8 0 -4 2301.20 98.50 3

8 0 4 2357.36 101.31 3

0 4 8 2224.63 99.69 3

4 -8 0 2201.69 101.82 1

-8 0 4 2264.12 108.86 2

-4 -8 0 2293.24 102.66 1

8 -1 4 308.378 16.370 3

-8 1 4 275.549 17.749 2

8 1 -4 286.323 13.988 3

4 8 -1 296.155 15.003 3

-1 -4 -8 283.945 16.290 2

1 -4 8 272.262 14.774 3

4 -8 1 287.744 15.450 1

-8 -1 -4 270.056 16.200 2

1 4 -8 280.496 17.291 2

-4 -8 -1 285.369 16.325 1

-1 4 8 294.342 14.732 3

8 1 -4 275.156 14.723 1

-4 8 1 271.240 17.040 2

8 -1 4 264.931 14.276 1

1 -4 -8 295.078 14.218 1

1 -4 -8 293.915 16.776 2

-1 -4 8 297.999 15.085 3

4 8 1 291.031 15.489 3

-1 4 -8 300.991 18.308 2

-4 8 -1 283.600 17.287 2

-4 -8 1 284.425 16.644 1

8 1 4 303.053 15.064 1

8 1 4 295.614 16.771 3

1 4 8 297.527 15.427 3

8 -1 -4 264.621 14.900 1

-8 1 -4 272.117 17.177 2

4 -8 -1 267.080 15.599 1

-8 -1 4 295.584 17.367 2

8 2 -4 18.3605 3.2115 1

-4 8 2 21.4201 4.5334 2

4 -8 2 15.1308 3.4132 1

-8 -2 -4 25.0370 4.0847 2

-4 -8 -2 15.4910 4.1742 1

2 -4 8 19.4034 3.2785 3

-2 4 8 21.4470 3.2164 3

-2 -4 -8 22.4438 3.7989 2

2 4 -8 13.6783 2.7350 3

8 -2 4 15.8448 2.7851 1

8 -2 4 22.0899 4.2962 3

8 2 -4 18.6793 2.8870 3

2 4 -8 19.8909 4.3428 2

-8 2 4 13.2573 4.9661 2

4 8 -2 28.1023 3.6289 3

-8 -2 -4 23.2013 3.5763 1

2 -4 -8 20.9277 2.6297 1

2 4 8 12.9835 3.3251 3

-8 2 -4 15.4485 4.3612 2

8 2 4 12.7359 4.1257 3

-4 -8 2 16.9049 4.3074 1

8 -2 -4 19.7972 3.0977 1

-2 4 -8 15.5234 4.5207 2

4 8 2 11.3776 3.1956 3

-4 8 -2 17.0426 4.2266 2

4 -8 -2 7.01589 2.97267 1

2 -4 -8 14.8522 3.5392 2

-8 -2 4 23.0342 4.3758 2

-8 3 4 192.040 13.900 2

8 3 -4 157.890 10.142 3

3 4 -8 198.993 12.731 2

-8 -3 -4 179.709 11.357 1

-4 -8 -3 196.861 11.934 1

8 -3 4 171.350 9.833 1

8 3 -4 188.527 10.759 1

4 -8 3 181.781 10.966 1

3 4 -8 178.103 10.081 3

-4 8 3 153.778 12.228 2

-8 -3 -4 185.974 11.325 2

8 -3 4 205.430 11.696 3

-3 -4 -8 181.407 11.712 2

3 -4 8 173.569 10.824 3

-3 4 8 169.088 9.972 3

4 8 -3 175.127 10.450 3

-8 3 -4 171.754 12.819 2

3 -4 -8 169.090 11.752 2

4 8 3 167.702 11.038 3

-3 4 -8 159.380 12.826 2

3 -4 -8 189.263 9.741 1

-4 8 -3 186.589 13.015 2

-4 -8 3 174.511 11.819 1

3 4 8 169.041 10.879 3

8 -3 -4 176.513 10.231 1

4 -8 -3 159.444 10.566 1

8 3 4 174.869 12.002 3

4 -8 4 2291.27 100.48 1

4 4 -8 2291.57 107.30 2

-8 -4 -4 2215.43 101.35 1

8 4 -4 2138.17 98.74 3

4 -8 4 2256.41 98.21 3

-4 8 4 2039.23 107.90 2

4 4 -8 2258.76 98.43 3

4 -4 8 2256.37 99.33 3

-4 -4 -8 2257.15 106.83 2

8 -4 4 2170.55 99.59 1

-8 4 4 2239.80 109.19 2

-4 -8 -4 2462.13 101.81 1

4 8 -4 2245.52 98.96 3

-8 -4 4 2214.60 99.75 1

4 4 8 2240.16 98.22 3

4 -8 -4 2138.56 99.23 1

4 8 4 2292.98 98.37 3

4 -4 -8 2106.06 105.61 2

8 -4 -4 2110.17 98.56 1

-4 -8 4 2185.40 100.05 1

-4 8 -4 2223.18 106.99 2

-8 4 -4 2200.92 106.98 2

8 4 4 2119.45 99.00 3

-4 -4 8 2324.01 99.41 1

-4 4 -8 2182.70 107.20 2

4 -4 -8 2265.79 98.14 1

4 -8 5 343.740 18.309 1

-8 -5 -4 355.621 19.706 1

5 -4 8 362.693 19.086 3

8 5 -4 331.604 18.400 3

8 -5 4 359.725 18.036 1

-5 -4 -8 366.911 20.215 2

-8 5 4 345.055 22.338 2

4 8 -5 340.275 18.337 3

-4 -8 -5 399.766 19.849 1

5 4 -8 357.812 17.904 3

4 -8 5 364.519 18.143 3

-4 8 5 354.916 21.996 2

5 4 -8 357.403 20.034 2

4 8 5 359.513 19.000 3

8 -5 -4 326.044 17.625 1

-8 5 -4 345.073 21.393 2

-8 -5 4 355.777 19.292 1

5 -4 -8 339.578 20.034 2

-4 8 -5 334.272 21.147 2

-5 -4 8 350.640 18.646 1

5 4 8 347.449 18.933 3

-5 4 -8 355.944 21.722 2

-4 -8 5 293.699 18.805 1

4 -8 -5 324.952 18.165 1

8 5 4 332.643 19.390 3

5 -4 -8 342.338 17.316 1

6 -4 8 18.0823 4.4229 3

-4 -8 -6 21.5306 4.3653 1

4 -8 6 14.1850 3.5202 3

-8 6 4 13.5397 5.5582 2

8 6 -4 20.3188 3.8227 3

4 8 -6 23.0163 3.6568 3

-8 -6 -4 25.6406 4.9946 1

-6 -4 -8 32.2039 4.9031 2

6 4 -8 16.9514 3.1613 3

-4 8 6 6.56152 4.87779 2

8 -6 4 16.6694 3.3250 1

-6 -4 8 20.2133 4.5716 1

4 -8 -6 24.8610 4.1226 1

-6 4 -8 20.6531 5.8414 2

-4 -8 6 19.9121 5.0268 1

8 6 4 18.5117 4.9322 3

6 -4 -8 19.7402 4.9354 2

6 4 8 13.3761 4.4459 3

4 8 6 22.5475 4.7084 3

6 -4 -8 21.8074 3.5922 1

-8 6 -4 15.2150 5.2091 2

8 -6 -4 19.0144 3.8707 1

-8 -6 4 18.9047 4.9732 1

-4 8 -6 13.0972 5.3854 2

4 -8 7 53.6177 5.8141 3

8 -7 4 53.8130 5.3841 1

-4 -8 -7 61.2477 6.3621 1

4 8 -7 44.5061 5.1796 3

-8 -7 -4 46.6977 6.5264 1

-4 8 7 67.6841 8.8170 2

-8 7 4 45.0846 7.6105 2

-7 4 8 43.2971 8.7880 2

-7 -4 -8 56.4553 6.3609 2

8 7 -4 44.8242 5.4052 3

7 4 -8 51.8423 5.0871 3

7 -4 8 59.4395 6.4799 3

8 -7 -4 46.2260 5.3208 1

7 4 8 39.8735 6.3088 3

-8 7 -4 29.6929 6.8156 2

-8 -7 4 51.9201 7.0783 1

8 7 4 51.3693 6.7699 3

-4 -8 7 42.9949 6.6376 1

4 -8 -7 46.7679 5.4334 1

-4 8 -7 38.5116 7.3018 2

-7 -4 8 47.2404 6.3413 1

-7 4 -8 51.3773 7.9017 2

4 8 7 59.1053 6.6609 3

7 -4 -8 47.1158 5.1042 1

8 0 5 362.186 18.977 3

0 -5 -8 353.978 18.984 2

-5 -8 0 351.449 18.964 1

-8 0 -5 321.458 19.331 2

-8 0 5 347.472 20.872 2

8 0 -5 327.906 15.928 3

8 0 5 359.803 16.922 1

0 5 8 328.498 17.478 3

5 -8 0 330.833 17.937 1

0 -5 -8 340.411 16.740 1

5 8 0 333.666 17.547 3

8 0 -5 324.316 17.236 1

0 -5 8 324.658 17.411 3

-5 8 0 354.635 20.020 2

0 5 -8 320.578 20.182 2

8 -1 5 151.860 8.802 1

-5 8 1 162.039 11.522 2

1 5 -8 152.509 9.118 3

-1 -5 -8 152.217 10.288 2

-1 5 8 140.202 9.304 3

1 -5 8 134.589 9.353 3

-8 -1 -5 151.527 10.812 2

8 1 -5 153.300 8.373 3

-8 1 5 151.272 12.393 2

1 5 -8 159.136 11.815 2

8 -1 5 150.414 10.766 3

5 8 -1 158.049 9.658 3

5 -8 1 155.046 9.909 1

-5 -8 -1 167.058 11.112 1

-8 -1 -5 155.832 9.439 1

8 1 -5 153.158 9.273 1

1 -5 -8 150.111 10.486 2

-8 1 -5 154.320 11.388 2

-1 5 -8 172.757 12.592 2

8 1 5 164.325 11.118 3

-5 -8 1 131.926 10.538 1

1 5 8 157.579 10.013 3

5 -8 -1 162.869 10.273 1

-5 8 -1 157.841 11.484 2

8 -1 -5 156.592 9.442 1

1 -5 -8 158.214 8.874 1

5 8 1 158.041 9.966 3

-8 -1 5 149.051 11.930 2

2 5 -8 123.841 7.767 3

-8 -2 -5 119.879 8.992 2

8 -2 5 126.767 9.300 3

8 2 -5 99.4109 7.4995 1

-8 -2 -5 109.627 8.172 1

2 -5 8 126.080 8.288 3

2 5 -8 116.432 9.780 2

-8 2 5 107.857 10.794 2

-2 -5 -8 139.953 8.987 2

5 8 -2 118.207 7.975 3

5 -8 2 117.220 8.324 1

-5 8 2 116.212 9.653 2

8 -2 5 113.856 7.224 1

-5 -8 -2 132.915 9.586 1

8 2 -5 112.168 7.248 3

-2 5 8 108.770 7.657 3

-8 2 -5 118.115 9.991 2

5 -8 -2 114.176 8.369 1

2 5 8 109.202 8.433 3

-5 -8 2 120.212 9.376 1

2 -5 -8 106.617 7.243 1

-2 5 -8 113.789 10.517 2

2 -5 -8 138.064 9.535 2

8 -2 -5 118.325 7.807 1

5 8 2 111.235 8.527 3

-5 8 -2 130.804 10.233 2

-8 -2 5 131.740 10.280 2

8 2 5 117.822 9.545 3

8 -3 5 88.0752 7.4043 3

3 5 -8 85.0960 8.0220 2

3 -5 8 69.8052 6.5232 3

-8 -3 -5 83.8175 7.2792 2

-5 8 3 70.7577 7.8167 2

-3 -5 -8 77.0125 6.8041 2

-8 3 5 71.3206 9.1721 2

-3 5 8 70.8374 5.9575 3

3 5 -8 76.0023 5.9026 3

5 -8 3 81.1721 6.6178 1

8 -3 5 76.3348 5.5503 1

-5 -8 -3 93.4044 7.9697 1

8 3 -5 71.8247 6.2338 1

5 8 -3 72.0258 6.2360 3

8 3 -5 81.6007 6.0088 3

-8 -3 -5 68.6051 6.7265 1

-3 5 -8 66.9707 8.2901 2

8 3 5 78.6484 7.7618 3

5 -8 -3 74.6479 6.6056 1

3 5 8 70.7807 6.8936 3

-8 3 -5 75.7836 8.3710 2

-5 -8 3 71.9107 7.3050 1

5 8 3 78.1424 7.0608 3

8 -3 -5 76.1244 6.1028 1

3 -5 -8 80.6218 6.0505 1

-5 8 -3 69.9297 8.1219 2

3 -5 -8 72.3268 7.1276 2

-4 -5 -8 354.923 19.619 2

4 -5 8 364.801 18.786 3

8 -4 5 347.079 17.410 1

-5 -8 -4 380.906 19.770 1

8 4 -5 353.061 18.032 3

5 -8 4 372.467 18.455 1

4 5 -8 342.831 17.823 3

4 5 -8 344.836 20.033 2

5 8 -4 328.539 18.097 3

-8 -4 -5 349.105 19.164 1

-8 4 5 362.030 22.907 2

-5 8 4 330.115 21.306 2

4 5 8 355.509 19.091 3

4 -5 -8 351.629 17.615 1

5 8 4 356.906 19.209 3

-5 -8 4 352.068 19.559 1

-4 5 -8 346.291 21.914 2

-5 8 -4 345.349 21.413 2

-8 4 -5 344.034 21.558 2

-4 -5 8 326.638 18.808 1

4 -5 -8 351.259 20.186 2

8 -4 -5 353.670 18.023 1

5 -8 -4 324.582 18.295 1

-8 -4 5 347.938 19.112 1

8 4 5 316.768 19.476 3

8 5 -5 174.674 11.096 3

5 -8 5 196.784 11.183 1

8 -5 5 185.213 10.568 1

-5 -5 -8 211.632 12.430 2

5 -5 8 185.596 11.851 3

-5 -8 -5 207.931 12.581 1

5 8 -5 192.322 11.288 3

-8 5 5 164.018 14.696 2

5 5 -8 169.832 10.741 3

-5 8 5 165.604 13.955 2

-8 -5 -5 178.474 12.216 1

-8 5 -5 172.606 14.053 2

8 -5 -5 168.893 10.732 1

5 -5 -8 173.970 12.652 2

-8 -5 5 168.159 12.121 1

-5 -5 8 186.869 12.027 1

-5 8 -5 172.659 14.147 2

8 5 5 161.617 12.335 3

5 5 8 171.192 12.004 3

5 -8 -5 175.892 11.202 1

5 8 5 188.785 12.389 3

-5 5 -8 200.253 14.839 2

5 -5 -8 174.937 10.545 1

-5 -8 5 178.606 12.332 1

6 -5 8 106.159 8.696 3

5 -8 6 97.8774 8.0399 3

-5 -8 -6 120.753 9.023 1

-8 6 5 91.5637 10.7851 2

5 8 -6 95.2793 7.4932 3

8 -6 5 96.2895 7.3261 1

6 5 -8 93.9555 7.2879 3

-5 8 6 111.133 11.133 2

-6 5 8 105.264 11.472 2

-6 -5 -8 99.8596 8.3225 2

8 6 -5 95.3503 7.7103 3

-8 -6 -5 97.8021 8.8084 1

8 6 5 103.769 9.198 3

-8 -6 5 94.5665 9.0022 1

-5 -8 6 106.774 9.376 1

8 -6 -5 100.932 7.753 1

6 -5 -8 99.7840 7.4265 1

5 -8 -6 90.3744 7.6952 1

-6 -5 8 96.8875 8.7349 1

-8 6 -5 93.1812 10.3208 2

6 5 8 95.7944 8.6829 3

5 8 6 77.0868 8.1646 3

-5 8 -6 95.8985 10.3532 2

-6 5 -8 98.1162 10.6492 2

-8 7 5 23.6721 6.8607 2

-5 -8 -7 20.3409 4.4321 1

-7 5 8 22.2592 7.5791 2

7 5 -8 23.5435 3.9642 3

5 -8 7 27.6253 4.7487 3

5 8 -7 28.3068 4.3783 3

7 -5 8 17.7781 4.9503 3

-8 -7 -5 25.9120 5.4442 1

-5 8 7 16.8324 6.5635 2

8 7 -5 13.6466 3.8287 3

7 5 8 21.0453 5.2995 3

5 8 7 24.5988 5.2532 3

-8 -7 5 25.3997 5.8072 1

8 -7 -5 22.2895 4.2912 1

8 7 5 21.9414 5.5802 3

5 -8 -7 16.2593 4.1473 1

7 -5 -8 20.3255 4.0397 1

-5 8 -7 19.0346 6.2325 2

-7 -5 8 18.0722 5.1927 1

-8 7 -5 14.2731 6.1138 2

-5 -8 7 30.7990 5.7355 1

-7 5 -8 25.7957 6.8842 2

0 6 8 7.77096 3.15279 3

-8 0 -6 1.70145 3.57595 2

0 -6 8 9.76496 3.04962 3

8 0 -6 4.53961 1.76366 3

6 8 0 5.82872 2.81751 3

-6 -8 0 3.54530 3.75601 1

6 -8 0 5.33766 2.92420 1

-6 8 0 2.06803 3.59573 2

-8 0 6 3.33218 4.49918 2

0 -6 -8 7.53097 2.95366 2

8 0 -6 8.61841 2.63560 1

0 6 -8 7.09190 4.17459 2

8 0 6 6.83177 3.79395 3

0 -6 -8 4.87989 2.37442 1

-8 1 6 105.119 10.715 2

8 1 -6 106.359 6.725 3

1 -6 8 110.475 8.165 3

-1 -6 -8 113.740 8.474 2

-6 -8 -1 119.801 9.395 1

8 1 -6 112.943 7.771 1

1 6 -8 131.863 10.405 2

6 -8 1 117.954 8.401 1

6 8 -1 116.543 8.145 3

-8 -1 -6 111.203 9.438 2

8 -1 6 119.107 9.183 3

-1 -6 -8 112.858 7.664 1

-6 8 1 121.387 10.201 2

-1 6 8 108.601 8.174 3

1 6 -8 120.703 7.814 3

8 1 6 98.9330 8.9930 3

1 6 8 123.396 8.642 3

1 -6 -8 111.216 8.526 2

1 -6 -8 108.046 7.436 1

-1 6 -8 102.800 10.018 2

8 -1 -6 103.159 7.442 1

6 -8 -1 116.353 8.311 1

-6 -8 1 115.841 9.208 1

-8 -1 6 103.607 9.916 2

-8 1 -6 108.762 9.544 2

-6 8 -1 117.762 9.813 2

6 8 1 118.429 8.357 3

8 2 -6 7.01254 2.23018 3

-2 6 8 2.14928 2.82445 3

6 -8 2 11.8503 3.4099 1

2 -6 8 12.2534 3.3889 3

-8 -2 -6 11.5671 3.6789 2

8 -2 6 8.90871 3.77547 3

2 6 -8 16.7487 4.6193 2

-8 2 6 9.95690 5.57531 2

8 2 -6 5.28391 2.59494 1

2 6 -8 7.53331 2.61084 3

6 8 -2 7.43200 2.78829 3

-6 -8 -2 0.23741 3.52402 1

-2 -6 -8 11.3782 3.0973 2

-6 8 2 8.08734 4.24352 2

8 -2 -6 6.58490 2.62918 1

-2 6 -8 2.62711 3.82772 2

-6 -8 2 10.8205 4.1687 1

2 6 8 7.66887 3.50678 3

-8 2 -6 3.95843 3.83432 2

-6 8 -2 7.80540 4.16495 2

8 2 6 8.16699 4.04151 3

-8 -2 6 2.91236 4.33361 2

2 -6 -8 13.9357 3.6301 2

6 8 2 9.30541 3.27714 3

6 -8 -2 10.8766 3.1884 1

2 -6 -8 6.07257 2.56026 1

-8 -3 -6 89.2512 7.6618 1

-6 -8 -3 83.2653 7.9827 1

6 8 -3 93.1791 7.1091 3

8 3 -6 74.3631 6.1185 3

-3 -6 -8 92.7409 7.3901 2

6 -8 3 91.9787 7.2209 1

3 6 -8 85.9702 6.7649 3

3 -6 8 93.7056 7.3066 3

-6 8 3 88.8020 8.9328 2

-8 -3 -6 93.7036 7.9046 2

-8 3 6 95.1664 10.6711 2

3 6 -8 91.4325 8.4347 2

8 -3 6 97.6325 8.0141 3

6 8 3 94.6557 8.1757 3

-3 6 -8 80.4574 9.4999 2

3 -6 -8 90.1555 6.7977 1

8 3 6 89.3167 8.4156 3

-6 8 -3 90.6744 9.2926 2

3 6 8 87.4492 7.8131 3

-8 3 -6 84.4535 9.1864 2

8 -3 -6 81.7994 6.6868 1

6 -8 -3 81.1187 7.1634 1

3 -6 -8 86.2203 7.8964 2

-6 -8 3 100.431 8.790 1

-8 -3 6 96.1835 7.9292 1

6 -8 4 18.3980 3.6590 1

-6 -8 -4 20.5149 4.8887 1

-4 -6 -8 16.1323 3.5265 2

8 4 -6 16.2887 3.3394 3

6 8 -4 9.59966 3.22050 3

8 -4 6 16.4136 4.2832 3

-6 8 4 26.5048 5.8142 2

4 6 -8 12.3556 3.1969 3

-8 -4 -6 16.1885 4.2704 1

-8 4 6 15.6122 6.6422 2

4 -6 8 25.0105 4.2273 3

6 -8 -4 17.9341 3.9186 1

-8 -4 6 17.0467 4.3662 1

4 -6 -8 19.4555 4.6893 2

6 8 4 18.5968 4.5102 3

8 4 6 18.5164 4.7459 3

4 6 8 25.1955 4.9435 3

-4 6 -8 15.4462 5.6295 2

4 -6 -8 12.4868 3.2815 1

-8 4 -6 15.2813 5.5004 2

-6 8 -4 9.72474 4.97611 2

8 -4 -6 14.5401 3.4297 1

-6 -8 4 22.4309 5.0464 1

-5 -6 -8 95.7752 7.6293 2

8 5 -6 98.7859 7.3419 3

-6 8 5 74.6542 9.4276 2

-5 6 8 105.310 11.195 2

6 8 -5 86.3270 7.2438 3

-8 -5 -6 106.528 8.482 1

6 -8 5 94.4320 7.3435 1

5 -6 8 84.5470 7.7288 3

5 6 -8 81.0758 6.9304 3

-8 5 6 87.6620 11.1790 2

-6 -8 -5 91.2663 8.2197 1

8 5 6 94.5724 8.8542 3

6 8 5 104.851 9.098 3

-8 5 -6 86.4599 10.0228 2

5 -6 -8 95.2230 7.3395 1

-6 8 -5 95.9295 10.3001 2

8 -5 -6 88.3607 7.3609 1

6 -8 -5 98.4880 8.0475 1

-5 -6 8 101.236 8.692 1

-6 -8 5 98.1982 8.8112 1

-5 6 -8 101.848 10.809 2

5 6 8 78.5502 8.2679 3

-8 -5 6 89.5443 8.5127 1

8 6 -6 -0.3379 2.6412 3

6 6 -8 -0.4978 2.5152 3

-6 -8 -6 2.89257 3.87634 1

-8 -6 -6 -2.6960 3.7086 1

-6 6 8 -1.9131 5.0892 2

-6 8 6 2.22071 5.01317 2

-8 6 6 9.58491 5.88685 2

6 -6 8 2.88161 3.81680 3

6 8 -6 1.61993 2.70751 3

-6 -8 6 -2.6653 3.8913 1

-8 6 -6 -1.0790 4.6490 2

-8 -6 6 0.86400 4.28531 1

6 -6 -8 -0.9826 2.6305 1

6 6 8 -0.1079 3.5487 3

-6 -6 8 3.28303 3.98493 1

-6 6 -8 -1.7936 4.5386 2

8 6 6 3.51434 3.91090 3

-6 8 -6 7.67759 5.17336 2

6 -8 -6 0.35891 2.95811 1

6 8 6 -4.3115 3.2046 3

8 -6 -6 -0.0734 2.8344 1

-8 -7 -6 34.9682 6.2347 1

-7 6 8 32.5488 8.5418 2

6 8 -7 34.0874 4.8224 3

-8 7 6 37.4985 8.1877 2

7 -6 8 38.6796 6.1292 3

7 6 -8 26.9868 4.3648 3

-6 8 7 21.9885 7.0965 2

8 7 -6 33.2845 4.8801 3

6 -8 -7 28.6222 4.9305 1

-6 -8 7 31.5254 6.1458 1

-8 7 -6 28.2245 7.1784 2

7 -6 -8 28.8201 4.6765 1

8 -7 -6 25.2502 4.6407 1

-8 -7 6 32.5771 6.2867 1

6 8 7 33.7047 5.8737 3

-7 -6 8 24.3898 5.7857 1

8 7 6 35.4100 6.1963 3

7 6 8 23.2715 5.5242 3

-6 8 -7 22.8019 6.7905 2

-7 6 -8 34.3272 7.5958 2

8 0 7 78.4805 7.6176 3

-7 8 0 72.0790 7.8791 2

7 -8 0 59.9020 6.0173 1

0 -7 -8 66.6983 6.3196 2

-8 0 7 60.5736 8.5427 2

0 -7 -8 70.8414 6.1678 1

0 7 -8 71.9461 8.2591 2

8 0 -7 55.9665 5.4109 1

7 8 0 75.5077 6.5273 3

-7 -8 0 63.5510 7.1727 1

0 7 8 54.1341 6.1349 3

0 7 -8 65.4187 5.7466 3

-8 0 -7 64.6336 7.3002 2

1 7 -8 3.34121 2.64017 3

-8 1 7 6.67308 5.41672 2

-1 7 8 3.37630 3.06584 3

8 -1 7 3.25433 3.51383 3

-8 -1 -7 9.36488 4.00639 2

-7 -8 -1 8.14888 4.12333 1

-1 -7 -8 5.83039 3.01783 2

8 1 -7 5.92107 2.08945 3

-1 -7 -8 7.04126 3.00729 1

8 1 -7 3.15051 2.55047 1

7 -8 1 5.17087 3.16153 1

1 7 -8 3.00394 4.07619 2

7 8 -1 8.52745 3.08016 3

1 -7 8 2.40676 2.97078 3

-7 8 1 3.88360 4.22694 2

-8 1 -7 2.82536 3.91659 2

-8 -1 7 4.05488 4.88669 2

-7 -8 1 8.32661 4.27764 1

1 -7 -8 8.87822 3.26041 2

7 -8 -1 2.08335 2.92094 1

-7 8 -1 1.27878 3.58452 2

1 -7 -8 5.44958 2.87337 1

-1 7 -8 2.14997 4.13783 2

8 -1 -7 3.48612 2.46583 1

1 7 8 3.28927 3.39083 3

8 1 7 13.7644 4.6141 3

7 8 1 5.07957 2.95579 3

7 8 -2 38.9276 4.7204 3

-8 2 7 22.6036 7.2810 2

2 -7 8 38.7009 4.9414 3

2 7 -8 30.9686 5.6874 2

8 2 -7 33.9487 4.3272 1

-7 -8 -2 32.6640 5.7094 1

8 2 -7 38.9958 3.8879 3

-7 8 2 38.4491 6.4465 2

7 -8 2 35.4034 4.7953 1

-8 -2 -7 31.2933 5.3109 2

-2 -7 -8 36.9979 4.7437 2

2 7 -8 27.2683 4.1313 3

8 -2 7 43.5493 5.7812 3

8 2 7 42.8956 6.1163 3

8 -2 -7 42.6930 4.5902 1

-7 -8 2 34.2497 5.9732 1

7 8 2 34.8295 5.1024 3

-8 2 -7 37.7067 6.3868 2

2 -7 -8 36.0829 4.4336 1

2 -7 -8 41.1739 5.3566 2

-8 -2 7 31.8259 6.4866 2

7 -8 -2 29.9957 4.7117 1

-2 7 -8 44.0461 7.1867 2

-7 8 -2 36.5422 6.4492 2

2 7 8 40.6292 5.4372 3

3 -7 8 30.5840 4.7553 3

7 8 -3 26.3498 4.3053 3

3 7 -8 33.7333 4.3454 3

-3 -7 -8 35.5191 4.4976 2

8 3 -7 33.2876 3.9126 3

-7 -8 -3 33.1099 5.7609 1

8 -3 7 31.8655 5.2230 3

-8 3 7 28.9507 7.9342 2

7 -8 3 27.0539 4.5133 1

-8 -3 -7 28.7903 4.9634 2

-7 8 3 23.7185 5.8595 2

-3 7 -8 27.0745 6.5884 2

7 8 3 31.1690 5.2061 3

-7 -8 3 31.8493 5.7791 1

8 -3 -7 25.2130 3.9964 1

-8 -3 7 29.1594 5.0512 1

3 7 8 32.0761 5.3126 3

-7 8 -3 22.6919 6.0362 2

3 -7 -8 35.9695 4.4970 1

7 -8 -3 29.8422 4.4876 1

3 -7 -8 33.4745 5.3511 2

-8 3 -7 36.4160 6.6250 2

8 3 7 27.4313 5.3677 3

-7 -8 -4 62.5405 6.8415 1

8 -4 7 42.2274 6.0475 3

7 8 -4 46.1547 5.2811 3

7 -8 4 50.8652 5.5481 1

-4 7 8 50.7638 8.2037 2

-4 -7 -8 55.9574 5.4831 2

8 4 -7 40.0892 4.7128 3

-8 4 7 60.5519 9.6092 2

4 7 -8 38.0343 4.9315 3

4 -7 8 51.5405 6.0331 3

-7 8 4 31.6422 6.7532 2

8 4 7 43.6033 6.4970 3

-4 7 -8 45.2424 8.0025 2

-7 -8 4 32.7802 6.2031 1

4 -7 -8 49.9181 5.2870 1

7 -8 -4 46.9551 5.5682 1

-8 4 -7 47.7925 7.5967 2

8 -4 -7 33.5844 4.7451 1

-8 -4 7 49.3118 6.3555 1

-7 8 -4 59.5250 8.0265 2

7 8 4 37.9074 5.9072 3

4 7 8 48.6214 6.3483 3

7 8 -5 20.4902 4.0758 3

-7 8 5 28.8950 6.9034 2

-5 7 8 27.9701 7.3190 2

8 5 -7 25.5604 4.1213 3

5 -7 8 20.6704 4.7212 3

5 7 -8 24.4708 4.1922 3

-8 5 7 14.8731 7.4140 2

7 -8 5 23.7198 4.1995 1

-7 -8 -5 31.5794 5.7626 1

5 7 8 26.1886 5.2872 3

-5 7 -8 21.5824 6.8565 2

5 -7 -8 21.8151 4.0959 1

-8 -5 7 22.0371 5.3574 1

8 -5 -7 22.1383 4.2353 1

8 5 7 27.2730 5.7025 3

-7 -8 5 14.5074 5.2072 1

-7 8 -5 17.7706 6.1675 2

-5 -7 8 23.2481 5.5713 1

7 -8 -5 22.3922 4.4595 1

-8 5 -7 26.6049 6.7575 2

7 8 5 26.8784 5.5716 3

6 -7 8 28.6251 5.7215 3

6 7 -8 37.0496 4.8608 3

7 8 -6 27.6305 4.6392 3

-7 8 6 29.5898 7.6034 2

8 6 -7 33.3873 4.6583 3

-8 6 7 32.6588 8.8006 2

-7 -8 -6 39.1114 5.9470 1

-6 7 8 26.0384 7.6083 2

-8 -6 7 30.4138 6.2627 1

-6 -7 8 29.0939 6.0408 1

-7 -8 6 35.3258 6.4020 1

8 -6 -7 36.8925 5.1237 1

7 -8 -6 39.8799 5.4067 1

-6 7 -8 33.1264 7.5635 2

6 -7 -8 34.0929 5.0029 1

-7 8 -6 14.4686 6.5794 2

6 7 8 30.3887 5.9680 3

-8 6 -7 32.0519 7.4384 2

8 6 7 27.8290 5.9077 3

7 8 6 38.0317 6.3554 3

7 8 -7 45.9379 5.4774 3

-7 8 7 53.5030 9.3668 2

8 7 -7 50.4215 5.6396 3

-7 7 8 51.3758 9.7894 2

7 7 -8 32.7372 5.1206 3

-8 7 7 45.3271 9.0748 2

-7 -8 7 60.3486 7.6916 1

8 7 7 56.1162 7.4415 3

7 7 8 52.2430 7.2693 3

7 8 7 46.3546 7.0204 3

8 -7 -7 50.3310 6.1169 1

-8 -7 7 51.5239 7.4978 1

-7 7 -8 63.3211 9.3239 2

-7 -7 8 52.7914 7.4391 1

-7 8 -7 41.6762 8.2310 2

-8 7 -7 56.0264 9.1447 2

7 -8 -7 46.3767 5.9379 1

7 -7 -8 50.2883 6.0950 1

0 8 8 1460.53 63.07 3

8 0 8 1424.38 64.12 3

8 8 0 1324.73 63.03 3

0 -8 -8 1523.69 67.54 2

0 8 -8 1357.89 69.36 2

0 8 -8 1341.66 62.26 3

-8 0 8 1387.14 70.42 2

-8 8 0 1379.30 69.32 2

8 -8 0 1422.64 63.92 1

8 0 -8 1289.90 62.90 1

-8 -8 0 1357.52 65.16 1

0 -8 -8 1304.67 63.54 1

-8 0 -8 1383.23 68.76 2

8 1 -8 242.483 13.878 1

-8 -8 -1 254.130 16.165 1

1 8 -8 258.033 14.180 3

-8 1 8 244.971 18.990 2

1 -8 8 242.166 14.710 3

-8 8 1 239.277 17.293 2

-1 -8 -8 267.318 15.244 2

1 8 -8 254.297 16.783 2

8 1 -8 247.164 12.833 3

-8 -1 -8 260.793 16.427 2

8 -8 1 263.272 14.745 1

-1 -8 -8 235.832 14.530 1

8 -1 8 252.193 15.587 3

8 8 -1 246.818 14.525 3

8 -8 -1 261.920 15.013 1

-1 8 -8 250.662 17.659 2

-8 8 -1 242.753 17.297 2

1 -8 -8 247.207 14.602 1

8 1 8 265.607 16.081 3

-8 1 -8 261.528 17.209 2

1 8 8 260.455 15.158 3

-8 -1 8 248.394 17.794 2

8 -1 -8 247.574 14.138 1

1 -8 -8 286.168 15.991 2

-8 -8 1 244.683 16.137 1

8 8 1 239.834 14.845 3

8 -2 8 31.8903 5.7248 3

2 8 -8 25.2754 4.2197 3

2 -8 8 24.0318 4.7189 3

-8 2 8 35.9472 8.1179 2

-2 -8 -8 38.0826 4.8683 2

-8 -8 -2 22.9257 5.5344 1

8 -8 2 28.3974 4.6929 1

-8 -2 -8 28.4104 5.4765 2

8 2 -8 27.5144 3.5858 3

-8 8 2 23.1928 6.2638 2

8 8 -2 31.9565 4.6785 3

8 2 -8 33.1334 4.3867 1

8 -8 -2 26.1726 4.5568 1

-2 8 -8 23.8930 6.4033 2

-8 -8 2 30.7316 6.0681 1

-8 8 -2 25.7855 6.3767 2

-8 2 -8 28.4476 6.4299 2

8 2 8 38.8346 6.1375 3

2 8 8 26.7773 5.2310 3

8 8 2 31.9658 5.1324 3

2 -8 -8 36.4256 4.8632 1

8 -2 -8 28.7237 4.2802 1

8 -8 3 129.311 9.103 1

-8 -8 -3 120.481 10.434 1

3 8 -8 119.474 8.558 3

3 -8 8 104.073 8.850 3

-3 8 8 127.020 11.960 2

8 8 -3 108.295 8.681 3

-8 -3 -8 106.331 9.801 2

-8 8 3 117.049 11.192 2

8 -3 8 142.366 10.342 3

-8 3 8 125.496 13.260 2

8 3 -8 116.573 7.829 3

-8 -3 8 123.299 9.715 1

-8 8 -3 125.151 11.816 2

-8 3 -8 98.9472 10.9458 2

-3 8 -8 135.603 12.494 2

8 -3 -8 122.598 8.658 1

8 8 3 105.575 9.550 3

-8 -8 3 106.991 10.176 1

3 -8 -8 127.861 9.072 1

8 3 8 124.215 10.351 3

3 8 8 119.173 9.788 3

8 -8 -3 120.491 9.093 1

8 8 -4 886.186 44.390 3

8 4 -8 936.031 43.714 3

8 -4 8 1029.46 45.71 3

-4 8 8 959.459 50.935 2

-8 -8 -4 944.322 46.272 1

-8 8 4 965.509 50.465 2

-8 4 8 917.084 52.140 2

8 -8 4 1004.85 44.93 1

4 -8 8 936.468 44.894 3

4 8 -8 928.883 44.295 3

4 8 8 893.873 43.769 3

-4 8 -8 897.427 49.290 2

-8 4 -8 985.245 49.367 2

8 -8 -4 873.555 43.541 1

8 8 4 872.794 44.071 3

-8 -4 8 858.618 44.102 1

4 -8 -8 971.452 43.713 1

8 -4 -8 910.312 43.077 1

-8 -8 4 903.276 45.007 1

-8 8 -4 943.482 48.883 2

8 -5 8 268.358 16.261 3

5 -8 8 264.535 15.794 3

5 8 -8 258.085 14.964 3

-5 8 8 276.050 19.679 2

-8 5 8 229.094 19.980 2

8 8 -5 234.052 14.818 3

-8 -8 -5 306.406 16.929 1

-8 8 5 251.165 18.725 2

8 5 -8 264.806 14.701 3

-8 5 -8 299.346 19.730 2

8 -8 -5 248.765 15.298 1

8 -5 -8 262.734 15.166 1

-8 8 -5 285.506 19.405 2

-8 -8 5 239.632 16.768 1

-8 -5 8 277.742 16.840 1

5 -8 -8 262.920 15.480 1

5 8 8 255.168 16.216 3

8 8 5 240.360 16.217 3

8 5 8 245.177 16.400 3

-5 8 -8 261.218 19.586 2

6 8 -8 23.6072 4.3986 3

-6 8 8 24.5827 7.8039 2

-8 6 8 36.5692 9.4518 2

8 6 -8 28.7919 4.6717 3

-8 8 6 23.3837 7.6933 2

8 8 -6 28.3972 4.6606 3

-8 -8 -6 23.5737 5.7860 1

8 8 6 28.1029 6.1040 3

8 -8 -6 30.5148 5.3024 1

8 6 8 31.9302 6.0928 3

6 8 8 38.0293 6.3864 3

-8 8 -6 22.4294 7.0201 2

-8 6 -8 28.1329 7.4423 2

-6 8 -8 33.5965 7.8505 2

8 -6 -8 28.4694 5.1540 1

6 -8 -8 30.1833 5.2075 1

-8 -8 6 22.3625 6.4322 1

-8 -6 8 21.9802 5.9145 1

7 8 -8 44.8301 5.6526 3

-8 7 8 39.6172 9.6245 2

-7 8 8 61.4674 10.3845 2

8 8 -7 49.5570 5.8798 3

-8 8 7 64.4103 10.3159 2

8 7 -8 43.9033 5.6398 3

-8 7 -8 45.6457 9.0070 2

-8 -8 7 37.8417 7.5130 1

8 8 7 39.3783 7.0235 3

7 8 8 51.4117 7.3706 3

-7 8 -8 37.5648 8.4438 2

8 7 8 53.5199 7.3701 3

8 -7 -8 50.8855 6.3505 1

8 -8 -7 56.9700 6.6471 1

-8 8 -7 55.0755 9.3617 2

7 -8 -8 43.7554 6.2253 1

-7 -8 8 48.9282 7.3916 1

-8 -7 8 42.9750 7.6759 1

-8 8 8 596.625 36.119 2

8 8 -8 582.053 29.357 3

8 -8 -8 618.636 31.621 1

-8 8 -8 610.960 36.764 2

0 0 -9 1.50594 2.85840 2

0 0 9 0.28540 1.94456 3

0 9 0 -1.8960 1.9524 3

0 9 0 1.38452 2.52019 2

9 0 0 2.36067 2.51954 3

0 -9 0 -3.1699 2.8748 1

9 0 0 -3.5728 1.7822 1

-9 0 0 -5.8433 3.0902 2

9 -1 0 526.237 25.747 1

0 -9 1 506.663 27.381 1

0 9 -1 555.924 25.344 3

0 9 1 562.679 25.357 3

-9 1 0 509.698 28.381 2

-1 0 9 522.540 25.436 3

9 -1 0 563.934 25.496 3

9 1 0 522.083 26.260 3

1 0 -9 516.376 28.862 2

-9 -1 0 512.878 27.479 2

-1 0 -9 494.743 28.727 2

9 1 0 569.842 26.085 1

1 0 9 521.034 25.794 3

0 -9 -1 559.085 27.611 1

0 9 1 596.187 27.759 2

-9 2 0 47.0058 6.2631 2

-2 0 -9 43.8509 5.7705 2

0 9 2 48.4651 4.8407 2

0 9 -2 47.6636 4.4058 3

-2 0 9 40.5990 4.1150 3

0 -9 2 33.9360 5.1647 1

0 9 -2 56.8117 5.7881 2

0 9 2 52.6933 4.5287 3

9 2 0 39.2355 5.0729 3

0 -9 -2 39.5049 5.3901 1

2 0 -9 54.2713 6.1512 2

9 2 0 52.1016 4.8955 1

2 0 9 48.4797 4.9079 3

9 -2 0 51.4435 4.5881 1

-9 3 0 327.108 21.160 2

-9 -3 0 368.215 19.452 1

0 -9 -3 346.670 19.492 1

9 -3 0 351.760 18.246 1

0 9 3 373.561 18.022 3

3 0 9 351.451 18.733 3

0 9 -3 399.778 20.662 2

0 -9 3 329.421 19.689 1

9 3 0 376.091 19.356 3

0 9 3 360.580 19.745 2

3 0 -9 374.073 20.995 2

0 9 -3 374.408 17.997 3

-3 0 -9 353.211 20.983 2

-3 0 9 354.238 17.636 3

-9 4 0 94.5884 9.7065 2

4 0 9 97.8234 7.8851 3

0 9 -4 107.681 9.136 2

4 0 -9 112.228 9.168 2

0 -9 4 97.3981 8.8967 1

9 -4 0 108.642 7.512 1

-4 0 9 108.273 6.861 3

0 9 4 97.4455 8.5038 2

-9 -4 0 104.746 8.813 1

0 -9 -4 105.755 8.646 1

9 4 0 102.955 8.473 3

-4 0 -9 117.920 9.521 2

0 9 4 109.587 7.623 3

0 9 -4 105.205 7.220 3

0 9 5 573.647 29.623 2

-9 -5 0 551.016 28.713 1

-5 0 9 544.841 25.918 3

0 9 -5 528.621 26.336 3

0 9 5 576.846 26.960 3

-5 0 -9 542.082 30.178 2

0 -9 -5 563.496 28.107 1

9 -5 0 562.744 27.137 1

0 9 -5 585.765 30.358 2

0 -9 5 548.280 28.311 1

-9 5 0 520.004 30.759 2

5 0 9 540.531 27.458 3

5 0 -9 569.324 30.028 2

9 5 0 536.356 27.627 3

0 9 6 22.5903 4.2383 3

-9 -6 0 21.7804 5.2994 1

0 9 -6 24.1623 3.6620 3

-6 0 -9 16.6527 4.7956 2

6 0 9 22.5478 4.6015 3

9 -6 0 16.5057 3.7283 1

6 0 -9 22.2861 4.7227 2

9 6 0 24.3428 4.5274 3

6 0 -9 18.0817 3.1818 1

0 9 6 21.6385 4.8583 2

0 -9 6 21.1418 4.6525 1

-6 0 9 21.4973 3.1179 3

0 9 -6 13.4697 4.8990 2

0 -9 -6 21.4082 4.2990 1

-9 6 0 24.6724 6.0130 2

0 -9 -7 131.150 9.830 1

9 -7 0 140.295 9.648 1

7 0 9 143.797 10.658 3

-7 0 -9 146.476 11.541 2

9 7 0 132.649 9.810 3

-7 0 9 123.375 8.108 3

0 9 -7 120.905 8.617 3

-9 7 0 141.322 12.095 2

7 0 -9 132.498 8.613 1

0 9 7 147.908 11.315 2

0 9 -7 128.934 11.480 2

7 0 -9 124.451 10.637 2

-9 -7 0 103.533 10.487 1

0 9 -8 223.308 13.111 3

9 8 0 204.521 13.625 3

8 0 9 252.128 15.164 3

9 -8 0 219.001 13.629 1

0 9 8 235.260 15.534 2

-9 -8 0 206.157 15.139 1

8 0 -9 215.135 12.775 1

-8 0 -9 215.944 15.587 2

-9 8 0 234.901 16.582 2

0 -9 -8 232.187 13.922 1

9 0 1 531.417 26.059 1

0 1 9 519.153 25.927 3

0 1 -9 533.347 29.356 2

-9 0 1 534.355 28.479 2

1 9 0 571.266 25.868 3

-1 9 0 619.568 28.712 2

0 -1 9 527.316 25.955 3

9 0 -1 537.672 25.631 3

-9 0 -1 542.959 28.466 2

-1 9 0 562.707 25.441 3

9 0 -1 563.771 26.373 1

0 -1 -9 533.542 29.057 2

9 0 1 535.592 27.026 3

1 -9 0 530.981 27.576 1

-1 -9 0 525.495 27.627 1

9 -1 1 475.824 23.689 1

-1 -1 -9 487.059 26.676 2

1 -1 9 479.690 23.827 3

-9 1 1 439.964 26.278 2

9 -1 1 478.350 24.325 3

-1 9 1 547.275 23.299 3

1 1 -9 455.268 26.732 2

-1 9 1 507.265 25.835 2

-1 1 9 510.032 23.661 3

-1 -9 -1 479.850 25.249 1

9 1 -1 483.898 23.795 3

1 9 -1 527.211 23.626 3

1 -9 1 491.077 25.327 1

-9 -1 -1 490.320 25.505 2

9 1 -1 494.349 23.986 1

1 1 9 487.161 24.332 3

9 -1 -1 491.955 24.288 1

-1 -1 9 498.022 23.951 3

9 1 1 509.970 24.384 1

-1 -9 1 481.323 25.832 1

1 -9 -1 484.376 25.656 1

-1 9 -1 560.659 26.761 2

-9 1 -1 469.182 26.697 2

1 9 1 516.616 24.048 3

-9 -1 1 491.360 26.038 2

9 1 1 485.952 25.420 3

-1 9 -1 531.555 23.663 3

-1 1 -9 513.580 27.659 2

1 -1 -9 489.403 27.046 2

1 -9 2 30.6918 4.7849 1

-2 -1 -9 30.2522 4.9403 2

9 2 -1 25.4769 3.8965 3

2 -1 9 36.8617 4.2322 3

-1 9 2 34.8508 4.4895 2

1 9 -2 32.6775 3.8128 3

2 1 -9 30.1662 5.0367 2

-1 -9 -2 29.0878 4.7405 1

-2 1 9 28.1248 3.4841 3

-9 -2 -1 34.9576 4.1558 2

9 2 -1 28.2442 3.8219 1

-1 9 2 39.3013 3.7067 3

-9 2 1 23.4010 5.1676 2

9 -2 1 28.0721 3.6173 1

-1 9 -2 29.8264 3.4693 3

-2 1 -9 32.7126 5.2555 2

1 -9 -2 36.6515 4.9921 1

1 9 2 37.9858 3.9944 3

-2 -1 9 29.1957 3.5170 3

9 2 1 32.1744 5.0778 3

2 -1 -9 34.5066 5.1003 2

-9 2 -1 26.1679 4.9438 2

-1 -9 2 28.2344 4.9051 1

9 2 1 29.8490 3.9373 1

2 1 9 27.3267 4.0812 3

9 -2 -1 30.0418 3.7760 1

-1 9 -2 38.5776 5.1652 2

1 -9 3 660.735 33.525 1

-9 3 1 656.816 36.203 2

9 -3 1 670.387 32.221 1

-1 -9 -3 706.699 33.902 1

-3 -1 -9 654.017 35.633 2

9 3 -1 634.238 32.479 3

-9 -3 -1 672.742 33.368 1

1 9 -3 694.206 31.896 3

-3 1 9 702.939 31.626 3

-1 9 3 692.856 35.056 2

9 3 -1 710.331 32.866 1

3 -1 9 646.243 32.463 3

-1 9 3 747.010 31.602 3

3 1 -9 705.514 36.103 2

1 9 3 704.381 32.457 3

9 3 1 695.134 34.024 3

-1 9 -3 810.621 36.707 2

-1 -9 3 662.841 34.098 1

-3 1 -9 633.005 36.217 2

1 -9 -3 689.247 33.987 1

-3 -1 9 689.243 31.945 3

1 9 3 719.969 34.767 2

-1 9 -3 650.042 31.767 3

9 -3 -1 699.317 32.761 1

-9 3 -1 665.395 36.500 2

3 1 9 689.281 32.867 3

3 -1 -9 686.869 36.081 2

9 -4 1 457.044 22.083 1

-9 4 1 410.712 25.334 2

4 1 -9 465.283 25.041 2

9 4 -1 424.301 22.530 3

1 9 -4 454.786 21.833 3

-9 -4 -1 446.769 23.496 1

1 -9 4 432.477 23.127 1

-1 -9 -4 463.552 23.445 1

-1 9 4 433.915 24.330 2

4 -1 9 426.905 22.545 3

-4 1 9 455.062 21.305 3

-4 -1 -9 440.297 24.796 2

4 -1 -9 436.385 24.467 2

1 9 4 437.464 21.875 3

-4 1 -9 432.057 24.878 2

-9 -4 1 430.589 23.122 1

-9 4 -1 409.745 24.989 2

-1 9 -4 429.930 21.117 3

-1 9 -4 497.023 25.227 2

1 9 4 456.369 23.576 2

4 1 9 434.948 22.243 3

-4 -1 9 455.008 21.150 3

-1 -9 4 383.277 22.953 1

9 4 1 431.046 23.179 3

1 -9 -4 430.516 22.977 1

9 -4 -1 446.078 21.866 1

-1 9 5 377.274 22.352 2

-9 -5 -1 398.115 21.753 1

9 -5 1 389.456 20.014 1

1 9 -5 416.086 19.882 3

-1 -9 -5 394.189 21.127 1

5 -1 9 373.349 20.569 3

-5 -1 -9 389.822 22.553 2

-5 1 9 402.579 19.067 3

9 5 -1 390.335 20.657 3

5 1 -9 387.977 22.439 2

-9 5 1 371.888 23.492 2

1 -9 5 404.610 20.994 1

1 9 5 406.400 20.372 3

-1 9 -5 389.067 19.347 3

1 -9 -5 378.497 20.842 1

-5 -1 9 396.996 19.190 3

-9 -5 1 362.375 21.446 1

-9 5 -1 366.615 23.207 2

5 1 9 392.116 20.653 3

9 -5 -1 392.370 20.108 1

-1 -9 5 371.459 21.295 1

-1 9 -5 414.944 23.231 2

5 -1 -9 413.041 22.830 2

1 9 5 388.556 21.775 2

9 5 1 396.636 21.236 3

-5 1 -9 409.472 23.324 2

-9 -6 -1 26.2682 5.7307 1

-6 1 9 30.3919 3.5195 3

-1 9 6 35.3255 6.0690 2

-1 -9 -6 35.0334 5.2301 1

-6 -1 -9 40.4420 6.0168 2

1 9 -6 32.2705 4.2486 3

6 1 -9 26.3289 5.0903 2

-9 6 1 38.2308 6.7807 2

9 6 -1 35.4406 4.9419 3

9 -6 1 38.9994 4.7438 1

6 -1 9 37.1892 5.3186 3

1 -9 6 36.4801 5.1814 1

9 -6 -1 43.1595 4.9302 1

1 -9 -6 30.5442 4.8658 1

-9 -6 1 34.8148 5.9939 1

6 -1 -9 39.1302 4.0914 1

-6 -1 9 32.8815 3.8481 3

6 -1 -9 30.9071 5.4990 2

6 1 9 34.8081 5.2857 3

1 9 6 43.6453 5.7145 2

-1 9 -6 37.5025 6.4301 2

9 6 1 34.8428 5.1959 3

-6 1 -9 25.7263 5.7164 2

-1 9 -6 28.7875 3.9889 3

-1 -9 6 35.8753 5.3492 1

-9 6 -1 32.3423 6.5255 2

1 9 6 33.3590 4.8337 3

-7 -1 -9 346.387 20.893 2

1 9 -7 319.018 17.843 3

9 -7 1 351.485 18.629 1

7 -1 9 345.376 19.468 3

-9 -7 -1 349.193 20.405 1

7 1 -9 325.699 17.694 1

-9 7 1 333.857 21.557 2

-1 -9 -7 360.435 19.206 1

7 1 -9 357.036 20.476 2

-1 9 7 362.670 21.334 2

9 7 -1 348.682 18.880 3

-9 7 -1 321.985 21.931 2

1 9 7 362.229 19.249 3

9 7 1 333.483 19.426 3

7 1 9 347.652 19.858 3

9 -7 -1 368.703 19.278 1

-7 -1 9 347.314 17.644 3

7 -1 -9 346.426 18.175 1

1 -9 -7 378.307 19.761 1

-7 1 -9 389.945 22.190 2

1 9 7 352.197 20.616 2

7 -1 -9 378.336 21.369 2

-9 -7 1 351.687 20.860 1

-1 9 -7 348.062 22.136 2

-1 9 -7 337.498 18.209 3

-8 1 9 175.532 15.948 2

8 1 -9 175.449 11.143 1

-1 9 8 169.040 13.964 2

-1 -9 -8 220.663 12.665 2

-9 8 1 154.674 14.086 2

8 -1 9 183.428 13.122 3

-9 -8 -1 177.329 13.541 1

8 1 -9 179.923 12.921 2

-8 -1 -9 201.550 13.848 2

1 -9 8 185.636 12.308 3

1 9 -8 180.946 11.370 3

9 -8 1 198.361 12.320 1

-1 -9 -8 187.330 12.255 1

9 8 -1 162.588 11.603 3

-1 9 -8 164.342 14.296 2

8 1 9 171.735 12.928 3

1 -9 -8 178.872 12.035 1

-9 -8 1 178.082 13.582 1

-9 8 -1 184.669 14.541 2

1 9 8 208.948 12.575 3

8 -1 -9 180.471 11.056 1

-8 1 -9 170.056 13.833 2

9 -8 -1 201.205 12.247 1

9 8 1 179.009 12.149 3

9 0 -2 46.5090 4.0374 3

0 2 -9 48.5242 6.2623 2

9 0 -2 45.5319 4.5084 1

9 0 2 54.6824 6.1332 3

9 0 2 57.8187 4.7788 1

2 9 0 47.0715 4.5426 3

0 -2 -9 30.5356 4.9999 2

-2 9 0 48.4557 4.0904 3

0 -2 9 47.1581 4.5740 3

0 2 9 49.3516 4.7392 3

-9 0 2 54.2064 6.0802 2

-9 0 -2 47.8897 5.4402 2

2 -9 0 48.1846 5.6031 1

-2 9 0 51.2978 5.5204 2

-2 -9 0 45.4513 5.9025 1

1 2 -9 31.8698 5.3865 2

9 1 -2 32.6374 3.6456 3

-1 -2 -9 17.9830 4.1594 2

9 -1 2 30.7869 4.6562 3

-2 9 1 33.2308 4.6345 2

-1 2 9 31.0954 3.7569 3

1 -2 9 28.5531 3.8152 3

9 -1 2 29.2109 3.4722 1

-2 -9 -1 30.1561 4.9386 1

9 1 -2 32.8827 3.7537 1

2 9 -1 21.8188 3.3275 3

-9 -1 -2 27.2791 4.2454 2

-9 1 2 22.6564 4.9731 2

2 -9 1 28.4513 4.6051 1

-2 -9 1 29.8800 5.1135 1

-9 -1 2 22.4325 4.3319 2

-1 -2 9 29.2161 3.6938 3

-2 9 -1 36.6786 3.3025 3

9 1 2 30.2111 5.0899 3

-9 1 -2 28.9641 4.8622 2

-1 2 -9 30.8883 5.3001 2

9 1 2 29.0691 3.7529 1

2 9 1 32.1005 3.8374 3

2 -9 -1 28.7193 4.6385 1

1 2 9 40.9355 4.3406 3

1 -2 -9 31.0175 4.6254 2

9 -1 -2 26.8753 3.5604 1

-2 9 -1 30.8313 4.7686 2

-9 2 2 13.9129 4.5173 2

9 -2 2 16.0130 2.8260 1

9 2 -2 11.2040 2.8749 3

2 2 -9 7.46763 3.73881 2

2 9 -2 17.2274 3.1160 3

2 -2 9 14.1176 3.2455 3

-9 -2 -2 7.68804 3.14049 2

-2 2 9 19.8252 3.0968 3

9 2 -2 14.1607 3.0348 1

-2 -9 -2 4.46832 3.36378 1

2 -9 2 16.5194 3.8596 1

-2 9 2 9.46584 3.28863 2

-2 -2 -9 13.6357 3.8482 2

-2 -2 9 13.8390 2.7719 3

-9 2 -2 13.6047 4.4530 2

2 -9 -2 17.0554 3.7959 1

-2 9 -2 12.0220 2.4546 3

9 2 2 16.5181 3.3038 1

9 2 2 16.6666 4.3665 3

-2 9 -2 14.3785 4.2067 2

2 2 9 13.6015 3.3126 3

2 -2 -9 10.1020 3.4842 2

-2 -9 2 11.2285 4.1340 1

2 9 2 11.8410 2.9181 3

9 -2 -2 9.83907 2.57652 1

-2 2 -9 10.7713 4.1065 2

-3 2 9 2.80803 2.20810 3

3 -2 9 5.37075 2.83138 3

-2 -9 -3 3.83991 3.40873 1

-3 -2 -9 6.43748 3.24059 2

2 -9 3 7.73770 3.45161 1

9 3 -2 7.49725 2.87282 3

-2 9 3 8.24720 3.35141 2

9 -3 2 8.41635 2.51284 1

3 2 -9 6.48919 3.51777 2

-9 -3 -2 7.44573 3.36503 1

2 9 -3 8.85252 2.64553 3

9 3 -2 5.04254 2.70718 1

-9 3 2 3.72967 4.07580 2

-2 9 -3 7.32610 2.22632 3

-9 3 -2 -0.5394 3.7185 2

9 3 2 12.5922 4.1483 3

-3 2 -9 -1.4821 3.0214 2

-2 -9 3 5.88364 3.92304 1

9 -3 -2 6.43385 2.53216 1

2 9 3 3.18727 2.49936 3

-2 9 -3 7.31511 3.88532 2

-3 -2 9 9.06161 2.56835 3

2 -9 -3 3.27701 2.94620 1

3 2 9 4.45414 2.89573 3

3 -2 -9 7.80882 3.18689 2

2 9 -4 50.6626 4.9219 3

-4 2 9 51.8396 4.4950 3

9 4 -2 42.9520 5.0254 3

-9 -4 -2 57.1368 6.3359 1

4 -2 9 42.2294 5.3579 3

4 2 -9 51.3956 6.2704 2

-4 -2 -9 46.8846 5.9982 2

-9 4 2 55.2144 7.2725 2

2 -9 4 40.5312 5.3129 1

9 -4 2 42.3294 4.5315 1

-2 9 4 36.7934 5.6904 2

-2 -9 -4 44.4124 5.6028 1

9 4 2 46.2634 6.3129 3

9 -4 -2 47.2889 4.7936 1

2 -9 -4 38.3994 5.3546 1

2 9 4 55.7547 5.5306 3

4 2 9 46.1358 5.3531 3

-4 2 -9 46.5330 6.6286 2

4 -2 -9 55.4512 6.2164 2

-2 -9 4 34.3190 6.0350 1

-2 9 -4 45.6677 4.2529 3

-2 9 -4 59.1969 7.0126 2

-4 -2 9 49.8274 4.5108 3

-9 -4 2 49.0178 5.9639 1

-9 4 -2 28.4208 6.1513 2

-5 2 9 127.488 7.640 3

-9 5 2 119.033 11.083 2

9 5 -2 109.925 8.343 3

5 2 -9 114.136 9.516 2

-2 9 5 109.397 10.182 2

-5 -2 -9 126.394 9.841 2

2 -9 5 118.976 8.845 1

-9 -5 -2 93.9250 9.0798 1

9 -5 2 119.403 8.167 1

5 -2 9 110.192 8.880 3

2 9 -5 119.418 8.019 3

-2 -9 -5 119.630 9.303 1

5 -2 -9 124.440 9.844 2

5 -2 -9 121.112 7.407 1

-9 5 -2 92.6418 9.9356 2

-2 9 -5 124.534 10.713 2

2 -9 -5 109.405 8.713 1

-2 -9 5 107.644 9.804 1

5 2 9 111.940 8.665 3

9 -5 -2 116.688 8.186 1

9 5 2 105.158 9.398 3

-9 -5 2 110.395 9.451 1

-5 -2 9 112.527 7.407 3

2 9 5 102.405 8.489 3

-5 2 -9 114.316 10.313 2

-2 9 -5 109.175 7.430 3

9 -6 2 32.3936 4.5020 1

2 -9 6 42.5823 5.0516 1

-9 6 2 45.2977 7.1501 2

-9 -6 -2 20.2303 5.4553 1

2 9 -6 44.0604 4.8016 3

6 2 -9 40.0492 3.9650 3

-2 -9 -6 51.9783 5.7999 1

9 6 -2 30.7307 4.7700 3

-6 -2 -9 33.0259 5.6021 2

-2 9 6 36.5725 6.2899 2

6 2 -9 42.9938 5.8430 2

6 -2 9 41.0270 5.6555 3

9 6 2 31.9571 5.6366 3

-6 2 -9 38.5719 6.6003 2

-2 -9 6 23.2995 5.5908 1

6 -2 -9 28.1765 5.4747 2

-9 6 -2 37.4117 6.8911 2

2 -9 -6 36.2457 5.1841 1

-2 9 -6 42.3520 4.3970 3

6 2 9 32.6280 5.3841 3

-2 9 -6 42.1803 7.0928 2

2 9 6 44.7713 5.6628 3

9 -6 -2 39.8985 4.8339 1

6 -2 -9 39.1176 4.2514 1

-9 -6 2 39.6839 6.2319 1

-9 7 2 39.8451 7.3495 2

7 2 -9 53.8017 6.3829 2

-2 9 7 38.1489 6.8443 2

7 -2 9 36.1112 5.8988 3

7 2 -9 37.5550 4.1735 3

9 -7 2 52.7736 5.6106 1

-2 -9 -7 43.9869 5.7720 1

9 7 -2 39.4406 5.2800 3

2 -9 7 45.6537 5.5838 3

-7 -2 -9 40.7626 6.3081 2

2 9 -7 43.5316 5.0201 3

-9 -7 -2 56.5521 7.2613 1

-9 -7 2 33.9968 6.5658 1

7 2 9 40.9842 6.2772 3

9 -7 -2 44.9902 5.4382 1

-9 7 -2 38.7472 7.0629 2

7 -2 -9 40.8087 6.3422 2

-7 2 -9 31.3687 6.6687 2

-2 9 -7 44.4978 7.6116 2

7 -2 -9 34.2500 4.3748 1

9 7 2 38.0963 5.8540 3

-2 -9 7 39.8079 6.2634 1

2 -9 -7 40.7821 5.4566 1

-7 -2 9 36.0933 5.3140 1

2 9 7 36.9370 5.6386 3

-9 8 2 51.3208 8.2309 2

-9 -8 -2 43.9809 7.0741 1

2 -9 8 40.7999 5.9557 3

8 -2 9 59.2289 7.1400 3

-8 2 9 60.5376 9.9325 2

-8 -2 -9 52.2323 7.0875 2

2 9 -8 48.6793 5.4580 3

-2 -9 -8 58.9297 6.3734 1

-2 9 8 45.6577 7.5992 2

9 8 -2 43.6928 5.7551 3

9 -8 2 48.2346 6.0526 1

8 2 -9 52.5833 4.8058 3

-2 9 -8 49.9906 8.1247 2

-9 -8 2 63.8523 7.9850 1

9 -8 -2 45.8978 5.9206 1

8 -2 -9 45.3228 5.2449 1

-8 2 -9 46.7946 7.6035 2

-8 -2 9 50.5020 6.3355 1

8 2 9 43.7821 6.9501 3

2 9 8 66.9660 6.9932 3

9 8 2 48.9746 6.3115 3

2 -9 -8 43.6857 5.9699 1

-9 8 -2 45.1230 7.7464 2

-3 9 0 407.284 21.070 2

-9 0 -3 333.088 20.368 2

-9 0 3 366.110 20.811 2

-3 -9 0 369.199 20.214 1

3 9 0 364.146 18.510 3

9 0 3 375.420 18.455 1

0 3 -9 352.061 21.407 2

9 0 3 394.516 20.352 3

0 3 9 376.347 18.640 3

9 0 -3 353.185 18.563 1

9 0 -3 361.942 17.376 3

0 -3 9 364.217 18.463 3

0 -3 -9 359.298 20.557 2

3 -9 0 357.505 19.620 1

-9 1 3 663.067 35.944 2

1 3 -9 694.404 36.521 2

-3 -9 -1 687.441 34.042 1

9 -1 3 659.332 33.509 3

3 9 -1 708.790 32.247 3

-1 -3 -9 651.907 35.247 2

9 1 -3 672.788 32.546 1

1 -3 9 694.416 32.414 3

-9 -1 -3 701.615 34.984 2

-1 3 9 683.331 32.124 3

9 -1 3 697.215 32.228 1

9 1 -3 685.197 31.521 3

3 -9 1 684.191 33.600 1

-3 9 1 721.975 35.591 2

9 -1 -3 678.324 32.950 1

-1 -3 9 688.242 32.585 3

1 -3 -9 683.806 35.941 2

1 3 9 713.526 32.996 3

-9 -1 3 694.785 35.451 2

-3 9 -1 786.086 36.493 2

9 1 3 705.997 32.887 1

-9 1 -3 689.273 36.211 2

-3 -9 1 695.043 34.573 1

-1 3 -9 682.957 37.017 2

3 -9 -1 674.292 34.022 1

9 1 3 692.653 34.505 3

3 9 1 683.009 32.655 3

9 2 -3 3.00930 2.38739 1

-3 9 2 6.40812 3.27670 2

-2 -3 -9 7.42549 3.27478 2

3 -9 2 1.74965 2.91672 1

3 9 -2 5.05442 2.43672 3

-9 2 3 14.4218 4.6224 2

9 -2 3 8.99509 3.60662 3

-9 -2 -3 5.32297 3.07448 2

2 3 -9 6.95380 3.73055 2

9 2 -3 3.75587 2.43516 3

-2 3 9 8.33265 2.69727 3

-3 -9 -2 5.76996 3.67559 1

9 -2 3 6.10004 2.42673 1

2 -3 9 5.00274 2.69767 3

-9 -2 -3 4.59573 2.96518 1

2 3 9 8.13671 3.01258 3

9 2 3 5.64420 3.92896 3

2 -3 -9 6.01585 3.13038 2

-2 3 -9 2.54713 3.41703 2

9 -2 -3 5.43922 2.59103 1

-2 -3 9 5.27319 2.45962 3

-3 9 -2 9.80473 3.86458 2

3 9 2 2.14355 2.54110 3

3 -9 -2 6.37269 3.26406 1

-9 2 -3 1.30187 3.24074 2

-3 -9 2 6.52632 3.73693 1

-3 -3 -9 473.166 25.989 2

9 3 -3 468.340 23.884 1

3 -3 9 469.390 23.924 3

9 -3 3 492.793 23.339 1

-3 9 3 440.898 25.892 2

3 3 -9 487.851 26.725 2

-3 3 9 494.099 23.172 3

-3 -9 -3 519.186 25.225 1

9 3 -3 475.810 23.539 3

-9 3 3 440.965 26.932 2

3 9 -3 514.327 23.670 3

-9 -3 -3 477.379 24.617 1

3 -9 3 476.001 24.486 1

-3 3 -9 440.225 26.495 2

3 -9 -3 439.395 23.804 1

3 9 3 466.284 23.375 3

-3 9 -3 422.512 21.739 3

-3 9 -3 558.371 26.869 2

3 -3 -9 463.086 25.533 2

9 3 3 462.823 24.869 3

-3 -3 9 503.408 22.806 3

3 3 9 468.600 23.504 3

-9 3 -3 460.633 26.328 2

-3 -9 3 450.846 24.632 1

9 -3 -3 459.763 23.039 1

-3 -9 -4 312.530 16.189 1

9 4 -3 247.714 14.733 3

-9 4 3 287.856 18.062 2

3 9 -4 276.796 14.702 3

4 3 -9 253.534 16.649 2

4 3 -9 277.419 14.048 3

9 -4 3 259.075 14.323 1

4 -3 9 263.155 15.318 3

-3 9 4 250.064 16.765 2

-9 -4 -3 259.579 15.906 1

-4 -3 -9 272.944 16.482 2

3 -9 4 278.020 15.425 1

-4 3 9 277.792 14.247 3

-9 4 -3 262.262 17.199 2

-4 3 -9 266.436 17.430 2

4 3 9 258.089 14.860 3

-3 9 -4 239.136 13.073 3

9 4 3 257.690 16.088 3

3 9 4 267.790 14.888 3

-9 -4 3 250.482 15.237 1

-3 9 -4 300.899 17.620 2

9 -4 -3 245.931 14.034 1

4 -3 -9 260.472 16.188 2

-3 -9 4 220.561 15.343 1

3 -9 -4 247.133 14.925 1

5 3 -9 348.212 18.047 3

5 -3 9 358.948 19.709 3

3 -9 5 373.261 19.354 1

3 -9 5 362.833 18.706 3

9 5 -3 347.603 19.115 3

3 9 -5 363.552 18.857 3

-3 -9 -5 403.005 20.177 1

-5 -3 -9 355.244 20.897 2

5 3 -9 389.909 21.264 2

-3 9 5 361.001 21.981 2

-9 5 3 346.890 22.352 2

9 -5 3 365.303 18.736 1

-9 -5 -3 372.505 20.568 1

-5 3 -9 374.245 22.927 2

9 -5 -3 387.978 19.521 1

3 9 5 403.417 20.254 3

-9 -5 3 404.575 21.269 1

3 -9 -5 351.572 20.001 1

-3 9 -5 419.876 23.377 2

5 3 9 376.281 20.214 3

5 -3 -9 368.444 21.841 2

9 5 3 336.496 20.683 3

5 -3 -9 356.320 18.518 1

-3 -9 5 332.442 20.588 1

-9 5 -3 356.189 22.842 2

-9 6 3 0.85283 4.83894 2

-3 9 6 5.80291 4.72755 2

-6 -3 -9 12.1440 4.2887 2

9 6 -3 9.65541 3.32011 3

6 -3 9 7.65156 3.84008 3

-9 -6 -3 9.72266 4.39725 1

3 -9 6 3.80132 3.09409 3

9 -6 3 9.60474 3.25448 1

6 3 -9 3.27526 2.34186 3

6 3 -9 6.03356 3.70915 2

-3 -9 -6 -2.5773 3.1796 1

3 9 -6 7.67275 3.03507 3

-3 -9 6 8.95312 4.55697 1

-9 6 -3 10.3681 4.9286 2

9 6 3 3.42717 3.93788 3

6 3 9 5.28160 3.82829 3

6 -3 -9 6.37459 2.74529 1

3 -9 -6 4.92321 3.39434 1

-9 -6 3 1.03505 4.10104 1

6 -3 -9 10.0098 4.2815 2

-3 9 -6 8.66159 4.66451 2

9 -6 -3 4.35520 2.94317 1

3 9 6 8.60677 4.01521 3

-6 3 -9 8.11448 4.93682 2

-9 7 3 400.875 25.157 2

-3 -9 -7 445.106 22.331 1

-9 -7 -3 425.947 23.303 1

7 3 -9 401.827 20.408 3

3 9 -7 366.985 20.887 3

7 -3 9 421.324 22.452 3

-3 9 7 422.072 25.275 2

9 -7 3 440.708 21.647 1

3 -9 7 396.546 21.546 3

-7 -3 -9 437.644 23.849 2

9 7 -3 396.016 21.603 3

9 7 3 390.032 21.962 3

7 3 9 418.977 22.178 3

7 -3 -9 391.968 20.116 1

-3 9 -7 437.859 25.246 2

-7 3 -9 414.027 24.750 2

-9 -7 3 365.519 22.349 1

9 -7 -3 354.551 20.465 1

-3 -9 7 338.788 21.642 1

3 -9 -7 383.995 21.103 1

-9 7 -3 394.842 24.458 2

3 9 7 431.224 21.649 3

-9 -8 -3 135.378 11.875 1

3 9 -8 142.925 9.823 3

-8 -3 -9 154.188 11.773 2

-3 9 8 143.843 13.386 2

-8 3 9 126.541 14.208 2

8 -3 9 143.101 11.292 3

8 3 -9 132.593 8.905 3

9 8 -3 142.622 10.056 3

9 -8 3 162.548 10.498 1

3 -9 8 134.818 10.585 3

-9 8 3 137.535 12.897 2

8 3 9 148.821 11.823 3

-3 9 -8 150.935 13.832 2

8 -3 -9 139.496 9.880 1

-8 3 -9 158.235 13.565 2

3 9 8 148.760 11.180 3

-8 -3 9 150.311 11.327 1

3 -9 -8 127.381 10.279 1

9 8 3 141.339 11.006 3

-9 -8 3 158.828 12.580 1

-9 8 -3 148.417 13.288 2

9 -8 -3 138.725 10.379 1

4 -9 0 105.537 8.371 1

0 4 9 106.938 7.648 3

-4 -9 0 105.402 8.985 1

0 -4 -9 96.6040 8.4324 2

0 4 -9 106.258 9.843 2

0 -4 9 103.006 7.633 3

-9 0 -4 107.027 8.830 2

9 0 -4 93.9546 7.2863 1

4 9 0 121.748 7.857 3

-9 0 4 95.2022 9.1162 2

9 0 4 110.429 7.319 1

9 0 4 120.661 9.529 3

-4 9 0 106.773 9.128 2

-9 1 4 410.846 25.264 2

9 1 -4 457.113 21.139 3

1 -4 9 438.271 22.178 3

4 9 -1 450.896 22.096 3

-1 -4 -9 441.168 24.220 2

-4 -9 -1 447.261 23.677 1

1 4 -9 459.540 25.638 2

-9 -1 -4 450.933 24.063 2

-1 4 9 449.054 21.972 3

9 -1 4 444.017 21.616 1

9 -1 4 489.406 23.925 3

-4 9 1 449.982 24.674 2

4 -9 1 434.079 23.039 1

9 1 -4 418.061 22.045 1

-4 9 -1 472.221 25.326 2

9 -1 -4 459.401 22.738 1

-1 4 -9 446.937 26.323 2

-9 -1 4 464.213 25.103 2

4 -9 -1 422.994 23.466 1

-4 -9 1 441.645 24.231 1

9 1 4 443.695 24.243 3

9 1 4 482.387 22.570 1

4 9 1 461.651 22.698 3

1 -4 -9 452.843 24.834 2

-9 1 -4 467.155 25.458 2

-1 -4 9 451.878 22.599 3

1 4 9 474.814 23.011 3

4 9 -2 49.3781 4.8550 3

-2 4 9 45.5847 4.7198 3

-9 2 4 45.5853 6.9040 2

-9 -2 -4 42.1070 5.2452 2

9 -2 4 50.3641 4.4593 1

9 2 -4 51.6097 4.5320 3

2 -4 9 47.7729 5.1503 3

9 -2 4 55.0559 6.3449 3

-4 -9 -2 44.2393 5.8022 1

-4 9 2 36.2061 5.6807 2

-2 -4 -9 41.8177 5.3525 2

9 2 -4 44.7824 4.7125 1

-9 -2 -4 47.4502 5.2861 1

2 4 -9 44.0180 6.1934 2

4 -9 2 40.5418 5.2195 1

-4 9 -2 63.0176 6.9339 2

4 -9 -2 30.5229 4.9687 1

-2 4 -9 38.1163 6.5315 2

-2 -4 9 49.0954 4.8983 3

9 2 4 42.9641 6.1435 3

2 -4 -9 39.1086 5.3972 2

4 9 2 54.0264 5.2843 3

-4 -9 2 44.3844 6.0241 1

9 -2 -4 57.6171 5.0488 1

-9 2 -4 55.9322 6.8500 2

2 4 9 44.9039 5.1798 3

9 3 -4 262.244 14.622 1

3 -4 9 263.461 14.992 3

-4 9 3 235.934 16.183 2

9 -3 4 266.323 13.886 1

4 9 -3 268.213 14.441 3

4 -9 3 264.746 15.144 1

-3 -4 -9 264.821 16.035 2

3 4 -9 245.706 13.801 3

-3 4 9 279.085 14.155 3

-4 -9 -3 288.146 16.080 1

-9 -3 -4 282.928 15.559 1

9 3 -4 264.704 14.176 3

3 4 -9 248.902 16.626 2

-9 3 4 268.491 17.782 2

-4 9 -3 318.400 17.830 2

-3 4 -9 236.242 17.263 2

9 -3 -4 251.253 14.320 1

4 9 3 272.849 15.124 3

-4 -9 3 252.617 15.962 1

4 -9 -3 228.736 14.865 1

3 4 9 271.871 15.160 3

3 -4 -9 274.468 16.411 2

-9 3 -4 279.254 17.657 2

9 3 4 255.118 16.186 3

4 4 -9 175.560 9.915 3

4 -4 9 168.227 11.092 3

4 4 -9 164.968 12.131 2

-4 -9 -4 189.698 11.875 1

-9 4 4 160.111 13.318 2

-4 -4 -9 159.434 11.536 2

9 -4 4 177.091 9.916 1

-9 -4 -4 153.495 11.458 1

4 -9 4 167.278 10.833 1

4 9 -4 169.561 10.408 3

9 4 -4 145.725 10.057 3

-4 9 4 162.287 12.423 2

9 -4 -4 160.252 10.322 1

4 -4 -9 151.490 11.839 2

4 9 4 178.463 11.533 3

-4 4 -9 184.969 13.926 2

-9 4 -4 167.346 13.520 2

-9 -4 4 157.257 11.489 1

4 -4 -9 175.509 9.919 1

4 4 9 170.642 11.360 3

4 -9 -4 157.665 11.117 1

-4 -9 4 178.143 12.378 1

-4 9 -4 191.009 13.855 2

9 4 4 172.375 12.361 3

9 5 -4 344.900 19.373 3

9 -5 4 392.031 19.214 1

-4 9 5 353.611 22.542 2

5 -4 9 373.651 20.367 3

4 -9 5 386.736 19.758 1

5 4 -9 356.955 18.796 3

-9 5 4 397.217 23.667 2

5 4 -9 382.726 21.469 2

4 9 -5 366.001 19.401 3

-4 -9 -5 424.381 20.789 1

-9 -5 -4 369.648 20.953 1

-5 -4 -9 371.638 21.423 2

9 -5 -4 381.746 19.927 1

5 -4 -9 389.977 19.335 1

-4 9 -5 412.171 23.910 2

9 5 4 377.389 21.693 3

-4 -9 5 364.926 21.418 1

-9 -5 4 392.239 21.489 1

-9 5 -4 362.756 23.534 2

4 9 5 387.646 20.747 3

-5 4 -9 390.467 23.884 2

4 -9 -5 345.921 20.288 1

5 4 9 400.877 21.100 3

5 -4 -9 370.554 22.189 2

-6 -4 -9 35.1544 5.4781 2

6 -4 9 42.5019 5.9024 3

9 6 -4 45.3468 5.2443 3

9 -6 4 37.7653 4.7477 1

-9 6 4 35.1595 7.1837 2

4 9 -6 37.0913 4.7185 3

-4 9 6 31.1671 6.8506 2

-4 -9 -6 34.8287 5.2650 1

-9 -6 -4 34.5165 5.9172 1

4 -9 6 26.8832 4.5878 3

6 4 -9 34.5428 4.3019 3

-4 -9 6 37.3806 6.3282 1

-9 -6 4 38.2250 6.3979 1

4 -9 -6 30.4252 4.9955 1

-4 9 -6 29.2974 6.6174 2

6 4 9 20.4529 5.1210 3

9 6 4 31.1366 6.1041 3

4 9 6 32.2915 5.4434 3

-9 6 -4 33.2545 7.1268 2

9 -6 -4 25.7638 4.4275 1

-6 4 -9 35.9679 7.1640 2

6 -4 -9 34.4787 5.9655 2

6 -4 -9 36.2038 4.3798 1

-9 -7 -4 110.255 10.108 1

-9 7 4 130.217 12.131 2

7 4 -9 117.522 8.165 3

9 -7 4 123.829 8.845 1

-4 9 7 108.328 11.467 2

-7 -4 -9 119.683 9.911 2

4 -9 7 107.033 8.891 3

-7 4 9 116.481 13.304 2

-4 -9 -7 134.830 9.891 1

4 9 -7 108.917 8.520 3

7 -4 9 114.340 9.895 3

9 7 -4 111.141 8.747 3

4 -9 -7 107.756 8.810 1

4 9 7 126.428 9.820 3

-4 9 -7 133.710 12.285 2

-4 -9 7 82.0630 9.1985 1

7 4 9 116.536 10.037 3

-7 4 -9 96.3084 11.1140 2

-9 -7 4 87.2184 9.8149 1

9 -7 -4 105.371 8.525 1

7 -4 -9 123.201 8.446 1

9 7 4 101.702 9.593 3

-9 7 -4 102.241 11.145 2

8 -4 9 217.122 14.211 3

-9 -8 -4 223.394 14.935 1

8 4 -9 202.195 12.074 3

9 -8 4 217.217 13.151 1

-8 4 9 205.019 18.827 2

-4 9 8 209.088 16.598 2

4 9 -8 205.333 12.725 3

9 8 -4 204.537 12.991 3

4 -9 8 187.366 13.158 3

-9 8 4 204.522 16.302 2

8 -4 -9 214.539 13.007 1

4 9 8 228.981 14.438 3

-4 9 -8 243.486 17.819 2

9 -8 -4 175.368 13.004 1

8 4 9 205.349 14.567 3

-8 4 -9 246.431 17.557 2

-9 -8 4 211.620 15.096 1

4 -9 -8 217.342 13.799 1

-9 8 -4 207.402 16.673 2

9 8 4 201.924 14.217 3

0 -5 -9 569.730 30.000 2

0 5 9 555.283 27.781 3

9 0 5 625.269 29.633 3

9 0 -5 543.261 27.738 1

0 -5 9 568.120 27.953 3

5 -9 0 547.079 28.595 1

9 0 5 635.313 27.525 1

-9 0 -5 546.921 30.405 2

-9 0 5 559.913 31.448 2

-5 9 0 576.344 30.877 2

5 9 0 564.891 27.789 3

0 5 -9 575.651 31.898 2

-5 -9 0 586.732 29.492 1

9 1 -5 375.145 20.224 1

-9 -1 -5 380.541 22.237 2

-1 -5 -9 394.849 21.964 2

5 -9 1 392.324 21.066 1

-9 -1 -5 417.515 20.583 1

1 5 -9 377.366 23.399 2

-9 1 5 374.577 23.718 2

9 1 -5 386.029 19.001 3

-5 9 1 405.697 23.132 2

1 -5 9 408.164 20.687 3

-5 -9 -1 437.705 22.258 1

-1 5 9 402.009 20.328 3

5 9 -1 420.404 20.416 3

9 -1 5 422.977 22.019 3

9 -1 5 413.733 19.821 1

-5 -9 1 373.068 21.544 1

-5 9 -1 423.982 23.038 2

-9 -1 5 391.436 22.890 2

-9 1 -5 404.714 22.970 2

5 9 1 375.966 20.231 3

1 5 9 379.956 20.440 3

5 -9 -1 408.832 21.206 1

-1 -5 9 427.756 20.598 3

9 1 5 423.584 22.236 3

9 -1 -5 385.663 20.139 1

-1 5 -9 389.316 23.704 2

1 -5 -9 390.576 21.960 2

-9 -2 -5 113.571 9.045 2

2 5 -9 115.796 10.206 2

9 2 -5 115.822 7.442 3

-5 9 2 104.805 9.762 2

-9 -2 -5 115.816 8.637 1

-2 -5 -9 115.816 8.902 2

9 2 -5 104.441 7.866 1

2 -5 9 106.167 8.276 3

2 5 -9 121.378 7.866 3

5 -9 2 110.798 8.661 1

-2 5 9 120.321 8.115 3

-5 -9 -2 125.620 9.749 1

5 9 -2 116.142 8.137 3

9 -2 5 143.676 10.321 3

-9 2 5 106.284 10.744 2

9 -2 5 119.836 7.516 1

-5 9 -2 118.118 10.031 2

5 -9 -2 87.0987 8.2046 1

2 5 9 132.757 8.947 3

2 -5 -9 115.213 9.157 2

9 2 5 118.929 9.887 3

9 -2 -5 102.906 7.776 1

5 9 2 117.771 8.439 3

-2 5 -9 117.439 10.890 2

-5 -9 2 114.893 9.707 1

-9 2 -5 118.767 10.287 2

-5 9 3 340.273 21.888 2

-9 3 5 375.663 23.557 2

9 -3 5 454.318 21.321 3

3 -5 9 367.845 19.807 3

9 -3 5 361.526 18.452 1

-5 -9 -3 417.884 21.016 1

-3 -5 -9 356.487 20.663 2

5 9 -3 375.637 19.231 3

3 5 -9 379.116 22.019 2

-9 -3 -5 380.539 20.224 1

9 3 -5 354.626 18.585 3

3 5 -9 382.838 18.907 3

5 -9 3 375.813 19.792 1

9 3 -5 332.976 19.207 1

-9 3 -5 362.250 22.445 2

9 -3 -5 355.516 19.072 1

-3 5 -9 375.955 23.257 2

3 -5 -9 377.411 21.392 2

3 5 9 379.963 20.097 3

-5 9 -3 396.951 22.626 2

5 9 3 390.391 20.067 3

5 -9 -3 345.514 19.821 1

9 3 5 373.697 21.118 3

-5 -9 3 355.583 20.740 1

-4 -5 -9 366.856 21.253 2

5 9 -4 366.456 19.679 3

4 -5 9 382.485 20.512 3

9 -4 5 401.420 19.228 1

9 4 -5 389.394 19.647 3

-5 9 4 371.012 22.812 2

-5 -9 -4 444.929 21.644 1

5 -9 4 403.508 20.334 1

-9 -4 -5 366.111 20.776 1

4 5 -9 388.215 19.456 3

4 5 -9 368.309 21.883 2

-9 4 5 338.411 23.899 2

-9 -4 5 374.316 20.837 1

-4 5 -9 415.163 24.159 2

4 -5 -9 342.483 21.392 2

5 -9 -4 354.204 20.101 1

5 9 4 386.666 20.510 3

4 -5 -9 386.931 19.100 1

-5 -9 4 354.523 20.914 1

-5 9 -4 383.113 23.266 2

-9 4 -5 380.296 23.353 2

4 5 9 403.767 20.860 3

9 4 5 362.193 21.271 3

9 -4 -5 363.975 19.479 1

-9 5 5 134.517 12.808 2

5 5 -9 125.993 8.547 3

5 -5 9 140.200 10.155 3

-5 -5 -9 124.389 9.801 2

-9 -5 -5 113.364 9.899 1

5 9 -5 122.778 8.842 3

-5 9 5 129.248 11.765 2

5 -9 5 139.241 9.268 1

9 5 -5 115.918 8.864 3

-5 -9 -5 132.339 10.378 1

-5 -9 5 130.058 10.886 1

5 -5 -9 122.165 8.706 1

-5 9 -5 149.384 12.749 2

5 -5 -9 125.738 10.628 2

9 5 5 125.116 10.851 3

5 5 9 130.798 10.431 3

-9 -5 5 142.427 11.004 1

5 -9 -5 141.385 9.943 1

-9 5 -5 147.552 13.054 2

5 9 5 140.985 10.330 3

9 -5 -5 117.237 9.050 1

-5 5 -9 124.299 12.122 2

-6 -5 -9 94.2362 8.7858 2

-5 9 6 109.357 10.917 2

-9 6 5 94.5731 11.0299 2

9 6 -5 97.1814 7.9785 3

5 9 -6 97.5298 7.8276 3

-9 -6 -5 106.998 9.334 1

6 5 -9 96.8555 7.4904 3

-5 -9 -6 107.085 8.877 1

6 -5 9 93.1490 8.7690 3

-6 5 9 87.3941 11.4443 2

-9 6 -5 87.5619 10.5286 2

9 -6 -5 102.898 8.172 1

-5 9 -6 104.019 10.857 2

-5 -9 6 101.121 9.447 1

6 -5 -9 85.5718 7.5829 1

-6 5 -9 105.003 11.275 2

5 9 6 88.4467 8.7518 3

9 6 5 101.937 9.582 3

5 -9 -6 94.2950 8.3246 1

-9 -6 5 111.437 9.837 1

6 5 9 113.419 9.606 3

-5 9 7 295.396 22.123 2

7 -5 9 334.237 19.517 3

-9 7 5 368.414 23.055 2

-5 -9 -7 408.988 19.624 1

-7 5 9 313.418 23.721 2

5 9 -7 326.430 18.235 3

9 7 -5 316.501 18.376 3

7 5 -9 329.914 17.899 3

-9 -7 -5 360.986 20.083 1

-5 -9 7 313.494 19.716 1

-7 5 -9 366.431 23.343 2

-5 9 -7 325.049 22.216 2

-9 7 -5 330.685 22.480 2

7 -5 -9 331.158 18.095 1

9 7 5 290.588 19.323 3

5 9 7 327.194 19.272 3

9 -7 -5 327.765 18.379 1

-9 -7 5 350.478 20.464 1

7 5 9 345.661 19.827 3

5 -9 -7 344.553 18.966 1

-5 9 8 180.669 16.166 2

5 9 -8 185.759 11.863 3

8 5 -9 179.423 11.368 3

-9 8 5 181.113 15.642 2

9 8 -5 168.249 11.789 3

-9 -8 -5 183.856 13.383 1

8 -5 9 203.304 13.741 3

-8 5 9 191.391 17.802 2

5 -9 -8 176.798 12.467 1

-8 5 -9 186.064 15.950 2

-9 8 -5 191.119 15.875 2

-9 -8 5 171.188 13.944 1

-5 9 -8 188.512 15.903 2

8 5 9 179.494 13.428 3

9 8 5 181.912 13.351 3

5 9 8 178.312 12.948 3

9 -8 -5 171.640 12.072 1

8 -5 -9 172.434 11.754 1

-6 9 0 21.8255 5.1905 2

0 -6 -9 18.1509 3.9696 2

-9 0 -6 18.8270 4.7920 2

0 -6 9 21.9222 4.1236 3

-6 -9 0 16.7303 4.7798 1

-9 0 6 17.8048 5.8407 2

0 6 -9 15.8298 5.3330 2

0 6 9 17.2535 4.0137 3

9 0 6 12.8601 4.8143 3

9 0 -6 18.3600 3.4716 1

6 9 0 19.6635 3.8383 3

6 -9 0 21.2694 4.2991 1

6 -9 1 33.6824 4.9258 1

1 -6 9 33.1366 4.8457 3

-9 1 6 30.6023 7.1990 2

-1 -6 -9 44.8050 5.1910 2

-6 9 1 36.4692 6.0845 2

-6 -9 -1 36.5104 5.9671 1

-9 -1 -6 36.6197 5.6543 2

9 1 -6 31.0900 3.4580 3

-1 6 9 41.2267 4.9283 3

1 6 -9 44.6351 6.5508 2

6 9 -1 35.8461 4.5947 3

9 -1 6 39.7183 6.1201 3

9 1 -6 29.8053 4.2443 1

9 1 6 35.8442 5.9888 3

-9 1 -6 39.2440 6.0825 2

6 -9 -1 41.0153 5.2865 1

-1 -6 9 32.5877 4.7790 3

1 6 9 36.7700 5.2685 3

9 -1 -6 34.2362 4.3450 1

-6 -9 1 32.4650 5.7123 1

6 9 1 38.9552 4.8561 3

-1 6 -9 32.0345 6.4293 2

-9 -1 6 35.8177 6.9108 2

-6 9 -1 43.7152 6.4436 2

1 -6 -9 37.6147 5.2167 2

9 2 -6 43.4918 4.1172 3

2 6 -9 34.4229 4.4215 3

2 6 -9 37.0756 6.2139 2

-6 -9 -2 46.7633 6.2940 1

6 9 -2 33.8483 4.5597 3

-2 -6 -9 39.1094 5.0633 2

9 -2 6 41.8641 6.2478 3

-6 9 2 40.8791 6.3546 2

6 -9 2 45.7004 5.4273 1

-9 2 6 24.4425 7.0683 2

9 2 -6 26.2618 4.1556 1

-9 -2 -6 35.2178 5.3428 2

2 -6 9 43.0493 5.2660 3

2 6 9 37.1514 5.3954 3

2 -6 -9 38.8418 5.3986 2

6 9 2 44.1358 5.2486 3

-6 9 -2 35.6810 6.3250 2

9 -2 -6 44.8848 4.8232 1

-6 -9 2 29.6168 5.7274 1

-2 6 -9 40.6983 7.1513 2

-9 2 -6 44.7562 6.5877 2

9 2 6 35.4784 6.0692 3

6 -9 -2 32.2258 4.9292 1

9 -3 6 8.55862 4.48870 3

9 3 -6 3.96850 2.53308 3

6 -9 3 6.51501 3.56132 1

3 -6 9 7.14009 3.61942 3

3 6 -9 6.04532 2.96603 3

-6 -9 -3 11.7453 4.5166 1

6 9 -3 4.00250 2.58958 3

3 6 -9 1.16263 3.77726 2

-3 -6 -9 4.06724 3.13873 2

-9 3 6 14.2373 6.5468 2

-6 9 3 5.85111 4.26712 2

9 3 6 3.00777 3.86330 3

9 -3 -6 3.46287 2.76156 1

3 6 9 5.34355 3.72327 3

-3 6 -9 3.82156 4.64175 2

-6 9 -3 4.28044 4.70509 2

-9 -3 6 9.47090 3.99880 1

3 -6 -9 2.58332 3.15595 2

6 9 3 4.38925 3.15160 3

-9 3 -6 10.1839 4.9510 2

-6 -9 3 -0.3263 4.0781 1

6 -9 -3 4.01029 3.15656 1

9 4 -6 36.2451 4.3859 3

-9 4 6 34.7501 7.9424 2

6 9 -4 31.6179 4.4566 3

4 6 -9 29.3455 4.2718 3

4 -6 9 29.6901 5.2104 3

-6 -9 -4 30.2583 5.6902 1

-4 -6 -9 24.3075 4.6509 2

6 -9 4 37.5956 4.9796 1

-6 9 4 28.4763 6.1948 2

9 -4 -6 35.3461 4.7899 1

-4 6 -9 31.9539 7.1977 2

-9 4 -6 38.8391 7.1156 2

6 9 4 34.7947 5.4560 3

4 6 9 26.4191 5.3347 3

4 -6 -9 32.6947 4.3661 1

-6 9 -4 29.6181 6.7253 2

9 4 6 36.3929 6.1734 3

6 -9 -4 36.6816 5.1529 1

-9 -4 6 32.5931 5.7651 1

-6 -9 4 28.1331 5.7567 1

4 -6 -9 31.6015 5.5208 2

-5 6 9 103.921 11.399 2

6 -9 5 106.392 8.361 1

6 9 -5 99.4440 7.9475 3

5 -6 9 87.4096 8.5021 3

9 5 -6 99.8330 7.8000 3

5 6 -9 100.812 7.731 3

-9 5 6 102.305 11.665 2

-6 -9 -5 120.652 9.593 1

-6 9 5 93.7197 10.4139 2

-5 -6 -9 98.0556 8.4060 2

5 6 9 91.8995 8.6890 3

5 -6 -9 95.8541 7.7034 1

9 5 6 101.902 9.427 3

9 -5 -6 96.8078 7.9539 1

-9 5 -6 108.027 11.264 2

-5 6 -9 91.4608 10.9055 2

6 -9 -5 82.5879 8.0444 1

-9 -5 6 89.9249 9.0152 1

-6 9 -5 109.595 11.148 2

-6 -9 5 86.2456 8.9777 1

6 9 5 102.166 8.962 3

6 9 -6 48.0880 5.6530 3

9 6 -6 39.9685 5.4256 3

-6 9 6 51.2286 8.2909 2

-6 6 9 67.1682 10.1672 2

-9 -6 -6 42.5873 6.4796 1

6 -6 9 55.3808 7.1401 3

6 6 -9 47.1914 5.4638 3

-6 -9 -6 64.7182 6.9923 1

-9 6 6 33.6673 8.6416 2

6 -6 -9 48.1581 5.6626 1

6 9 6 47.7893 6.8376 3

9 -6 -6 46.1251 5.8099 1

-6 -9 6 37.8909 6.8643 1

-9 6 -6 45.0237 8.3733 2

-9 -6 6 58.6162 7.6997 1

6 6 9 50.6563 7.0815 3

9 6 6 32.9328 6.5650 3

-6 6 -9 54.5858 8.9204 2

-6 9 -6 46.0366 8.2984 2

6 -9 -6 61.1906 6.8023 1

-9 -7 -6 41.4477 6.6789 1

9 7 -6 28.5069 4.9531 3

7 6 -9 37.4496 5.0443 3

-7 6 9 40.6195 9.2875 2

-6 9 7 35.9657 7.8006 2

-9 7 6 23.2807 7.8162 2

7 -6 9 28.1719 6.2586 3

6 9 -7 35.4595 5.1079 3

9 7 6 30.9926 6.4302 3

6 9 7 31.2390 6.0856 3

7 -6 -9 29.1504 5.1133 1

-6 -9 7 27.1113 6.5889 1

6 -9 -7 33.3675 5.6153 1

-6 9 -7 34.7193 7.8550 2

9 -7 -6 30.1589 5.1937 1

-9 7 -6 32.8773 7.7734 2

-7 6 -9 39.2826 8.4716 2

-9 -7 6 23.4446 6.4002 1

7 6 9 39.5190 6.5340 3

-6 9 8 26.9642 8.0253 2

8 6 -9 39.0314 5.2079 3

9 8 -6 33.5826 5.2984 3

6 9 -8 34.9781 5.1801 3

-8 6 9 39.6050 10.0197 2

-9 8 6 34.9911 8.4104 2

-9 -8 6 36.9216 7.2087 1

6 9 8 39.3207 6.4694 3

-9 8 -6 29.5550 7.9681 2

8 6 9 28.2938 6.1743 3

6 -9 -8 23.6411 5.1636 1

9 8 6 37.7486 6.7533 3

8 -6 -9 26.1161 5.0911 1

9 -8 -6 22.7041 5.2051 1

-6 9 -8 31.9770 8.0168 2

-8 6 -9 39.4438 8.6143 2

9 0 7 146.263 11.151 3

-9 0 7 125.426 12.202 2

0 -7 -9 145.958 10.070 2

0 -7 9 123.769 9.551 3

7 9 0 126.882 9.215 3

0 7 9 121.784 9.854 3

7 -9 0 123.975 9.797 1

-7 -9 0 128.698 10.737 1

-9 0 -7 122.945 10.932 2

0 7 -9 138.538 11.998 2

-7 9 0 136.815 11.692 2

9 0 -7 126.125 8.781 1

-9 1 7 316.546 23.098 2

1 7 -9 350.040 22.038 2

-7 -9 -1 393.352 20.940 1

7 9 -1 348.491 18.934 3

1 -7 9 357.299 19.594 3

9 -1 7 407.201 20.997 3

-9 -1 -7 360.354 21.285 2

7 -9 1 362.001 19.644 1

-1 -7 -9 363.430 20.369 2

9 1 -7 349.224 18.867 1

-7 9 1 356.297 21.938 2

1 7 -9 363.189 18.758 3

-1 7 -9 330.094 22.405 2

-7 9 -1 361.932 22.019 2

1 7 9 356.722 19.495 3

-9 1 -7 366.197 21.961 2

-7 -9 1 366.066 20.642 1

-9 -1 7 346.989 22.471 2

7 9 1 351.766 19.051 3

9 1 7 390.865 20.917 3

9 -1 -7 335.112 18.609 1

1 -7 -9 378.724 20.570 2

7 -9 -1 344.569 19.503 1

7 9 -2 35.7136 4.7931 3

9 -2 7 58.5999 7.1225 3

-7 9 2 37.4668 6.8891 2

-7 -9 -2 28.7672 5.8932 1

-9 2 7 30.5872 7.6201 2

9 2 -7 40.9307 4.8611 1

7 -9 2 40.1803 5.4923 1

-2 -7 -9 38.2110 5.0523 2

-9 -2 -7 37.6340 5.9205 2

2 7 -9 38.3762 4.7023 3

9 2 -7 37.1861 4.0031 3

2 -7 9 33.8052 5.2695 3

2 7 -9 33.2898 6.1613 2

-7 -9 2 37.2788 6.4448 1

7 -9 -2 36.2884 5.3714 1

9 2 7 44.8032 6.6794 3

-2 7 -9 38.8953 7.3813 2

-9 2 -7 31.9715 6.4471 2

9 -2 -7 30.8094 4.5708 1

7 9 2 38.0053 5.3328 3

-7 9 -2 35.8954 6.6579 2

2 -7 -9 50.5996 6.0039 2

2 7 9 34.6992 5.6090 3

9 -3 7 424.636 22.292 3

3 -7 9 397.874 21.384 3

-3 -7 -9 400.931 21.920 2

-7 -9 -3 421.066 22.640 1

7 -9 3 428.909 21.510 1

-7 9 3 381.297 24.035 2

9 3 -7 403.358 19.921 3

-9 3 7 390.735 26.381 2

3 7 -9 393.347 20.594 3

7 9 -3 364.043 20.454 3

3 -7 -9 387.804 20.488 1

7 -9 -3 351.846 21.042 1

-9 -3 7 381.550 21.633 1

9 3 7 376.871 22.298 3

-3 7 -9 392.120 25.154 2

3 7 9 376.026 21.436 3

3 -7 -9 393.207 22.773 2

-9 3 -7 417.279 24.605 2

-7 -9 3 402.492 22.516 1

9 -3 -7 424.191 20.848 1

7 9 3 422.856 21.599 3

-7 9 -3 420.956 24.518 2

4 7 -9 108.265 8.425 3

-7 -9 -4 122.682 10.215 1

-7 9 4 104.054 10.864 2

-4 7 9 118.431 12.011 2

-4 -7 -9 121.236 9.023 2

9 4 -7 106.658 8.021 3

4 -7 9 109.534 9.375 3

-9 4 7 130.329 13.578 2

7 9 -4 102.968 8.319 3

7 -9 4 131.291 9.544 1

4 7 9 113.808 9.939 3

-4 7 -9 111.540 11.846 2

9 -4 -7 117.447 8.556 1

-7 9 -4 119.299 11.378 2

-9 4 -7 104.971 11.258 2

7 9 4 120.688 9.823 3

-7 -9 4 109.349 10.037 1

7 -9 -4 96.2304 8.8883 1

9 4 7 105.496 9.983 3

4 -7 -9 112.532 8.573 1

-9 -4 7 106.751 9.632 1

5 -7 9 358.063 19.613 3

-9 5 7 359.165 24.411 2

7 9 -5 307.337 18.194 3

-7 9 5 316.136 21.916 2

-7 -9 -5 364.383 19.970 1

-5 7 9 358.557 23.816 2

5 7 -9 334.747 18.309 3

9 5 -7 346.282 18.201 3

9 -5 -7 349.993 18.503 1

7 -9 -5 341.923 19.124 1

5 -7 -9 322.743 18.188 1

-5 7 -9 337.987 23.135 2

5 7 9 338.242 19.598 3

-9 -5 7 305.867 19.590 1

9 5 7 319.678 19.854 3

7 9 5 317.776 19.301 3

-7 -9 5 317.361 19.770 1

-7 9 -5 349.174 22.589 2

-9 5 -7 380.322 23.286 2

-6 7 9 26.0306 7.7081 2

9 6 -7 35.1131 4.8536 3

6 -7 9 25.3168 6.2554 3

6 7 -9 31.2305 4.8582 3

-9 6 7 32.0274 8.9038 2

-7 9 6 39.4814 8.0902 2

-7 -9 -6 34.7505 6.2337 1

7 9 -6 33.8133 5.0422 3

9 6 7 30.4970 6.3555 3

-9 6 -7 32.1422 7.8596 2

-9 -6 7 31.4595 6.5485 1

7 -9 -6 35.0784 5.7546 1

6 7 9 33.4474 6.2720 3

-7 -9 6 34.6228 6.7917 1

7 9 6 37.3427 6.4375 3

6 -7 -9 38.6168 5.5121 1

9 -6 -7 30.2408 5.2096 1

-7 9 -6 31.8867 7.5109 2

-6 7 -9 32.8434 8.2858 2

7 7 -9 188.585 12.213 3

-7 9 7 194.951 16.638 2

-7 7 9 194.513 17.686 2

-9 7 7 196.487 17.407 2

7 9 -7 203.079 12.423 3

9 7 -7 177.314 11.972 3

7 9 7 183.632 13.814 3

-7 7 -9 194.387 17.275 2

9 7 7 203.089 14.395 3

7 -7 -9 189.762 12.684 1

-9 7 -7 206.730 16.862 2

7 -9 -7 172.485 12.872 1

-9 -7 7 201.869 14.575 1

-7 -9 7 196.017 14.328 1

9 -7 -7 190.808 12.885 1

-7 9 -7 181.944 16.214 2

7 7 9 187.849 13.883 3

8 7 -9 53.9734 6.4211 3

7 9 -8 61.1155 6.6189 3

-8 7 9 66.8970 11.6050 2

-7 9 8 66.4928 10.4813 2

-9 8 7 67.8540 10.4817 2

9 8 -7 50.9196 6.2535 3

9 8 7 75.0358 8.4894 3

-9 -8 7 62.0205 8.8733 1

-9 8 -7 52.7174 9.8575 2

-7 9 -8 72.9778 10.4776 2

8 7 9 61.8593 8.3546 3

-8 7 -9 59.5609 10.3774 2

7 -9 -8 69.9782 7.8303 1

9 -8 -7 71.8879 7.8347 1

7 9 8 65.7365 8.2626 3

8 -7 -9 62.0794 7.3097 1

8 -9 0 232.732 14.236 1

-8 -9 0 235.243 15.457 1

8 9 0 223.188 13.703 3

0 8 9 217.846 14.037 3

-8 9 0 219.582 16.145 2

-9 0 -8 216.720 15.629 2

0 -8 -9 260.860 14.767 2

9 0 8 240.369 15.476 3

-9 0 8 251.566 18.156 2

0 8 -9 224.176 16.540 2

9 0 -8 205.632 13.009 1

0 -8 9 217.645 14.130 3

-9 -1 -8 171.667 13.151 2

8 -9 1 172.390 12.057 1

-8 -9 -1 163.865 13.082 1

-1 -8 -9 196.783 12.339 2

-9 1 8 182.707 15.994 2

8 9 -1 177.942 11.563 3

9 -1 8 190.554 13.402 3

9 1 -8 189.590 11.312 1

-8 9 1 178.733 14.230 2

1 -8 9 168.033 12.151 3

1 8 -9 192.036 11.438 3

9 1 8 187.773 13.375 3

-9 -1 8 158.217 14.683 2

8 9 1 168.244 11.512 3

-8 9 -1 200.764 14.414 2

1 8 9 186.224 12.373 3

-8 -9 1 176.632 13.140 1

8 -9 -1 184.953 12.216 1

-9 1 -8 181.729 13.881 2

1 -8 -9 184.291 12.340 2

-1 8 -9 154.864 14.190 2

9 -1 -8 170.081 11.049 1

9 -2 8 54.7006 7.4048 3

8 -9 2 57.2975 6.4196 1

-9 2 8 52.8264 9.5458 2

-8 -9 -2 41.8502 6.9135 1

9 2 -8 43.3057 5.2211 1

-2 -8 -9 54.0953 5.9819 2

2 8 -9 53.3126 5.5958 3

8 9 -2 40.8609 5.4013 3

-8 9 2 51.8100 8.0622 2

2 -8 9 52.1616 6.4944 3

-9 -2 -8 44.7607 6.6465 2

9 2 -8 47.9100 4.6105 3

8 -9 -2 45.4489 6.0337 1

2 8 9 52.9734 6.7384 3

9 2 8 51.1771 7.3350 3

8 9 2 39.1325 5.7293 3

9 -2 -8 48.3849 5.3387 1

-8 -9 2 40.6026 6.8560 1

-2 8 -9 35.5443 7.4506 2

2 -8 -9 50.6888 6.3982 2

-8 9 -2 55.0999 7.9126 2

-9 2 -8 60.2405 8.3402 2

3 -8 9 136.204 10.432 3

-9 3 8 109.627 13.654 2

-8 9 3 144.646 12.689 2

9 -3 8 144.095 11.561 3

-3 8 9 127.612 12.654 2

3 8 -9 145.236 9.726 3

8 -9 3 142.227 10.145 1

8 9 -3 126.651 9.461 3

9 3 -8 142.152 8.902 3

-8 -9 -3 152.941 11.752 1

-3 8 -9 123.486 12.943 2

-9 -3 8 134.182 11.082 1

-8 -9 3 139.369 11.837 1

8 9 3 151.348 10.886 3

-8 9 -3 148.430 12.973 2

3 8 9 138.690 11.105 3

-9 3 -8 142.364 13.010 2

9 3 8 161.714 12.131 3

8 -9 -3 133.716 10.400 1

9 -3 -8 146.608 10.030 1

3 -8 -9 142.097 10.155 1

8 9 -4 203.836 13.195 3

-9 4 8 239.592 19.386 2

8 -9 4 244.134 14.051 1

-8 -9 -4 212.879 15.041 1

4 8 -9 206.360 13.088 3

-8 9 4 226.412 16.789 2

9 4 -8 209.617 12.564 3

4 -8 9 223.202 14.323 3

-4 8 9 234.864 17.822 2

4 -8 -9 207.641 13.542 1

-9 -4 8 216.680 14.734 1

9 4 8 239.061 15.411 3

-8 -9 4 232.876 15.361 1

9 -4 -8 202.963 13.129 1

-9 4 -8 239.028 17.531 2

8 9 4 184.750 13.887 3

-8 9 -4 234.945 17.147 2

8 -9 -4 206.260 13.735 1

4 8 9 212.039 14.342 3

-4 8 -9 232.501 17.791 2

-8 9 5 190.818 15.651 2

-9 5 8 170.282 17.253 2

5 8 -9 180.930 11.552 3

-5 8 9 138.800 14.929 2

9 5 -8 173.400 11.191 3

5 -8 9 173.240 12.753 3

8 9 -5 178.822 11.702 3

-8 -9 -5 204.886 13.579 1

-8 -9 5 172.110 13.755 1

5 -8 -9 161.005 12.088 1

8 -9 -5 172.275 12.512 1

9 -5 -8 185.393 12.067 1

8 9 5 177.014 13.120 3

5 8 9 198.896 13.499 3

-5 8 -9 183.387 16.205 2

9 5 8 200.991 14.038 3

-8 9 -5 205.524 16.145 2

-9 -5 8 186.367 13.884 1

-9 5 -8 206.570 16.419 2

-9 6 8 50.3900 10.4558 2

6 8 -9 34.4431 5.2666 3

-8 -9 -6 36.0690 6.7371 1

8 9 -6 44.5556 5.6451 3

-8 9 6 38.9294 8.4058 2

9 6 -8 34.4004 5.2179 3

-6 8 9 35.6777 9.1460 2

9 -6 -8 48.1787 6.2236 1

-9 6 -8 43.2049 8.7181 2

-9 -6 8 34.0211 7.0868 1

6 8 9 35.7337 6.5483 3

8 -9 -6 30.7757 5.5807 1

-6 8 -9 37.1448 8.6267 2

-8 9 -6 36.9068 8.3414 2

-8 -9 6 31.8448 7.0291 1

6 -8 -9 40.3865 5.9726 1

9 6 8 46.4685 7.2042 3

8 9 6 22.8539 6.0882 3

-9 7 8 65.2142 11.3565 2

8 9 -7 64.3403 6.7681 3

-8 9 7 64.8769 10.3493 2

7 8 -9 64.2664 6.7908 3

-7 8 9 50.2016 10.3345 2

9 7 -8 75.4765 7.0959 3

8 -9 -7 60.3211 7.4850 1

-9 7 -8 37.6583 9.0880 2

-8 -9 7 60.5532 8.4438 1

-8 9 -7 56.5523 9.7648 2

9 -7 -8 67.2828 7.5450 1

-7 8 -9 65.4374 10.4622 2

7 8 9 68.4869 8.3143 3

8 9 7 50.2278 7.5631 3

-9 -7 8 57.4046 8.7088 1

9 7 8 60.7823 8.0488 3

7 -8 -9 69.8924 7.5090 1

-9 8 8 206.251 18.100 2

8 9 -8 178.774 12.128 3

-8 8 9 191.825 17.792 2

-8 9 8 165.483 16.528 2

8 8 -9 191.295 12.358 3

9 8 -8 164.616 11.868 3

-9 8 -8 181.908 17.000 2

-8 9 -8 178.962 16.988 2

8 9 8 204.080 14.623 3

-8 8 -9 196.900 17.645 2

8 -8 -9 198.708 13.675 1

8 8 9 194.262 14.450 3

-9 -8 8 194.019 15.025 1

8 -9 -8 180.651 13.553 1

9 -8 -8 196.542 13.757 1

0 -9 9 291.286 18.151 3

-9 -9 0 308.054 19.486 1

9 0 9 327.481 19.408 3

9 0 -9 292.640 17.051 1

-9 0 9 304.809 22.048 2

-9 9 0 294.594 20.378 2

0 -9 -9 343.690 18.922 2

9 9 0 320.361 17.878 3

-9 0 -9 333.970 20.406 2

9 -9 0 324.945 18.232 1

1 9 -9 112.307 8.527 3

1 -9 9 102.369 9.222 3

-9 -9 -1 101.301 10.075 1

-1 9 9 98.0249 10.3925 2

9 9 -1 93.5091 8.4774 3

-9 -1 -9 114.320 10.428 2

9 1 -9 106.322 8.285 1

-9 1 9 103.612 12.793 2

9 -9 1 104.855 9.097 1

-9 9 1 109.594 11.264 2

-1 -9 -9 120.078 9.299 2

9 -1 9 121.543 10.526 3

-9 -9 1 85.1261 9.7934 1

9 -9 -1 107.356 9.277 1

-9 9 -1 99.0470 10.7553 2

9 -1 -9 99.2503 8.0679 1

9 9 1 115.288 9.086 3

-1 9 -9 123.338 11.874 2

9 1 9 129.552 10.982 3

-9 1 -9 106.811 10.509 2

9 9 -2 153.723 10.676 3

-9 9 2 129.949 12.837 2

9 -9 2 163.112 11.006 1

-9 -2 -9 164.630 12.375 2

9 -2 9 161.457 12.464 3

2 -9 9 145.232 11.332 3

2 9 -9 136.405 10.040 3

-9 2 9 123.702 14.486 2

-2 9 9 143.889 13.327 2

-9 -9 -2 159.703 12.493 1

-9 9 -2 151.544 13.672 2

-9 -9 2 137.125 12.270 1

-9 -2 9 154.992 11.688 1

9 -2 -9 146.350 10.207 1

-9 2 -9 149.922 13.276 2

-2 9 -9 143.511 13.740 2

9 9 2 146.090 10.911 3

9 -9 -2 157.940 11.178 1

9 2 9 162.098 12.587 3

2 9 9 150.921 11.442 3

3 9 -9 229.496 13.006 3

3 -9 9 182.434 13.364 3

-9 -9 -3 229.793 15.090 1

9 3 -9 183.359 11.657 3

9 -9 3 233.339 13.903 1

9 9 -3 173.594 12.476 3

9 -3 9 250.841 15.332 3

-3 9 9 219.453 16.780 2

-9 3 9 192.209 18.256 2

-9 9 3 195.757 16.144 2

9 -3 -9 197.636 12.606 1

-9 -9 3 224.270 15.336 1

3 9 9 202.930 13.862 3

9 -9 -3 195.946 13.260 1

-3 9 -9 231.230 17.446 2

-9 3 -9 234.380 17.044 2

-9 -3 9 199.811 14.277 1

9 9 3 200.265 13.527 3

-9 9 -3 186.260 15.981 2

9 3 9 216.529 14.873 3

3 -9 -9 186.639 13.121 1

9 -4 9 305.804 18.181 3

-4 9 9 272.991 20.205 2

-9 -9 -4 305.000 17.996 1

4 -9 9 252.179 16.782 3

4 9 -9 271.548 15.831 3

9 4 -9 270.862 15.162 3

-9 9 4 269.000 19.713 2

-9 4 9 275.009 21.980 2

9 9 -4 264.976 15.941 3

9 -9 4 283.529 16.507 1

9 4 9 270.019 17.548 3

9 -4 -9 264.034 15.621 1

-9 -9 4 257.821 17.548 1

9 9 4 241.563 16.204 3

-4 9 -9 267.948 20.066 2

-9 9 -4 269.794 19.597 2

4 9 9 276.235 16.878 3

-9 4 -9 292.934 20.244 2

9 -9 -4 270.678 16.381 1

4 -9 -9 260.992 16.220 1

-9 9 5 45.8624 8.8709 2

-9 5 9 36.7736 10.4750 2

-5 9 9 40.6729 9.0069 2

9 5 -9 42.4308 5.2804 3

9 9 -5 37.0126 5.5570 3

5 9 -9 46.5154 5.7052 3

-9 -9 -5 36.6862 6.9324 1

5 9 9 46.9606 6.9732 3

-5 9 -9 33.5204 8.4464 2

9 5 9 49.9145 7.5726 3

-9 9 -5 44.5553 8.8851 2

-9 5 -9 54.2782 9.4731 2

9 -5 -9 35.2402 5.7915 1

9 9 5 48.6541 6.9277 3

5 -9 -9 35.2687 5.9532 1

-9 -9 5 40.5542 7.5343 1

9 -9 -5 47.6567 6.5651 1

-9 9 6 106.175 12.486 2

9 6 -9 106.193 8.620 3

-9 6 9 110.474 14.256 2

9 9 -6 95.7711 8.6542 3

6 9 -9 103.690 8.711 3

-6 9 9 113.735 13.214 2

9 9 6 99.2506 9.8110 3

-6 9 -9 96.7917 12.4262 2

6 -9 -9 113.529 9.801 1

9 -6 -9 95.9772 8.9316 1

-9 -9 6 108.477 10.768 1

-9 9 -6 98.9932 12.0554 2

9 -9 -6 114.814 9.992 1

6 9 9 110.231 10.239 3

-9 6 -9 100.557 12.486 2

9 6 9 105.607 10.299 3

9 9 -7 139.018 10.503 3

-7 9 9 145.913 15.086 2

7 9 -9 134.711 10.357 3

-9 9 7 140.807 14.714 2

9 7 -9 145.606 10.618 3

-9 7 9 163.067 16.443 2

-9 7 -9 144.065 14.773 2

9 7 9 157.055 12.485 3

-9 9 -7 156.110 14.799 2

7 9 9 133.509 11.675 3

-7 9 -9 134.325 14.357 2

-9 -9 7 136.668 12.429 1

7 -9 -9 121.249 11.059 1

9 -9 -7 117.956 10.804 1

9 -7 -9 125.650 10.911 1

9 9 7 146.864 12.017 3

10 0 0 1.89616 2.25558 1

0 10 0 -0.3493 2.0951 3

0 0 10 -1.1629 2.0990 3

10 0 0 -1.0584 2.3757 3

0 -10 0 3.83540 3.61167 1

-10 0 0 5.04308 3.53995 2

0 0 -10 -0.2166 2.9524 2

10 -1 0 149.361 9.197 1

10 1 0 145.978 9.218 1

0 -10 -1 150.718 10.805 1

0 -10 1 149.537 10.835 1

0 10 1 156.099 8.948 3

1 0 10 147.132 9.559 3

1 0 -10 137.178 11.011 2

-1 0 -10 138.433 11.176 2

0 10 -1 164.110 9.056 3

-10 -1 0 136.615 10.053 2

-10 1 0 145.692 11.051 2

-1 0 10 146.692 9.204 3

10 1 0 140.067 9.716 3

10 2 0 2.18887 2.65593 1

-10 2 0 -2.0806 3.9488 2

2 0 10 1.01970 2.71643 3

2 0 -10 4.71188 3.39980 2

-2 0 10 4.14577 2.39669 3

0 10 -2 -1.4425 2.1045 3

0 10 2 4.17186 2.55724 3

-2 0 -10 1.30326 3.33928 2

0 -10 -2 2.23912 3.37441 1

10 2 0 -0.6926 2.6885 3

10 -2 0 -1.5891 2.1328 1

0 -10 2 -0.2254 3.7889 1

0 -10 3 109.607 9.598 1

0 10 3 121.973 8.822 2

3 0 -10 125.561 10.151 2

-3 0 -10 114.358 10.038 2

10 -3 0 112.035 8.028 1

-10 -3 0 131.165 9.470 1

0 -10 -3 124.754 9.557 1

0 10 3 122.254 7.840 3

3 0 10 102.615 8.404 3

0 10 -3 125.793 7.837 3

-10 3 0 105.609 10.051 2

-3 0 10 107.084 7.639 3

10 3 0 130.794 9.344 3

-4 0 10 -0.1447 2.1095 3

4 0 10 0.04648 2.82332 3

0 -10 4 11.8405 4.3699 1

10 -4 0 0.50308 2.68110 1

0 10 -4 4.78325 2.62624 3

0 10 4 -2.8495 2.3263 3

0 10 4 3.52757 3.25745 2

4 0 -10 0.45378 3.28392 2

-10 -4 0 2.95755 3.62672 1

10 4 0 4.95958 3.32387 3

-10 4 0 7.02128 4.72636 2

0 -10 -4 11.6370 3.9676 1

-4 0 -10 2.62250 3.71332 2

10 -5 0 53.8579 5.5735 1

-5 0 -10 42.7814 6.6705 2

-10 5 0 43.4593 7.3292 2

-10 -5 0 51.3587 6.8660 1

0 -10 5 51.3143 6.4496 1

10 5 0 48.3237 6.0594 3

5 0 10 49.4833 5.9767 3

0 -10 -5 53.5496 6.3206 1

0 10 5 56.0342 6.5074 2

0 10 -5 49.9345 5.0705 3

5 0 -10 51.3543 6.5400 2

-5 0 10 48.9285 4.6372 3

10 -6 0 40.6146 5.2958 1

0 10 6 48.7969 6.3983 2

0 -10 6 36.7897 6.0181 1

10 6 0 42.0369 5.8310 3

-10 6 0 40.8515 7.3790 2

-6 0 -10 39.3158 6.7368 2

6 0 10 41.9141 6.0801 3

0 -10 -6 40.9590 5.7608 1

0 10 -6 36.5727 4.5164 3

-6 0 10 43.3166 4.4203 3

-10 -6 0 37.2340 6.7540 1

6 0 -10 36.1617 6.1446 2

10 -7 0 59.3150 6.3104 1

10 7 0 58.2449 6.7216 3

0 10 -7 51.9418 5.6079 3

7 0 -10 58.0875 7.3136 2

-7 0 -10 59.0125 7.8793 2

7 0 10 57.9083 7.2719 3

-10 -7 0 59.9163 8.1538 1

-10 7 0 69.3387 8.8848 2

0 -10 -7 62.6377 6.7825 1

0 10 7 47.7890 7.0889 2

10 8 0 2.66413 3.68718 3

0 -10 -8 10.5823 4.3398 1

-8 0 -10 8.12721 4.79316 2

-10 8 0 2.46138 5.24389 2

8 0 10 8.54009 5.02513 3

0 10 8 1.48245 4.67183 2

10 -8 0 4.42653 3.77868 1

8 0 -10 5.63271 4.27660 2

0 10 -8 4.76259 3.41427 3

-10 -8 0 1.03848 4.99745 1

9 0 10 -0.1896 5.1910 3

-10 9 0 8.20353 6.09100 2

10 -9 0 2.90113 4.22127 1

-10 -9 0 9.86678 6.19292 1

-9 0 10 12.5528 8.0128 2

0 10 9 7.77412 5.48426 2

-9 0 -10 6.00599 4.96492 2

0 -10 9 9.72634 4.78376 3

10 9 0 7.32244 3.99827 3

-10 0 -1 131.984 10.526 2

10 0 1 170.808 10.757 3

0 1 10 135.115 9.298 3

-1 10 0 182.464 10.821 2

1 10 0 152.388 9.128 3

0 1 -10 140.635 11.329 2

-1 10 0 164.517 8.960 3

-1 -10 0 143.148 10.691 1

0 -1 -10 145.206 11.074 2

10 0 -1 154.801 9.366 1

0 -1 10 138.647 9.281 3

-10 0 1 141.993 10.793 2

10 0 1 134.872 9.056 1

1 -10 0 146.911 10.697 1

-1 1 10 -1.1851 1.9774 3

-1 -10 -1 0.16923 3.25079 1

-10 -1 -1 2.20179 3.07607 2

-10 1 1 -4.3591 3.4301 2

1 -10 1 -4.3352 3.0897 1

10 1 -1 3.91648 2.49308 1

-1 10 1 -0.1403 2.7704 2

1 1 -10 0.08172 3.11689 2

-1 -1 -10 5.17050 3.40274 2

10 1 -1 -0.1367 2.4320 3

10 -1 1 1.28788 2.25753 1

1 10 -1 2.62945 2.33492 3

1 -1 10 4.48475 2.76208 3

-10 1 -1 -2.4740 3.4051 2

-1 10 -1 -0.5255 3.0682 2

10 1 1 1.49240 3.33569 3

-1 -1 10 2.30881 2.31097 3

-10 -1 1 -0.0152 3.1138 2

1 -1 -10 0.53584 2.90476 2

-1 10 -1 -0.5247 2.0611 3

1 10 1 3.30073 2.29546 3

1 -10 -1 0.36938 3.12962 1

1 1 10 -0.9775 2.4816 3

-1 -10 1 -2.7484 3.1981 1

10 1 1 -2.2187 2.3012 1

-1 1 -10 1.55054 3.16120 2

10 -1 -1 3.61043 2.48138 1

-2 -1 -10 250.351 16.690 2

2 -1 10 266.944 14.902 3

10 2 -1 278.256 14.768 1

1 -10 2 246.132 15.830 1

10 2 -1 248.020 14.687 3

-2 1 10 270.713 14.318 3

-1 10 2 267.544 15.677 2

-10 2 1 254.630 17.010 2

10 -2 1 260.050 14.314 1

2 1 -10 269.812 17.056 2

-1 -10 -2 282.583 16.193 1

1 10 -2 301.764 14.500 3

-2 -1 10 256.818 14.331 3

-1 10 -2 282.442 14.202 3

2 -1 -10 274.394 17.054 2

-1 10 -2 335.876 17.358 2

2 1 10 260.829 15.105 3

10 2 1 267.707 14.906 1

-10 2 -1 235.318 17.045 2

1 10 2 307.052 14.726 3

1 -10 -2 258.383 16.186 1

-2 1 -10 259.799 17.292 2

10 2 1 274.901 16.266 3

-1 -10 2 243.592 15.884 1

10 -2 -1 294.712 14.963 1

-1 10 3 39.3786 4.9827 2

-3 1 10 28.1342 3.9821 3

10 -3 1 31.4557 4.1211 1

1 10 -3 39.9775 4.3510 3

-10 3 1 23.2065 5.7674 2

-3 -1 -10 23.7676 5.2612 2

3 1 -10 36.4934 5.7953 2

10 3 -1 31.1173 4.6126 3

3 -1 10 25.4965 4.4562 3

-1 -10 -3 30.1557 5.2477 1

1 -10 3 46.0083 5.7438 1

-10 -3 -1 40.0618 5.4129 1

10 -3 -1 30.8180 4.2337 1

-1 -10 3 25.3672 5.2294 1

-3 1 -10 37.6541 6.0458 2

3 -1 -10 35.4666 5.4846 2

-10 3 -1 38.9700 6.2154 2

3 1 10 33.5767 4.8441 3

-1 10 -3 36.6076 4.0454 3

10 3 1 34.6592 5.5550 3

1 -10 -3 35.9551 5.4298 1

1 10 3 35.9605 4.2522 3

-10 -3 1 35.5963 5.3549 1

-1 10 -3 40.5974 5.8924 2

-3 -1 10 39.6277 4.1424 3

1 10 -4 121.758 7.867 3

1 -10 4 106.443 9.082 1

10 4 -1 112.408 8.593 3

-4 -1 -10 102.678 9.626 2

-1 -10 -4 116.279 9.071 1

4 1 -10 112.492 9.602 2

4 -1 10 100.909 8.507 3

-10 -4 -1 116.484 9.451 1

-4 1 10 113.801 7.552 3

-1 10 4 102.670 9.013 2

-10 4 1 92.5757 9.8048 2

10 -4 1 113.445 7.979 1

-4 -1 10 111.334 7.388 3

-10 4 -1 88.8701 9.7965 2

1 -10 -4 93.5242 8.6439 1

10 -4 -1 111.326 7.921 1

-10 -4 1 116.214 9.552 1

10 4 1 106.679 9.075 3

1 10 4 121.091 7.975 3

-1 10 -4 133.554 10.462 2

-1 -10 4 106.826 9.328 1

4 -1 -10 111.128 9.468 2

-1 10 -4 107.712 7.376 3

-4 1 -10 93.4440 9.5238 2

4 1 10 109.613 8.560 3

-10 -5 -1 13.2393 5.0090 1

-5 -1 -10 12.9986 4.4782 2

1 10 -5 15.4241 3.5498 3

-1 10 5 16.6159 4.5577 2

-5 1 10 16.4580 3.1872 3

1 -10 5 8.73958 4.08491 1

-1 -10 -5 22.3220 4.6710 1

5 1 -10 14.9770 4.5594 2

-10 5 1 19.5992 5.9012 2

10 -5 1 16.9852 3.7246 1

10 5 -1 13.8825 3.9571 3

5 -1 10 14.2824 4.2776 3

10 5 1 9.52162 3.97574 3

5 -1 -10 10.8348 4.4839 2

-1 10 -5 20.6870 3.4044 3

-5 1 -10 13.3660 4.9554 2

-1 10 -5 11.3271 5.1234 2

5 1 10 14.8244 4.2058 3

1 10 5 17.4027 3.9499 3

-1 -10 5 12.5622 4.6472 1

-10 5 -1 16.0763 5.6437 2

-5 -1 10 15.5406 3.1282 3

10 -5 -1 11.2895 3.5529 1

1 -10 -5 19.4264 4.2894 1

-10 -5 1 13.2993 4.7083 1

6 1 -10 206.942 15.338 2

10 -6 1 261.444 14.169 1

10 6 -1 219.894 14.167 3

1 10 -6 231.965 13.322 3

-6 -1 -10 237.990 16.036 2

-10 6 1 220.913 16.736 2

6 -1 10 240.260 14.756 3

-1 -10 -6 243.963 14.646 1

-10 -6 -1 231.836 15.695 1

1 -10 6 231.276 14.591 1

-1 10 6 245.164 16.000 2

-10 -6 1 213.549 15.134 1

1 -10 -6 219.014 14.278 1

6 1 10 227.594 14.518 3

-6 -1 10 225.527 12.516 3

-1 10 -6 220.670 12.858 3

6 -1 -10 235.272 15.758 2

-1 -10 6 227.003 14.882 1

10 -6 -1 225.831 13.571 1

10 6 1 231.060 14.531 3

-6 1 -10 244.653 16.349 2

-10 6 -1 228.256 16.835 2

1 10 6 239.004 13.753 3

-7 -1 -10 85.6433 8.9770 2

-10 -7 -1 72.1172 8.7493 1

7 1 -10 75.6376 8.2606 2

-1 10 7 87.9703 9.2634 2

10 7 -1 80.9559 7.6222 3

-1 -10 -7 85.9345 7.8929 1

10 -7 1 86.9713 7.6398 1

1 10 -7 81.3510 6.9716 3

-10 7 1 83.2109 9.9905 2

7 -1 10 78.4295 8.3552 3

-10 -7 1 67.2542 8.6207 1

-7 1 -10 93.8533 9.6663 2

7 1 10 77.4875 8.2355 3

-1 10 -7 73.6182 6.5889 3

-7 -1 10 85.4475 6.6210 3

1 -10 -7 76.7006 7.7560 1

-10 7 -1 68.3553 9.5464 2

10 7 1 76.9833 7.8182 3

10 -7 -1 84.8884 7.5465 1

7 -1 -10 82.8687 8.6922 2

1 -10 8 68.4597 7.6388 3

-8 1 10 66.0796 10.9037 2

8 -1 10 74.8353 8.6207 3

10 -8 1 80.1927 7.5677 1

-1 10 8 79.6446 9.1116 2

10 8 -1 67.9634 7.1641 3

8 1 -10 69.4823 8.1443 2

-10 -8 -1 91.6351 9.5895 1

1 10 -8 79.4207 7.0633 3

-10 8 1 72.0415 9.7007 2

-8 -1 -10 80.8331 8.9025 2

-1 -10 -8 80.5823 7.7511 1

1 -10 -8 83.3352 8.0919 1

-10 -8 1 72.7847 8.9927 1

10 -8 -1 82.2068 7.9433 1

8 1 10 73.0240 8.6616 3

-10 8 -1 85.4769 10.1005 2

10 8 1 92.7420 8.2645 3

-8 1 -10 83.6906 9.5259 2

1 -10 9 22.8604 5.6623 3

10 -9 1 38.9202 6.0753 1

-10 9 1 26.2070 7.3169 2

1 10 -9 43.2809 5.3954 3

-10 -9 -1 30.7406 7.0566 1

-1 10 9 27.6482 7.0065 2

-9 -1 -10 19.3198 6.0699 2

-9 1 10 16.5868 8.5243 2

9 -1 10 34.7808 7.2017 3

10 9 -1 27.0396 5.2740 3

9 1 10 43.0390 7.5930 3

10 9 1 38.5553 5.9504 3

-10 -9 1 32.0048 7.2115 1

-10 9 -1 48.9732 8.3278 2

10 -9 -1 30.8104 5.8327 1

-9 1 -10 31.6037 7.0052 2

0 -2 10 0.46610 2.46889 3

10 0 2 3.73557 3.56757 3

10 0 2 -3.9057 2.2114 1

0 -2 -10 0.52107 2.81586 2

-2 -10 0 2.22165 3.12318 1

-2 10 0 -3.1966 2.9891 2

-10 0 -2 2.19992 3.20600 2

-10 0 2 -0.1149 3.2860 2

2 10 0 -0.4464 2.2130 3

2 -10 0 -0.1639 3.1183 1

0 2 10 0.41696 2.35995 3

10 0 -2 -1.8241 2.2661 1

0 2 -10 -3.8194 2.9204 2

10 1 -2 271.101 14.222 3

2 10 -1 273.564 14.563 3

10 -1 2 255.816 14.359 1

-1 -2 -10 261.671 16.671 2

1 2 -10 259.750 17.310 2

-2 10 1 313.083 16.517 2

-10 1 2 278.166 17.106 2

2 -10 1 274.646 16.139 1

-2 -10 -1 260.497 16.026 1

1 -2 10 266.315 14.903 3

10 1 -2 271.021 14.774 1

-1 2 10 274.317 14.689 3

-10 -1 -2 259.360 15.881 2

-2 -10 1 284.197 16.236 1

-10 1 -2 271.290 16.656 2

10 1 2 251.790 14.428 1

-10 -1 2 256.772 15.744 2

10 1 2 257.144 16.051 3

-1 2 -10 247.657 17.135 2

1 2 10 283.754 14.961 3

10 -1 -2 266.229 14.535 1

2 10 1 245.689 14.166 3

1 -2 -10 277.841 16.707 2

-1 -2 10 260.803 14.362 3

-2 10 -1 334.451 17.073 2

2 -10 -1 266.068 15.953 1

-2 10 2 1527.95 74.05 2

-2 -2 -10 1543.25 75.02 2

2 10 -2 1624.34 68.50 3

10 2 -2 1515.01 69.58 1

-2 -10 -2 1554.38 70.73 1

10 -2 2 1546.09 69.20 1

-10 2 2 1454.33 75.08 2

2 2 -10 1527.20 75.67 2

2 -2 10 1472.78 68.89 3

-2 2 10 1547.27 68.39 3

2 -10 2 1519.74 70.79 1

10 2 -2 1500.10 68.37 3

2 10 2 1540.64 68.34 3

10 2 2 1629.07 69.74 1

10 2 2 1523.09 70.43 3

2 -2 -10 1534.44 74.97 2

-2 2 -10 1497.07 75.76 2

-2 -2 10 1546.66 68.35 3

-2 -10 2 1505.67 71.08 1

2 -10 -2 1529.18 70.93 1

-10 2 -2 1441.78 74.98 2

10 -2 -2 1554.97 69.54 1

2 2 10 1498.36 68.93 3

10 3 -2 264.174 15.343 1

3 -2 10 260.170 15.551 3

-10 3 2 260.377 17.779 2

2 -10 3 293.426 16.460 1

-10 -3 -2 285.186 16.197 1

-2 -10 -3 298.708 16.554 1

-3 2 10 282.031 14.850 3

-3 -2 -10 279.589 17.260 2

-2 10 3 289.191 16.793 2

2 10 -3 293.222 15.038 3

10 -3 2 270.634 14.884 1

10 3 -2 264.422 15.187 3

3 2 -10 267.648 17.499 2

-2 10 -3 346.218 18.532 2

10 3 2 241.201 16.435 3

10 -3 -2 267.425 15.041 1

-3 -2 10 284.983 14.817 3

2 10 3 267.266 14.940 3

-2 10 -3 280.714 14.236 3

-10 3 -2 252.792 17.654 2

-3 2 -10 285.163 18.096 2

3 -2 -10 270.916 17.085 2

-2 -10 3 278.743 16.818 1

3 2 10 262.233 15.485 3

2 -10 -3 294.362 16.471 1

2 10 -4 19.6339 3.5948 3

2 -10 4 9.93026 4.05328 1

-10 -4 -2 23.6282 4.9931 1

-2 -10 -4 11.8152 4.1661 1

-4 2 10 12.4175 3.0786 3

10 4 -2 9.26578 3.44870 3

4 2 -10 14.2740 4.5647 2

10 -4 2 15.3166 3.3979 1

-2 10 4 5.35509 3.67614 2

4 -2 10 9.46513 3.82368 3

-4 -2 -10 16.7985 4.3710 2

-10 4 2 17.3172 5.6312 2

2 10 4 13.3475 3.4315 3

-10 4 -2 15.1682 5.5412 2

-2 -10 4 12.0239 4.6316 1

10 -4 -2 12.6476 3.3587 1

-4 -2 10 17.8723 3.3296 3

10 4 2 10.4192 4.4046 3

-10 -4 2 11.1650 4.3419 1

4 2 10 14.6335 4.0739 3

-2 10 -4 11.4103 2.8964 3

-4 2 -10 14.8925 5.0150 2

4 -2 -10 15.2212 4.2900 2

-2 10 -4 15.5858 5.1434 2

2 -10 -4 16.1419 4.2820 1

10 5 -2 131.850 9.969 3

-10 5 2 151.257 12.905 2

-10 -5 -2 144.200 11.387 1

-2 10 5 147.567 11.541 2

2 10 -5 152.742 9.589 3

-5 2 10 158.155 9.377 3

2 -10 5 161.406 10.883 1

-2 -10 -5 167.524 11.149 1

5 -2 10 133.888 10.505 3

5 2 -10 139.028 11.377 2

-5 -2 -10 142.937 11.514 2

10 -5 2 144.012 9.657 1

-10 -5 2 134.856 11.244 1

2 10 5 156.823 10.339 3

5 2 10 152.944 10.767 3

-5 2 -10 141.756 12.398 2

-2 10 -5 181.150 13.354 2

-10 5 -2 132.954 12.549 2

-2 10 -5 140.654 9.108 3

2 -10 -5 158.938 11.085 1

10 5 2 166.752 11.804 3

5 -2 -10 155.221 11.794 2

-5 -2 10 148.094 9.335 3

10 -5 -2 150.443 9.984 1

-2 -10 5 139.179 11.249 1

10 6 -2 1016.49 48.55 3

-10 6 2 1035.08 54.35 2

-10 -6 -2 975.226 50.400 1

2 10 -6 1042.11 47.90 3

6 2 -10 1035.01 52.77 2

-6 -2 -10 1040.86 52.96 2

2 -10 6 1031.14 49.07 1

-2 10 6 1006.93 53.21 2

6 -2 10 1026.51 49.18 3

-2 -10 -6 1167.44 50.04 1

10 -6 2 1049.01 48.74 1

-10 -6 2 982.356 49.090 1

-2 10 -6 961.117 45.657 3

10 -6 -2 1016.27 47.32 1

-6 2 -10 1018.20 52.38 2

2 10 6 1050.69 47.00 3

-2 -10 6 964.959 48.295 1

-6 -2 10 993.547 45.694 3

10 6 2 1021.42 48.04 3

6 2 10 1000.37 47.64 3

6 -2 -10 1038.37 51.66 2

2 -10 -6 955.397 47.923 1

7 2 -10 224.436 15.442 2

7 -2 10 248.047 15.433 3

-7 -2 -10 232.121 15.986 2

2 -10 7 213.753 14.117 3

2 10 -7 230.449 13.669 3

-10 7 2 226.453 17.215 2

-10 -7 -2 239.269 16.081 1

10 -7 2 251.732 14.504 1

-2 10 7 217.540 16.366 2

10 7 -2 234.070 14.327 3

-2 -10 -7 271.956 15.158 1

10 7 2 209.180 14.374 3

-7 2 -10 224.749 16.699 2

-2 10 -7 263.709 17.797 2

-10 -7 2 218.575 15.785 1

-7 -2 10 219.549 12.718 3

2 -10 -7 212.741 14.171 1

2 10 7 260.893 14.714 3

7 -2 -10 229.381 15.896 2

7 2 10 229.346 15.096 3

-10 7 -2 204.362 16.660 2

10 -7 -2 233.789 14.033 1

2 -10 8 1.01108 3.94956 3

-10 8 2 8.91835 5.61102 2

8 -2 10 1.30068 4.64435 3

-8 -2 -10 5.35764 4.60653 2

-8 2 10 -1.8749 6.4826 2

10 -8 2 4.54974 3.87483 1

-2 10 8 -0.4534 4.8236 2

10 8 -2 6.12165 3.60828 3

-10 -8 -2 -3.5767 4.8198 1

2 10 -8 0.56711 3.10478 3

10 -8 -2 1.83198 3.70382 1

2 10 8 -0.0697 4.1237 3

-10 -8 2 -3.8167 5.2382 1

2 -10 -8 4.82566 4.02114 1

8 2 10 2.53096 4.80154 3

-8 2 -10 7.72175 5.36018 2

10 8 2 7.07926 4.18128 3

-2 10 -8 1.21487 5.61029 2

-10 8 -2 -2.1621 5.2065 2

-8 -2 10 -0.7080 2.6585 3

-10 9 2 37.3668 8.0395 2

-10 -9 -2 42.5680 7.8131 1

9 -2 10 53.7600 8.1308 3

10 9 -2 51.9594 6.3562 3

2 10 -9 57.7028 6.2307 3

-9 2 10 52.7420 10.7576 2

10 -9 2 51.0473 6.7161 1

-2 10 9 40.6568 7.9746 2

-9 -2 -10 50.0970 7.2399 2

2 -10 9 57.2715 7.4390 3

-10 9 -2 41.5392 8.3411 2

-10 -9 2 46.0656 8.0109 1

-9 2 -10 47.8666 8.0117 2

-2 10 -9 63.3842 9.1903 2

10 -9 -2 46.2727 6.4631 1

9 2 10 42.2752 7.6146 3

10 9 2 41.5068 6.2023 3

-3 10 0 129.398 9.742 2

0 -3 10 127.760 8.340 3

-10 0 -3 103.980 9.271 2

10 0 -3 111.620 7.914 1

3 -10 0 107.457 9.072 1

10 0 3 98.3996 9.3547 3

10 0 3 110.207 7.767 1

0 3 10 116.877 8.267 3

0 -3 -10 130.085 9.588 2

-10 0 3 110.702 9.659 2

-3 -10 0 121.160 9.598 1

0 3 -10 107.834 10.160 2

3 10 0 119.548 7.959 3

-3 10 1 28.2608 5.0721 2

-1 -3 -10 37.3260 5.3911 2

10 -1 3 24.5600 5.2681 3

10 1 -3 29.6820 3.7468 3

10 -1 3 31.4496 3.9879 1

3 10 -1 38.1585 4.3917 3

-1 3 10 39.7322 4.6614 3

3 -10 1 35.7130 5.3672 1

10 1 -3 37.7966 4.3900 1

-3 -10 -1 27.9779 5.2319 1

-10 -1 -3 40.7843 5.1633 2

1 -3 10 33.5135 4.6038 3

-10 1 3 37.0893 6.0705 2

1 3 -10 35.1258 6.1959 2

-10 1 -3 29.9175 5.5994 2

-3 -10 1 38.9573 5.9727 1

1 -3 -10 32.1013 5.1653 2

10 -1 -3 27.2278 4.0487 1

-3 10 -1 37.1699 5.6019 2

-1 3 -10 30.7369 5.8420 2

1 3 10 39.4452 4.7994 3

10 1 3 44.0748 4.6184 1

3 -10 -1 33.2080 5.2431 1

10 1 3 21.9960 5.4459 3

-1 -3 10 35.7287 4.4061 3

3 10 1 32.9284 4.2754 3

-2 -3 -10 291.419 17.076 2

10 -2 3 280.294 14.620 1

-10 -2 -3 279.699 15.639 1

-3 10 2 275.351 16.684 2

-3 -10 -2 289.962 16.614 1

-2 3 10 283.967 14.886 3

2 -3 10 266.196 15.405 3

2 3 -10 250.489 17.469 2

3 10 -2 290.333 14.896 3

-10 2 3 238.827 17.414 2

3 -10 2 268.752 16.171 1

10 2 -3 272.056 15.000 1

10 2 -3 270.690 14.514 3

3 -10 -2 265.933 16.102 1

-2 -3 10 282.868 14.945 3

2 -3 -10 278.179 16.919 2

-3 10 -2 316.720 17.919 2

3 10 2 263.946 14.781 3

2 3 10 277.703 15.435 3

-2 3 -10 244.386 17.718 2

10 -2 -3 273.708 14.971 1

-3 -10 2 271.259 16.752 1

-10 2 -3 269.391 17.598 2

10 2 3 273.862 16.976 3

3 3 -10 90.5886 8.7695 2

-3 3 10 88.1608 6.8002 3

3 -3 10 68.4752 6.9960 3

10 3 -3 80.5726 6.9704 3

3 10 -3 86.0261 6.7011 3

-10 3 3 67.5249 8.5511 2

3 -10 3 72.1576 7.3764 1

10 -3 3 94.4949 6.7626 1

10 3 -3 77.3838 6.8986 1

-3 10 3 88.0641 8.0577 2

-3 -10 -3 101.375 8.408 1

-10 -3 -3 87.1105 7.7616 1

-3 -3 -10 86.9303 8.1285 2

-3 3 -10 74.7126 8.7684 2

-3 -10 3 82.1696 8.2559 1

3 -3 -10 81.5305 7.8557 2

3 10 3 80.6660 6.9053 3

-3 -3 10 105.933 7.205 3

10 -3 -3 71.4254 6.6125 1

-3 10 -3 94.6322 9.0373 2

3 3 10 91.0527 7.6509 3

-10 3 -3 75.5865 8.6984 2

3 -10 -3 93.6569 7.9739 1

10 3 3 84.9113 8.6834 3

-3 10 4 92.3686 9.3104 2

4 -3 10 113.933 9.209 3

-3 -10 -4 126.053 9.351 1

-10 4 3 117.877 11.252 2

10 -4 3 115.734 8.029 1

10 4 -3 106.882 8.485 3

3 10 -4 114.678 8.159 3

4 3 -10 104.727 9.786 2

3 -10 4 110.533 9.184 1

-4 -3 -10 118.681 9.777 2

-10 -4 -3 127.760 9.924 1

-4 3 10 129.511 8.249 3

-4 3 -10 113.096 10.844 2

-3 -10 4 110.807 9.806 1

4 -3 -10 110.977 9.687 2

3 -10 -4 108.226 9.125 1

-10 -4 3 112.029 9.576 1

3 10 4 102.284 8.349 3

-4 -3 10 118.461 8.011 3

10 4 3 92.4452 9.4073 3

-3 10 -4 127.563 11.077 2

4 3 10 113.873 9.061 3

10 -4 -3 109.444 8.182 1

-10 4 -3 126.335 11.259 2

5 3 -10 9.86629 4.19534 2

10 5 -3 7.69604 3.33583 3

3 10 -5 9.99112 3.37376 3

-10 5 3 5.04299 5.31226 2

-5 -3 -10 8.98792 3.98442 2

5 -3 10 1.34606 3.79748 3

-10 -5 -3 10.9456 4.6316 1

-3 -10 -5 3.85499 3.74933 1

10 -5 3 4.62734 3.15411 1

3 -10 5 8.47536 3.87296 1

-3 10 5 13.6498 4.8868 2

-10 -5 3 9.26637 4.75829 1

-3 -10 5 7.31474 4.62967 1

-3 10 -5 7.17294 4.98530 2

5 3 10 6.59591 3.83355 3

3 10 5 8.96944 3.79176 3

-5 -3 10 6.96435 2.97338 3

3 -10 -5 6.15683 3.83367 1

10 -5 -3 4.61720 3.15029 1

-10 5 -3 8.05840 5.25159 2

-5 3 -10 8.75705 4.89933 2

10 5 3 7.94149 4.48703 3

5 -3 -10 12.2083 4.4504 2

-3 10 6 281.662 18.889 2

-10 -6 -3 319.906 18.457 1

-6 -3 -10 275.969 18.080 2

3 10 -6 283.631 16.053 3

10 6 -3 267.757 16.406 3

-10 6 3 285.746 19.916 2

10 -6 3 319.774 16.476 1

-3 -10 -6 328.058 17.632 1

3 -10 6 290.542 16.340 3

6 -3 10 280.182 17.269 3

6 3 -10 286.640 17.958 2

-6 -3 10 268.929 15.500 3

-6 3 -10 284.715 19.664 2

6 -3 -10 286.021 18.638 2

3 -10 -6 289.999 17.157 1

3 10 6 293.929 16.867 3

10 6 3 283.496 17.810 3

6 3 10 291.262 17.417 3

10 -6 -3 284.962 16.392 1

-3 -10 6 293.393 18.066 1

-10 -6 3 317.470 18.689 1

-3 10 -6 343.649 20.485 2

-10 6 -3 263.781 19.611 2

-3 -10 -7 132.035 9.693 1

3 -10 7 90.1868 8.5039 3

3 10 -7 99.3445 8.1857 3

10 7 -3 115.274 8.838 3

-3 10 7 99.8890 10.7363 2

-10 -7 -3 97.8415 10.1014 1

7 -3 10 115.281 9.955 3

7 3 -10 102.289 7.566 3

10 -7 3 108.424 8.829 1

-7 -3 -10 110.509 9.964 2

-10 7 3 100.583 11.265 2

-7 3 -10 114.724 11.509 2

-3 -10 7 110.107 10.077 1

-3 10 -7 129.095 12.233 2

-10 7 -3 118.043 11.836 2

7 3 10 108.147 9.939 3

10 -7 -3 110.063 8.920 1

10 7 3 106.376 9.794 3

3 -10 -7 90.8883 8.8344 1

-10 -7 3 89.0139 9.9488 1

7 -3 -10 124.284 10.784 2

3 10 7 107.114 9.256 3

3 -10 8 55.6380 7.0781 3

10 8 -3 55.4288 6.4526 3

-8 -3 -10 61.0347 7.6829 2

-10 -8 -3 67.3920 8.4057 1

-10 8 3 54.0600 9.1500 2

8 -3 10 68.2983 8.2952 3

8 3 -10 62.1997 5.7117 3

3 10 -8 70.8006 6.5968 3

-8 3 10 56.7807 11.0602 2

10 -8 3 65.8099 7.0765 1

-3 10 8 68.2527 9.2574 2

10 8 3 55.9894 7.3040 3

10 -8 -3 53.9288 6.5816 1

-10 -8 3 66.4592 8.9225 1

-3 10 -8 65.0055 9.5449 2

-8 3 -10 51.4198 8.5707 2

3 10 8 66.7708 7.5227 3

8 3 10 71.2582 8.4990 3

3 -10 -8 52.3852 6.6591 1

-10 8 -3 65.2373 9.2863 2

3 -10 9 10.0492 5.1028 3

-10 9 3 13.8190 6.8536 2

3 10 -9 11.0115 4.0123 3

-10 -9 -3 7.85395 5.64189 1

9 -3 10 10.8147 6.0447 3

-3 10 9 2.48001 5.77521 2

10 9 -3 7.84158 3.94689 3

-9 3 10 15.2378 8.5982 2

10 -9 3 3.10018 4.38614 1

3 10 9 14.0431 5.1913 3

-10 -9 3 13.6714 5.9654 1

-10 9 -3 4.99837 5.77162 2

9 3 10 7.35322 5.22506 3

-9 3 -10 16.0588 6.6325 2

-3 10 -9 12.4901 6.9369 2

10 9 3 15.4795 5.0082 3

10 -9 -3 9.50526 4.58379 1

0 -4 -10 0.85357 3.15117 2

-4 10 0 4.65450 3.74013 2

10 0 4 1.20416 3.85791 3

-10 0 -4 1.72207 3.42264 2

-10 0 4 -1.6912 3.9697 2

0 4 -10 2.21802 4.01482 2

4 10 0 3.23590 2.52768 3

0 4 10 3.70012 2.90623 3

10 0 4 6.05267 2.71467 1

0 -4 10 -1.0220 2.4886 3

10 0 -4 7.34715 2.87461 1

4 -10 0 2.69909 3.36868 1

-4 -10 0 0.59822 3.55743 1

-10 1 4 121.517 10.267 2

10 -1 4 108.814 7.442 1

4 -10 1 110.284 8.965 1

10 1 -4 95.3171 7.6041 1

-4 -10 -1 110.633 9.356 1

-10 -1 -4 94.5056 8.6405 2

1 -4 10 98.5966 8.1140 3

10 -1 4 118.789 9.946 3

4 10 -1 106.880 7.612 3

1 4 -10 102.060 9.878 2

-1 4 10 117.599 8.146 3

-1 -4 -10 113.541 8.968 2

-4 10 1 104.138 9.262 2

4 -10 -1 114.874 9.127 1

10 -1 -4 107.457 7.784 1

-1 4 -10 103.284 10.229 2

1 -4 -10 108.430 8.963 2

10 1 4 103.785 9.593 3

10 1 4 112.799 7.914 1

-10 1 -4 103.521 9.495 2

-1 -4 10 104.100 7.992 3

-4 10 -1 105.917 9.507 2

4 10 1 115.967 7.994 3

-4 -10 1 104.414 9.334 1

1 4 10 114.044 8.453 3

-10 -2 -4 8.06954 3.83402 1

-10 2 4 6.99093 5.01819 2

2 4 -10 14.9672 4.8576 2

2 -4 10 10.6569 3.6061 3

-4 -10 -2 8.64258 4.19089 1

-2 -4 -10 13.5304 4.0412 2

-4 10 2 11.2930 4.3064 2

-2 4 10 17.1143 3.5964 3

4 -10 2 13.2704 4.0792 1

10 2 -4 15.9160 3.1352 3

10 -2 4 5.85649 2.80241 1

4 10 -2 12.6808 3.1841 3

10 2 -4 11.3863 3.2479 1

4 -10 -2 7.09924 3.74329 1

-2 -4 10 10.7648 3.3630 3

2 -4 -10 8.85869 3.61519 2

10 -2 -4 18.3170 3.6107 1

-2 4 -10 10.5249 5.0266 2

-10 2 -4 9.23738 4.66616 2

10 2 4 4.61689 4.30055 3

-4 10 -2 23.9169 5.1330 2

-4 -10 2 9.70409 4.51772 1

2 4 10 6.43987 3.48552 3

4 10 2 16.4012 3.5043 3

10 3 -4 111.808 8.396 1

3 -4 10 109.701 8.875 3

4 10 -3 115.809 8.229 3

10 3 -4 106.350 7.881 3

-10 3 4 111.415 10.818 2

-10 -3 -4 121.564 9.374 1

-3 -4 -10 108.548 9.227 2

10 -3 4 130.370 8.001 1

-3 4 10 120.639 8.267 3

3 4 -10 119.791 10.503 2

4 -10 3 111.930 9.072 1

-4 -10 -3 117.912 9.511 1

-4 10 3 93.6631 9.3763 2

-4 10 -3 129.256 10.661 2

-3 -4 10 124.062 8.456 3

10 -3 -4 110.272 8.049 1

4 -10 -3 107.720 9.141 1

10 3 4 120.410 10.375 3

-3 4 -10 114.909 11.042 2

4 10 3 105.731 8.219 3

3 4 10 114.167 8.999 3

3 -4 -10 116.544 9.626 2

-10 3 -4 108.624 10.448 2

-4 -10 3 90.2441 9.0950 1

-4 10 4 7.28460 4.28810 2

-10 4 4 -0.5479 4.8395 2

4 4 -10 10.4865 4.3744 2

4 10 -4 15.0729 3.5225 3

10 4 -4 7.90201 3.20621 3

4 -10 4 7.89570 3.89268 1

-10 -4 -4 8.36702 4.25131 1

4 -4 10 1.96076 3.58498 3

-4 -10 -4 12.8085 4.2186 1

10 -4 4 4.96422 2.96614 1

-4 -4 -10 7.02606 4.07198 2

10 -4 -4 6.15297 3.06217 1

-4 -4 10 5.91077 3.05122 3

-10 4 -4 8.02925 5.34637 2

-4 10 -4 12.8295 5.2562 2

4 10 4 6.35670 3.44328 3

10 4 4 3.81045 4.18285 3

4 4 10 4.82018 3.64057 3

-10 -4 4 6.25168 4.17903 1

4 -4 -10 12.8793 4.1656 2

-4 -10 4 4.78668 4.33830 1

4 -10 -4 9.50401 3.93863 1

-4 4 -10 1.53683 4.23372 2

-10 5 4 73.1564 9.1266 2

-10 -5 -4 59.7190 7.4905 1

5 -4 10 60.2163 7.4230 3

5 4 -10 71.4301 6.0882 3

10 5 -4 58.0464 6.1931 3

4 -10 5 73.6002 6.9144 1

-4 -10 -5 83.0096 7.4579 1

5 4 -10 59.3533 7.2339 2

-5 -4 -10 64.0576 7.3534 2

10 -5 4 68.9846 6.1273 1

4 10 -5 80.1842 6.6138 3

-4 10 5 57.4976 7.8440 2

10 5 4 63.5768 7.9998 3

-5 4 -10 58.8800 8.5826 2

4 10 5 64.2594 6.8143 3

-4 -10 5 69.2713 7.7556 1

4 -10 -5 71.3238 7.2206 1

-10 -5 4 51.5749 7.3482 1

-10 5 -4 50.4170 8.3666 2

-4 10 -5 78.7786 9.0793 2

5 4 10 61.4155 7.0504 3

5 -4 -10 60.5588 7.3377 2

10 -5 -4 60.3801 6.1444 1

6 -4 10 1.60452 4.23950 3

10 -6 4 3.49138 3.45444 1

-10 6 4 8.12239 5.87508 2

-4 -10 -6 5.46820 3.96787 1

10 6 -4 4.17306 3.30949 3

-10 -6 -4 4.02840 4.73105 1

-4 10 6 2.93854 4.80222 2

6 4 -10 4.89394 2.88472 3

-6 -4 -10 4.71300 4.09793 2

4 10 -6 15.3104 3.7120 3

-4 -10 6 13.4921 5.2033 1

10 -6 -4 6.38718 3.57783 1

4 10 6 7.71920 4.18482 3

-10 6 -4 9.31386 6.24656 2

6 4 10 7.22300 4.55632 3

10 6 4 3.81332 4.53532 3

6 -4 -10 11.2899 4.6119 2

-4 10 -6 2.07491 5.22811 2

4 -10 -6 4.18227 3.87268 1

-10 -6 4 8.17097 5.05891 1

-6 4 -10 4.55706 5.47878 2

-10 -7 -4 47.9684 7.1664 1

-7 -4 -10 49.5109 6.8172 2

7 -4 10 52.4725 7.3514 3

7 4 -10 48.9864 5.2788 3

-4 10 7 55.7275 8.3825 2

-7 4 10 69.6854 10.9929 2

10 7 -4 36.7141 5.5880 3

-4 -10 -7 64.7709 7.0531 1

10 -7 4 50.8066 6.0782 1

-10 7 4 50.3069 8.6640 2

4 10 -7 58.6962 5.9553 3

-4 10 -7 51.9907 8.4687 2

-7 4 -10 42.5741 8.3466 2

10 -7 -4 44.6672 5.9509 1

-4 -10 7 40.9360 7.3178 1

4 10 7 48.5863 6.5923 3

4 -10 -7 45.8737 6.3362 1

7 4 10 38.7518 6.7594 3

-10 -7 4 46.3568 7.6981 1

-10 7 -4 33.4936 8.0015 2

10 7 4 39.6446 6.6243 3

10 8 -4 16.4956 4.4572 3

8 4 -10 11.6076 3.5376 3

-10 -8 -4 15.5807 6.0168 1

4 10 -8 15.5272 4.1776 3

-10 8 4 21.6531 7.1617 2

-8 4 10 9.36934 7.95296 2

8 -4 10 20.6438 6.0866 3

10 -8 4 13.6325 4.7200 1

-4 10 8 13.4296 6.4988 2

4 -10 -8 8.49697 4.44122 1

-4 10 -8 31.7399 7.8196 2

4 10 8 16.5814 5.3000 3

10 -8 -4 13.3619 4.4657 1

10 8 4 6.05444 4.83731 3

-10 8 -4 20.2936 6.9972 2

-10 -8 4 7.48248 5.80882 1

8 4 10 9.35432 5.03105 3

-8 4 -10 19.9316 6.9656 2

-10 -9 -4 14.1464 6.0437 1

-10 9 4 8.41845 6.56712 2

-9 4 10 16.3263 9.4039 2

4 10 -9 11.0614 4.1354 3

9 4 -10 8.77866 3.56077 3

-4 10 9 24.6560 7.2762 2

10 9 -4 6.04913 3.78684 3

9 -4 10 12.3813 6.3894 3

-10 -9 4 15.8394 6.6027 1

10 -9 -4 12.1839 4.8065 1

9 4 10 7.32301 5.46501 3

4 -10 -9 18.2010 5.4108 1

-10 9 -4 5.13635 6.31223 2

10 9 4 0.65118 4.13110 3

4 10 9 18.6862 5.5669 3

-4 10 -9 13.4430 7.1150 2

-9 4 -10 -0.7516 5.6445 2

0 5 -10 40.6131 6.9873 2

0 5 10 58.1901 5.8873 3

-5 -10 0 62.0428 7.1418 1

-10 0 -5 51.1399 6.5806 2

5 -10 0 49.2323 6.2035 1

10 0 5 53.1770 5.1527 1

10 0 -5 55.4886 5.3804 1

5 10 0 51.5251 5.4584 3

0 -5 10 60.0308 6.0566 3

0 -5 -10 51.9877 6.0170 2

10 0 5 45.4790 6.8619 3

-10 0 5 58.2437 7.5587 2

-5 10 0 48.3968 6.7849 2

-1 -5 -10 14.9214 4.0370 2

5 10 -1 16.3395 3.5278 3

-1 5 10 8.63621 3.43566 3

10 -1 5 16.6302 3.3808 1

-10 -1 -5 10.2564 3.6511 1

1 -5 10 20.3982 4.1369 3

10 1 -5 14.9863 3.5373 1

-5 -10 -1 18.1062 4.8705 1

5 -10 1 15.9272 4.4515 1

1 5 -10 19.1730 5.2615 2

-10 -1 -5 12.2730 4.3070 2

-5 10 1 18.4112 4.8860 2

-10 1 5 17.4181 5.6666 2

10 -1 5 16.4920 5.0779 3

1 -5 -10 18.0118 4.2691 2

-5 10 -1 21.0313 5.1932 2

1 5 10 14.0362 4.0090 3

-5 -10 1 10.2263 4.4531 1

5 10 1 18.2693 3.7574 3

-10 1 -5 16.1330 4.9825 2

-1 -5 10 13.2822 3.7152 3

-1 5 -10 16.5668 5.6318 2

5 -10 -1 8.83532 4.03517 1

10 -1 -5 11.2638 3.3140 1

10 1 5 17.6266 5.0440 3

-2 -5 -10 144.110 10.762 2

2 5 -10 138.678 11.945 2

2 -5 10 155.615 10.578 3

5 10 -2 132.767 9.479 3

10 2 -5 140.862 9.705 1

-5 10 2 134.337 11.578 2

10 -2 5 157.779 9.260 1

5 -10 2 146.056 10.683 1

10 -2 5 173.808 12.220 3

-2 5 10 157.399 10.026 3

-10 2 5 142.911 12.840 2

-5 -10 -2 139.395 11.045 1

10 2 -5 157.560 9.084 3

-10 -2 -5 155.757 10.559 1

10 2 5 173.590 12.502 3

5 10 2 160.277 10.324 3

-5 -10 2 157.997 11.935 1

-10 2 -5 155.288 12.562 2

5 -10 -2 148.955 10.919 1

-2 -5 10 152.291 10.284 3

10 -2 -5 152.122 10.033 1

-5 10 -2 156.358 12.249 2

-2 5 -10 149.297 13.034 2

2 5 10 162.063 10.890 3

2 -5 -10 154.228 11.417 2

3 -5 10 5.68508 3.61585 3

-5 -10 -3 7.54799 4.42571 1

-5 10 3 7.14573 4.24550 2

-3 -5 -10 7.52508 3.76062 2

-10 -3 -5 7.89304 4.17344 1

-10 3 5 11.8646 5.8294 2

5 -10 3 7.71193 3.85589 1

10 3 -5 5.80288 2.84696 3

5 10 -3 11.8448 3.2971 3

3 5 -10 8.73228 4.51077 2

3 -5 -10 6.92396 3.83390 2

10 -3 -5 8.39844 3.28108 1

10 3 5 2.41317 4.07321 3

-10 -3 5 10.1450 4.3527 1

-5 10 -3 9.00423 4.82999 2

5 10 3 7.40559 3.25479 3

3 5 10 6.44924 3.90501 3

-3 5 -10 5.82316 4.96285 2

-5 -10 3 9.35906 4.74580 1

-10 3 -5 4.56429 4.88189 2

5 -10 -3 6.90505 3.81464 1

-3 -5 10 9.62155 3.51913 3

-5 10 4 64.5141 7.9399 2

4 -5 10 66.5341 7.3191 3

-4 -5 -10 59.3365 6.9428 2

-10 -4 -5 65.5645 7.4449 1

5 10 -4 56.8817 5.9977 3

5 -10 4 76.2193 7.0573 1

-5 -10 -4 77.3124 7.6862 1

10 4 -5 65.1461 6.1882 3

4 5 -10 75.0362 7.8807 2

4 5 -10 67.6827 5.9748 3

-10 4 5 69.4358 9.3142 2

4 5 10 72.0468 7.5873 3

10 4 5 63.1464 8.0034 3

-5 10 -4 80.4453 9.1719 2

4 -5 -10 66.5331 7.4624 2

-4 5 -10 72.9527 9.4226 2

5 -10 -4 68.7781 7.1853 1

10 -4 -5 64.0941 6.3249 1

-5 -10 4 59.2162 7.5334 1

5 10 4 71.2967 7.0079 3

-10 -4 5 73.5644 7.7410 1

-10 4 -5 63.1917 8.7962 2

5 5 -10 77.3102 6.2428 3

5 -5 10 58.7595 7.1798 3

-5 -5 -10 55.0419 6.7334 2

5 -10 5 59.0760 6.4771 1

-5 10 5 50.6394 7.6377 2

10 5 -5 43.0069 5.5700 3

-5 -10 -5 65.9780 7.0356 1

5 10 -5 62.3361 6.1332 3

-10 -5 -5 66.9319 7.6441 1

-10 5 5 52.8048 8.9095 2

-10 -5 5 66.7471 8.0475 1

10 5 5 59.3429 7.6692 3

10 -5 -5 63.8152 6.3420 1

-10 5 -5 70.7675 9.4314 2

5 5 10 57.0104 7.1997 3

5 -10 -5 61.1979 6.9950 1

-5 5 -10 51.6433 8.6137 2

5 -5 -10 58.7623 7.1476 2

-5 10 -5 54.7694 8.4845 2

5 10 5 63.0242 6.9765 3

-5 -10 5 49.4125 7.2462 1

6 -5 10 160.274 11.890 3

-5 10 6 133.514 12.662 2

-5 -10 -6 161.099 10.968 1

-10 -6 -5 135.120 11.321 1

5 10 -6 136.349 9.628 3

-10 6 5 153.012 14.022 2

-6 -5 -10 141.766 11.102 2

6 5 -10 131.715 9.220 3

10 6 -5 121.593 9.797 3

5 10 6 145.420 10.927 3

-10 -6 5 121.993 11.409 1

6 5 10 145.820 11.488 3

5 -10 -6 139.834 10.627 1

10 6 5 135.653 11.636 3

-10 6 -5 116.638 12.863 2

-5 10 -6 165.502 13.695 2

-6 5 -10 152.480 13.740 2

-5 -10 6 128.358 11.514 1

10 -6 -5 131.092 9.979 1

-7 5 10 29.1089 9.5144 2

-5 -10 -7 50.4827 6.6565 1

5 10 -7 54.2514 5.8483 3

7 -5 10 36.3806 6.8539 3

-10 7 5 35.6704 8.1926 2

-5 10 7 60.2805 8.7635 2

-10 -7 -5 40.5995 6.9341 1

10 7 -5 43.0274 5.8442 3

7 5 -10 46.2521 5.4146 3

-10 -7 5 59.1041 8.3463 1

-5 10 -7 57.4242 8.9073 2

7 5 10 39.4357 6.6553 3

10 7 5 45.6282 7.1157 3

5 10 7 48.8518 6.9009 3

5 -10 -7 41.6497 6.4691 1

10 -7 -5 40.1305 5.8735 1

-7 5 -10 52.2938 8.9500 2

-10 7 -5 41.3672 8.6520 2

-5 -10 7 42.3834 7.2071 1

-10 -8 -5 43.3299 7.4016 1

8 -5 10 57.4551 8.0707 3

10 8 -5 38.3476 5.8069 3

5 10 -8 37.7857 5.4304 3

8 5 -10 45.5196 5.5893 3

-10 8 5 46.3564 9.0861 2

-5 10 8 52.4091 8.9131 2

-8 5 10 53.2630 11.1529 2

-10 -8 5 41.9724 7.9797 1

8 5 10 59.9973 8.0252 3

-5 10 -8 68.9626 10.1581 2

10 -8 -5 48.1898 6.5880 1

-8 5 -10 49.7423 9.3412 2

10 8 5 45.9964 7.0928 3

5 10 8 44.2060 6.8972 3

-10 8 -5 33.8459 8.7233 2

5 -10 -8 44.7027 6.7828 1

10 9 -5 54.9480 6.7130 3

-5 10 9 50.3612 9.7741 2

5 10 -9 71.1965 7.0860 3

-10 -9 -5 78.8344 9.1478 1

9 5 -10 67.3213 6.6128 3

-9 5 10 45.9462 11.3515 2

-10 9 5 75.1389 10.6893 2

5 -10 -9 56.0888 7.5100 1

10 9 5 54.5576 7.4694 3

5 10 9 65.5362 8.0044 3

-9 5 -10 65.9671 10.3793 2

-5 10 -9 85.0338 11.3567 2

-10 9 -5 46.6602 9.3083 2

-10 -9 5 48.6258 8.5424 1

9 5 10 50.8507 7.8305 3

10 -9 -5 50.7995 7.0554 1

-10 0 -6 39.3112 6.3151 2

0 6 10 32.9777 5.1784 3

0 -6 10 26.9104 5.0546 3

6 10 0 33.6326 4.7489 3

-10 0 6 39.1712 7.1535 2

-6 10 0 27.9340 6.0496 2

-6 -10 0 45.8891 6.5902 1

6 -10 0 43.5250 5.7188 1

10 0 -6 36.3808 4.7441 1

0 6 -10 40.5691 7.1724 2

0 -6 -10 42.9060 5.4215 2

10 0 6 47.4739 6.9651 3

-6 10 1 229.130 15.873 2

1 -6 10 231.541 14.170 3

-10 -1 -6 231.521 15.299 2

-1 -6 -10 251.688 14.816 2

-6 -10 -1 248.644 15.402 1

10 -1 6 251.904 15.674 3

-1 6 10 207.307 13.596 3

1 6 -10 245.880 16.740 2

10 1 -6 232.846 13.578 1

-10 1 6 205.695 16.515 2

6 10 -1 220.171 13.316 3

6 -10 1 240.143 14.498 1

-1 6 -10 238.059 17.235 2

1 6 10 231.195 14.322 3

10 -1 -6 217.480 13.435 1

-10 1 -6 246.883 16.103 2

-6 10 -1 237.744 16.261 2

-6 -10 1 241.465 15.488 1

6 -10 -1 227.559 14.512 1

10 1 6 250.534 15.993 3

1 -6 -10 221.806 14.918 2

6 10 1 246.708 13.906 3

-1 -6 10 230.393 14.231 3

-10 2 6 977.739 54.105 2

-6 -10 -2 1111.86 50.25 1

6 10 -2 961.831 47.539 3

-2 -6 -10 1046.62 51.49 2

10 -2 6 1237.22 50.37 3

10 2 -6 954.666 48.186 1

-6 10 2 996.082 52.753 2

6 -10 2 1039.76 49.12 1

2 -6 10 999.242 48.424 3

2 6 -10 1031.96 53.25 2

10 2 6 1074.84 49.32 3

6 -10 -2 951.407 48.302 1

6 10 2 1018.04 47.20 3

-10 2 -6 1010.91 52.19 2

10 -2 -6 959.322 47.353 1

-2 6 -10 934.558 53.078 2

-6 10 -2 1018.52 52.23 2

2 6 10 1022.22 47.93 3

2 -6 -10 1069.45 51.20 2

-2 -6 10 1013.50 47.58 3

-6 -10 2 1066.17 49.48 1

3 6 -10 286.147 16.098 3

-3 -6 -10 312.842 17.817 2

-10 3 6 303.006 21.056 2

10 3 -6 301.973 15.616 3

6 10 -3 273.792 16.095 3

-6 10 3 290.647 19.238 2

-6 -10 -3 332.125 18.294 1

3 6 -10 292.705 18.798 2

6 -10 3 305.136 17.224 1

3 -6 10 284.270 17.283 3

-6 10 -3 326.978 19.736 2

6 -10 -3 257.965 16.828 1

-6 -10 3 269.156 17.804 1

-3 6 -10 286.990 20.406 2

10 3 6 309.579 18.391 3

3 6 10 290.010 17.250 3

6 10 3 297.862 16.696 3

3 -6 -10 315.518 18.307 2

-10 3 -6 314.810 19.953 2

-10 -3 6 285.372 17.515 1

10 -3 -6 273.737 16.221 1

-4 -6 -10 6.25606 3.83613 2

10 4 -6 9.18811 3.36076 3

-10 4 6 1.60620 6.12268 2

6 -10 4 11.8166 4.2571 1

4 -6 10 14.5924 4.7223 3

6 10 -4 9.19584 3.42088 3

4 6 -10 14.4298 3.5924 3

-6 -10 -4 10.8298 4.9262 1

-6 10 4 15.4353 5.5131 2

6 10 4 4.63506 3.81514 3

4 -6 -10 2.60566 3.64913 2

10 4 6 8.19724 5.03632 3

-6 -10 4 9.49171 5.01690 1

-4 6 -10 6.48409 5.77346 2

6 -10 -4 5.89711 3.87110 1

10 -4 -6 2.16252 3.22886 1

-10 -4 6 8.79186 4.83856 1

4 6 10 0.11216 3.98223 3

-10 4 -6 0.73846 5.20768 2

-6 10 -4 12.0950 5.6527 2

6 10 -5 142.969 9.864 3

5 -6 10 142.351 11.344 3

-6 10 5 114.344 11.733 2

-6 -10 -5 166.118 11.774 1

10 5 -6 140.690 9.828 3

-10 5 6 134.831 13.887 2

-5 -6 -10 148.126 10.796 2

5 6 -10 139.514 9.594 3

-6 10 -5 178.548 14.257 2

-6 -10 5 152.871 12.016 1

6 10 5 137.571 10.831 3

10 5 6 142.846 11.944 3

-10 -5 6 149.533 12.103 1

-10 5 -6 156.176 13.925 2

10 -5 -6 130.936 10.050 1

5 -6 -10 140.095 11.567 2

5 6 10 142.354 11.341 3

-5 6 -10 138.414 13.560 2

6 -10 -5 129.082 10.673 1

10 6 -6 631.163 32.734 3

-6 -10 -6 770.413 34.327 1

6 -6 10 645.157 34.300 3

6 10 -6 635.670 32.667 3

-6 10 6 641.866 37.574 2

-6 6 10 671.255 39.854 2

-10 6 6 730.344 39.485 2

6 6 -10 681.963 32.727 3

-6 10 -6 729.988 38.853 2

-6 -10 6 618.491 34.702 1

6 -10 -6 676.040 34.366 1

6 6 10 686.663 34.604 3

10 -6 -6 646.952 33.546 1

6 10 6 691.038 34.274 3

-6 6 -10 716.591 39.610 2

-10 -6 6 639.812 35.270 1

-10 6 -6 701.874 39.147 2

10 6 6 671.469 35.077 3

-7 6 10 258.029 19.968 2

-6 10 7 234.001 17.721 2

10 7 -6 212.101 13.582 3

7 -6 10 212.846 15.182 3

6 10 -7 198.580 13.217 3

-10 7 6 224.762 18.187 2

7 6 -10 226.599 13.431 3

-7 6 -10 243.968 18.515 2

-6 10 -7 246.243 17.840 2

6 -10 -7 203.965 14.518 1

-10 -7 6 211.162 15.834 1

-6 -10 7 195.699 15.078 1

10 -7 -6 205.179 13.900 1

-10 7 -6 221.766 17.877 2

6 10 7 221.573 14.630 3

10 7 6 216.738 15.271 3

-6 10 8 12.6617 7.1441 2

6 10 -8 10.2502 3.7805 3

-8 6 10 -0.5029 7.6684 2

10 8 -6 8.23718 3.97354 3

-10 8 6 17.1683 7.9197 2

8 6 -10 16.6761 4.2282 3

8 6 10 6.49749 5.03874 3

10 -8 -6 19.2787 5.2321 1

10 8 6 19.5592 5.9162 3

6 10 8 17.3526 5.3950 3

-10 8 -6 13.2222 6.7506 2

-10 -8 6 22.1549 6.8301 1

-8 6 -10 17.0833 7.4201 2

6 -10 -8 12.1970 5.0838 1

-6 10 -8 21.3998 7.5648 2

9 6 -10 32.7799 5.3644 3

-6 10 9 15.1788 7.6247 2

6 10 -9 30.4722 5.2267 3

-10 9 6 45.2157 9.3758 2

10 9 -6 29.5529 5.4187 3

-9 6 10 32.5893 10.5936 2

6 -10 -9 34.9117 6.5093 1

-9 6 -10 41.1187 9.1925 2

6 10 9 32.8705 6.6283 3

-10 -9 6 40.0487 7.9580 1

10 9 6 30.9210 6.3556 3

9 6 10 37.4839 7.1002 3

-10 9 -6 25.2442 7.9434 2

-6 10 -9 49.0482 9.9497 2

10 -9 -6 33.0696 6.4516 1

10 0 -7 60.9430 6.0268 1

-7 10 0 72.8377 8.3088 2

0 7 -10 52.5374 8.3812 2

-7 -10 0 66.0304 7.8894 1

0 -7 -10 71.1818 7.0221 2

7 -10 0 64.9067 7.0033 1

-10 0 7 53.2812 9.1183 2

7 10 0 59.9145 6.1527 3

10 0 7 66.7508 8.0312 3

0 7 10 49.6008 6.5459 3

-10 0 -7 59.4706 7.7604 2

0 -7 10 63.1803 6.9806 3

10 -1 7 73.9259 8.6078 3

-10 -1 -7 83.2744 8.4039 2

1 7 -10 77.5616 9.0636 2

-10 1 7 88.9580 10.6969 2

7 -10 1 78.2187 7.6045 1

-1 -7 -10 76.1756 7.3842 2

1 -7 10 84.1279 8.0222 3

-7 10 1 76.8916 8.7426 2

7 10 -1 76.0477 6.8393 3

10 1 -7 61.3112 6.3758 1

-7 -10 -1 70.5238 8.2642 1

-1 7 -10 81.1676 9.7656 2

1 7 10 67.4979 7.5292 3

10 1 7 78.2498 8.8450 3

-10 1 -7 65.4184 8.4400 2

-1 -7 10 89.4568 7.9847 3

-7 -10 1 83.0022 8.6707 1

-7 10 -1 82.5521 9.0653 2

7 10 1 80.5488 7.2374 3

10 -1 -7 69.1505 6.5736 1

1 -7 -10 83.4617 7.8440 2

7 -10 -1 65.9232 7.3818 1

7 10 -2 213.512 13.508 3

-7 -10 -2 242.135 15.735 1

2 -7 10 230.348 14.587 3

10 2 -7 217.878 13.609 1

7 -10 2 234.610 14.584 1

10 -2 7 284.191 16.617 3

-2 -7 -10 242.203 14.715 2

-10 2 7 222.366 18.058 2

-7 10 2 235.141 16.648 2

-7 -10 2 245.868 15.952 1

-2 -7 10 228.754 14.740 3

2 7 10 248.221 15.109 3

2 -7 -10 260.806 15.629 2

7 -10 -2 231.073 14.835 1

10 -2 -7 238.186 13.940 1

-10 2 -7 233.238 16.703 2

-2 7 -10 221.472 17.718 2

7 10 2 222.542 14.025 3

10 2 7 229.517 15.957 3

-7 10 -2 235.337 16.745 2

3 7 -10 111.725 8.408 3

-7 10 3 103.217 10.687 2

-10 3 7 99.8353 12.3121 2

3 -7 10 115.240 9.763 3

7 10 -3 96.1265 8.0608 3

-3 -7 -10 111.661 9.027 2

-7 -10 -3 126.265 10.579 1

10 3 -7 97.7620 7.4749 3

7 -10 3 119.605 9.387 1

3 -7 -10 106.672 9.324 2

-10 -3 7 96.7259 9.3042 1

-7 10 -3 103.134 10.614 2

7 -10 -3 87.7832 8.5897 1

10 3 7 117.459 10.295 3

7 10 3 108.606 8.872 3

-10 3 -7 111.307 11.115 2

-7 -10 3 95.0003 9.7149 1

3 7 10 101.237 9.235 3

-3 7 -10 105.388 11.700 2

10 -3 -7 103.858 8.412 1

-7 10 4 36.5768 7.2454 2

7 -10 4 37.4524 5.9039 1

10 4 -7 41.4601 5.0630 3

-7 -10 -4 44.8656 6.7839 1

-10 4 7 43.7341 9.4429 2

4 -7 10 47.3727 6.6602 3

4 7 -10 46.9706 5.5267 3

7 10 -4 49.1188 5.5910 3

-4 7 -10 45.3760 8.7032 2

-10 -4 7 47.7426 7.1724 1

-7 10 -4 41.1125 7.8493 2

10 4 7 57.1113 7.7357 3

10 -4 -7 44.9632 5.8256 1

7 10 4 40.0206 6.0341 3

4 -7 -10 51.2301 6.7981 2

7 -10 -4 47.3209 6.4586 1

4 7 10 40.8935 6.6389 3

-10 4 -7 41.2731 8.0273 2

-7 -10 4 53.9262 7.3501 1

-5 7 10 34.2484 8.6021 2

5 7 -10 40.5800 5.5716 3

10 5 -7 40.4248 5.2896 3

7 10 -5 49.9212 5.7181 3

-7 10 5 43.1679 7.9826 2

5 -7 10 38.4155 6.8673 3

-7 -10 -5 46.5146 7.0977 1

-10 5 7 51.2615 9.8624 2

-7 -10 5 48.0632 7.5602 1

-5 7 -10 35.3425 8.2731 2

7 10 5 41.9554 6.4383 3

-7 10 -5 53.9531 9.0368 2

-10 5 -7 43.3266 8.4542 2

10 -5 -7 49.0782 6.0019 1

10 5 7 54.5728 7.8223 3

-10 -5 7 50.6157 7.6744 1

5 7 10 43.3097 6.8502 3

7 -10 -5 39.6172 6.4407 1

6 7 -10 238.121 13.572 3

-6 7 10 217.894 18.468 2

6 -7 10 226.434 15.524 3

-7 -10 -6 256.576 15.196 1

7 10 -6 193.517 12.995 3

-10 6 7 230.405 19.064 2

-7 10 6 185.709 16.349 2

10 6 -7 194.673 12.990 3

10 -6 -7 219.056 13.854 1

7 -10 -6 206.657 14.247 1

-6 7 -10 202.676 17.636 2

10 6 7 206.079 15.076 3

-7 -10 6 197.531 15.081 1

-7 10 -6 234.961 17.630 2

-10 -6 7 234.918 16.045 1

-10 6 -7 200.561 17.315 2

7 10 6 192.732 14.134 3

7 7 -10 105.520 8.666 3

-7 10 7 98.4087 11.6817 2

-10 7 7 119.431 13.508 2

7 10 -7 99.2892 8.4698 3

10 7 -7 91.4427 8.3229 3

-7 7 10 107.018 13.444 2

7 7 10 94.1651 9.8638 3

-7 10 -7 132.051 12.954 2

-7 -10 7 98.4944 10.4180 1

-10 7 -7 104.510 12.570 2

-7 7 -10 126.449 13.627 2

7 -10 -7 113.027 9.864 1

10 -7 -7 102.704 9.278 1

-10 -7 7 108.126 11.002 1

10 7 7 84.5228 9.8552 3

7 10 7 94.9859 9.6173 3

-7 10 8 27.7967 8.6013 2

10 8 -7 23.4871 5.0490 3

8 7 -10 33.8691 5.4179 3

-10 8 7 34.7537 9.2406 2

-8 7 10 37.6236 10.4454 2

7 10 -8 27.7860 5.0798 3

10 -8 -7 24.6497 5.9632 1

7 10 8 31.2171 6.4864 3

-8 7 -10 40.6934 9.4109 2

8 7 10 33.5219 6.8365 3

-7 10 -8 33.2165 8.6887 2

10 8 7 24.7658 6.6773 3

-10 -8 7 37.2999 7.8376 1

7 -10 -8 29.3625 6.0923 1

-10 8 -7 15.1653 7.8439 2

8 -10 0 8.37951 4.56101 1

10 0 -8 1.06942 3.07816 1

-10 0 -8 8.56969 5.08131 2

10 0 8 5.34635 5.09188 3

8 10 0 11.1763 3.8119 3

0 8 -10 5.31291 5.69167 2

0 -8 -10 3.57732 3.67052 2

0 -8 10 2.46758 4.19359 3

-8 10 0 3.30673 4.99165 2

-10 0 8 7.95421 6.77918 2

-8 -10 0 -0.5297 4.7725 1

-10 1 8 84.4525 11.3404 2

-8 10 1 68.5361 9.1931 2

10 -1 8 103.170 9.867 3

8 10 -1 85.6383 7.4510 3

8 -10 1 77.7440 8.0145 1

1 -8 10 82.0241 8.4073 3

-10 -1 -8 92.5547 9.0318 2

-8 -10 -1 84.0578 9.0953 1

10 1 -8 78.8270 7.2408 1

-1 -8 -10 80.6600 7.8061 2

1 -8 -10 90.6887 8.3164 2

-1 8 -10 68.3661 9.7664 2

1 8 10 69.0811 7.9238 3

8 10 1 88.1670 7.6928 3

10 -1 -8 73.3649 6.8732 1

-10 1 -8 82.9666 9.3148 2

-8 10 -1 79.4680 9.2821 2

10 1 8 93.4925 9.5743 3

-8 -10 1 84.7016 9.0853 1

-1 -8 10 90.1695 8.5103 3

8 -10 -1 65.9194 7.7725 1

-2 -8 -10 9.11127 3.86388 2

-10 2 8 0.64056 6.83524 2

8 -10 2 2.13250 4.24220 1

-8 10 2 3.14285 4.93435 2

8 10 -2 3.16756 2.98261 3

-8 -10 -2 -0.2061 4.6339 1

10 -2 8 10.0129 5.6130 3

2 -8 10 1.51751 3.96001 3

-2 8 -10 6.67387 5.83746 2

-8 -10 2 4.09000 5.39515 1

-8 10 -2 3.83501 5.08796 2

2 8 10 1.55187 4.40166 3

2 -8 -10 3.58998 3.76485 2

-2 -8 10 0.89200 4.03149 3

8 10 2 5.81651 3.74641 3

10 2 8 1.93243 5.10467 3

8 -10 -2 5.48915 4.13435 1

10 -2 -8 4.69099 3.38205 1

-10 2 -8 7.15984 5.13314 2

-3 8 10 75.3256 9.6268 2

-8 10 3 80.6305 9.6387 2

3 -8 10 69.8797 7.7893 3

3 8 -10 62.7291 6.6448 3

8 10 -3 64.5259 6.5473 3

-8 -10 -3 67.1357 8.4731 1

8 -10 3 62.7653 7.2398 1

-10 3 8 69.5988 11.2533 2

10 -3 -8 63.2905 6.5517 1

3 8 10 82.1951 8.3204 3

-10 -3 8 61.6097 7.7668 1

-8 10 -3 54.5567 8.6728 2

8 10 3 64.7427 7.0975 3

-3 8 -10 58.0836 9.4815 2

-8 -10 3 82.4966 9.1033 1

-10 3 -8 69.1561 9.3779 2

10 3 8 51.3310 7.8836 3

8 -10 -3 64.2062 7.4215 1

-8 10 4 15.4011 6.3899 2

8 -10 4 10.6355 4.8511 1

4 8 -10 16.4262 4.1747 3

-10 4 8 12.1039 7.8131 2

8 10 -4 12.4802 4.0991 3

-8 -10 -4 11.1643 5.7239 1

10 4 -8 14.6537 3.7489 3

-4 8 10 7.84371 6.44130 2

4 -8 10 12.9621 5.3732 3

8 -10 -4 10.9250 4.7005 1

4 8 10 24.9245 5.7403 3

10 -4 -8 12.7654 4.2284 1

-10 4 -8 7.26160 6.43071 2

8 10 4 16.6454 4.9253 3

-8 -10 4 4.26148 5.24131 1

-10 -4 8 15.9228 5.9834 1

10 4 8 10.0306 5.3534 3

-4 8 -10 13.8285 6.7675 2

-8 10 -4 7.16392 6.17888 2

-8 10 5 55.8181 9.1130 2

-10 5 8 67.9355 11.1144 2

5 -8 10 43.5444 7.4612 3

-5 8 10 53.5750 9.7866 2

8 10 -5 44.0020 5.7547 3

-8 -10 -5 51.7096 7.5787 1

10 5 -8 50.8431 5.7679 3

5 8 -10 54.4902 6.2676 3

5 8 10 51.2711 7.2049 3

-8 10 -5 52.1983 9.0569 2

8 10 5 49.6792 6.8352 3

-10 5 -8 35.3304 8.5427 2

8 -10 -5 50.0968 6.8519 1

-5 8 -10 39.4842 8.8727 2

-8 -10 5 43.7132 7.5347 1

10 -5 -8 50.3167 6.3635 1

10 5 8 43.3785 7.4700 3

-10 -5 8 52.5353 8.2132 1

10 6 -8 10.4946 4.0222 3

8 10 -6 7.90510 3.85078 3

-8 -10 -6 11.1417 5.7847 1

-8 10 6 5.50085 6.23243 2

6 8 -10 10.7753 3.8975 3

-10 6 8 9.69461 8.00728 2

-6 8 10 11.2719 7.5748 2

-10 6 -8 12.3673 7.2865 2

10 6 8 11.6999 5.5982 3

8 -10 -6 14.6184 5.2348 1

-10 -6 8 12.4401 6.4594 1

-8 -10 6 10.6808 6.3608 1

10 -6 -8 20.4348 5.0011 1

-6 8 -10 16.8105 7.7161 2

-8 10 -6 13.1965 6.5766 2

8 10 6 5.82652 4.56421 3

6 8 10 19.4230 5.7558 3

-8 10 7 39.6975 8.6307 2

10 7 -8 22.8077 5.0040 3

8 10 -7 33.7175 5.3289 3

-7 8 10 31.0136 9.2526 2

7 8 -10 24.5562 5.1892 3

-10 7 8 29.8441 9.8857 2

-7 8 -10 38.1405 9.1730 2

7 8 10 28.5076 6.7485 3

-10 -7 8 25.4314 7.7039 1

8 -10 -7 31.7345 6.3347 1

10 -7 -8 29.8759 6.0202 1

-8 -10 7 39.9337 7.7837 1

-8 10 -7 28.5707 8.0372 2

-10 7 -8 31.8124 8.8872 2

10 7 8 24.4899 6.6120 3

8 10 7 27.4784 6.3882 3

-9 10 0 9.83243 6.00792 2

0 -9 -10 8.92573 4.38290 2

9 -10 0 8.19665 4.61306 1

10 0 -9 9.90706 3.78832 1

10 0 9 11.9301 6.2470 3

-10 0 -9 4.59233 5.19844 2

0 -9 10 4.71935 4.78571 3

9 10 0 10.9531 3.9598 3

-9 -10 0 7.49925 5.62075 1

9 10 -1 25.2509 4.9436 3

9 -10 1 24.3670 5.7119 1

-10 -1 -9 35.3499 6.5166 2

-9 10 1 35.5022 7.5066 2

-1 -9 -10 39.1536 5.7893 2

1 -9 10 32.1319 6.2181 3

-10 1 9 37.5331 9.4067 2

10 1 -9 28.3197 4.9119 1

10 -1 9 49.1339 7.7885 3

-9 -10 -1 37.9225 7.1935 1

-1 9 -10 25.4351 7.5181 2

-9 -10 1 33.4945 7.1234 1

9 10 1 32.9363 5.4255 3

-9 10 -1 30.6321 7.2351 2

-10 1 -9 24.1219 6.4923 2

10 -1 -9 27.3581 4.8971 1

10 1 9 38.6785 7.1899 3

-1 -9 10 31.3685 6.1027 3

9 -10 -1 43.3630 6.4880 1

9 -10 2 48.3944 6.6483 1

-9 10 2 37.9297 7.8305 2

2 -9 10 35.9505 6.5822 3

9 10 -2 43.3284 5.8187 3

-9 -10 -2 46.9368 7.5407 1

-2 9 10 37.6743 8.0364 2

2 9 -10 40.6613 5.6953 3

10 -2 9 49.4513 8.2174 3

-10 2 9 43.0756 10.0085 2

-10 2 -9 47.3749 7.9985 2

-2 9 -10 38.7497 8.7550 2

-10 -2 9 46.3732 7.1155 1

9 -10 -2 34.7270 6.2679 1

2 9 10 48.1791 6.7858 3

-9 10 -2 37.1563 7.7734 2

10 -2 -9 52.9325 6.0028 1

9 10 2 58.2986 6.5981 3

10 2 9 41.8440 7.5858 3

-9 -10 2 40.7941 7.5401 1

3 -9 10 0.58772 4.37888 3

-3 9 10 11.5104 6.2183 2

9 -10 3 9.48189 4.80875 1

9 10 -3 11.0688 4.0213 3

3 9 -10 20.5531 4.5590 3

-10 3 9 18.5464 8.9233 2

-9 10 3 7.68068 6.02146 2

-9 -10 -3 19.2536 6.5328 1

-3 9 -10 17.3331 6.9716 2

-10 3 -9 8.08232 6.21143 2

-9 10 -3 9.65026 6.19657 2

9 10 3 8.66549 4.28570 3

-10 -3 9 9.61773 5.93606 1

10 3 9 2.45590 5.35803 3

10 -3 -9 6.67872 3.90509 1

-9 -10 3 4.77296 5.79665 1

9 -10 -3 9.76267 4.83926 1

3 9 10 23.2683 5.6436 3

-9 -10 -4 7.62001 5.78973 1

9 10 -4 5.45943 3.75142 3

-4 9 10 11.5991 6.8815 2

4 -9 10 7.96346 5.52990 3

4 9 -10 14.0027 4.1273 3

10 4 -9 14.0948 3.7864 3

-10 4 9 16.5355 9.0896 2

-9 10 4 5.61314 6.21585 2

9 10 4 3.52576 4.05891 3

9 -10 -4 5.88939 4.57444 1

-9 -10 4 17.7503 6.4328 1

4 9 10 8.53230 5.07557 3

-4 9 -10 10.0130 6.8197 2

10 4 9 16.1704 5.9453 3

-10 4 -9 9.11796 6.46167 2

10 -4 -9 10.2316 4.3265 1

-9 10 -4 1.08943 6.02520 2

-9 10 5 75.9114 10.5205 2

10 5 -9 63.1351 6.5201 3

-5 9 10 65.7412 10.6164 2

-9 -10 -5 73.0778 8.6837 1

-10 5 9 48.7486 11.3190 2

5 9 -10 58.7479 6.7622 3

9 10 -5 63.3637 6.8050 3

9 10 5 61.3773 7.5835 3

9 -10 -5 53.1261 7.6247 1

-5 9 -10 77.8299 11.0398 2

-9 10 -5 66.2057 10.1727 2

-9 -10 5 69.8429 9.0747 1

10 5 9 63.1891 8.3332 3

10 -5 -9 58.9218 7.1729 1

-10 5 -9 74.3197 11.0402 2

5 9 10 63.5265 8.1191 3

6 9 -10 33.4039 5.5203 3

10 6 -9 34.1077 5.4395 3

9 10 -6 31.2754 5.2958 3

-10 6 9 31.8262 10.1577 2

-6 9 10 33.2662 9.4810 2

-9 10 6 31.5577 8.4199 2

-9 -10 6 44.0729 8.0862 1

-9 10 -6 43.4169 9.0137 2

-10 6 -9 51.2398 9.8906 2

-6 9 -10 42.6496 9.5308 2

9 -10 -6 34.7353 6.5506 1

10 6 9 27.7714 6.8174 3

6 9 10 29.9225 6.6247 3

9 10 6 44.4141 7.0885 3

10 -6 -9 38.5578 6.3810 1

0 -10 10 9.43575 5.60646 3

-10 0 -10 25.6309 6.5806 2

-10 10 0 20.7238 7.3237 2

10 -10 0 24.4971 5.9174 1

10 0 10 18.0901 7.1425 3

10 10 0 24.4602 5.0617 3

-10 -10 0 24.3647 6.9585 1

-10 10 1 173.743 15.395 2

-10 -10 -1 177.531 14.562 1

10 10 -1 182.099 12.516 3

-1 10 10 195.893 15.575 2

-10 1 10 183.374 17.534 2

-10 -1 -10 199.487 14.935 2

1 -10 10 160.681 13.392 3

10 -1 10 216.228 15.345 3

10 -10 1 197.674 13.519 1

10 1 10 185.495 14.438 3

-10 1 -10 184.431 15.003 2

-1 -10 10 171.160 13.572 3

-10 10 -1 187.253 15.834 2

-10 -10 1 180.153 14.521 1

10 10 1 171.788 12.441 3

10 -10 -1 193.938 13.512 1

10 10 -2 423.487 23.875 3

2 -10 10 431.165 25.100 3

-10 -10 -2 488.995 26.684 1

10 -2 10 520.251 27.225 3

-10 2 10 430.705 30.339 2

10 -10 2 486.204 25.309 1

2 10 -10 431.175 23.723 3

-2 10 10 457.831 28.236 2

-10 10 2 462.363 28.462 2

-10 -10 2 468.918 27.305 1

10 10 2 469.189 25.283 3

-10 10 -2 469.372 29.280 2

10 2 10 502.573 27.263 3

-10 2 -10 483.324 28.899 2

10 -10 -2 459.266 25.900 1

10 10 -3 218.852 13.657 3

10 -10 3 220.663 14.668 1

-3 10 10 215.526 17.295 2

3 10 -10 208.604 13.518 3

-10 -10 -3 236.163 16.267 1

-10 10 3 221.404 17.414 2

-10 3 10 204.268 19.600 2

3 -10 10 171.471 14.327 3

-10 3 -10 233.594 17.755 2

-10 -10 3 241.741 16.825 1

-10 10 -3 220.728 17.501 2

10 -10 -3 195.596 14.572 1

10 3 10 220.743 16.162 3

10 10 3 199.334 14.140 3

-3 10 -10 235.369 18.498 2

3 10 10 208.853 14.716 3

-10 -10 -4 41.9766 7.9258 1

10 10 -4 26.3686 5.2038 3

-10 10 4 31.7490 8.6017 2

4 10 -10 34.3104 5.5211 3

-4 10 10 29.5072 8.6030 2

-10 4 10 21.1978 9.6246 2

10 4 10 30.3933 7.2437 3

-10 -10 4 38.1529 7.8939 1

4 10 10 19.8361 5.9825 3

-10 10 -4 28.9292 8.1866 2

-4 10 -10 46.2460 9.5456 2

10 -10 -4 34.1704 6.4201 1

10 10 4 33.9927 6.0510 3

-10 4 -10 45.1480 9.1478 2

0 0 11 -1.4551 2.4093 3

0 11 0 1.33382 2.39663 3

0 0 -11 -2.3743 3.1268 2

11 0 0 -0.9086 2.5363 1

0 -11 0 7.91427 4.02326 1

-11 0 0 -3.1949 3.3926 2

-1 0 -11 8.90257 4.17245 2

0 11 -1 16.9191 3.2567 3

-11 1 0 5.44399 4.54790 2

1 0 -11 4.24665 3.84513 2

0 11 1 14.0163 3.1848 3

0 -11 -1 9.31476 4.25048 1

0 -11 1 8.40083 4.47535 1

11 -1 0 7.58171 3.21295 1

11 1 0 12.6251 3.3989 1

1 0 11 8.72225 3.50425 3

-1 0 11 7.23786 3.05485 3

11 1 0 9.53045 3.46112 3

-2 0 11 5.95928 2.93736 3

11 2 0 5.36399 3.04688 1

0 11 -2 7.98030 2.85048 3

2 0 -11 5.41090 3.79911 2

0 -11 2 5.10399 4.34864 1

2 0 11 7.23480 3.58473 3

11 2 0 3.06444 3.30467 3

0 -11 -2 7.08151 3.84348 1

-2 0 -11 5.01778 3.97173 2

11 -2 0 7.75051 3.12519 1

-11 2 0 0.27573 4.35967 2

-3 0 -11 60.8309 7.3193 2

3 0 -11 46.3420 6.6941 2

3 0 11 62.1981 6.3192 3

11 3 0 39.4557 5.6126 3

0 -11 -3 52.9907 6.4987 1

-3 0 11 45.2517 5.1212 3

0 11 -3 63.0475 5.3154 3

-11 3 0 41.3081 7.3451 2

-11 -3 0 50.9987 6.4325 1

0 -11 3 36.4556 6.4625 1

11 -3 0 54.8288 5.4825 1

-11 4 0 17.2122 5.6835 2

-4 0 -11 3.20731 3.84088 2

-11 -4 0 6.88047 4.27035 1

11 4 0 4.66415 3.77971 3

0 -11 4 9.98778 4.39578 1

4 0 11 5.90094 3.66937 3

4 0 -11 6.89756 4.14454 2

11 -4 0 9.69474 3.39535 1

0 11 -4 4.24596 2.80263 3

0 -11 -4 9.68333 4.10265 1

-4 0 11 6.16012 2.97273 3

0 -11 5 2.00802 4.39893 1

-5 0 -11 12.1025 4.8523 2

-5 0 11 5.60010 3.02992 3

5 0 -11 17.9450 4.7360 2

-11 -5 0 12.6428 4.9950 1

11 5 0 7.23040 4.05624 3

0 11 -5 9.57495 3.16242 3

5 0 11 -0.1545 3.5630 3

0 -11 -5 8.30342 4.26524 1

11 -5 0 2.97263 3.38785 1

-11 5 0 -0.5579 5.1736 2

-6 0 -11 12.8443 5.1878 2

0 -11 -6 6.13483 4.12363 1

11 -6 0 9.06589 3.89447 1

6 0 11 2.61229 4.39952 3

-11 6 0 15.5007 6.3172 2

-11 -6 0 3.00553 4.80673 1

11 6 0 13.7640 4.5300 3

6 0 -11 1.30596 4.30032 2

-6 0 11 11.2253 3.3358 3

0 11 -6 10.5530 3.3974 3

0 -11 6 4.37859 4.56803 1

11 -7 0 109.951 9.284 1

7 0 -11 106.101 10.376 2

-11 -7 0 119.443 11.401 1

0 -11 -7 122.473 10.143 1

7 0 11 111.450 10.286 3

-7 0 -11 121.706 11.487 2

0 11 -7 121.651 8.837 3

11 7 0 115.908 9.607 3

-11 7 0 105.876 11.781 2

8 0 -11 13.6712 5.5074 2

0 -11 -8 20.2866 5.2282 1

-11 -8 0 16.0957 6.5253 1

11 -8 0 14.4643 4.7687 1

-8 0 -11 18.9302 6.0154 2

11 8 0 11.4190 4.5714 3

-11 8 0 24.9539 7.2758 2

8 0 11 17.1306 6.0527 3

0 11 -8 9.79245 3.80149 3

-11 9 0 27.2663 7.7517 2

-9 0 -11 30.3124 7.0303 2

11 9 0 26.8165 5.5447 3

-11 -9 0 36.7637 7.7693 1

0 -11 9 26.4089 6.5694 3

11 -9 0 36.0283 6.3408 1

9 0 11 30.1833 7.4705 3

-9 0 11 24.8967 9.8492 2

10 0 11 4.86086 6.58309 3

11 10 0 11.1502 4.7690 3

0 -11 10 9.13712 5.90140 3

-11 -10 0 4.70933 6.72710 1

11 -10 0 23.8745 5.9637 1

-10 0 -11 9.59305 6.00774 2

-11 10 0 15.6103 7.3201 2

11 0 1 10.3750 3.3838 1

0 1 -11 10.1029 4.4994 2

-11 0 1 15.2185 4.4726 2

0 1 11 11.3882 3.5217 3

0 -1 -11 14.6487 4.3650 2

11 0 -1 6.08340 3.09047 1

1 11 0 10.4299 3.1357 3

1 -11 0 7.49387 4.09774 1

0 -1 11 12.2921 3.5163 3

-11 0 -1 11.0597 4.2141 2

-1 -11 0 3.22101 3.76220 1

11 -1 1 389.899 19.117 1

1 1 -11 333.782 21.765 2

11 1 -1 406.838 19.488 1

1 -11 1 363.666 20.616 1

1 -1 11 381.526 19.535 3

-11 1 1 325.255 21.025 2

-1 -1 -11 352.193 21.619 2

1 11 -1 384.138 18.703 3

-1 -11 -1 362.355 20.587 1

-1 1 11 349.944 18.953 3

-1 -11 1 367.959 20.612 1

11 1 1 374.959 18.986 1

-1 -1 11 364.767 18.950 3

11 1 1 345.362 19.746 3

1 11 1 392.063 18.547 3

-1 1 -11 331.132 21.700 2

1 1 11 358.828 19.320 3

1 -11 -1 358.308 20.478 1

-11 1 -1 341.476 21.179 2

11 -1 -1 376.072 19.063 1

1 -1 -11 365.255 21.554 2

11 2 -1 250.817 13.736 1

11 2 -1 223.064 13.483 3

-1 -11 -2 242.622 14.784 1

-2 -1 -11 223.182 15.541 2

2 -1 11 213.848 13.607 3

2 1 -11 216.541 15.585 2

1 11 -2 256.448 13.171 3

-11 2 1 222.667 15.695 2

11 -2 1 223.283 13.128 1

-2 1 11 237.818 13.272 3

1 -11 2 237.025 14.955 1

1 -11 -2 229.359 14.830 1

11 2 1 258.546 13.887 1

-2 1 -11 228.861 16.149 2

-1 -11 2 241.622 15.263 1

2 -1 -11 220.024 15.452 2

11 2 1 215.971 14.569 3

-11 2 -1 224.815 15.810 2

11 -2 -1 246.924 13.637 1

-2 -1 11 235.656 13.480 3

2 1 11 233.433 14.067 3

-1 11 -2 263.565 13.087 3

1 11 2 243.365 13.270 3

11 -3 1 194.007 11.701 1

-3 1 11 202.497 11.682 3

-11 -3 -1 181.976 12.811 1

3 1 -11 193.423 14.101 2

-1 -11 -3 209.130 13.255 1

3 -1 11 170.376 12.281 3

11 3 -1 181.143 12.053 3

-3 -1 -11 201.879 14.205 2

-11 3 1 187.197 14.364 2

1 -11 3 189.046 13.145 1

1 11 -3 221.564 11.744 3

-3 1 -11 202.019 14.559 2

1 -11 -3 198.592 13.406 1

11 3 1 185.694 13.000 3

-1 11 -3 198.821 11.287 3

1 11 3 213.982 11.778 3

-3 -1 11 202.192 11.707 3

-11 3 -1 189.516 14.608 2

-1 -11 3 182.318 13.345 1

3 -1 -11 189.792 13.849 2

-11 -3 1 199.557 13.217 1

11 -3 -1 208.994 12.114 1

3 1 11 190.287 12.521 3

-4 -1 -11 0.61078 3.89116 2

-11 4 1 4.74608 5.11506 2

1 11 -4 11.3777 3.1918 3

11 4 -1 3.67757 3.43447 3

-1 -11 -4 9.54514 4.00618 1

-11 -4 -1 3.60990 4.27569 1

4 1 -11 7.67739 4.21415 2

4 -1 11 0.90259 3.46322 3

1 -11 4 7.20787 4.38476 1

11 -4 1 5.88242 3.22999 1

-4 1 11 11.4458 3.1490 3

4 1 11 3.12660 3.57192 3

-4 1 -11 5.78342 4.10146 2

-11 4 -1 4.63356 4.91349 2

1 11 4 6.52219 3.12998 3

11 4 1 2.01610 3.64179 3

-1 -11 4 9.59007 4.56444 1

-11 -4 1 2.65540 4.02713 1

4 -1 -11 7.94387 4.04973 2

1 -11 -4 9.98350 4.00769 1

-1 11 -4 4.51684 2.83218 3

-4 -1 11 6.28359 2.79686 3

11 -4 -1 13.8264 3.6428 1

5 -1 11 274.134 16.311 3

-5 -1 -11 289.676 18.114 2

-11 -5 -1 276.103 17.300 1

1 11 -5 301.114 15.141 3

1 -11 5 246.110 16.213 1

-11 5 1 230.728 18.220 2

11 5 -1 256.228 15.741 3

11 -5 1 268.030 15.329 1

-1 -11 -5 281.122 16.521 1

-5 1 11 264.593 14.665 3

5 1 -11 261.515 17.510 2

5 -1 -11 275.428 17.771 2

5 1 11 246.444 15.920 3

11 5 1 288.346 16.659 3

-5 1 -11 286.539 18.475 2

11 -5 -1 268.827 15.432 1

-11 -5 1 261.679 17.179 1

-1 11 -5 289.534 14.744 3

1 -11 -5 270.005 16.503 1

-11 5 -1 230.283 18.140 2

-1 -11 5 271.451 16.829 1

-5 -1 11 250.104 14.366 3

1 11 -6 187.338 11.052 3

11 6 -1 151.782 11.465 3

-1 -11 -6 180.984 12.316 1

-11 6 1 162.027 14.209 2

-11 -6 -1 176.241 13.426 1

11 -6 1 193.416 11.684 1

6 -1 11 168.969 12.346 3

-6 -1 -11 163.064 13.459 2

6 1 -11 151.775 12.831 2

1 -11 6 166.648 12.296 1

-11 6 -1 158.164 14.083 2

-6 1 -11 179.803 13.927 2

6 -1 -11 175.699 13.333 2

-11 -6 1 145.529 12.670 1

-1 -11 6 175.638 12.803 1

11 -6 -1 167.264 11.421 1

-6 -1 11 150.627 10.156 3

11 6 1 176.565 12.274 3

1 -11 -6 169.072 12.127 1

-1 11 -6 173.169 10.771 3

6 1 11 182.106 12.548 3

7 -1 11 132.597 11.206 3

-1 -11 -7 124.385 10.231 1

1 11 -7 127.413 9.108 3

7 1 -11 130.360 11.302 2

11 7 -1 122.178 9.980 3

-7 -1 -11 120.094 11.352 2

11 -7 1 134.594 10.012 1

-11 7 1 115.215 12.255 2

-11 -7 -1 119.109 11.348 1

-1 11 -7 126.345 9.047 3

11 -7 -1 134.978 9.846 1

7 1 11 124.576 10.842 3

-7 1 -11 138.337 12.351 2

-11 -7 1 122.160 11.682 1

-11 7 -1 119.737 12.406 2

1 -11 -7 109.259 10.173 1

7 -1 -11 113.368 11.110 2

11 7 1 134.001 10.401 3

-7 -1 11 115.881 8.408 3

1 -11 8 5.81295 4.55541 3

1 11 -8 8.43140 3.84816 3

8 -1 11 15.6563 5.8155 3

11 8 -1 13.3160 4.6121 3

-11 -8 -1 7.90383 5.84412 1

-8 -1 -11 9.57488 5.00833 2

-11 8 1 14.6871 6.5912 2

11 -8 1 5.49572 4.58680 1

8 1 -11 8.72329 5.04846 2

-1 -11 -8 7.58063 4.33870 1

-11 8 -1 8.68192 6.47153 2

11 -8 -1 5.74602 4.27239 1

-8 1 -11 9.33102 5.39822 2

8 -1 -11 8.84932 4.91952 2

1 -11 -8 12.3352 4.7832 1

-11 -8 1 12.2638 6.1277 1

11 8 1 6.55592 4.19586 3

8 1 11 9.36992 5.70472 3

11 9 -1 184.494 13.131 3

9 -1 11 200.488 15.461 3

-11 9 1 208.338 16.767 2

-9 -1 -11 199.490 15.306 2

-9 1 11 155.804 17.625 2

1 11 -9 223.577 13.069 3

1 -11 9 183.370 14.080 3

11 -9 1 218.575 14.130 1

-11 9 -1 188.290 16.674 2

11 -9 -1 227.827 14.631 1

11 9 1 200.405 13.890 3

-9 1 -11 217.301 16.437 2

-11 -9 1 212.385 16.079 1

9 1 11 218.285 15.987 3

1 -11 10 75.7882 9.5338 3

11 -10 1 88.8949 9.0863 1

-11 -10 -1 76.7541 10.1335 1

-10 1 11 82.8859 12.7646 2

11 10 -1 72.4458 8.0929 3

10 -1 11 108.273 11.323 3

-11 -10 1 70.7192 9.9741 1

10 1 11 80.0517 10.3082 3

-1 -11 10 81.8061 9.3982 3

-10 1 -11 97.4806 10.8171 2

11 -10 -1 87.2311 9.0632 1

-11 10 -1 65.0604 10.5686 2

0 -2 11 3.87397 3.17006 3

11 0 -2 1.15524 2.76960 1

2 -11 0 3.99755 4.04712 1

-11 0 2 2.06875 3.96750 2

11 0 2 -2.3581 2.7179 1

-2 11 0 6.55649 3.92000 2

0 2 11 11.3282 3.4787 3

0 2 -11 1.21755 3.89463 2

2 11 0 3.65912 2.79650 3

-11 0 -2 2.44694 3.91039 2

-2 -11 0 2.16549 3.46378 1

0 -2 -11 8.47664 3.80301 2

1 2 -11 219.381 15.926 2

11 1 -2 225.900 13.335 1

2 -11 1 249.662 15.144 1

-11 1 2 226.373 15.504 2

11 -1 2 237.361 13.175 1

-1 -2 -11 212.977 15.074 2

1 -2 11 229.002 13.674 3

2 11 -1 238.060 13.029 3

-1 2 11 239.072 13.605 3

-2 -11 -1 225.887 14.614 1

-2 11 1 255.719 15.025 2

2 11 1 246.350 13.266 3

-2 -11 1 225.147 14.761 1

11 1 2 212.885 14.812 3

2 -11 -1 235.393 14.823 1

11 -1 -2 236.517 13.530 1

-2 11 -1 270.980 15.737 2

-1 2 -11 236.697 16.497 2

11 1 2 254.259 13.648 1

-1 -2 11 221.007 13.486 3

-11 1 -2 237.026 15.534 2

1 -2 -11 223.225 15.285 2

1 2 11 235.523 13.866 3

2 -11 2 14.9740 4.9324 1

2 2 -11 18.2478 5.0355 2

11 -2 2 25.3274 3.8943 1

-2 2 11 20.5193 4.0224 3

-2 -2 -11 28.3736 5.3775 2

-2 -11 -2 24.4625 4.9806 1

-11 2 2 17.3413 5.4440 2

11 2 -2 17.8838 3.8578 1

-11 -2 -2 18.0719 4.5061 1

2 -2 11 19.3719 4.3110 3

-2 11 2 18.1978 4.4292 2

11 2 -2 19.9043 3.8974 3

2 11 -2 19.4917 3.7787 3

-2 2 -11 17.4665 5.2943 2

-2 -2 11 23.6500 3.9828 3

11 2 2 16.7987 5.0714 3

2 -11 -2 21.5774 4.7209 1

-2 -11 2 19.5132 5.0630 1

11 -2 -2 22.3849 3.9262 1

2 2 11 26.1332 4.5866 3

-11 2 -2 18.5459 5.4795 2

-2 11 -2 23.7321 5.2294 2

2 -2 -11 15.8769 4.5253 2

2 11 2 22.6857 3.8965 3

-11 3 2 253.156 17.776 2

3 2 -11 241.710 17.260 2

-11 -3 -2 252.493 16.019 1

2 -11 3 270.694 16.458 1

-3 -2 -11 261.090 17.201 2

11 3 -2 257.665 15.023 3

2 11 -3 299.110 14.941 3

-2 11 3 261.458 16.407 2

-3 2 11 289.150 15.015 3

11 -3 2 271.369 14.873 1

3 -2 11 264.875 15.855 3

-2 -11 -3 285.428 16.502 1

11 3 2 268.911 16.712 3

-3 2 -11 268.214 17.985 2

2 -11 -3 280.151 16.531 1

2 11 3 293.956 14.812 3

3 -2 -11 273.117 17.071 2

-11 -3 2 264.983 16.257 1

11 -3 -2 255.856 14.877 1

-3 -2 11 262.213 14.616 3

-11 3 -2 239.720 17.513 2

3 2 11 269.785 15.621 3

-2 -11 3 247.601 16.334 1

2 11 -4 7.68780 3.14782 3

-11 4 2 3.48705 5.11097 2

11 4 -2 3.77508 3.24851 3

2 -11 4 7.73465 4.28782 1

-4 -2 -11 4.80618 4.02460 2

-2 11 4 4.16751 3.92427 2

-11 -4 -2 6.30047 4.25787 1

11 -4 2 1.67889 3.06374 1

-2 -11 -4 5.31280 3.82199 1

4 -2 11 2.95852 3.81651 3

-4 2 11 4.71916 3.04366 3

4 2 -11 9.71689 4.46923 2

4 2 11 4.08640 3.84356 3

-4 -2 11 4.30997 2.91159 3

11 -4 -2 6.49599 3.32117 1

-11 4 -2 8.84647 5.35929 2

-11 -4 2 3.35906 4.16157 1

4 -2 -11 7.10239 3.88395 2

-4 2 -11 0.56629 4.24334 2

11 4 2 6.35310 4.28918 3

-2 -11 4 7.46410 4.56614 1

2 11 4 2.84906 2.98737 3

2 -11 -4 6.62785 3.86699 1

-2 11 5 159.194 12.139 2

11 -5 2 151.557 10.631 1

2 11 -5 172.871 10.675 3

5 -2 11 159.145 11.744 3

5 2 -11 168.962 12.845 2

-11 5 2 145.724 13.596 2

-11 -5 -2 150.975 12.287 1

-5 -2 -11 166.002 12.631 2

11 5 -2 142.057 10.843 3

-2 -11 -5 200.099 12.278 1

2 -11 5 164.934 11.810 1

-5 -2 11 161.682 10.058 3

-2 -11 5 138.581 11.543 1

5 -2 -11 159.242 12.308 2

-11 -5 2 130.597 11.917 1

2 -11 -5 157.043 11.411 1

2 11 5 169.213 10.609 3

11 -5 -2 156.499 10.613 1

-5 2 -11 174.311 13.472 2

5 2 11 141.163 11.084 3

-11 5 -2 143.639 13.530 2

11 5 2 144.201 11.684 3

11 -6 2 102.667 8.535 1

6 2 -11 92.7592 9.2491 2

-2 11 6 100.904 9.608 2

6 -2 11 85.1874 8.9304 3

-11 6 2 110.206 11.233 2

-6 -2 -11 94.9668 9.6272 2

-2 -11 -6 107.015 9.330 1

11 6 -2 93.9363 8.4099 3

-11 -6 -2 104.650 10.126 1

2 11 -6 96.8673 7.7863 3

11 6 2 92.8191 8.9021 3

-11 -6 2 82.7248 9.6586 1

-6 2 -11 83.2891 9.8357 2

-6 -2 11 92.5156 7.4664 3

-11 6 -2 99.6266 11.2209 2

6 -2 -11 112.580 10.230 2

11 -6 -2 97.0604 8.3011 1

2 -11 -6 90.5061 8.8080 1

6 2 11 109.016 9.364 3

2 11 6 106.862 8.618 3

-2 -11 6 77.5241 8.7809 1

-2 11 7 259.195 17.463 2

-11 7 2 269.041 19.118 2

2 -11 7 234.467 15.377 3

7 -2 11 258.676 16.584 3

-2 -11 -7 274.917 16.015 1

-11 -7 -2 246.479 16.947 1

11 -7 2 251.660 15.369 1

-7 -2 -11 263.777 17.442 2

7 2 -11 250.341 16.985 2

11 7 -2 234.841 15.241 3

2 11 -7 256.586 14.680 3

-7 -2 11 216.206 13.334 3

7 -2 -11 245.313 16.712 2

2 -11 -7 239.478 15.255 1

11 7 2 224.242 15.144 3

11 -7 -2 234.899 14.734 1

-7 2 -11 243.592 17.514 2

7 2 11 259.105 16.185 3

-11 7 -2 227.249 18.040 2

-11 -7 2 245.332 16.757 1

-8 -2 -11 5.89557 4.90571 2

-2 11 8 5.60009 5.33092 2

-8 2 11 2.70465 7.56927 2

11 -8 2 1.16584 4.27739 1

-11 8 2 7.53296 6.50271 2

2 11 -8 4.00746 3.56669 3

-11 -8 -2 5.70926 5.42309 1

2 -11 8 5.95122 4.76187 3

11 8 -2 4.70772 3.97100 3

8 -2 11 -0.2622 5.0730 3

-8 2 -11 5.29387 5.51409 2

-11 8 -2 3.91429 6.44801 2

11 8 2 6.72628 4.26795 3

2 -11 -8 9.24752 4.70899 1

8 2 11 1.80796 5.02771 3

11 -8 -2 0.11597 3.96260 1

-11 -8 2 5.31362 5.72781 1

2 -11 9 60.6377 8.2248 3

9 -2 11 89.3200 10.0545 3

11 9 -2 64.7675 7.3067 3

-11 9 2 62.2321 10.2705 2

-9 -2 -11 64.7362 8.6234 2

-11 -9 -2 76.3180 9.7269 1

-9 2 11 48.6035 11.5459 2

11 -9 2 65.6413 7.8199 1

-2 11 9 59.1516 9.1722 2

2 11 -9 71.5243 7.0776 3

11 9 2 74.8184 8.1243 3

9 2 11 74.5410 9.4099 3

-11 -9 2 73.5254 9.9320 1

11 -9 -2 71.8843 8.1844 1

-9 2 -11 64.9132 9.7999 2

-11 9 -2 79.0251 10.5761 2

0 3 11 56.3591 5.9576 3

0 3 -11 39.9814 6.9120 2

3 -11 0 65.5189 7.0533 1

-3 -11 0 61.6276 6.8127 1

-11 0 -3 48.7929 6.4403 2

-11 0 3 56.6339 7.0280 2

-3 11 0 56.7608 6.7279 2

0 -3 -11 56.8110 6.5625 2

3 11 0 55.5703 5.3677 3

0 -3 11 39.7799 5.3691 3

11 0 3 57.0540 5.5389 1

11 0 3 55.7435 7.3666 3

11 0 -3 57.4124 5.5536 1

11 1 -3 190.702 11.722 1

-1 -3 -11 184.280 13.198 2

1 3 -11 183.765 14.297 2

11 -1 3 194.370 11.466 1

1 -3 11 206.930 12.417 3

-1 3 11 194.050 11.894 3

-3 -11 -1 171.260 12.491 1

-3 11 1 216.171 13.507 2

3 11 -1 203.756 11.590 3

-11 1 3 179.702 13.970 2

3 -11 1 196.019 13.210 1

11 1 3 205.189 14.158 3

-3 11 -1 220.633 14.156 2

-3 -11 1 199.290 13.449 1

-11 1 -3 177.887 13.717 2

3 11 1 196.137 11.646 3

11 -1 -3 199.481 12.005 1

1 -3 -11 198.300 13.520 2

1 3 11 188.900 12.218 3

-1 3 -11 196.452 14.765 2

3 -11 -1 198.756 13.243 1

11 1 3 199.163 11.807 1

-1 -3 11 196.173 12.043 3

-2 -3 -11 264.802 16.952 2

11 -2 3 270.718 14.835 1

-11 2 3 255.013 17.714 2

11 2 -3 275.538 15.239 1

2 -3 11 269.552 15.782 3

2 3 -11 243.611 17.726 2

3 -11 2 292.054 16.756 1

3 11 -2 279.755 14.930 3

-3 -11 -2 270.805 16.284 1

-11 -2 -3 258.890 15.845 1

-2 3 11 284.252 15.189 3

-3 11 2 279.027 16.898 2

-2 3 -11 260.195 18.089 2

-3 11 -2 321.524 17.965 2

3 -11 -2 246.515 16.003 1

11 2 3 289.009 17.375 3

2 3 11 252.318 15.362 3

-3 -11 2 264.153 16.376 1

-11 2 -3 250.040 17.529 2

-2 -3 11 272.268 14.998 3

3 11 2 271.171 14.804 3

2 -3 -11 261.786 16.739 2

11 -2 -3 264.381 14.961 1

3 -3 11 168.175 12.132 3

-3 -3 -11 198.297 13.489 2

11 -3 3 190.248 11.221 1

-3 11 3 164.549 12.765 2

-3 3 11 183.912 11.356 3

3 3 -11 183.644 13.839 2

-3 -11 -3 188.654 12.579 1

3 11 -3 189.332 11.365 3

-11 3 3 174.605 14.296 2

11 3 -3 172.627 11.200 3

-11 -3 -3 204.182 12.924 1

3 -11 3 195.016 12.859 1

-3 11 -3 229.161 14.743 2

-3 -11 3 181.660 12.916 1

-11 -3 3 187.956 12.721 1

11 -3 -3 178.021 11.360 1

3 3 11 177.815 11.958 3

-3 3 -11 175.685 14.253 2

-11 3 -3 179.565 14.339 2

3 11 3 179.327 11.209 3

-3 -3 11 176.940 11.256 3

11 3 3 171.642 13.353 3

3 -11 -3 183.586 12.706 1

3 -3 -11 178.895 12.939 2

11 4 -3 58.8278 6.0328 3

3 11 -4 62.9937 5.9493 3

-11 4 3 49.3839 8.0287 2

-11 -4 -3 62.6688 7.3361 1

-3 -11 -4 68.8416 7.1550 1

11 -4 3 61.9470 5.8816 1

-4 -3 -11 62.0509 7.2402 2

3 -11 4 62.9894 7.0608 1

4 3 -11 46.3166 7.0608 2

4 -3 11 42.7116 6.5149 3

-3 11 4 52.4799 6.9638 2

-11 -4 3 55.9684 7.4215 1

11 4 3 55.7078 7.7640 3

11 -4 -3 49.6105 5.7924 1

3 -11 -4 58.2767 7.0126 1

3 11 4 56.1143 5.9244 3

-3 11 -4 68.5273 8.3712 2

4 -3 -11 69.0514 7.6483 2

4 3 11 77.9982 7.3632 3

-4 -3 11 62.2376 6.0415 3

-3 -11 4 57.7859 7.3605 1

-11 4 -3 64.0150 8.7260 2

-4 3 -11 62.0094 8.6134 2

5 3 -11 251.428 17.041 2

-3 11 5 254.980 16.992 2

-11 -5 -3 258.631 16.852 1

3 11 -5 267.846 14.874 3

5 -3 11 262.870 16.323 3

-3 -11 -5 310.882 16.664 1

-11 5 3 232.775 18.271 2

11 5 -3 228.462 14.930 3

-5 -3 -11 265.991 17.184 2

3 -11 5 274.921 15.933 1

11 -5 3 276.780 15.175 1

3 -11 -5 219.224 15.565 1

11 -5 -3 275.947 15.300 1

-5 3 -11 257.038 18.319 2

-3 11 -5 322.742 18.941 2

-11 5 -3 227.519 17.898 2

-11 -5 3 248.865 16.808 1

3 11 5 276.506 15.203 3

-5 -3 11 261.920 14.689 3

5 3 11 260.589 16.044 3

11 5 3 260.780 16.842 3

5 -3 -11 252.988 17.042 2

-3 -11 5 235.242 16.281 1

-3 -11 -6 210.827 13.328 1

-11 -6 -3 173.197 13.897 1

6 -3 11 204.963 13.815 3

3 11 -6 213.605 12.231 3

-11 6 3 190.133 15.887 2

-3 11 6 174.697 14.179 2

11 -6 3 187.009 12.161 1

6 3 -11 176.859 13.766 2

11 6 -3 180.007 12.339 3

-6 -3 -11 204.150 14.169 2

-6 -3 11 186.775 11.678 3

6 3 11 185.034 13.313 3

11 -6 -3 186.634 12.319 1

-3 11 -6 204.998 15.638 2

-3 -11 6 188.508 13.645 1

11 6 3 206.943 13.918 3

-11 -6 3 181.790 14.094 1

3 11 6 186.726 12.487 3

-6 3 -11 191.983 15.427 2

3 -11 -6 184.226 13.120 1

-11 6 -3 183.737 15.591 2

6 -3 -11 169.841 13.872 2

-11 7 3 69.5477 9.8789 2

11 -7 3 59.9566 6.7550 1

11 7 -3 53.2963 6.5630 3

-3 -11 -7 62.0977 7.0045 1

7 -3 11 57.7609 8.0453 3

-11 -7 -3 54.7446 8.3083 1

-7 -3 -11 49.5692 7.3336 2

3 11 -7 60.8334 6.3058 3

-3 11 7 58.8484 8.3993 2

3 -11 -7 39.3599 6.4899 1

-3 11 -7 56.4590 9.0179 2

-11 -7 3 51.9184 8.2822 1

7 3 11 75.2777 8.4025 3

-11 7 -3 45.7655 8.7451 2

-7 -3 11 47.5104 5.7151 3

11 7 3 50.5812 7.1558 3

3 11 7 53.9285 6.6971 3

7 -3 -11 40.8945 7.2582 2

11 -7 -3 58.1855 6.6014 1

-7 3 -11 51.6140 8.7891 2

-11 -8 -3 35.3977 7.2441 1

-3 11 8 26.5346 7.1540 2

11 8 -3 36.9901 5.6166 3

11 -8 3 31.2673 5.9383 1

-8 3 11 40.8844 10.1674 2

-11 8 3 26.2765 7.7380 2

8 -3 11 29.5876 6.9801 3

-8 -3 -11 27.2931 6.4523 2

3 11 -8 43.5325 5.5931 3

3 11 8 30.7483 6.1044 3

11 -8 -3 28.0176 5.5785 1

8 3 11 39.1139 7.2278 3

-11 -8 3 46.8438 8.3493 1

3 -11 -8 29.3675 5.9022 1

-8 3 -11 32.0349 7.7127 2

-11 8 -3 23.6454 7.4201 2

-8 -3 11 31.9199 4.9042 3

11 8 3 30.1758 5.9831 3

-11 -9 -3 142.008 12.824 1

-3 11 9 152.545 14.153 2

11 9 -3 135.216 10.828 3

9 -3 11 186.394 14.136 3

-11 9 3 166.858 15.253 2

11 -9 3 142.744 11.700 1

3 11 -9 154.630 10.972 3

-9 3 11 124.940 16.102 2

-11 9 -3 146.237 14.287 2

11 -9 -3 158.480 12.048 1

-9 3 -11 178.063 15.163 2

-11 -9 3 153.381 13.662 1

11 9 3 140.585 11.537 3

9 3 11 138.371 12.656 3

-4 -11 0 3.92367 4.22155 1

-11 0 -4 3.77559 4.01672 2

-4 11 0 11.5315 4.3482 2

4 11 0 5.60721 2.93376 3

11 0 -4 10.1546 3.2669 1

4 -11 0 2.92682 3.73729 1

11 0 4 11.7395 3.1814 1

11 0 4 6.93681 4.86497 3

0 4 -11 8.75005 4.81635 2

0 4 11 8.10553 3.58354 3

0 -4 11 3.72730 3.30236 3

0 -4 -11 8.54789 3.66272 2

1 4 -11 4.21286 4.48477 2

-4 11 1 6.00631 4.12425 2

-11 -1 -4 3.30318 3.52558 1

-1 4 11 4.94108 3.29956 3

-1 -4 -11 5.30064 3.76444 2

-11 1 4 10.3120 5.1585 2

1 -4 11 2.05398 3.27702 3

4 -11 1 -0.7525 3.8775 1

11 1 -4 7.73744 3.23318 1

-4 -11 -1 5.22846 4.19475 1

11 -1 4 10.0439 3.1289 1

4 11 -1 3.13254 2.67445 3

11 1 4 8.44951 4.96264 3

-1 -4 11 6.19421 3.23506 3

-1 4 -11 4.46814 4.51689 2

4 11 1 13.2808 3.4461 3

1 -4 -11 8.00456 3.79886 2

4 -11 -1 8.54567 4.18390 1

-11 1 -4 11.0600 4.7675 2

11 -1 -4 1.55940 2.78491 1

-4 -11 1 9.79304 4.55615 1

-4 11 -1 10.6491 4.4326 2

1 4 11 6.31975 3.62004 3

2 -4 11 7.82400 3.72379 3

4 11 -2 6.46577 3.02398 3

2 4 -11 5.80752 4.54880 2

-4 11 2 7.30508 4.22680 2

-2 -4 -11 8.56635 3.94411 2

-2 4 11 5.56593 3.34103 3

11 -2 4 2.03256 2.76098 1

-4 -11 -2 3.53203 4.07978 1

-11 2 4 6.49825 5.33889 2

-11 -2 -4 3.90737 3.85924 1

11 2 -4 6.71789 3.33251 1

4 -11 2 1.77715 4.10385 1

2 4 11 0.86417 3.40311 3

11 -2 -4 5.99641 3.13697 1

-4 -11 2 7.10965 4.67876 1

-2 4 -11 8.26283 5.04007 2

-2 -4 11 7.45170 3.56044 3

2 -4 -11 2.09694 3.11255 2

-11 2 -4 2.52316 4.76087 2

-4 11 -2 18.1690 5.3156 2

4 -11 -2 3.71718 3.91789 1

4 11 2 3.67875 3.03287 3

11 2 4 1.11246 4.63513 3

-4 11 3 49.8919 6.9979 2

11 3 -4 47.2448 5.4551 3

-3 -4 -11 55.5239 6.8979 2

3 -4 11 54.3953 6.7631 3

3 4 -11 56.9697 7.5701 2

11 -3 4 57.4076 5.6203 1

4 11 -3 66.6956 5.9707 3

-11 3 4 47.2574 8.0270 2

-4 -11 -3 60.6250 6.8978 1

-11 -3 -4 61.6037 7.0709 1

4 -11 3 67.0496 7.3086 1

-11 -3 4 61.9812 7.3790 1

-11 3 -4 52.0262 8.0709 2

-3 4 -11 62.8525 8.5221 2

11 -3 -4 53.8764 5.7862 1

-4 -11 3 62.4956 7.6262 1

3 -4 -11 65.1040 7.2525 2

11 3 4 61.1677 7.9181 3

4 11 3 54.8803 5.8027 3

-3 -4 11 62.3879 6.3021 3

4 -11 -3 50.1141 6.7390 1

-4 11 -3 61.2298 8.0127 2

3 4 11 51.2901 6.4814 3

4 11 -4 9.74956 3.32229 3

-11 -4 -4 5.55038 4.67583 1

4 4 -11 5.60123 4.55088 2

-11 4 4 6.44645 5.77512 2

-4 -4 -11 4.19979 4.10974 2

-4 11 4 -0.7212 4.0319 2

11 4 -4 3.35016 3.20278 3

11 -4 4 6.69438 3.34676 1

-4 -11 -4 1.62900 4.08562 1

4 -4 11 13.0313 4.7646 3

4 -11 4 6.76813 4.36696 1

-4 11 -4 18.3249 5.9191 2

-4 4 -11 7.62770 5.09287 2

11 -4 -4 8.24937 3.50000 1

4 -4 -11 3.53951 3.75816 2

-11 4 -4 11.1344 5.9189 2

-4 -4 11 8.08990 3.60323 3

-4 -11 4 4.93878 4.79562 1

11 4 4 -0.2635 4.4482 3

4 11 4 6.30101 3.47281 3

4 -11 -4 4.59879 3.93832 1

-11 -4 4 5.65259 4.57212 1

4 4 11 5.48680 3.88893 3

11 -5 4 9.30882 3.76801 1

5 4 -11 6.67326 4.47411 2

-5 -4 -11 4.74679 4.37675 2

-4 -11 -5 10.3532 4.4426 1

-4 11 5 5.61132 4.60545 2

4 11 -5 6.74775 3.21793 3

5 -4 11 5.68860 4.70820 3

11 5 -4 6.39474 3.58688 3

-11 5 4 12.9510 6.2403 2

4 -11 5 4.60510 4.23819 1

-11 -5 -4 7.14728 4.93819 1

5 -4 -11 4.83413 4.04625 2

-4 -11 5 1.77806 4.98404 1

-5 4 -11 8.68823 5.49839 2

-11 -5 4 8.60118 5.30209 1

11 -5 -4 6.66186 3.57634 1

4 -11 -5 3.33388 4.13206 1

4 11 5 4.40998 3.60731 3

-11 5 -4 13.5266 6.2649 2

-4 11 -5 8.43012 5.16995 2

-5 -4 11 1.99568 3.21215 3

5 4 11 8.40011 4.48513 3

11 5 4 10.8609 5.4177 3

-11 -6 -4 11.3857 5.4066 1

6 -4 11 -2.2911 4.5341 3

11 6 -4 2.83162 3.71934 3

4 11 -6 2.36323 3.18902 3

-4 11 6 7.61772 5.21732 2

-4 -11 -6 8.67941 4.46635 1

11 -6 4 8.94704 3.94745 1

-11 6 4 15.8442 6.8917 2

-6 -4 -11 -0.2434 4.2162 2

4 11 6 6.46676 4.04089 3

4 -11 -6 6.75231 4.31957 1

-11 -6 4 5.79152 5.29552 1

-6 -4 11 1.17102 3.22370 3

11 -6 -4 2.73882 3.57147 1

6 4 11 9.78916 4.77919 3

-4 -11 6 8.45099 5.26606 1

11 6 4 4.99370 4.53597 3

6 -4 -11 5.71289 4.53928 2

-11 6 -4 4.16723 6.21411 2

-6 4 -11 7.49961 5.59449 2

-4 11 -6 2.50555 5.44691 2

7 -4 11 101.282 9.762 3

11 7 -4 77.3092 7.7703 3

4 11 -7 98.9014 7.9949 3

-11 7 4 77.0473 10.8847 2

-11 -7 -4 77.6180 9.1189 1

-4 -11 -7 110.824 9.320 1

-7 4 11 84.9719 12.2796 2

-4 11 7 56.0592 8.9044 2

-7 -4 -11 91.7171 9.1146 2

11 -7 -4 75.4396 7.8303 1

-11 -7 4 74.0602 9.6959 1

-7 4 -11 83.1616 10.6569 2

11 7 4 77.3759 8.7631 3

-7 -4 11 74.9998 7.1927 3

-11 7 -4 83.9308 11.2770 2

7 4 11 76.6304 8.8858 3

4 11 7 101.634 8.711 3

4 -11 -7 73.1512 8.3611 1

-4 11 -7 100.328 11.029 2

-4 11 8 14.6181 6.5482 2

-11 -8 -4 13.2557 6.1229 1

8 -4 11 20.7369 6.6475 3

-11 8 4 16.3226 7.6003 2

11 8 -4 13.2589 4.3721 3

-8 4 11 15.4324 8.9651 2

4 11 -8 16.1660 4.4033 3

4 -11 -8 5.53485 4.71489 1

11 -8 -4 15.7004 4.9716 1

-11 8 -4 18.6400 7.5379 2

8 4 11 22.8311 6.5401 3

-4 11 -8 16.8627 7.3955 2

-8 -4 11 14.4720 4.2342 3

11 8 4 12.5901 5.0401 3

-11 -8 4 11.3487 6.6634 1

4 11 8 12.2730 5.1175 3

-8 4 -11 16.8747 7.2308 2

11 9 -4 32.1025 5.6794 3

-11 -9 -4 22.9180 6.9292 1

-4 11 9 33.8768 8.0851 2

4 11 -9 28.1899 5.2531 3

9 -4 11 34.6119 7.9122 3

-11 9 4 25.0659 8.1847 2

-9 4 11 20.4204 9.9360 2

11 -9 -4 24.5018 5.8646 1

-11 9 -4 29.7145 8.2680 2

4 11 9 33.4777 6.5792 3

9 4 11 25.2734 7.0212 3

-9 4 -11 36.3997 8.5937 2

-11 -9 4 23.8908 7.6706 1

11 9 4 36.4273 6.3613 3

-4 11 -9 48.3948 9.2644 2

0 5 11 7.45716 3.68847 3

5 -11 0 7.64303 4.34615 1

11 0 5 0.01824 4.60077 3

-5 11 0 0.09170 4.02254 2

5 11 0 6.91446 3.23123 3

0 5 -11 11.2819 5.1735 2

0 -5 -11 8.12463 3.78858 2

0 -5 11 10.7210 4.0662 3

-11 0 -5 7.98305 4.49837 2

-5 -11 0 8.80474 4.59371 1

11 0 -5 4.82002 3.32647 1

-11 1 5 244.336 17.900 2

-1 -5 -11 254.750 16.509 2

5 -11 1 268.855 16.487 1

5 11 -1 266.883 14.935 3

11 1 -5 272.378 15.347 1

-5 11 1 261.479 17.137 2

-11 -1 -5 258.821 15.509 1

1 5 -11 266.995 18.482 2

-1 5 11 269.079 15.415 3

1 -5 11 292.654 16.194 3

-5 -11 -1 281.484 16.819 1

1 -5 -11 251.937 16.577 2

-11 1 -5 271.886 17.523 2

11 -1 -5 276.405 15.367 1

-5 11 -1 299.464 17.812 2

5 -11 -1 257.400 16.424 1

11 1 5 292.764 17.738 3

-1 5 -11 249.841 18.401 2

1 5 11 250.988 15.532 3

-1 -5 11 273.015 15.775 3

5 11 1 266.636 15.065 3

-2 -5 -11 170.171 11.699 2

-5 11 2 149.548 12.178 2

11 2 -5 153.374 10.369 1

2 -5 11 152.485 11.225 3

5 -11 2 162.040 11.546 1

-11 -2 -5 137.800 10.802 1

5 11 -2 150.795 9.885 3

-5 -11 -2 192.140 12.473 1

2 5 -11 127.450 12.207 2

-11 2 5 122.134 12.444 2

11 -2 -5 164.259 10.709 1

2 -5 -11 160.426 11.975 2

-2 -5 11 177.611 11.394 3

-2 5 -11 151.764 13.881 2

-5 11 -2 182.685 13.386 2

5 11 2 158.210 10.588 3

11 2 5 181.260 13.250 3

-5 -11 2 179.497 12.563 1

2 5 11 162.782 11.378 3

5 -11 -2 132.231 11.430 1

-11 2 -5 139.869 12.573 2

3 5 -11 229.701 17.084 2

5 11 -3 255.328 14.675 3

3 -5 11 258.367 16.025 3

-11 3 5 229.097 18.129 2

-5 -11 -3 311.387 16.790 1

-11 -3 -5 274.162 16.287 1

-3 -5 -11 264.096 16.488 2

5 -11 3 262.000 16.097 1

-5 11 3 258.372 17.207 2

5 -11 -3 230.045 15.877 1

11 -3 -5 246.485 14.857 1

-5 -11 3 240.946 16.237 1

-5 11 -3 311.913 18.378 2

3 -5 -11 273.159 16.837 2

-3 -5 11 265.275 15.411 3

11 3 5 265.427 17.239 3

-11 -3 5 247.998 16.263 1

5 11 3 270.237 15.002 3

-3 5 -11 283.342 19.281 2

-11 3 -5 228.226 17.691 2

3 5 11 254.767 15.884 3

5 -11 4 12.6822 4.5952 1

-5 -11 -4 3.02230 4.13523 1

-4 -5 -11 3.97692 4.16303 2

4 5 -11 5.38944 4.50838 2

11 4 -5 6.78438 3.43453 3

4 -5 11 8.02278 4.55343 3

5 11 -4 5.88803 3.38296 3

-11 4 5 4.70495 6.09787 2

-5 11 4 -0.0136 4.3304 2

-11 -4 -5 3.07209 4.62400 1

4 5 11 4.40321 4.40489 3

11 4 5 7.29222 5.20737 3

4 -5 -11 11.1490 4.3942 2

-5 11 -4 3.29976 5.13521 2

5 -11 -4 3.61750 3.90673 1

-4 -5 11 10.3835 3.8843 3

-4 5 -11 9.39491 5.70300 2

11 -4 -5 2.37456 3.24586 1

-11 4 -5 6.77690 5.72618 2

-5 -11 4 2.41106 4.67389 1

5 11 4 0.79384 3.20032 3

-11 -4 5 7.85522 4.94319 1

-5 -5 -11 289.649 17.820 2

11 5 -5 241.559 15.706 3

5 -5 11 279.336 17.845 3

-5 11 5 281.048 18.845 2

-11 5 5 280.793 20.226 2

-11 -5 -5 268.776 17.566 1

-5 -11 -5 348.116 17.751 1

5 11 -5 282.838 15.870 3

11 -5 -5 255.981 15.670 1

5 -11 -5 253.960 16.560 1

-5 11 -5 321.460 19.885 2

-5 -11 5 244.431 17.031 1

-5 5 -11 296.731 20.375 2

11 5 5 274.634 17.643 3

-11 5 -5 264.756 19.641 2

5 11 5 272.552 15.988 3

-11 -5 5 251.643 17.549 1

5 -5 -11 258.181 17.362 2

5 5 11 273.833 17.050 3

-5 -5 11 268.212 15.857 3

5 11 -6 110.373 8.736 3

-5 -11 -6 132.533 10.429 1

-5 11 6 115.933 11.367 2

-6 -5 -11 121.993 10.464 2

-11 6 5 113.488 12.714 2

6 -5 11 110.950 10.649 3

11 6 -5 110.410 9.220 3

-11 -6 -5 114.768 10.645 1

-5 11 -6 134.059 12.393 2

-6 -5 11 101.606 9.093 3

-11 -6 5 117.957 11.376 1

-11 6 -5 106.931 12.308 2

6 -5 -11 112.596 10.625 2

-6 5 -11 112.097 12.315 2

-5 -11 6 110.495 10.558 1

6 5 11 120.030 10.674 3

11 -6 -5 118.450 9.529 1

11 6 5 105.507 10.511 3

5 -11 -6 112.608 10.093 1

5 11 6 117.835 10.002 3

-11 -7 -5 100.826 10.241 1

5 11 -7 116.704 8.923 3

-7 5 11 81.0270 12.8778 2

11 7 -5 107.180 9.258 3

-5 11 7 110.899 11.586 2

-11 7 5 124.385 13.140 2

-5 -11 -7 118.284 10.266 1

7 -5 11 124.722 11.201 3

-11 7 -5 83.9285 11.7823 2

5 11 7 105.252 9.429 3

5 -11 -7 100.248 9.581 1

11 -7 -5 97.0159 9.0381 1

7 5 11 105.272 10.270 3

-7 5 -11 102.141 12.098 2

-5 11 -7 117.014 12.226 2

11 7 5 116.377 10.451 3

-5 -11 7 105.096 10.373 1

-11 -7 5 91.2370 10.6717 1

-5 11 8 3.32906 6.27025 2

5 11 -8 6.49718 3.79042 3

8 5 -11 7.82644 3.80628 3

-8 5 11 6.20347 8.57436 2

-11 8 5 7.30714 7.52101 2

8 -5 11 14.7365 6.5967 3

-11 -8 -5 9.53113 6.11612 1

11 8 -5 7.13648 4.08102 3

11 -8 -5 6.94026 4.54418 1

-11 -8 5 8.36341 6.64857 1

5 -11 -8 11.3096 5.3148 1

-11 8 -5 15.0044 7.5092 2

-5 11 -8 5.61230 6.57334 2

-8 5 -11 8.00386 6.87050 2

5 11 8 5.88649 4.67515 3

11 8 5 7.90003 5.00103 3

8 5 11 6.29091 5.47754 3

-6 -11 0 4.44790 4.79319 1

0 -6 -11 11.8758 4.2022 2

11 0 6 10.8837 5.2950 3

11 0 -6 6.34667 3.55203 1

0 6 -11 5.18879 5.23029 2

-6 11 0 9.87716 5.05755 2

-11 0 -6 2.13686 4.44786 2

0 6 11 6.82850 4.23094 3

6 11 0 6.45777 3.27131 3

0 -6 11 2.42121 3.68465 3

6 -11 0 9.95446 4.48562 1

-6 11 1 167.843 13.183 2

6 -11 1 155.320 11.765 1

11 -1 6 195.056 14.010 3

-11 1 6 145.435 13.568 2

6 11 -1 166.331 10.718 3

-6 -11 -1 176.150 12.643 1

-1 6 11 152.471 11.306 3

-1 -6 -11 179.214 12.062 2

11 1 -6 166.739 10.936 1

1 -6 11 162.375 11.839 3

1 6 -11 151.157 13.545 2

6 -11 -1 167.561 12.146 1

1 6 11 171.106 12.087 3

-1 6 -11 188.071 14.979 2

1 -6 -11 161.504 12.075 2

6 11 1 163.390 10.958 3

11 -1 -6 161.713 10.930 1

11 1 6 171.469 13.294 3

-6 11 -1 167.435 13.270 2

-6 -11 1 180.110 12.918 1

-1 -6 11 163.721 11.806 3

-11 1 -6 170.790 13.187 2

6 -11 2 92.2195 8.5600 1

2 -6 11 99.3005 8.9203 3

-2 -6 -11 107.880 8.925 2

11 2 -6 91.4051 7.8592 1

-6 -11 -2 96.4802 9.2353 1

2 6 -11 85.1709 9.8210 2

-6 11 2 81.4781 9.0400 2

6 11 -2 99.8078 7.6861 3

-11 2 6 77.6690 10.2251 2

2 -6 -11 97.0541 8.8703 2

-11 2 -6 96.9341 10.0642 2

-6 11 -2 70.8024 9.4541 2

-2 6 -11 97.1973 10.9122 2

6 11 2 98.4225 8.0337 3

11 -2 -6 89.3378 7.7285 1

-2 -6 11 93.8677 8.5529 3

6 -11 -2 94.1129 8.9399 1

2 6 11 88.0932 8.7135 3

11 2 6 107.614 10.253 3

-6 -11 2 93.6127 9.2839 1

-6 -11 -3 210.342 14.000 1

-11 3 6 185.097 15.690 2

6 -11 3 189.830 13.050 1

-6 11 3 206.576 14.847 2

-3 -6 -11 210.598 13.476 2

3 -6 11 171.902 13.099 3

6 11 -3 179.038 11.789 3

-6 11 -3 226.182 15.600 2

-11 -3 6 192.207 13.956 1

11 3 6 191.307 14.549 3

6 11 3 204.354 12.611 3

6 -11 -3 170.822 13.193 1

-6 -11 3 202.067 14.309 1

3 6 11 218.733 13.907 3

-3 6 -11 205.084 16.453 2

-3 -6 11 195.943 13.032 3

11 -3 -6 193.243 12.513 1

-11 3 -6 186.708 15.283 2

3 -6 -11 189.763 13.755 2

4 -6 11 -3.0941 4.0693 3

6 11 -4 3.27511 3.11418 3

-4 -6 -11 6.15718 4.02272 2

11 4 -6 6.81166 3.27921 3

-6 11 4 4.30884 5.14690 2

6 -11 4 7.43776 4.58478 1

-6 -11 -4 2.35630 4.49887 1

-11 4 6 9.60783 6.57952 2

11 4 6 10.3198 5.4809 3

6 11 4 5.15526 3.66835 3

-6 -11 4 0.33791 4.82123 1

6 -11 -4 6.31897 4.39417 1

-6 11 -4 6.81964 5.70279 2

4 -6 -11 3.31950 3.82747 2

-4 6 -11 15.3576 6.6524 2

11 -4 -6 9.96622 3.87424 1

-4 -6 11 10.8661 4.3593 3

4 6 11 4.08306 4.56018 3

-11 4 -6 3.48684 5.46126 2

-11 -4 6 7.61448 5.35734 1

5 -6 11 115.239 10.675 3

6 11 -5 115.311 8.889 3

-6 11 5 102.991 10.868 2

-11 5 6 137.448 13.569 2

11 5 -6 115.839 8.887 3

-6 -11 -5 111.211 9.953 1

5 -6 -11 114.398 10.564 2

5 6 11 127.130 10.840 3

-5 -6 11 124.591 9.874 3

-11 5 -6 146.542 13.510 2

11 -5 -6 123.027 9.590 1

11 5 6 120.923 11.220 3

-11 -5 6 126.457 11.500 1

-6 -11 5 101.589 10.381 1

-5 6 -11 114.911 12.729 2

-6 11 -5 120.471 12.294 2

6 -11 -5 104.276 9.735 1

6 11 5 110.578 9.549 3

6 11 -6 118.724 8.983 3

-6 6 11 125.425 14.128 2

-6 11 6 109.825 11.385 2

-6 -11 -6 135.100 10.492 1

-11 6 6 114.663 13.174 2

11 6 -6 102.962 9.002 3

6 6 -11 114.546 8.989 3

6 -6 11 118.998 11.193 3

-6 -11 6 98.0817 10.2940 1

11 6 6 131.033 11.418 3

11 -6 -6 116.245 9.689 1

-6 11 -6 122.218 12.371 2

-11 -6 6 108.540 11.419 1

6 11 6 113.773 10.066 3

6 -11 -6 113.311 10.337 1

6 6 11 128.621 11.091 3

-6 6 -11 118.792 13.103 2

-11 6 -6 117.830 13.027 2

6 11 -7 166.921 11.517 3

-11 7 6 201.647 17.132 2

-6 11 7 172.023 15.031 2

11 7 -6 153.396 11.613 3

7 -6 11 159.228 13.680 3

-7 6 11 183.997 17.522 2

7 6 -11 188.381 11.793 3

-7 6 -11 170.264 16.052 2

11 7 6 161.799 13.299 3

-6 -11 7 152.752 13.451 1

6 11 7 180.479 12.817 3

11 -7 -6 160.148 12.084 1

-6 11 -7 186.867 15.913 2

-11 -7 6 174.192 14.430 1

6 -11 -7 176.791 13.311 1

7 6 11 172.038 13.435 3

-11 7 -6 153.709 15.439 2

11 8 -6 0.11003 3.69193 3

8 6 -11 9.71652 4.22238 3

-11 8 6 9.37581 8.16633 2

-8 6 11 5.83730 9.18430 2

6 11 -8 4.19188 3.66819 3

-6 11 8 2.84138 6.11377 2

6 11 8 9.32020 4.86406 3

-8 6 -11 9.41743 6.97719 2

-11 -8 6 8.93782 6.61152 1

11 -8 -6 3.23116 4.68654 1

6 -11 -8 3.34176 4.97298 1

-6 11 -8 8.96648 7.30631 2

11 8 6 -2.4221 4.7061 3

8 6 11 5.65238 5.12672 3

-11 8 -6 5.44594 6.47103 2

11 0 7 129.607 11.456 3

-11 0 -7 105.218 10.318 2

11 0 -7 103.446 8.625 1

7 -11 0 122.978 10.154 1

-7 11 0 126.055 11.642 2

0 7 -11 85.3382 10.8684 2

0 -7 -11 140.941 10.137 2

0 -7 11 117.193 10.275 3

-7 -11 0 121.527 10.925 1

7 11 0 116.422 8.857 3

11 -1 7 139.789 12.033 3

7 -11 1 129.056 10.500 1

7 11 -1 129.198 9.328 3

-1 -7 -11 140.874 10.510 2

-11 1 7 129.734 12.759 2

-7 -11 -1 132.601 11.430 1

-7 11 1 102.678 10.998 2

11 1 -7 127.322 9.383 1

1 7 -11 100.479 11.237 2

1 -7 11 139.592 10.745 3

-7 -11 1 140.921 11.433 1

-1 7 -11 105.288 11.939 2

7 -11 -1 112.348 10.017 1

7 11 1 138.068 9.536 3

-11 1 -7 98.3649 10.4824 2

11 1 7 120.065 11.323 3

1 7 11 110.913 9.915 3

-1 -7 11 130.674 10.478 3

1 -7 -11 137.243 10.442 2

11 -1 -7 110.089 8.989 1

-7 11 -1 130.129 11.775 2

7 -11 2 253.265 15.544 1

11 2 -7 238.423 14.395 1

7 11 -2 241.592 14.099 3

2 -7 11 236.803 15.565 3

-7 11 2 224.774 16.735 2

-2 -7 -11 246.816 15.382 2

-11 2 7 224.517 18.292 2

2 -7 -11 257.058 16.144 2

-7 -11 2 252.748 16.605 1

7 -11 -2 231.651 15.642 1

2 7 11 236.759 15.636 3

-2 7 -11 239.629 18.726 2

11 -2 -7 232.936 14.613 1

-7 11 -2 282.198 18.255 2

-2 -7 11 256.398 15.912 3

7 11 2 251.856 14.904 3

-11 2 -7 234.881 17.563 2

11 2 7 262.603 17.141 3

3 -7 11 59.6999 7.7045 3

-7 -11 -3 50.6606 7.5741 1

-3 -7 -11 44.6441 6.3842 2

7 -11 3 72.3646 7.4874 1

7 11 -3 59.6063 6.2742 3

-11 3 7 72.5946 10.6678 2

-7 11 3 50.5504 8.0592 2

-11 3 -7 52.4348 8.2832 2

3 -7 -11 50.6794 6.7691 2

7 -11 -3 42.4748 6.7559 1

7 11 3 52.6232 6.2812 3

3 7 11 59.8467 7.4045 3

-7 -11 3 59.0633 7.8938 1

-3 -7 11 48.8142 6.7747 3

-7 11 -3 49.0222 7.9206 2

11 -3 -7 55.9695 6.2212 1

-11 -3 7 61.3812 7.8187 1

-3 7 -11 54.4099 9.2317 2

11 3 7 44.3125 7.6937 3

-7 -11 -4 95.0633 9.3519 1

7 11 -4 81.1276 7.3725 3

-11 4 7 96.8334 11.8556 2

-7 11 4 77.5639 9.8330 2

4 -7 11 75.3521 8.8980 3

7 -11 4 82.9179 8.2527 1

7 -11 -4 77.7390 8.4990 1

4 7 11 92.7041 9.1411 3

-4 7 -11 70.5112 10.7931 2

-4 -7 11 69.4310 8.0027 3

11 4 7 84.9053 9.4055 3

7 11 4 85.7617 7.9926 3

11 -4 -7 71.5846 7.5216 1

-11 4 -7 90.8412 10.5881 2

-7 11 -4 94.3743 10.6949 2

-7 -11 4 89.8000 9.5116 1

4 -7 -11 103.272 9.331 2

-11 -4 7 64.2347 8.8390 1

-5 7 11 118.080 13.148 2

5 7 -11 110.290 8.993 3

11 5 -7 112.864 8.696 3

-11 5 7 115.206 13.248 2

7 11 -5 103.790 8.651 3

-7 11 5 94.5999 11.0656 2

5 -7 11 111.400 10.763 3

-7 -11 -5 116.563 10.152 1

-7 11 -5 125.201 12.274 2

-5 7 -11 112.447 13.003 2

5 7 11 104.522 10.152 3

7 11 5 125.073 9.891 3

-7 -11 5 99.3542 10.2518 1

-11 -5 7 100.848 10.860 1

-11 5 -7 108.124 12.214 2

7 -11 -5 98.6262 9.8600 1

11 5 7 104.187 10.718 3

11 -5 -7 102.042 9.074 1

11 6 -7 176.327 11.767 3

-6 7 11 177.934 16.949 2

6 7 -11 175.815 11.912 3

6 -7 11 164.370 14.244 3

-7 -11 -6 227.157 13.862 1

-7 11 6 125.146 13.858 2

-11 6 7 181.916 16.876 2

-7 -11 6 155.270 13.515 1

6 7 11 173.484 13.273 3

7 11 6 183.190 12.679 3

-7 11 -6 195.657 15.799 2

-6 7 -11 178.845 16.602 2

-11 6 -7 189.248 16.603 2

7 -11 -6 158.701 12.984 1

-11 -6 7 157.829 14.100 1

11 6 7 181.487 14.044 3

11 -6 -7 159.055 12.145 1

7 7 -11 28.7060 5.2740 3

7 11 -7 23.5750 4.8672 3

11 7 -7 20.0022 4.9128 3

-11 7 7 28.4844 9.5531 2

-7 7 11 20.5941 8.7111 2

-7 11 7 27.1072 7.5053 2

7 11 7 22.1016 5.5527 3

11 7 7 10.4006 5.7363 3

11 -7 -7 17.4774 5.5159 1

7 -11 -7 19.0314 5.8060 1

7 7 11 24.1118 6.5859 3

-7 11 -7 30.0228 8.0154 2

-11 7 -7 29.0963 8.6005 2

-11 -7 7 29.0854 7.8748 1

-7 -11 7 25.8467 7.0991 1

-7 7 -11 19.8750 8.3153 2

-8 -11 0 13.0900 5.9565 1

11 0 8 6.53166 6.00701 3

-11 0 -8 2.63469 4.72255 2

0 -8 -11 16.2352 4.7453 2

0 -8 11 15.0254 5.4657 3

8 11 0 7.69851 3.82548 3

0 8 -11 4.28025 5.73891 2

-8 11 0 13.0522 5.9913 2

11 0 -8 7.60545 3.82345 1

8 -11 0 11.7749 5.0104 1

-1 -8 -11 13.4484 4.4697 2

11 1 -8 5.80233 3.75258 1

-8 11 1 17.4526 6.0799 2

8 -11 1 4.00460 4.39996 1

11 -1 8 -0.2165 5.7032 3

1 -8 11 10.6876 5.2240 3

8 11 -1 9.09195 3.78528 3

-8 -11 -1 5.09402 5.30229 1

-11 1 8 14.0174 7.7095 2

8 -11 -1 7.02593 4.89439 1

-1 -8 11 5.54883 4.67330 3

11 1 8 17.1589 6.2996 3

-11 1 -8 6.87661 5.41456 2

-8 -11 1 7.31339 5.60741 1

-8 11 -1 6.32836 5.60282 2

1 -8 -11 10.3329 4.3293 2

11 -1 -8 4.05932 3.64980 1

8 11 1 8.29205 3.99551 3

-1 8 -11 9.50635 6.39749 2

-8 11 2 1.99414 5.13014 2

-8 -11 -2 9.24838 5.61300 1

2 -8 11 3.63391 4.56148 3

-11 2 8 2.45097 6.99650 2

-2 -8 -11 2.70856 3.84141 2

8 11 -2 0.74751 3.08916 3

8 -11 2 1.55345 4.41833 1

2 8 11 5.88059 4.86503 3

8 -11 -2 -0.4154 4.2875 1

-2 -8 11 0.59137 4.30120 3

-11 -2 8 -3.9334 4.7597 1

8 11 2 4.94246 3.73151 3

11 2 8 6.91545 5.52014 3

11 -2 -8 6.20258 3.82328 1

-8 11 -2 2.30187 5.56798 2

-2 8 -11 7.97512 6.18692 2

-11 2 -8 -2.8023 5.0970 2

-8 -11 2 11.7711 5.7668 1

2 -8 -11 2.81521 3.96183 2

8 -11 3 42.3260 6.6156 1

-11 3 8 32.8549 9.3142 2

3 -8 11 21.0149 6.2077 3

-8 11 3 40.1975 7.6863 2

-8 -11 -3 38.8777 7.0398 1

8 11 -3 36.6343 5.2420 3

11 3 8 42.9444 7.1519 3

-8 -11 3 25.7400 6.8375 1

-3 -8 11 31.7856 6.2198 3

11 -3 -8 32.4738 5.4890 1

-8 11 -3 28.9900 7.1760 2

8 -11 -3 27.1046 6.1239 1

-3 8 -11 23.4227 7.7619 2

-11 -3 8 23.4378 6.3355 1

3 8 11 40.6256 6.7102 3

8 11 3 37.3269 5.7598 3

-11 3 -8 33.4346 7.7994 2

4 -8 11 9.24137 5.71873 3

-4 8 11 19.5670 7.5480 2

-8 -11 -4 14.5176 6.0920 1

-11 4 8 13.7979 8.4325 2

8 11 -4 15.9157 4.3079 3

-8 11 4 15.2078 6.4367 2

-8 11 -4 11.5087 6.4649 2

4 8 11 11.4627 5.2910 3

-11 -4 8 8.94461 6.21506 1

11 4 8 12.2453 5.6479 3

8 11 4 17.5686 4.9028 3

-4 8 -11 23.2444 7.7445 2

-8 -11 4 12.2123 5.9498 1

11 -4 -8 14.0339 4.6381 1

-11 4 -8 21.6379 7.5845 2

8 -11 -4 18.7767 5.5877 1

-4 -8 11 14.3507 5.2021 3

-8 -11 -5 7.27450 5.60666 1

8 11 -5 10.3350 3.9958 3

-8 11 5 3.60347 6.21437 2

5 8 -11 13.3656 4.4411 3

11 5 -8 6.84720 3.88011 3

5 -8 11 -3.8573 5.3737 3

-5 8 11 7.07102 6.72080 2

-11 5 8 13.0296 8.6600 2

5 8 11 10.1510 5.3612 3

-8 11 -5 4.97747 6.55004 2

-5 8 -11 11.8824 7.1999 2

8 -11 -5 9.05882 5.00289 1

-8 -11 5 4.05981 5.92445 1

-11 -5 8 11.9645 6.2896 1

11 5 8 3.93800 5.40815 3

-11 5 -8 16.7497 7.4367 2

8 11 5 3.30573 4.09028 3

11 -5 -8 3.70272 4.37129 1

8 11 -6 3.40712 3.67496 3

11 6 -8 -0.1490 3.6294 3

-8 -11 -6 1.29538 5.04264 1

-6 8 11 3.15123 7.09957 2

-11 6 8 6.73984 8.38357 2

6 8 -11 2.98397 4.03653 3

-8 11 6 4.09936 6.41181 2

8 -11 -6 7.15409 5.11049 1

-6 8 -11 0.16797 6.78085 2

-8 -11 6 1.20568 5.81997 1

-8 11 -6 6.51709 6.41605 2

-11 6 -8 -8.7984 6.4496 2

6 8 11 7.93564 5.18003 3

8 11 6 -2.3785 4.0163 3

11 6 8 11.0729 6.0717 3

-11 -6 8 -1.6264 6.4901 1

11 -6 -8 3.18917 4.62585 1

-11 0 -9 37.1612 7.2726 2

9 -11 0 26.2418 6.1219 1

9 11 0 43.9755 5.8873 3

-9 11 0 47.6696 8.3744 2

11 0 -9 23.7048 5.1288 1

-9 -11 0 24.3974 7.0514 1

11 0 9 33.7478 7.7868 3

0 -9 -11 38.5925 6.2377 2

0 -9 11 33.3177 6.9873 3

-9 -11 -1 241.020 16.347 1

1 -9 11 202.320 15.079 3

9 -11 1 217.480 14.978 1

9 11 -1 213.077 13.723 3

-1 -9 -11 237.125 14.877 2

11 1 -9 202.220 13.593 1

11 -1 9 263.270 17.083 3

-9 11 1 195.013 16.405 2

-1 -9 11 188.507 14.616 3

11 1 9 237.346 16.565 3

9 11 1 205.905 13.476 3

-9 -11 1 214.139 15.582 1

-11 1 -9 216.385 16.123 2

-9 11 -1 200.676 16.343 2

11 -1 -9 193.957 13.205 1

9 -11 -1 215.816 14.877 1

1 -9 -11 225.192 14.738 2

9 11 -2 81.9381 7.3801 3

2 -9 11 65.4800 8.6595 3

9 -11 2 67.1468 8.3675 1

-2 9 11 62.0740 9.8006 2

-9 11 2 70.6461 9.9012 2

-9 -11 -2 81.2188 9.5116 1

-11 2 9 70.7609 11.3259 2

11 2 9 79.8044 9.7499 3

-2 9 -11 83.4504 11.1523 2

9 11 2 70.9979 7.5814 3

-9 -11 2 86.0397 9.6413 1

11 -2 -9 75.6026 7.4905 1

-11 -2 9 49.3661 8.4238 1

9 -11 -2 72.6567 8.6212 1

-2 -9 11 66.9671 8.5166 3

-9 11 -2 70.8196 9.8550 2

-11 2 -9 72.5099 9.9281 2

9 11 -3 134.377 10.345 3

-9 11 3 161.423 14.187 2

3 -9 11 136.239 12.280 3

9 -11 3 145.153 11.816 1

-3 9 11 142.244 14.135 2

-11 3 9 148.614 15.460 2

-9 -11 -3 148.469 12.748 1

-11 3 -9 150.868 14.504 2

-3 -9 11 128.867 12.038 3

-9 11 -3 155.920 14.408 2

3 9 11 162.981 12.472 3

11 3 9 163.892 13.610 3

11 -3 -9 148.998 11.225 1

-11 -3 9 150.414 13.173 1

-9 -11 3 173.858 13.839 1

9 -11 -3 156.890 12.446 1

9 11 3 179.796 11.854 3

-3 9 -11 149.780 14.869 2

-9 11 4 42.7531 8.8373 2

4 9 -11 29.9755 5.3300 3

-11 4 9 26.3644 9.9757 2

-4 9 11 20.1063 8.1993 2

-9 -11 -4 34.9921 7.4140 1

9 11 -4 33.9464 5.2947 3

4 -9 11 19.8347 6.5439 3

-9 -11 4 39.1502 7.9744 1

4 9 11 32.6671 6.5017 3

11 4 9 35.8058 7.2706 3

9 -11 -4 24.9794 6.0525 1

-11 4 -9 23.6756 8.1154 2

11 -4 -9 33.4812 5.9267 1

-4 9 -11 25.7134 8.7252 2

9 11 4 31.4960 5.8160 3

-9 11 -4 28.7386 7.9981 2

0 -10 11 7.15900 5.70508 3

10 11 0 2.85569 3.86009 3

10 -11 0 6.54910 5.51270 1

-11 0 -10 10.8146 5.9210 2

-10 -11 0 12.6123 6.6538 1

11 0 10 9.08644 6.92666 3

-10 11 0 8.25940 6.67389 2

11 -1 10 69.3429 10.3519 3

10 11 -1 81.8000 8.0594 3

-10 -11 -1 96.8221 10.7814 1

10 -11 1 98.0997 9.6339 1

-1 10 11 80.8597 10.8781 2

-10 11 1 70.1998 10.6322 2

1 -10 11 83.1181 9.5672 3

-1 -10 11 68.3989 9.4595 3

11 1 10 114.302 11.562 3

10 11 1 85.4178 8.2617 3

10 -11 -1 96.6936 9.7582 1

-11 1 -10 68.3279 9.7588 2

-10 11 -1 84.4574 11.1638 2

12 0 0 842.226 38.209 1

0 0 -12 741.519 41.935 2

0 -12 0 777.661 39.573 1

0 0 12 810.344 38.106 3

0 -12 -1 214.963 14.343 1

1 0 -12 203.610 15.129 2

0 -12 1 236.809 14.875 1

12 -1 0 236.999 13.193 1

-1 0 12 221.696 13.314 3

1 0 12 198.716 13.373 3

12 1 0 225.960 13.156 1

-12 1 0 237.669 15.377 2

-1 0 -12 198.209 15.231 2

0 -12 2 -0.7053 4.0714 1

2 0 12 0.75644 3.41701 3

2 0 -12 -2.4724 3.5060 2

-2 0 -12 -1.0605 3.8001 2

-12 2 0 1.34929 4.78872 2

12 -2 0 -4.1055 2.9132 1

12 2 0 -0.9705 3.1409 1

-2 0 12 0.07722 2.83061 3

0 12 -2 -2.6292 2.5668 3

0 -12 -2 5.03264 3.80968 1

12 3 0 128.969 10.587 3

0 -12 -3 146.540 11.228 1

-12 -3 0 141.168 11.409 1

12 -3 0 150.723 10.218 1

-3 0 12 155.337 10.137 3

3 0 -12 147.733 12.079 2

0 -12 3 142.683 11.561 1

-3 0 -12 146.475 12.537 2

3 0 12 143.376 10.838 3

0 12 -3 176.291 10.142 3

-12 3 0 157.267 12.849 2

0 12 -4 904.118 38.556 3

12 -4 0 858.684 39.551 1

12 4 0 827.835 39.884 3

0 -12 -4 853.939 40.804 1

-4 0 -12 821.540 43.714 2

-4 0 12 803.211 38.511 3

-12 4 0 684.284 43.110 2

-12 -4 0 828.368 41.181 1

0 -12 4 787.375 40.693 1

4 0 12 792.222 39.793 3

-12 -5 0 286.792 18.175 1

-12 5 0 219.584 18.511 2

12 -5 0 286.439 16.213 1

5 0 -12 288.835 18.501 2

-5 0 -12 295.377 18.952 2

0 -12 5 260.191 17.037 1

0 12 -5 274.578 15.213 3

0 -12 -5 283.081 16.896 1

-5 0 12 274.994 15.427 3

5 0 12 274.635 16.920 3

12 5 0 266.931 16.553 3

-12 6 0 -0.2563 5.4999 2

0 -12 -6 6.97809 4.44379 1

6 0 -12 7.97042 5.12120 2

0 12 -6 6.01409 3.43629 3

-12 -6 0 7.47007 5.59383 1

12 -6 0 2.10493 3.87299 1

6 0 12 -1.4173 4.5224 3

-6 0 -12 15.3256 5.6175 2

12 6 0 4.37620 4.12172 3

-7 0 -12 57.8518 8.2559 2

-12 7 0 50.8934 9.0329 2

0 -12 -7 65.5417 7.3673 1

0 12 -7 54.5048 6.2496 3

7 0 12 55.4351 7.9181 3

12 -7 0 60.4824 7.1248 1

7 0 -12 50.4321 7.9328 2

12 7 0 56.0349 7.0069 3

-12 -7 0 45.3399 8.3691 1

12 8 0 386.764 22.773 3

8 0 12 426.744 24.516 3

0 -12 -8 439.936 23.627 1

0 12 -8 428.751 22.020 3

-12 8 0 411.054 27.034 2

12 -8 0 412.555 23.227 1

8 0 -12 412.062 25.332 2

-12 -8 0 415.990 25.040 1

12 0 -1 238.428 13.438 1

-1 -12 0 203.416 14.621 1

0 -1 -12 221.526 15.320 2

0 1 -12 194.650 15.441 2

1 -12 0 238.819 14.950 1

0 -1 12 203.925 13.481 3

12 0 1 235.129 13.252 1

1 12 0 250.961 12.957 3

0 1 12 213.907 13.546 3

1 -12 1 2.15708 4.16836 1

-1 -12 -1 4.29817 4.12672 1

-1 -1 -12 1.42592 3.99550 2

12 1 -1 8.96760 3.49671 1

-12 1 1 -2.1417 4.5319 2

12 -1 1 7.79255 3.34735 1

1 1 -12 7.24512 4.39142 2

1 12 -1 4.98018 3.00160 3

-1 1 12 6.80039 3.48340 3

1 -1 12 2.74774 3.67206 3

-1 -1 12 1.51484 3.30660 3

1 12 1 1.27560 2.92684 3

1 -12 -1 5.25062 4.25921 1

12 1 1 8.54422 3.45493 1

1 -1 -12 7.10704 4.26479 2

-1 1 -12 6.82569 4.65816 2

12 -1 -1 0.66174 3.06570 1

-1 -12 1 -2.4641 4.0026 1

-12 1 -1 7.39554 4.67747 2

1 1 12 4.95846 3.55065 3

2 -1 12 67.2737 7.0317 3

-2 1 12 57.4924 6.1547 3

-1 -12 -2 68.3252 7.2632 1

12 -2 1 60.4794 6.1081 1

2 1 -12 61.5080 7.8644 2

12 2 -1 61.2201 6.3201 1

-2 -1 -12 52.5965 7.5105 2

1 12 -2 70.8685 6.1196 3

-12 2 1 60.6145 8.2038 2

1 -12 2 68.2079 7.6516 1

-12 2 -1 56.2523 8.0683 2

-2 1 -12 66.3858 8.2870 2

2 -1 -12 72.9457 8.0548 2

2 1 12 71.0475 6.9423 3

1 -12 -2 68.3017 7.5585 1

-1 -12 2 53.9127 7.1946 1

12 -2 -1 71.8693 6.4644 1

-2 -1 12 77.3137 6.7184 3

12 2 1 38.5993 6.2081 3

12 -3 1 3.51652 3.25065 1

-3 1 12 6.61175 3.40011 3

-12 3 1 -3.8158 4.5607 2

-1 -12 -3 3.46264 3.92939 1

1 12 -3 -1.6206 2.7953 3

-3 -1 -12 3.30723 3.96269 2

3 -1 12 2.83067 3.90083 3

1 -12 3 2.39966 4.44362 1

12 3 -1 5.42729 3.68137 3

3 1 -12 3.32530 4.33425 2

-12 -3 -1 11.4693 4.8137 1

12 -3 -1 3.88183 3.38935 1

3 1 12 7.54010 4.21584 3

-1 -12 3 4.92627 4.60987 1

3 -1 -12 6.64252 4.11110 2

-12 -3 1 2.01491 4.11810 1

-12 3 -1 6.06574 5.39181 2

1 -12 -3 5.15891 3.99946 1

-3 1 -12 3.21825 4.30789 2

-3 -1 12 2.32649 3.10863 3

12 3 1 -4.8693 3.5224 3

-4 -1 -12 257.209 16.582 2

4 1 -12 224.007 16.011 2

12 4 -1 197.660 13.834 3

-12 -4 -1 221.535 15.288 1

-12 4 1 206.812 16.634 2

1 12 -4 264.152 13.696 3

4 -1 12 221.690 14.695 3

1 -12 4 220.330 15.034 1

12 -4 1 247.655 13.972 1

-1 -12 -4 221.070 14.850 1

-4 1 12 230.545 13.553 3

1 -12 -4 209.019 14.110 1

12 -4 -1 220.266 13.337 1

4 1 12 253.501 14.552 3

12 4 1 223.819 14.435 3

-12 4 -1 197.874 16.072 2

-4 1 -12 205.583 15.868 2

-1 -12 4 181.154 14.560 1

-12 -4 1 218.969 14.832 1

4 -1 -12 216.965 15.122 2

-4 -1 12 208.606 12.935 3

1 12 -5 30.4959 4.6255 3

12 -5 1 20.0807 4.6474 1

-1 -12 -5 17.8991 5.1936 1

12 5 -1 24.0232 5.0884 3

5 1 -12 28.8049 6.0479 2

5 -1 12 22.3814 5.6914 3

1 -12 5 29.7787 6.2516 1

-12 -5 -1 15.7436 5.9946 1

-5 -1 -12 38.7277 6.4987 2

-12 5 1 15.6313 6.5171 2

-12 -5 1 26.6042 6.5839 1

5 -1 -12 24.5694 5.8121 2

5 1 12 26.5480 5.6730 3

-5 1 -12 25.4616 6.4030 2

-5 -1 12 16.4638 4.1910 3

12 -5 -1 21.3472 4.7530 1

1 -12 -5 29.1616 5.5290 1

12 5 1 23.1095 5.4243 3

-1 -12 5 24.4522 6.1588 1

-12 5 -1 24.8372 7.2342 2

-6 -1 -12 59.9808 8.2037 2

6 -1 12 55.1609 7.6226 3

-1 -12 -6 62.4464 7.2080 1

6 1 -12 63.6735 8.1689 2

12 6 -1 63.7758 7.2522 3

12 -6 1 69.4699 7.1485 1

1 12 -6 58.1441 6.0409 3

-12 6 1 42.8561 8.1168 2

-12 -6 -1 49.9538 8.2757 1

12 6 1 47.7559 6.8201 3

6 1 12 53.7617 7.5297 3

-6 1 -12 64.7041 8.6976 2

12 -6 -1 57.6862 6.6645 1

-6 -1 12 55.0811 5.8640 3

1 -12 -6 54.1896 6.9164 1

-12 6 -1 30.9657 7.9184 2

-1 -12 6 55.4474 7.3274 1

6 -1 -12 62.8494 8.1215 2

-12 -6 1 55.6205 8.3981 1

-7 -1 -12 12.1049 5.5778 2

1 12 -7 5.70671 3.67184 3

-12 -7 -1 2.21320 5.66504 1

-12 7 1 11.4944 6.6654 2

7 1 -12 4.29549 4.77003 2

12 -7 1 9.60622 4.56284 1

-1 -12 -7 7.15336 4.55155 1

7 -1 12 3.60500 5.19735 3

12 7 -1 6.16151 4.00407 3

12 -7 -1 8.55519 4.42522 1

-12 7 -1 11.0928 6.7443 2

7 1 12 13.8284 5.5348 3

-12 -7 1 6.96068 5.89767 1

-1 12 -7 5.21783 3.80717 3

1 -12 -7 10.6393 4.8373 1

-7 1 -12 6.40531 5.51108 2

7 -1 -12 11.6102 5.3785 2

12 7 1 5.03410 4.59683 3

-12 8 1 153.241 15.147 2

1 12 -8 165.990 11.028 3

-12 -8 -1 153.647 13.834 1

1 -12 8 131.010 11.814 3

12 8 -1 143.424 11.498 3

8 -1 12 140.519 13.014 3

-8 -1 -12 160.229 13.564 2

12 -8 1 182.482 12.244 1

-12 8 -1 172.096 15.619 2

1 -12 -8 157.810 12.124 1

8 1 12 149.823 13.395 3

12 -8 -1 175.927 12.243 1

-8 1 -12 142.730 13.769 2

8 -1 -12 154.108 13.215 2

-12 -8 1 144.772 13.260 1

12 8 1 135.374 11.635 3

2 12 0 0.16973 2.71676 3

12 0 -2 -3.4672 2.6869 1

2 -12 0 -1.5189 3.5973 1

0 2 12 -0.8403 3.0703 3

0 -2 -12 2.60504 3.57416 2

0 -2 12 3.23640 3.20116 3

0 2 -12 -1.3875 3.9476 2

-2 -12 0 -2.8143 3.7022 1

12 0 2 0.18116 3.03283 1

2 12 -1 67.9795 5.9538 3

12 1 -2 64.9329 6.2744 1

-1 2 12 61.7213 6.3213 3

1 2 -12 57.7884 7.9421 2

1 -2 12 58.2313 6.4944 3

12 -1 2 54.1540 5.8437 1

2 -12 1 62.6699 7.5439 1

-12 1 2 62.7262 7.8640 2

-2 -12 -1 57.5336 6.9917 1

-1 -2 -12 56.7305 7.2231 2

-2 -12 1 60.7425 7.1745 1

-12 1 -2 42.0260 7.1161 2

1 -2 -12 58.9236 7.2900 2

2 12 1 85.5109 6.5759 3

-1 -2 12 67.2198 6.5946 3

12 1 2 69.4461 6.3236 1

2 -12 -1 50.2633 6.8819 1

-1 2 -12 68.5159 8.4596 2

1 2 12 67.4627 6.7639 3

12 -1 -2 58.8870 6.0307 1

12 2 -2 3.03231 3.18226 1

-12 2 2 -1.1576 4.7431 2

-2 2 12 9.85969 3.68325 3

12 -2 2 1.68451 3.12018 1

2 -12 2 0.19211 4.04970 1

2 12 -2 3.51166 3.00533 3

-2 -2 -12 -3.2332 3.5034 2

2 -2 12 4.76476 3.78850 3

2 2 -12 1.56963 4.18441 2

-12 -2 -2 4.62425 4.15106 1

-2 -12 -2 4.65421 3.97971 1

-2 2 -12 0.54325 4.02776 2

-12 2 -2 2.42296 4.72207 2

2 12 2 0.93401 2.82699 3

2 -2 -12 4.94183 3.92294 2

-2 -2 12 1.63466 3.11108 3

2 2 12 -2.2391 3.3595 3

12 -2 -2 3.69344 3.14398 1

-2 -12 2 4.45810 4.38416 1

2 -12 -2 0.99306 3.71695 1

12 2 2 2.84963 4.33039 3

12 -3 2 37.9283 5.1107 1

3 -2 12 35.6143 5.9478 3

-12 3 2 37.9977 7.4147 2

-3 2 12 35.8001 5.2513 3

-12 -3 -2 47.9897 6.6442 1

-3 -2 -12 38.8183 6.4961 2

2 12 -3 40.6425 5.0204 3

-2 -12 -3 42.4558 6.2070 1

3 2 -12 39.1239 6.9375 2

2 -12 3 47.2758 6.7334 1

-3 -2 12 40.9539 5.2972 3

3 2 12 39.2091 5.8384 3

2 -12 -3 42.3244 6.3035 1

2 12 3 51.4536 5.2765 3

-3 2 -12 38.0296 7.1602 2

-12 3 -2 36.9279 7.1274 2

12 3 2 34.7822 6.1898 3

-12 -3 2 42.3134 6.5555 1

12 -3 -2 48.2301 5.6172 1

-2 -12 3 40.1337 6.3717 1

3 -2 -12 40.6409 6.1879 2

-4 2 12 7.77772 3.68285 3

-12 4 2 5.48755 6.17385 2

2 12 -4 10.0274 3.4889 3

-12 -4 -2 7.22680 4.70331 1

2 -12 4 6.54255 4.63998 1

4 -2 12 8.48613 4.60432 3

12 4 -2 7.84150 4.05221 3

-2 -12 -4 16.7069 4.8304 1

12 -4 2 14.9093 4.1045 1

-4 -2 -12 7.65377 4.55053 2

4 2 -12 6.25344 4.56144 2

-2 -12 4 4.36419 4.69335 1

-4 -2 12 13.0947 3.6462 3

-12 -4 2 14.8269 5.4024 1

-12 4 -2 17.1665 6.5424 2

2 -12 -4 8.76198 4.47879 1

-4 2 -12 19.1289 5.6843 2

4 -2 -12 11.7843 4.7190 2

12 -4 -2 17.7051 4.2295 1

4 2 12 4.37166 4.12738 3

12 4 2 15.8702 5.2243 3

12 5 -2 55.7127 6.7822 3

5 2 -12 59.7298 7.9840 2

-5 -2 -12 69.9946 8.4292 2

5 -2 12 52.1017 7.4587 3

-2 -12 -5 69.5692 7.5886 1

-12 -5 -2 83.1525 8.9553 1

2 -12 5 74.4967 8.2184 1

-12 5 2 51.3370 9.0978 2

12 -5 2 65.5534 6.7768 1

2 12 -5 70.2312 6.5004 3

-2 -12 5 60.0518 7.7945 1

12 5 2 70.5240 8.0189 3

2 -12 -5 66.5344 7.7731 1

-12 5 -2 56.9094 9.1952 2

12 -5 -2 80.4672 7.2919 1

-5 2 -12 62.2381 8.9362 2

-12 -5 2 62.2195 8.5025 1

-5 -2 12 71.4296 6.5559 3

5 2 12 71.5935 7.9762 3

5 -2 -12 77.7807 8.5049 2

-12 6 2 1.54933 6.05044 2

-6 -2 -12 0.71484 4.37130 2

2 12 -6 -1.6244 3.0739 3

12 -6 2 0.01030 3.77021 1

6 -2 12 -4.8638 4.2687 3

-2 -12 -6 0.45226 3.87260 1

6 2 -12 3.53105 4.42630 2

-12 -6 -2 0.06006 4.93839 1

12 6 -2 3.18912 3.88611 3

2 -12 -6 -5.2647 3.8687 1

6 2 12 4.24124 4.70226 3

-12 6 -2 2.57285 5.81103 2

12 -6 -2 2.76071 3.74397 1

-6 -2 12 -1.6350 3.0204 3

-12 -6 2 1.72787 5.48269 1

12 6 2 -3.6621 3.7034 3

-2 -12 6 -3.2527 4.7122 1

-6 2 -12 -5.3378 4.7399 2

6 -2 -12 3.93360 4.62737 2

12 -7 2 18.8139 5.0645 1

-2 -12 -7 18.1644 5.0554 1

-12 -7 -2 21.5905 6.6320 1

2 12 -7 11.8011 3.9416 3

-12 7 2 21.8008 7.5843 2

7 -2 12 23.1350 6.5976 3

12 7 -2 14.1949 4.7720 3

-7 -2 -12 21.0330 6.1222 2

7 2 12 6.19112 5.68116 3

-7 -2 12 18.9016 4.2805 3

12 -7 -2 7.31594 4.47833 1

7 -2 -12 16.2316 5.5732 2

-12 7 -2 7.02646 6.33579 2

2 -12 -7 17.3762 5.4723 1

-7 2 -12 12.0567 6.1439 2

12 7 2 15.4670 5.1986 3

-12 -7 2 15.9269 6.6129 1

12 8 -2 18.6184 5.3213 3

-12 8 2 32.1495 8.7450 2

-12 -8 -2 21.5656 7.2214 1

12 -8 2 22.5599 5.7033 1

2 12 -8 33.5139 5.1908 3

-8 -2 -12 21.3360 6.1583 2

8 -2 12 21.0704 6.9254 3

-8 2 12 18.0026 9.3841 2

8 2 12 30.2737 7.1586 3

-12 -8 2 16.4726 6.9272 1

2 -12 -8 19.6240 5.9045 1

12 8 2 22.6115 5.6322 3

-12 8 -2 27.9192 8.1206 2

-8 2 -12 16.3193 6.7445 2

12 -8 -2 26.5544 5.9157 1

0 3 -12 145.105 12.972 2

12 0 -3 136.629 10.245 1

12 0 3 149.174 10.297 1

0 3 12 161.948 10.980 3

3 -12 0 168.623 12.183 1

-3 -12 0 148.854 11.412 1

0 -3 -12 160.736 12.076 2

-3 12 0 165.247 12.234 2

3 12 0 171.966 10.308 3

0 -3 12 170.194 11.196 3

-3 -12 -1 3.07411 3.92232 1

-12 1 3 -0.8600 4.8027 2

3 12 -1 -0.0278 2.7437 3

-1 3 12 2.01919 3.42894 3

1 -3 12 0.70338 3.32560 3

3 -12 1 6.57238 4.54684 1

-3 12 1 2.25469 4.06487 2

1 3 -12 3.99925 4.62410 2

12 1 -3 4.07270 3.30074 1

-1 -3 -12 -2.6049 3.4793 2

12 -1 3 -5.2283 2.6958 1

3 12 1 -1.8375 2.6139 3

-3 12 -1 5.50363 4.56961 2

12 1 3 4.42211 3.53741 1

-12 1 -3 1.04533 4.39281 2

12 -1 -3 4.23579 3.26651 1

1 3 12 2.66250 3.61524 3

-1 -3 12 2.26907 3.50398 3

-1 3 -12 5.30868 4.97837 2

-3 -12 1 4.46635 4.16220 1

1 -3 -12 5.28934 3.72073 2

3 -12 -1 4.74864 4.27695 1

2 3 -12 33.0316 6.8283 2

-2 3 12 55.8081 6.0275 3

12 2 -3 45.5930 5.5847 1

2 -3 12 45.8344 6.2516 3

-3 12 2 44.7269 6.4843 2

12 -2 3 48.2340 5.3160 1

-3 -12 -2 50.5131 6.4708 1

3 -12 2 42.3772 6.5906 1

-12 2 3 27.6845 6.9778 2

-12 -2 -3 45.6310 6.4112 1

-2 -3 -12 51.1295 6.8022 2

3 12 -2 41.3456 5.1274 3

3 12 2 43.0905 5.1357 3

12 -2 -3 45.1685 5.4405 1

2 -3 -12 46.9480 6.3757 2

-2 -3 12 40.9375 5.5037 3

-3 -12 2 47.5430 6.6726 1

-12 2 -3 37.7448 7.0728 2

2 3 12 41.1178 5.9907 3

-2 3 -12 42.8515 7.3822 2

3 -12 -2 45.7727 6.3680 1

12 2 3 38.3109 7.0784 3

-12 -3 -3 4.66442 4.56655 1

-3 -12 -3 13.4711 4.9363 1

-3 3 12 8.94963 3.77335 3

3 -3 12 7.47180 4.37160 3

3 12 -3 4.08733 3.20044 3

-3 -3 -12 2.86601 4.04296 2

12 -3 3 4.09679 3.32217 1

-12 3 3 15.5043 6.2697 2

-3 12 3 13.9491 4.7836 2

3 3 -12 11.6952 5.2309 2

3 -12 3 14.8084 5.0886 1

-12 -3 3 13.3657 4.9276 1

-12 3 -3 5.23903 5.43304 2

-3 -12 3 13.0853 4.9120 1

12 -3 -3 6.99650 3.56651 1

-3 3 -12 11.2790 5.2756 2

3 3 12 9.06509 4.29724 3

12 3 3 4.26200 4.80140 3

3 -12 -3 8.58993 4.65232 1

3 12 3 8.29352 3.44983 3

-3 -3 12 7.23700 3.54048 3

3 -3 -12 12.9259 4.2993 2

-3 -12 -4 139.898 10.440 1

4 -3 12 112.289 10.236 3

3 -12 4 122.972 10.304 1

-3 12 4 106.429 10.484 2

-12 4 3 98.6204 11.1972 2

-12 -4 -3 125.251 10.735 1

3 12 -4 123.889 8.777 3

12 4 -3 114.876 9.135 3

12 -4 3 134.597 9.255 1

4 3 -12 103.702 10.638 2

-4 -3 -12 114.489 10.764 2

12 -4 -3 121.573 9.186 1

4 3 12 115.038 9.988 3

12 4 3 119.243 10.867 3

-12 4 -3 116.431 11.927 2

3 -12 -4 113.621 10.332 1

-4 3 -12 112.831 11.753 2

-3 -12 4 116.258 10.398 1

-12 -4 3 111.268 10.564 1

-4 -3 12 120.897 8.921 3

3 12 4 108.456 8.581 3

4 -3 -12 117.659 10.483 2

5 -3 12 22.8732 5.9091 3

3 12 -5 22.3729 4.2867 3

12 5 -3 13.5909 4.3312 3

3 -12 5 22.8015 5.8432 1

-5 -3 -12 16.5076 5.6134 2

5 3 -12 16.3029 5.4953 2

-3 -12 -5 22.8415 5.2947 1

-12 5 3 18.1975 6.7099 2

-12 -5 -3 23.3053 6.2927 1

-3 12 5 18.5982 5.5538 2

12 -5 3 18.2058 4.5455 1

5 -3 -12 24.5578 5.7685 2

-3 -12 5 13.2987 5.4545 1

-12 -5 3 12.8114 5.8859 1

-12 5 -3 19.2449 7.0051 2

12 5 3 18.1408 5.6401 3

3 12 5 19.2090 4.3605 3

12 -5 -3 16.0495 4.4822 1

-5 -3 12 14.8504 4.1398 3

3 -12 -5 17.5918 5.3131 1

-5 3 -12 20.8778 6.4853 2

5 3 12 20.7913 5.6200 3

6 -3 12 38.2134 7.1957 3

-3 12 6 41.3886 6.8145 2

12 6 -3 35.7206 5.7415 3

3 12 -6 48.8430 5.5866 3

-3 -12 -6 45.6852 6.4825 1

-6 -3 -12 30.0944 6.5434 2

-12 -6 -3 28.0867 6.9199 1

12 -6 3 47.5579 6.1995 1

-12 6 3 29.5097 7.9483 2

-3 -12 6 38.5902 6.8916 1

6 3 12 31.5769 6.3818 3

-12 -6 3 38.2293 7.6752 1

-12 6 -3 32.0237 8.2348 2

12 6 3 32.1429 6.2883 3

3 -12 -6 43.2101 6.7037 1

6 -3 -12 35.6614 6.8634 2

-6 3 -12 36.6647 7.8363 2

-6 -3 12 38.6156 5.4967 3

12 -6 -3 31.6361 5.4572 1

-12 -7 -3 16.0508 6.5574 1

-3 12 7 9.23948 5.81903 2

-7 -3 -12 24.0432 6.2447 2

-12 7 3 26.7334 7.9953 2

12 7 -3 15.3874 4.9208 3

7 -3 12 19.4940 6.4187 3

12 -7 3 18.2192 5.3081 1

3 12 -7 20.0488 4.3346 3

-3 -12 -7 14.6652 5.2192 1

12 -7 -3 13.6864 4.9599 1

3 -12 -7 20.5132 5.5327 1

7 -3 -12 18.8174 6.0848 2

-12 -7 3 13.1367 6.6202 1

12 7 3 20.5509 5.4456 3

-7 3 -12 25.7088 7.5193 2

7 3 12 25.7477 6.4720 3

-7 -3 12 18.4097 4.5312 3

-12 7 -3 19.1659 7.5718 2

12 -8 3 117.982 10.149 1

3 12 -8 102.820 8.614 3

-12 -8 -3 90.3312 10.4687 1

-3 12 8 93.7463 10.6425 2

-8 3 12 76.0182 13.1004 2

8 -3 12 95.2050 10.6244 3

-12 8 3 106.230 12.707 2

12 8 -3 93.9837 8.8777 3

-8 3 -12 109.365 12.110 2

3 -12 -8 89.1820 9.4860 1

-12 -8 3 123.411 12.170 1

12 8 3 92.5822 9.3196 3

12 -8 -3 93.0847 9.1266 1

-12 8 -3 92.3734 12.1335 2

8 3 12 93.8381 10.4259 3

12 0 4 889.744 39.603 1

4 12 0 804.937 38.867 3

0 -4 12 842.953 40.225 3

12 0 -4 825.829 39.831 1

-4 -12 0 809.706 40.832 1

0 -4 -12 797.563 42.847 2

0 4 12 824.981 39.703 3

4 -12 0 780.266 41.120 1

0 4 -12 803.599 44.861 2

-4 12 0 897.329 43.407 2

4 -12 1 214.756 14.890 1

-1 4 12 235.991 13.899 3

-4 -12 -1 230.631 14.565 1

12 -1 4 225.447 13.301 1

1 -4 12 219.633 14.241 3

-1 -4 -12 220.235 15.058 2

-4 12 1 230.764 15.529 2

12 1 -4 220.543 13.364 1

-12 -1 -4 206.251 13.840 1

1 4 -12 202.443 16.303 2

4 12 -1 234.874 13.023 3

4 -12 -1 243.122 15.458 1

1 -4 -12 228.183 15.172 2

-12 1 -4 219.667 15.843 2

-1 -4 12 240.362 14.469 3

-1 4 -12 215.823 16.948 2

12 1 4 250.328 16.373 3

-4 -12 1 238.467 15.311 1

-4 12 -1 236.159 15.924 2

1 4 12 227.999 14.208 3

4 12 1 221.559 13.352 3

12 -1 -4 216.519 13.671 1

-2 -4 -12 11.8420 4.4630 2

-4 -12 -2 5.44793 4.54314 1

12 -2 4 1.14667 3.48696 1

-12 -2 -4 4.25090 4.28557 1

2 4 -12 7.98504 5.24617 2

-4 12 2 12.4942 4.9719 2

-2 4 12 7.55999 3.97896 3

4 12 -2 17.6798 3.7504 3

-12 2 4 5.80541 5.57435 2

12 2 -4 4.56378 3.54518 1

2 -4 12 5.92509 4.10886 3

4 -12 2 14.0201 5.1075 1

12 -2 -4 3.38670 3.52205 1

4 12 2 6.89209 3.43496 3

-2 -4 12 10.4396 3.9765 3

-4 12 -2 11.0510 5.4300 2

2 -4 -12 11.8379 4.3557 2

-12 2 -4 22.3831 6.0656 2

-2 4 -12 15.2856 5.9642 2

-4 -12 2 16.5594 5.2583 1

4 -12 -2 10.2278 4.6387 1

12 2 4 13.3864 5.7968 3

2 4 12 13.6062 4.4466 3

12 -3 4 127.433 9.012 1

-4 -12 -3 133.226 10.330 1

3 4 -12 107.957 11.011 2

-3 -4 -12 134.485 10.736 2

4 -12 3 116.995 10.269 1

4 12 -3 128.454 8.980 3

-12 -3 -4 118.923 10.571 1

-12 3 4 103.319 11.453 2

-4 12 3 110.420 10.742 2

3 -4 12 123.954 10.542 3

4 12 3 120.568 8.977 3

3 -4 -12 112.491 10.331 2

3 4 12 123.559 10.098 3

-12 -3 4 121.565 10.781 1

-4 12 -3 153.408 12.596 2

12 -3 -4 121.943 9.347 1

-3 4 -12 115.177 12.076 2

4 -12 -3 125.019 10.488 1

-12 3 -4 120.555 11.669 2

12 3 4 118.608 11.060 3

-4 -12 3 93.0744 9.8017 1

-3 -4 12 152.874 10.066 3

-12 4 4 507.304 33.262 2

4 -12 4 587.399 30.502 1

-4 12 4 573.159 32.189 2

-12 -4 -4 574.888 30.900 1

-4 -12 -4 657.508 30.829 1

4 4 -12 548.587 32.603 2

4 12 -4 593.772 28.891 3

-4 -4 -12 602.492 32.306 2

4 -4 12 613.441 31.110 3

12 -4 4 611.068 29.238 1

12 4 4 572.405 31.131 3

4 12 4 559.033 28.585 3

4 -4 -12 587.685 32.172 2

12 -4 -4 577.341 29.354 1

4 -12 -4 554.748 30.631 1

-4 -12 4 546.345 30.509 1

-4 4 -12 602.436 34.306 2

-4 -4 12 573.469 29.133 3

-12 4 -4 551.952 33.558 2

-4 12 -4 703.607 34.105 2

4 4 12 582.096 30.061 3

-12 -5 -4 221.782 15.786 1

-12 5 4 202.058 17.553 2

-5 -4 -12 236.790 16.060 2

-4 12 5 226.834 16.237 2

4 12 -5 240.897 13.841 3

5 -4 12 247.242 16.239 3

12 5 -4 217.395 14.116 3

-4 -12 -5 280.207 15.739 1

5 4 12 235.931 15.596 3

4 12 5 220.609 13.948 3

4 -12 -5 247.456 15.712 1

-12 5 -4 216.067 17.707 2

-5 4 -12 235.409 18.089 2

-4 -12 5 226.609 15.534 1

12 -5 -4 221.318 14.243 1

-12 -5 4 209.523 16.090 1

5 -4 -12 207.692 15.625 2

12 5 4 242.103 16.167 3

-5 -4 12 224.795 14.055 3

-4 12 -5 287.606 18.073 2

4 12 -6 15.7155 4.1466 3

6 -4 12 14.9251 6.0108 3

-12 -6 -4 16.4030 6.1339 1

12 6 -4 8.97910 4.23852 3

-6 -4 -12 4.93131 4.85720 2

-4 -12 -6 19.3781 5.1940 1

-12 6 4 17.2613 7.6564 2

-4 12 6 11.6407 5.4431 2

-6 -4 12 10.3652 4.1539 3

-4 -12 6 12.3334 5.6400 1

12 -6 -4 11.4030 4.3805 1

12 6 4 13.3218 5.5784 3

-12 -6 4 25.2721 7.0343 1

6 4 12 13.2704 5.6823 3

-6 4 -12 18.4960 6.8707 2

4 -12 -6 8.87213 4.92373 1

-12 6 -4 5.74539 6.38175 2

6 -4 -12 12.5072 4.9170 2

4 12 6 11.1959 4.4764 3

-12 7 4 80.4533 10.9978 2

-4 -12 -7 71.9229 7.6462 1

4 12 -7 47.4222 5.9957 3

-7 4 12 67.5496 11.3157 2

12 7 -4 38.0389 6.1100 3

-4 12 7 51.6278 8.1262 2

-12 -7 -4 49.2434 8.1810 1

7 -4 12 42.7826 7.8242 3

7 4 12 57.9321 8.2751 3

-7 4 -12 50.0552 9.2492 2

4 12 7 52.0072 6.6685 3

-7 -4 12 56.6758 6.6200 3

12 7 4 51.8637 7.6704 3

12 -7 -4 49.6674 6.8063 1

-12 -7 4 54.0866 9.0864 1

4 -12 -7 46.2821 7.2670 1

-12 7 -4 53.1000 9.7596 2

-4 12 8 378.750 24.777 2

4 12 -8 418.476 21.553 3

-8 4 12 360.008 28.236 2

12 8 -4 419.105 22.273 3

4 -12 -8 389.220 22.652 1

12 8 4 386.680 22.344 3

0 -5 -12 282.967 17.339 2

0 5 12 280.869 16.398 3

5 -12 0 266.746 17.250 1

0 5 -12 225.352 18.543 2

-5 12 0 293.995 18.161 2

0 -5 12 294.688 16.923 3

12 0 -5 262.269 15.726 1

5 12 0 279.849 15.433 3

-5 -12 0 281.285 17.447 1

-1 5 12 33.9607 5.3936 3

-5 -12 -1 31.7457 6.2010 1

-5 12 1 22.3171 5.8998 2

5 12 -1 21.5596 4.1645 3

5 -12 1 15.4627 5.4606 1

12 1 -5 20.9235 4.4927 1

1 5 -12 25.3734 6.6739 2

1 -5 12 29.5511 5.6632 3

-1 -5 -12 26.7752 5.4018 2

-5 -12 1 37.5932 6.4424 1

-1 -5 12 29.3309 5.3948 3

1 -5 -12 19.9477 4.8893 2

5 -12 -1 30.9065 5.9752 1

12 1 5 22.0825 6.6243 3

5 12 1 20.3864 4.1033 3

-12 1 -5 23.6481 5.9413 2

-5 12 -1 34.0388 6.3473 2

-1 5 -12 22.7595 6.6233 2

1 5 12 21.0780 5.0378 3

12 -1 -5 15.9419 4.2505 1

-5 12 2 69.0190 8.1152 2

-12 2 5 46.3013 8.5600 2

-5 -12 -2 70.9417 7.8579 1

5 -12 2 64.2833 7.7938 1

-2 -5 -12 75.1193 7.7814 2

2 5 -12 48.3135 8.1396 2

5 12 -2 76.2449 6.5569 3

2 -5 12 66.8891 7.7085 3

12 2 -5 69.9869 6.9199 1

-2 -5 12 60.1096 6.9925 3

12 2 5 68.5036 8.7379 3

5 12 2 72.6664 6.6478 3

-5 12 -2 67.4869 8.3650 2

5 -12 -2 52.8123 7.4823 1

-2 5 -12 69.5824 9.4478 2

12 -2 -5 53.9648 6.4366 1

2 -5 -12 71.0308 7.7609 2

-5 -12 2 68.3000 8.3227 1

-12 2 -5 64.5749 8.5859 2

2 5 12 69.1453 7.5622 3

-3 -5 -12 14.3845 4.9326 2

-5 12 3 22.2765 5.7495 2

-12 3 5 18.3712 7.2236 2

-5 -12 -3 23.6431 5.5407 1

3 -5 12 20.6041 5.8234 3

5 12 -3 17.5151 4.1314 3

3 5 -12 20.9958 6.0772 2

5 -12 3 19.1329 5.6301 1

-3 -5 12 19.9918 4.9113 3

12 3 5 18.3274 6.1199 3

-3 5 -12 17.3120 6.7513 2

-5 -12 3 6.65483 5.18532 1

3 5 12 21.3455 5.4657 3

-5 12 -3 22.9982 6.5718 2

3 -5 -12 16.9780 4.9693 2

-12 3 -5 20.3268 6.6488 2

12 -3 -5 22.3734 4.5784 1

-12 -3 5 20.3603 5.8926 1

5 12 3 23.0232 4.5232 3

5 -12 -3 10.9324 5.0687 1

5 -12 4 262.041 15.952 1

-4 -5 -12 246.215 15.987 2

-12 4 5 203.520 17.761 2

-5 -12 -4 261.055 15.587 1

5 12 -4 227.725 13.900 3

-5 12 4 242.675 16.737 2

4 -5 12 223.156 16.001 3

5 12 4 222.330 13.880 3

-5 12 -4 291.147 18.562 2

-12 4 -5 226.292 17.745 2

-4 -5 12 218.848 14.448 3

5 -12 -4 202.967 15.024 1

-4 5 -12 253.401 18.577 2

12 -4 -5 227.077 14.313 1

-5 -12 4 223.045 15.789 1

-12 -4 5 212.088 16.047 1

4 -5 -12 238.158 15.953 2

4 5 12 234.709 15.493 3

12 4 5 245.667 16.465 3

-12 5 5 36.0553 8.7835 2

5 12 -5 47.1610 5.6277 3

12 5 -5 41.8925 5.7544 3

-5 12 5 37.1174 7.0953 2

5 -5 12 41.4395 7.6108 3

-5 -5 -12 41.1830 6.6301 2

-5 -12 -5 57.9022 7.0908 1

-5 -5 12 38.9976 6.1246 3

12 -5 -5 36.4338 5.8153 1

5 5 12 33.0695 6.6974 3

12 5 5 33.0086 6.8859 3

5 -5 -12 47.2412 6.8850 2

-5 5 -12 41.3248 8.5210 2

5 -12 -5 27.4129 6.0977 1

-12 5 -5 32.5697 8.1738 2

-5 -12 5 23.3910 6.5502 1

-12 -5 5 46.5148 7.9205 1

-5 12 -5 51.9366 8.3773 2

5 12 5 37.3558 5.6307 3

5 12 -6 54.6045 6.0947 3

-5 12 6 43.3943 7.7388 2

-12 6 5 55.7780 9.9918 2

6 -5 12 43.3094 7.7635 3

12 6 -5 47.8386 6.2411 3

-5 -12 -6 50.3370 6.9885 1

5 12 6 52.5611 6.6629 3

-5 12 -6 55.0145 9.1589 2

-12 6 -5 49.1313 9.3665 2

6 5 12 57.1842 8.1567 3

6 -5 -12 55.7443 7.8631 2

-6 -5 12 55.4331 6.8812 3

-12 -6 5 50.6502 8.6679 1

12 6 5 58.0472 8.1451 3

5 -12 -6 43.5546 7.1437 1

-5 -12 6 49.2491 7.8308 1

-6 5 -12 51.9510 9.2277 2

12 -6 -5 53.6670 6.8179 1

12 7 -5 5.87958 4.16911 3

-5 12 7 16.1793 6.3711 2

-7 5 12 4.98188 8.04103 2

-12 7 5 14.6623 8.1965 2

5 12 -7 20.0497 4.4883 3

7 -5 12 5.08549 6.42758 3

12 -7 -5 10.5283 4.6836 1

-12 7 -5 15.6626 7.9032 2

5 12 7 10.7583 4.8458 3

5 -12 -7 7.47967 5.27662 1

-7 -5 12 7.21673 4.43939 3

-7 5 -12 9.68987 7.11588 2

7 5 12 8.76762 5.95848 3

-5 12 -7 22.9400 7.4895 2

12 7 5 8.70989 5.46686 3

-12 -7 5 12.5103 7.1807 1

0 6 12 3.42675 4.48904 3

0 -6 12 6.55793 4.28685 3

6 -12 0 4.78492 4.62812 1

0 -6 -12 3.29753 3.83366 2

-6 12 0 5.51175 4.91438 2

0 6 -12 -2.2922 4.6778 2

12 0 -6 2.14054 3.44294 1

-6 -12 0 6.24255 5.16546 1

6 12 0 2.99251 3.16275 3

1 6 -12 57.0492 8.9471 2

12 1 -6 61.5143 6.5840 1

-1 -6 -12 76.5000 7.5599 2

-6 -12 -1 53.4744 7.7700 1

6 -12 1 62.1616 7.6553 1

1 -6 12 51.5041 7.4308 3

-6 12 1 58.9583 7.9937 2

6 12 -1 66.9948 6.3854 3

-1 -6 12 62.6940 7.4191 3

-12 1 -6 58.9624 8.2237 2

1 -6 -12 61.7912 7.0864 2

-6 12 -1 53.8421 7.7404 2

12 -1 -6 52.1932 6.2728 1

6 12 1 60.5836 6.1308 3

-6 -12 1 62.8170 7.9698 1

12 1 6 65.1306 8.6233 3

-1 6 -12 44.6798 8.5297 2

6 -12 -1 46.1440 7.2640 1

1 6 12 44.4367 6.8710 3

2 6 -12 1.90720 5.03773 2

-12 2 6 2.79709 6.00965 2

2 -6 12 -1.5709 4.0927 3

-6 12 2 1.12722 4.47542 2

-6 -12 -2 0.95782 4.59808 1

-2 -6 -12 -0.1482 3.6053 2

12 2 -6 1.49606 3.56490 1

6 -12 2 1.09546 4.24761 1

6 12 -2 0.09837 3.12544 3

-6 12 -2 5.29588 5.14769 2

-12 2 -6 0.66842 5.10957 2

6 12 2 1.65620 3.19990 3

2 -6 -12 -0.2052 3.4620 2

2 6 12 2.90534 4.43470 3

6 -12 -2 4.74558 4.50446 1

-2 -6 12 -0.8417 3.8762 3

-2 6 -12 -1.8630 5.3035 2

12 -2 -6 -0.9947 3.3858 1

-6 -12 2 0.55652 4.87349 1

12 2 6 1.71102 5.34933 3

6 -12 3 37.9435 6.4483 1

6 12 -3 38.7994 5.1922 3

-6 12 3 30.9659 6.7087 2

-3 -6 -12 43.0575 6.1034 2

3 -6 12 31.5735 6.6374 3

-6 -12 -3 39.6139 6.7697 1

-12 3 6 25.1734 7.9223 2

-6 12 -3 30.7659 7.1556 2

3 -6 -12 40.3541 6.4244 2

6 -12 -3 26.8834 6.2106 1

-3 6 -12 46.7866 8.9781 2

12 3 6 34.1199 7.2833 3

-12 3 -6 35.6164 7.6013 2

12 -3 -6 29.8682 5.2546 1

3 6 12 40.1088 6.7365 3

6 12 3 36.0575 5.4192 3

-6 -12 3 38.1516 7.0760 1

-12 -3 6 31.5791 6.6498 1

-3 -6 12 50.3856 6.6641 3

6 12 -4 16.9425 4.0986 3

-6 12 4 13.1586 5.9410 2

-6 -12 -4 13.4699 5.2884 1

6 -12 4 15.5002 5.2785 1

4 -6 12 10.6077 5.6807 3

-12 4 6 13.3926 7.5297 2

-4 -6 -12 18.1611 5.1787 2

4 6 12 3.80356 4.99462 3

12 -4 -6 12.0817 4.3989 1

-4 -6 12 11.0977 4.7632 3

12 4 6 25.3753 6.6696 3

-4 6 -12 13.9900 7.0547 2

4 -6 -12 10.5839 4.8847 2

6 12 4 12.5246 4.2203 3

-6 -12 4 16.2162 5.8272 1

-12 4 -6 13.4608 6.7994 2

-6 12 -4 20.3200 6.7760 2

6 -12 -4 5.48861 4.61274 1

-12 -4 6 10.1494 6.0453 1

5 -6 12 58.9829 8.5689 3

6 12 -5 57.8663 6.2831 3

-6 -12 -5 56.1882 7.3406 1

-12 5 6 53.3693 9.8999 2

-6 12 5 46.2252 8.1754 2

6 12 5 56.2973 6.4997 3

-6 12 -5 69.3052 9.5672 2

5 -6 -12 48.8854 7.3591 2

-12 -5 6 35.1730 7.9898 1

-12 5 -6 45.0035 9.0918 2

12 5 6 41.9164 7.5488 3

12 -5 -6 51.2613 6.5269 1

5 6 12 50.1582 7.6638 3

-5 -6 12 49.4027 6.9604 3

-5 6 -12 46.5301 9.3726 2

-6 -12 5 51.7260 7.9559 1

6 -12 -5 36.1617 6.8890 1

-6 6 12 5.57353 7.32009 2

-6 12 6 0.80869 5.34027 2

6 -6 12 6.62808 6.08868 3

6 12 -6 2.17027 3.60644 3

-6 -12 -6 1.74363 4.78038 1

12 6 -6 -0.5342 3.5863 3

-12 6 6 13.5110 7.9884 2

12 -6 -6 -0.7078 4.2272 1

-12 6 -6 1.10359 6.74410 2

-6 12 -6 0.24317 6.06730 2

-6 6 -12 9.54870 7.10980 2

-12 -6 6 9.87185 6.69749 1

6 12 6 0.37418 3.92340 3

-6 -6 12 2.56903 4.26391 3

6 -12 -6 -1.2099 4.7888 1

12 6 6 8.22201 5.54828 3

6 6 12 6.40269 5.53664 3

-6 -12 6 -1.7433 5.6796 1

-7 -12 0 48.8838 7.5957 1

0 -7 12 46.4571 6.9649 3

0 7 -12 36.4982 8.0912 2

-7 12 0 47.5904 7.8954 2

12 0 -7 46.3313 6.1356 1

7 12 0 50.2058 5.8524 3

0 -7 -12 59.6185 6.9650 2

7 -12 0 64.4980 7.7202 1

7 -12 1 11.6665 5.0553 1

-1 -7 -12 13.6845 4.9146 2

-7 -12 -1 3.79519 5.06813 1

7 12 -1 6.72044 3.53724 3

12 1 -7 5.82288 3.75954 1

1 7 -12 3.32925 5.76589 2

1 -7 12 7.98818 4.99597 3

-7 12 1 4.49366 4.81378 2

12 1 7 2.68598 5.83223 3

7 -12 -1 2.86549 4.60284 1

12 -1 -7 7.31707 4.01052 1

1 7 12 19.1844 5.5450 3

-1 -7 12 11.6572 5.1464 3

1 -7 -12 10.4346 4.4140 2

-7 12 -1 7.69903 5.55667 2

-12 1 -7 6.86036 5.47694 2

-1 7 -12 7.72727 6.21800 2

-7 -12 1 9.41061 5.33123 1

7 12 1 10.2584 3.9731 3

-12 2 7 8.78333 6.96758 2

-7 12 2 6.37744 5.47450 2

7 -12 2 5.23138 4.93420 1

-2 -7 -12 18.6345 5.0048 2

-7 -12 -2 24.5359 6.2442 1

2 -7 12 18.5480 6.0150 3

7 12 -2 11.3914 3.8699 3

12 2 7 21.6767 6.6173 3

-7 12 -2 24.3765 6.5802 2

7 -12 -2 18.9443 5.7003 1

2 -7 -12 18.8383 5.1734 2

2 7 12 17.1541 5.4710 3

-2 -7 12 20.1448 5.7072 3

-2 7 -12 12.3196 6.7475 2

-12 2 -7 7.68637 6.18154 2

-7 -12 2 19.5558 6.0654 1

7 12 2 11.3700 4.0995 3

12 -2 -7 12.6683 4.2111 1

-12 -2 7 14.7750 5.7581 1

-3 -7 -12 11.4107 4.7096 2

-12 3 7 19.4903 7.9871 2

-7 -12 -3 17.7318 6.2641 1

3 -7 12 11.9345 5.6371 3

7 12 -3 15.2763 4.0504 3

7 -12 3 20.9219 5.9939 1

-7 12 3 23.8648 6.3749 2

12 3 7 16.5323 6.4291 3

3 7 12 16.5970 5.5717 3

3 -7 -12 26.1854 5.5373 2

7 -12 -3 11.6207 5.3220 1

-12 3 -7 15.4990 6.9460 2

12 -3 -7 13.4703 4.5356 1

-7 12 -3 16.6585 6.5796 2

-12 -3 7 14.9545 6.1050 1

7 12 3 14.9454 4.4800 3

-7 -12 3 16.4028 6.0283 1

-3 7 -12 21.8959 7.6946 2

-3 -7 12 20.3167 5.6965 3

-12 4 7 51.8141 10.3396 2

7 12 -4 57.6895 6.3998 3

-7 -12 -4 54.3508 7.8742 1

4 -7 12 61.1135 8.6744 3

-7 12 4 55.8011 8.6610 2

-4 -7 12 48.0230 7.3552 3

-12 4 -7 51.7945 9.5220 2

7 -12 -4 37.2862 7.0761 1

-12 -4 7 57.8075 8.8180 1

12 -4 -7 55.9447 6.7837 1

-7 -12 4 51.0043 8.0151 1

12 4 7 54.2029 8.4604 3

-4 7 -12 55.1816 10.0334 2

7 12 4 44.6431 6.2152 3

-7 12 -4 59.6108 9.1166 2

4 -7 -12 66.3037 7.8619 2

4 7 12 58.5795 7.9627 3

-5 7 12 15.5057 7.6787 2

7 12 -5 12.0242 4.1336 3

-7 12 5 14.0464 6.8757 2

5 -7 12 6.64705 6.29623 3

-12 5 7 15.7383 8.1816 2

-7 -12 -5 11.5782 5.6184 1

-7 12 -5 25.2885 7.5038 2

-5 7 -12 8.90338 7.25036 2

-7 -12 5 6.52358 5.86801 1

5 7 12 15.8582 6.1335 3

-12 -5 7 12.2880 6.7707 1

-12 5 -7 15.5533 7.4157 2

12 5 7 9.37574 6.03437 3

7 12 5 18.3354 4.6719 3

-5 -7 12 8.30209 5.21915 3

12 -5 -7 14.1963 4.9947 1

7 -12 -5 9.57531 5.53978 1

-8 12 0 402.039 24.939 2

12 0 -8 365.427 21.550 1

0 -8 -12 432.585 23.595 2

-8 -12 0 430.846 24.021 1

8 -12 0 402.157 23.331 1

0 -8 12 391.019 23.296 3

8 12 0 411.753 21.623 3

8 12 -1 159.602 11.093 3

-8 12 1 160.338 14.113 2

-1 -8 -12 172.399 12.428 2

-8 -12 -1 172.536 13.582 1

8 -12 1 150.828 12.525 1

12 1 -8 148.137 11.184 1

1 -8 12 144.760 12.837 3

12 1 8 189.021 14.512 3

8 -12 -1 153.870 12.916 1

1 -8 -12 185.877 12.888 2

12 -1 -8 133.416 11.031 1

-1 -8 12 168.374 13.193 3

-8 12 -1 152.048 13.979 2

8 12 1 160.819 11.325 3

-8 -12 1 184.143 13.853 1

-1 8 -12 144.830 14.736 2

-12 1 -8 169.603 14.017 2

-8 12 2 18.0956 6.8311 2

8 12 -2 23.9103 4.6349 3

-2 -8 -12 29.1459 5.8152 2

-8 -12 -2 22.5475 6.7825 1

2 -8 12 10.1321 5.9969 3

8 -12 2 28.5501 6.4359 1

12 2 8 33.8200 7.5253 3

-2 -8 12 27.2026 6.4090 3

-8 -12 2 21.9377 7.0591 1

2 8 12 29.7604 6.5650 3

-8 12 -2 22.6097 6.9609 2

-12 -2 8 18.2829 6.5562 1

2 -8 -12 24.2422 5.7124 2

12 -2 -8 24.6647 5.2650 1

8 12 2 29.3604 5.4776 3

-2 8 -12 21.9247 8.0008 2

-12 2 -8 25.3005 7.4170 2

8 -12 -2 26.6226 6.6337 1

3 -8 12 98.3606 10.4805 3

8 12 -3 107.366 8.770 3

-8 12 3 100.807 11.238 2

-12 3 8 89.9885 12.3106 2

-8 -12 -3 129.756 11.221 1

8 -12 3 97.6378 9.9647 1

-3 8 -12 99.9908 12.5341 2

-12 -3 8 89.8896 10.4621 1

-8 12 -3 109.451 11.834 2

-3 -8 12 97.9824 10.0462 3

8 -12 -3 104.680 10.349 1

12 3 8 94.3557 10.6440 3

-12 3 -8 112.684 12.294 2

-8 -12 3 94.0087 10.4979 1

3 8 12 103.509 10.167 3

3 -8 -12 106.227 9.959 2

8 12 3 100.622 8.900 3

12 -3 -8 105.321 9.273 1

4 -8 12 367.102 22.641 3

-12 4 8 367.143 25.559 2

-12 -4 8 387.081 24.392 1

12 -4 -8 395.512 22.266 1

8 12 4 420.450 22.085 3

0 0 -13 1.49645 4.12990 2

0 -13 0 -3.2410 4.2476 1

13 0 0 -1.1228 3.4380 1

0 0 13 -0.2659 3.6406 3

1 0 -13 314.604 20.867 2

13 1 0 325.830 18.364 1

13 -1 0 353.098 18.566 1

0 -13 1 360.818 20.052 1

0 -13 -1 334.017 19.755 1

-1 0 13 334.840 18.645 3

-1 0 -13 321.012 21.196 2

1 0 13 322.042 18.829 3

0 -13 -2 -3.1002 3.7000 1

2 0 13 1.81382 3.78794 3

13 -2 0 -1.3264 3.3104 1

-13 2 0 2.00276 5.23922 2

0 -13 2 -2.2855 4.0763 1

-2 0 13 1.39603 3.49282 3

2 0 -13 -0.1415 4.0614 2

-13 -2 0 -0.3641 4.2865 1

-2 0 -13 -1.4400 3.8401 2

0 -13 3 240.059 15.699 1

-13 3 0 199.814 16.526 2

-3 0 13 250.482 14.364 3

3 0 -13 218.579 16.161 2

-3 0 -13 220.676 16.540 2

13 -3 0 261.341 14.469 1

0 -13 -3 220.556 15.128 1

3 0 13 217.240 14.753 3

-13 -3 0 241.932 15.759 1

13 -4 0 37.1134 5.5559 1

-4 0 13 40.7026 5.5583 3

0 -13 4 31.3787 6.7069 1

4 0 13 34.8740 6.3349 3

0 -13 -4 40.2645 6.3791 1

13 4 0 26.4652 5.5845 3

4 0 -13 34.6770 6.6911 2

-4 0 -13 36.4610 7.1275 2

-13 -4 0 32.4252 6.8104 1

-13 4 0 22.1697 7.5415 2

5 0 13 218.331 15.729 3

-13 5 0 219.935 18.299 2

5 0 -13 236.996 17.168 2

0 -13 -5 273.247 16.004 1

13 -5 0 256.540 15.309 1

0 -13 5 225.302 16.056 1

-13 -5 0 230.144 16.833 1

-5 0 -13 278.007 18.010 2

13 5 0 240.398 15.540 3

-5 0 13 251.202 14.735 3

0 -13 -6 1.81232 4.13323 1

-13 -6 0 -8.3886 5.0830 1

-6 0 -13 2.51495 5.25514 2

-13 6 0 6.29999 6.68092 2

6 0 13 0.54461 4.86512 3

13 6 0 6.44116 4.23257 3

13 -6 0 0.15688 4.04450 1

6 0 -13 0.13008 4.65481 2

7 0 -13 125.060 12.062 2

13 -7 0 140.438 11.131 1

-13 -7 0 118.177 12.494 1

-7 0 -13 149.730 13.165 2

7 0 13 114.161 11.644 3

13 7 0 126.590 10.868 3

0 -13 -7 140.017 11.320 1

-13 7 0 109.028 13.258 2

0 -1 -13 306.335 20.308 2

0 1 -13 310.275 20.996 2

-1 -13 0 332.054 19.386 1

0 -1 13 305.412 18.345 3

13 0 -1 339.705 18.249 1

1 -13 0 342.718 19.883 1

13 0 1 352.184 18.161 1

0 1 13 315.018 18.231 3

-1 -13 -1 155.415 12.059 1

13 -1 1 167.590 11.035 1

-1 -1 -13 184.823 13.405 2

13 1 -1 158.468 10.888 1

1 1 -13 150.282 13.129 2

-1 1 13 164.678 11.410 3

1 -1 13 144.463 11.488 3

1 -13 1 156.663 12.445 1

13 -1 -1 168.314 10.907 1

1 -13 -1 126.635 11.425 1

13 1 1 164.483 10.772 1

1 -1 -13 157.087 12.384 2

-1 -13 1 162.417 12.082 1

-1 1 -13 143.869 12.982 2

1 1 13 135.962 10.803 3

-1 -1 13 157.506 11.004 3

-2 -1 -13 31.8000 6.5184 2

-13 -2 -1 31.7278 6.0000 1

2 1 -13 38.0524 6.8966 2

13 2 -1 38.4793 5.6449 1

-1 -13 -2 35.0080 6.0284 1

-13 2 1 40.0717 7.0373 2

13 -2 1 34.0165 5.2362 1

1 -13 2 30.3897 6.2902 1

-2 1 13 39.4074 5.6722 3

2 -1 13 37.2687 6.0900 3

13 -2 -1 34.9617 5.2869 1

1 -13 -2 34.3698 6.0704 1

2 -1 -13 33.2124 6.1742 2

-2 -1 13 36.6437 5.4172 3

2 1 13 38.5364 5.9519 3

-13 2 -1 37.4202 7.0927 2

-1 -13 2 28.6730 6.0113 1

-2 1 -13 29.4762 6.7176 2

13 -3 1 166.374 11.142 1

1 13 -3 187.513 10.779 3

3 -1 13 151.698 11.829 3

-3 -1 -13 158.841 13.136 2

-13 -3 -1 165.770 12.521 1

-13 3 1 140.557 13.748 2

1 -13 3 164.894 12.710 1

-3 1 13 139.422 10.922 3

-1 -13 -3 183.402 12.456 1

3 1 -13 143.807 12.914 2

3 1 13 169.242 11.895 3

1 -13 -3 159.406 11.931 1

-3 -1 13 149.523 10.778 3

3 -1 -13 158.833 12.616 2

-3 1 -13 155.905 13.450 2

-13 -3 1 158.606 12.393 1

-1 -13 3 131.800 11.863 1

-13 3 -1 131.481 12.895 2

13 -3 -1 173.735 11.144 1

-1 -13 -4 239.607 15.882 1

1 -13 4 265.073 16.806 1

-4 -1 -13 279.392 18.090 2

13 -4 1 258.069 15.286 1

-13 -4 -1 259.280 17.129 1

4 1 -13 251.960 17.580 2

4 -1 13 234.568 16.037 3

1 13 -4 265.577 14.668 3

-13 4 1 210.161 17.850 2

-4 1 13 266.003 15.163 3

13 -4 -1 262.682 15.358 1

1 -13 -4 253.292 16.289 1

-4 -1 13 254.470 14.937 3

4 -1 -13 267.854 17.476 2

-13 4 -1 220.678 18.132 2

-1 -13 4 256.109 16.514 1

4 1 13 248.857 16.031 3

-4 1 -13 252.698 18.071 2

13 4 1 238.517 16.025 3

-13 -4 1 255.986 17.090 1

1 13 -5 83.0347 7.0228 3

-13 5 1 50.4611 9.3425 2

1 -13 5 55.0191 7.9574 1

5 -1 13 44.4508 7.6613 3

5 1 -13 64.6274 8.4292 2

13 5 -1 51.1716 6.9048 3

-13 -5 -1 61.6732 8.7441 1

13 -5 1 65.9530 7.1776 1

-1 -13 -5 55.6278 7.2551 1

-5 -1 -13 71.5627 8.7022 2

-5 1 -13 61.6238 9.0536 2

5 -1 -13 60.7369 8.3946 2

-5 -1 13 73.5690 7.1749 3

-13 5 -1 71.5630 10.1455 2

13 5 1 56.2877 7.5606 3

-1 -13 5 41.4889 7.7546 1

-13 -5 1 61.8041 8.8360 1

5 1 13 62.1360 7.9679 3

1 -13 -5 66.6065 7.8412 1

13 -5 -1 70.4126 7.2949 1

-1 -13 -6 34.9516 5.9974 1

-13 -6 -1 30.7924 7.5207 1

13 -6 1 40.6116 6.3154 1

6 1 -13 29.6811 6.8167 2

-6 -1 -13 36.2777 7.2024 2

13 6 -1 33.9627 6.0087 3

1 13 -6 43.2454 5.4346 3

6 -1 13 37.3512 7.2302 3

-13 6 1 10.7639 7.2699 2

1 -13 -6 32.6294 6.3246 1

-6 1 -13 38.1067 7.6227 2

6 1 13 28.9537 6.6195 3

13 6 1 30.4422 6.2789 3

-13 6 -1 19.5684 7.6877 2

13 -6 -1 31.4264 5.8235 1

6 -1 -13 45.5216 7.5639 2

-13 -6 1 23.6312 7.5250 1

7 1 -13 171.853 14.235 2

-13 7 1 171.922 16.184 2

-7 -1 -13 173.582 14.303 2

13 -7 1 178.381 12.814 1

-13 -7 -1 167.416 14.600 1

13 7 -1 158.275 12.515 3

7 -1 13 167.740 14.184 3

-1 -13 -7 162.939 12.775 1

1 13 -7 185.475 11.789 3

-13 -7 1 151.790 13.867 1

7 -1 -13 162.076 13.679 2

1 -13 -7 150.023 12.219 1

7 1 13 161.406 13.514 3

-13 7 -1 124.745 14.478 2

-7 1 -13 169.396 14.685 2

13 7 1 158.184 12.219 3

13 -7 -1 162.242 12.149 1

0 -2 13 0.48413 3.57166 3

0 -2 -13 -0.5471 3.6670 2

2 13 0 -0.0999 2.9031 3

2 -13 0 0.67182 4.29674 1

0 2 13 -6.8022 3.1201 3

0 2 -13 -3.7215 4.1296 2

13 0 2 -0.3614 3.2579 1

13 0 -2 -0.1127 3.4112 1

-2 -13 0 -10.894 3.565 1

-1 -2 -13 35.7497 6.4097 2

1 -2 13 28.7612 5.4260 3

13 -1 2 34.6278 5.2137 1

13 1 -2 38.0315 5.4555 1

1 2 -13 29.2597 6.6566 2

2 13 -1 48.4630 5.3823 3

2 -13 1 34.0274 6.2823 1

-1 2 13 34.3835 5.4533 3

-2 -13 -1 32.9559 5.9891 1

-1 2 -13 35.2199 7.1845 2

-1 -2 13 40.4645 5.8475 3

-2 -13 1 29.8195 6.1997 1

13 1 2 42.4746 5.5845 1

2 -13 -1 37.1567 6.4471 1

1 2 13 22.5899 5.2282 3

13 -1 -2 40.8074 5.4639 1

1 -2 -13 28.6663 5.9126 2

-2 2 13 4.92589 3.92572 3

-13 2 2 -3.6861 4.7406 2

-13 -2 -2 -2.3667 4.1130 1

2 13 -2 2.70444 3.17598 3

2 2 -13 1.78598 4.59242 2

-2 -13 -2 4.87714 4.37984 1

2 -13 2 -0.5412 4.3642 1

13 2 -2 3.42537 3.57975 1

-2 -2 -13 1.72230 4.30260 2

2 -2 13 2.15625 4.06384 3

13 -2 2 -1.0447 3.3672 1

-13 2 -2 1.91774 5.47033 2

-2 -13 2 1.30632 4.28163 1

2 -2 -13 -0.8923 3.6695 2

2 -13 -2 2.24232 4.27969 1

13 -2 -2 2.74917 3.56797 1

-2 -2 13 0.10936 3.44260 3

2 2 13 1.49119 3.87374 3

-2 2 -13 -2.6598 4.3300 2

2 13 -3 4.61223 3.35436 3

-3 -2 -13 0.26595 4.11406 2

3 -2 13 3.94950 4.56355 3

2 -13 3 -0.7287 4.7758 1

-2 -13 -3 2.68344 3.94765 1

-3 2 13 1.87878 3.74711 3

-13 3 2 2.35332 5.55723 2

13 -3 2 -0.0800 3.5764 1

-13 -3 -2 0.46159 4.63215 1

3 2 -13 -0.4926 4.3781 2

13 -3 -2 10.5359 3.9362 1

3 2 13 7.78322 4.54935 3

-13 3 -2 -5.2619 5.4019 2

-3 -2 13 0.94563 3.37933 3

3 -2 -13 4.00465 4.20188 2

-3 2 -13 7.13326 5.06736 2

-2 -13 3 2.12400 4.68875 1

2 -13 -3 4.10602 4.31133 1

-13 -3 2 3.49919 4.70876 1

4 2 -13 3.08497 4.83483 2

-13 4 2 -1.8685 5.6090 2

-2 -13 -4 -0.9911 4.1570 1

-13 -4 -2 -0.2242 5.0804 1

2 -13 4 -1.2854 4.3016 1

-4 -2 -13 -0.2208 4.5480 2

2 13 -4 2.31332 3.43627 3

4 -2 13 6.31626 4.69267 3

13 -4 2 -0.9018 3.7608 1

-4 -2 13 0.43071 3.53323 3

-13 4 -2 2.51542 5.91405 2

-2 -13 4 -1.0603 4.8782 1

2 -13 -4 -0.5078 4.0084 1

-4 2 -13 -0.0517 4.9366 2

-13 -4 2 3.68792 5.12616 1

13 4 2 0.10017 4.47601 3

4 -2 -13 1.94075 4.13332 2

4 2 13 -1.2936 4.1424 3

13 -4 -2 1.40100 3.66310 1

-2 -13 -5 93.2083 8.7741 1

5 -2 13 90.3833 9.5352 3

-13 5 2 66.6736 10.0420 2

13 5 -2 78.8291 8.1164 3

13 -5 2 83.3232 7.9894 1

2 13 -5 88.6750 7.7479 3

-13 -5 -2 88.4178 9.8948 1

2 -13 5 74.7926 8.8766 1

-5 -2 -13 89.9611 9.6006 2

5 2 -13 76.2077 9.1407 2

2 -13 -5 85.0573 8.9878 1

5 2 13 92.3755 9.3090 3

-2 -13 5 96.7819 9.4519 1

-5 -2 13 79.1431 7.6755 3

-13 5 -2 68.7400 10.4314 2

13 5 2 86.2835 8.9866 3

-5 2 -13 96.3130 10.8981 2

5 -2 -13 86.9179 9.5582 2

-13 -5 2 77.6502 9.8676 1

13 -5 -2 87.5823 8.2550 1

13 -6 2 5.18825 4.64184 1

6 -2 13 0.06324 5.27411 3

13 6 -2 -0.5705 4.0476 3

2 13 -6 2.67540 3.47962 3

-13 -6 -2 0.44948 5.73609 1

-6 -2 -13 1.84003 5.10729 2

-2 -13 -6 7.35666 4.67323 1

6 2 -13 8.35647 5.25011 2

-13 6 2 3.11436 6.40853 2

6 -2 -13 7.52926 5.30360 2

2 -13 -6 7.86159 4.70353 1

13 -6 -2 10.3104 4.8012 1

-6 -2 13 7.23687 4.04087 3

-13 -6 2 5.62592 6.18333 1

13 6 2 6.15834 4.71626 3

-6 2 -13 8.63769 5.84397 2

6 2 13 2.46890 5.07543 3

-13 6 -2 3.00478 6.63720 2

-13 -7 -2 11.5603 6.7509 1

13 -7 2 13.6340 5.3400 1

7 -2 13 0.39507 6.10702 3

-13 7 2 1.32651 6.86200 2

-2 -13 -7 3.16968 4.82825 1

13 7 -2 2.40168 4.44724 3

-7 -2 -13 3.97794 5.23992 2

2 13 -7 11.8746 4.2083 3

13 -7 -2 4.57148 4.78317 1

-13 -7 2 10.5734 6.7557 1

-13 7 -2 1.94274 6.84977 2

2 -13 -7 5.43401 5.18606 1

-7 2 -13 9.63098 6.33799 2

13 7 2 3.88224 4.80514 3

7 -2 -13 3.24884 5.34655 2

3 -13 0 220.055 15.561 1

0 -3 13 248.063 15.221 3

0 3 13 231.753 14.816 3

13 0 -3 226.595 14.259 1

3 13 0 260.793 14.020 3

0 3 -13 266.194 18.209 2

0 -3 -13 218.075 15.924 2

13 0 3 250.091 14.416 1

-3 -13 0 247.840 15.586 1

13 -1 3 188.123 11.238 1

-13 -1 -3 131.226 11.303 1

1 3 -13 151.492 13.565 2

1 -3 13 138.231 11.524 3

3 13 -1 179.433 10.728 3

-1 -3 -13 152.861 12.395 2

-3 -13 -1 161.836 12.048 1

-1 3 13 157.575 11.397 3

13 1 -3 160.421 11.002 1

3 -13 1 171.472 12.722 1

13 -1 -3 149.889 11.025 1

-1 3 -13 182.075 14.681 2

3 13 1 175.941 10.808 3

3 -13 -1 164.117 12.630 1

1 3 13 172.208 11.936 3

1 -3 -13 158.163 12.488 2

-3 -13 1 157.760 12.245 1

-1 -3 13 167.284 11.791 3

-13 -2 -3 3.96216 4.71251 1

-2 -3 -13 -2.9855 3.8493 2

-2 3 13 3.86146 3.96404 3

3 -13 2 6.36387 4.76420 1

13 2 -3 4.23854 3.74406 1

2 -3 13 -2.0989 4.1082 3

3 13 -2 -1.2389 3.0812 3

-3 -13 -2 3.51339 4.29481 1

2 3 -13 4.17481 5.04436 2

13 -2 3 -3.1797 3.4353 1

-2 -3 13 1.88253 3.76891 3

2 3 13 1.84892 3.93420 3

2 -3 -13 0.12708 3.69355 2

3 13 2 6.47294 3.41200 3

-2 3 -13 3.37950 4.88190 2

13 -2 -3 -0.5186 3.5091 1

-3 -13 2 3.62606 4.27258 1

3 -13 -2 5.26149 4.66360 1

-13 2 -3 -1.2600 5.1923 2

-13 -3 -3 195.719 14.503 1

-13 3 3 162.304 15.683 2

3 -3 13 201.145 14.518 3

3 -13 3 215.730 14.717 1

-3 -13 -3 217.157 14.369 1

3 13 -3 242.881 13.148 3

-3 -3 -13 225.607 15.334 2

3 3 -13 190.458 15.526 2

13 -3 3 220.923 13.203 1

13 -3 -3 212.034 13.296 1

3 3 13 191.680 13.778 3

-13 3 -3 196.074 15.983 2

-3 -3 13 225.386 13.737 3

-13 -3 3 196.884 14.635 1

-3 3 -13 212.918 16.720 2

3 -3 -13 183.694 14.562 2

3 13 3 233.100 12.855 3

-3 -13 3 225.486 14.565 1

3 -13 -3 192.365 14.398 1

13 3 3 215.218 14.971 3

-3 -13 -4 236.272 14.785 1

4 -3 13 199.208 14.864 3

-13 -4 -3 221.092 15.514 1

-4 -3 -13 203.571 15.453 2

13 -4 3 238.932 14.040 1

3 -13 4 226.985 15.447 1

3 13 -4 232.695 13.307 3

4 3 -13 198.557 15.948 2

-13 4 3 191.543 16.581 2

-4 -3 13 216.083 13.640 3

13 4 3 203.890 14.967 3

-4 3 -13 232.286 17.360 2

-13 4 -3 230.586 17.506 2

4 -3 -13 194.454 15.024 2

-3 -13 4 214.113 14.800 1

3 -13 -4 195.148 14.539 1

13 -4 -3 220.147 13.725 1

-13 -4 3 202.615 15.310 1

4 3 13 224.083 14.758 3

3 13 -5 133.341 9.524 3

13 -5 3 137.296 10.148 1

-13 5 3 103.212 12.444 2

-3 -13 -5 160.704 11.373 1

-5 -3 -13 118.733 11.279 2

-13 -5 -3 124.605 11.544 1

5 3 -13 107.220 11.054 2

5 -3 13 103.174 10.738 3

-5 -3 13 141.908 10.379 3

-13 -5 3 141.569 12.742 1

-3 -13 5 130.291 11.841 1

-5 3 -13 151.512 13.641 2

-13 5 -3 116.656 13.243 2

13 5 3 136.828 11.655 3

5 -3 -13 137.742 12.187 2

3 -13 -5 130.788 11.351 1

13 -5 -3 133.517 10.495 1

5 3 13 143.046 11.893 3

3 13 -6 9.23698 3.86037 3

6 -3 13 9.46047 5.89565 3

-3 -13 -6 8.89799 5.05078 1

13 -6 3 2.24001 4.34317 1

-6 -3 -13 6.64890 5.51004 2

-13 6 3 5.04661 6.94505 2

13 6 -3 4.23629 4.39429 3

-13 -6 -3 14.7872 6.2403 1

6 -3 -13 8.40201 5.39913 2

13 6 3 3.93631 4.92982 3

3 -13 -6 2.46883 4.85621 1

-13 6 -3 -2.5286 6.6183 2

13 -6 -3 4.23061 4.55570 1

-13 -6 3 8.40289 6.66243 1

6 3 13 9.17035 5.88204 3

-3 -13 6 9.23641 5.90170 1

-6 3 -13 7.08653 6.11544 2

-6 -3 13 3.08960 3.78354 3

-4 -13 0 45.9349 6.5543 1

13 0 -4 23.2898 5.0721 1

0 4 -13 35.2571 7.7011 2

0 -4 13 35.3994 5.9545 3

0 4 13 35.2277 5.8740 3

4 13 0 47.0697 5.3904 3

4 -13 0 35.3628 6.7338 1

0 -4 -13 31.4585 5.9148 2

4 13 -1 247.774 14.191 3

1 -4 13 229.163 15.583 3

-13 -1 -4 228.169 15.238 1

-1 4 13 257.873 15.374 3

-1 -4 -13 253.768 16.496 2

1 4 -13 235.803 17.982 2

-4 -13 -1 245.902 15.657 1

4 -13 1 278.744 16.824 1

13 1 -4 247.217 14.848 1

4 13 1 252.222 14.468 3

-1 4 -13 238.657 18.722 2

13 -1 -4 235.851 15.009 1

1 -4 -13 247.284 16.634 2

-4 -13 1 265.471 16.402 1

-1 -4 13 261.958 15.972 3

1 4 13 246.910 15.669 3

4 -13 -1 290.349 17.224 1

4 13 -2 4.74793 3.42588 3

-4 -13 -2 3.87528 4.31271 1

4 -13 2 3.84519 4.74996 1

2 -4 13 -3.6157 4.0458 3

-2 -4 -13 0.87488 3.93757 2

2 4 -13 -1.8069 4.8766 2

-13 -2 -4 0.87866 4.64326 1

13 2 -4 1.28710 3.53768 1

13 -2 -4 -0.9484 3.6143 1

2 4 13 3.34820 4.13736 3

-13 2 -4 1.93656 4.93581 2

-4 -13 2 1.75526 4.47685 1

2 -4 -13 2.13737 3.77715 2

-2 4 -13 1.83702 5.26720 2

4 -13 -2 -1.1813 4.1085 1

4 13 2 2.46843 3.25054 3

-2 -4 13 3.36686 4.07809 3

-4 -13 -3 240.469 14.817 1

4 -13 3 218.779 15.022 1

-13 3 4 185.339 16.243 2

4 13 -3 228.684 13.022 3

3 4 -13 208.000 16.152 2

3 -4 13 187.515 14.512 3

-13 -3 -4 195.320 14.570 1

-3 -4 -13 215.703 15.058 2

4 13 3 235.072 13.432 3

3 4 13 214.018 14.687 3

-3 4 -13 201.573 17.111 2

13 3 4 234.708 16.265 3

-4 -13 3 251.064 15.601 1

4 -13 -3 180.794 14.637 1

-13 3 -4 224.350 16.879 2

3 -4 -13 219.143 15.323 2

-3 -4 13 228.212 14.297 3

-13 -3 4 224.183 15.824 1

13 -3 -4 210.903 13.767 1

4 -4 13 51.0096 7.8725 3

-4 -13 -4 58.1494 7.2895 1

4 -13 4 45.6382 7.3901 1

-13 4 4 47.5873 8.9760 2

-13 -4 -4 52.6066 8.0437 1

4 4 -13 51.8655 8.1351 2

4 13 -4 69.4840 6.5008 3

-4 -4 -13 51.0046 7.5351 2

4 13 4 79.5339 7.0024 3

-4 -13 4 51.4914 7.5856 1

-13 -4 4 61.4842 8.9074 1

-13 4 -4 49.3250 9.0078 2

13 4 4 52.9783 8.2267 3

4 -13 -4 60.0131 7.9790 1

4 -4 -13 67.8552 8.0043 2

13 -4 -4 48.7862 6.4578 1

-4 4 -13 49.2501 9.0817 2

4 4 13 64.9650 8.0786 3

-4 -4 13 54.2994 6.7959 3

-4 -13 -5 266.047 15.975 1

-13 -5 -4 221.859 16.220 1

5 -4 13 242.668 16.679 3

-13 5 4 196.957 17.802 2

-5 -4 -13 236.926 16.413 2

4 13 -5 245.408 14.123 3

-5 -4 13 221.348 14.256 3

5 4 13 224.871 15.860 3

13 5 4 262.014 16.414 3

-5 4 -13 213.218 17.906 2

5 -4 -13 209.905 15.909 2

-4 -13 5 199.799 15.022 1

4 13 5 238.855 13.798 3

-13 -5 4 239.593 17.203 1

13 -5 -4 227.865 14.448 1

4 -13 -5 225.271 15.684 1

-13 5 -4 212.934 17.885 2

-13 -6 -4 8.33773 6.22049 1

4 13 -6 4.57643 3.65690 3

-4 -13 -6 3.12406 4.65537 1

-6 -4 -13 4.18081 5.02852 2

13 6 -4 6.66756 4.45335 3

6 -4 13 -1.2515 5.4922 3

-13 6 4 9.16516 7.41541 2

13 6 4 -0.1603 4.9274 3

-6 4 -13 -2.1404 5.7845 2

-6 -4 13 7.24718 4.33302 3

6 4 13 4.86101 5.70317 3

-13 -6 4 8.12798 6.60715 1

-4 -13 6 4.03563 5.50758 1

6 -4 -13 4.87953 5.12680 2

-13 6 -4 -7.2449 6.2409 2

4 -13 -6 6.27038 5.02673 1

13 -6 -4 2.18468 4.11930 1

-5 -13 0 234.368 15.818 1

13 0 -5 254.515 15.365 1

5 13 0 251.527 14.707 3

5 -13 0 271.537 17.055 1

0 5 13 252.684 15.852 3

-5 13 0 267.368 17.608 2

0 -5 13 247.715 16.252 3

0 5 -13 240.282 18.751 2

0 -5 -13 271.147 16.859 2

1 -5 13 73.4007 8.3245 3

13 1 -5 60.3256 6.9543 1

5 13 -1 53.0452 6.0656 3

-1 -5 -13 75.7756 8.0553 2

1 5 -13 60.4523 9.2145 2

-5 13 1 68.2532 8.5292 2

5 -13 1 67.4688 8.3358 1

-5 -13 -1 68.9249 8.0102 1

1 5 13 60.8223 7.4723 3

1 -5 -13 58.6216 7.4856 2

13 -1 -5 69.0192 7.0573 1

5 -13 -1 65.4101 8.3956 1

-1 -5 13 70.5958 7.9477 3

5 13 1 67.6598 6.6457 3

-5 13 -1 59.6304 8.6188 2

-5 -13 1 74.9120 8.2452 1

-1 5 -13 51.9214 9.0198 2

13 2 -5 82.1169 7.9340 1

-5 -13 -2 97.4014 9.0420 1

-2 -5 -13 82.8537 8.5849 2

2 5 -13 82.4122 10.1930 2

5 -13 2 90.3834 9.3323 1

-5 13 2 83.8436 9.3247 2

2 -5 13 71.6096 8.7517 3

5 13 -2 75.1924 6.9882 3

2 5 13 84.2849 8.7904 3

5 13 2 84.2974 7.4441 3

13 -2 -5 71.2634 7.5611 1

-13 2 -5 71.9188 9.4089 2

5 -13 -2 90.8102 9.3383 1

-5 -13 2 89.3089 9.0836 1

-5 13 -2 88.6167 9.8032 2

-2 -5 13 94.7854 8.8727 3

-2 5 -13 75.5550 10.4534 2

2 -5 -13 85.6418 8.7785 2

3 -5 13 138.904 12.035 3

5 13 -3 157.723 10.209 3

-5 13 3 108.980 11.214 2

-3 -5 -13 154.453 11.891 2

-5 -13 -3 141.406 11.308 1

5 -13 3 164.832 12.509 1

-13 3 5 108.480 12.782 2

3 5 -13 127.132 12.629 2

-13 -3 5 134.883 12.152 1

-5 -13 3 114.414 10.949 1

-13 3 -5 147.599 13.518 2

3 -5 -13 135.616 11.339 2

13 3 5 127.987 12.109 3

-3 5 -13 135.341 13.578 2

5 13 3 141.323 9.936 3

3 5 13 140.059 11.631 3

5 -13 -3 130.680 11.477 1

13 -3 -5 130.081 10.317 1

-3 -5 13 135.842 10.947 3

5 13 -4 262.428 14.614 3

4 -5 13 219.881 16.695 3

-5 13 4 234.985 17.157 2

-4 -5 -13 269.008 16.867 2

5 -13 4 236.168 16.131 1

-13 4 5 206.201 18.236 2

-5 -13 -4 253.662 15.957 1

4 -5 -13 246.623 16.471 2

-4 5 -13 236.841 18.790 2

-13 4 -5 221.907 18.044 2

-4 -5 13 234.520 15.129 3

-5 -13 4 228.042 15.860 1

5 13 4 222.202 14.104 3

13 4 5 245.373 16.888 3

13 -4 -5 223.558 14.701 1

5 -13 -4 237.326 16.239 1

4 5 13 273.579 16.692 3

-13 -4 5 226.888 16.844 1

-5 13 5 31.5722 7.1589 2

-5 -5 -13 35.0198 6.5541 2

-5 -13 -5 37.6052 6.5202 1

5 13 -5 27.5852 4.8727 3

-13 5 5 24.5296 8.6475 2

5 -5 13 22.1448 7.1444 3

13 5 5 39.2761 7.6044 3

-5 5 -13 29.1543 8.6075 2

-5 -5 13 38.1403 6.2542 3

5 -13 -5 35.5812 7.0929 1

5 5 13 41.2346 7.3966 3

-5 -13 5 24.6195 6.6326 1

-13 -5 5 14.9015 6.9914 1

13 -5 -5 24.0096 5.4177 1

-13 5 -5 31.5546 8.4497 2

5 -5 -13 25.2657 5.9564 2

5 13 5 35.4980 5.5103 3

-6 -13 0 -1.9905 4.3820 1

0 6 -13 1.46481 5.64069 2

0 -6 13 2.51924 4.48141 3

13 0 -6 -1.3296 3.5259 1

0 -6 -13 4.16768 4.19247 2

-6 13 0 3.57796 5.24064 2

6 -13 0 -2.7923 4.5702 1

6 13 0 2.00188 3.22301 3

-6 13 1 37.6605 7.4251 2

6 13 -1 43.0293 5.3850 3

6 -13 1 27.5797 6.6732 1

-6 -13 -1 39.7207 6.9056 1

1 6 -13 33.7381 7.7999 2

1 -6 13 37.0201 6.8941 3

13 1 -6 35.7679 5.6370 1

-1 -6 -13 34.9152 6.0512 2

13 -1 -6 22.8131 5.0921 1

6 -13 -1 35.4116 6.9852 1

-6 -13 1 37.6132 6.7909 1

1 6 13 31.9647 6.4267 3

6 13 1 22.4772 4.5687 3

-6 13 -1 37.2688 7.5274 2

-1 6 -13 30.1901 7.9318 2

1 -6 -13 38.2359 6.1782 2

-1 -6 13 23.4322 6.0501 3

-2 -6 -13 11.7308 4.5999 2

6 13 -2 6.74610 3.64337 3

-6 13 2 4.15021 5.24849 2

2 -6 13 -2.3915 4.7166 3

-6 -13 -2 3.02807 4.80752 1

6 -13 2 -1.1673 4.8631 1

-6 -13 2 9.92560 5.72223 1

13 -2 -6 4.72257 4.02176 1

2 6 13 3.39141 5.00570 3

-2 -6 13 8.94774 5.07624 3

2 -6 -13 9.37674 4.74812 2

-2 6 -13 10.6621 6.8072 2

-13 2 -6 4.72542 5.61891 2

6 -13 -2 4.94727 5.25216 1

-13 -2 6 8.00371 5.62127 1

-6 13 -2 6.70073 5.67640 2

6 13 2 7.50302 3.69500 3

6 -13 3 5.44370 5.41885 1

-3 -6 -13 4.63283 4.64289 2

-13 3 6 12.2169 7.4135 2

-6 -13 -3 1.66965 4.87975 1

6 13 -3 12.0891 4.0470 3

3 -6 13 6.69721 5.62448 3

-6 13 3 6.60449 5.54381 2

3 -6 -13 11.0941 5.1624 2

-6 -13 3 14.0587 6.0938 1

-3 -6 13 6.64858 4.95344 3

-13 -3 6 11.9491 6.2754 1

6 13 3 11.8999 4.0480 3

13 -3 -6 5.71185 4.47187 1

6 -13 -3 9.20754 5.35046 1

-6 13 -3 10.5327 6.4892 2

-3 6 -13 16.6271 7.4213 2

13 3 6 15.1227 6.7325 3

3 6 13 1.67959 5.04861 3

-13 3 -6 1.85126 6.33853 2

-13 4 6 -0.5912 7.5007 2

-4 -6 -13 3.90289 4.77494 2

4 -6 13 2.24972 5.67993 3

-6 13 4 0.54794 5.31659 2

-6 -13 -4 7.21647 5.19542 1

6 13 -4 4.64879 3.83704 3

-13 -4 6 4.22822 5.94541 1

-4 6 -13 3.10453 6.65437 2

-4 -6 13 2.45667 4.81737 3

6 13 4 1.45429 3.70805 3

13 4 6 1.43037 5.79363 3

6 -13 -4 2.12122 4.98231 1

4 -6 -13 5.36685 4.39765 2

13 -4 -6 2.81263 4.10016 1

-13 4 -6 2.88048 6.41745 2

-6 -13 4 -0.2989 5.2610 1

4 6 13 3.12086 5.22552 3

-6 13 -4 5.16178 6.47139 2

0 7 -13 93.3745 12.2368 2

-7 -13 0 158.962 12.457 1

-7 13 0 129.216 12.241 2

13 0 -7 109.565 9.838 1

0 -7 -13 159.696 11.596 2

7 -13 0 140.293 11.893 1

7 13 0 119.065 9.421 3

0 -7 13 123.672 11.555 3

7 13 -1 170.007 11.239 3

-7 13 1 165.121 14.164 2

1 -7 13 160.470 13.670 3

13 1 -7 148.220 11.590 1

7 -13 1 170.104 13.280 1

-7 -13 -1 167.056 13.384 1

-1 -7 -13 186.412 13.172 2

1 -7 -13 168.684 12.767 2

-7 13 -1 167.685 14.271 2

7 -13 -1 162.599 13.281 1

-7 -13 1 168.012 13.276 1

-1 -7 13 166.116 12.979 3

7 13 1 154.745 10.888 3

13 -1 -7 158.048 11.520 1

-1 7 -13 122.634 14.256 2

-7 -13 -2 10.9527 5.8279 1

2 -7 13 -0.1035 5.1563 3

7 -13 2 3.63080 5.58787 1

-7 13 2 2.81680 5.63803 2

-2 -7 -13 3.18530 4.72712 2

7 13 -2 6.93269 3.76643 3

-7 13 -2 6.66130 5.96292 2

-13 -2 7 2.77536 5.54727 1

7 13 2 4.77954 3.99329 3

13 2 7 -0.7383 6.1947 3

-2 -7 13 6.71445 5.05778 3

-13 2 -7 9.57626 6.53307 2

2 -7 -13 8.10948 4.85047 2

13 -2 -7 3.73956 4.24574 1

-2 7 -13 0.99475 6.45563 2

2 7 13 4.48325 5.29104 3

-7 -13 2 12.8164 6.0336 1

0 0 -14 1.22193 4.50245 2

14 0 0 2.11911 3.87864 1

0 -14 0 2.23640 4.53969 1

0 0 14 0.00869 3.75458 3

-1 0 -14 44.8617 7.6465 2

0 -14 -1 65.5515 7.6502 1

14 -1 0 52.8580 6.3295 1

1 0 14 55.8722 7.0839 3

-1 0 14 46.0592 6.5723 3

1 0 -14 39.7420 7.2409 2

14 1 0 57.9265 6.5862 1

0 -14 1 51.4391 7.2533 1

-2 0 -14 11.0348 5.5099 2

0 -14 -2 6.30457 4.66053 1

2 0 -14 5.56373 5.02757 2

14 -2 0 3.76955 4.04809 1

-14 -2 0 10.1932 5.4901 1

-2 0 14 3.83878 4.13036 3

2 0 14 1.24340 4.39206 3

0 -14 2 4.37508 5.22975 1

3 0 14 87.3780 8.8826 3

0 -14 3 76.8607 8.7382 1

-14 -3 0 76.3308 9.1399 1

-3 0 14 80.6601 8.1039 3

14 -3 0 85.8508 8.0090 1

0 -14 -3 87.9464 8.7160 1

-3 0 -14 80.5974 9.7084 2

3 0 -14 67.6640 8.8449 2

-4 0 14 2.03219 4.15691 3

4 0 14 -0.0857 4.6119 3

0 -14 4 0.76172 5.07985 1

0 -14 -4 -1.8004 3.9888 1

-4 0 -14 -0.9750 4.9970 2

4 0 -14 2.23892 4.70576 2

14 -4 0 2.31986 4.22559 1

-14 4 0 -0.9508 6.6243 2

-14 -4 0 -3.1289 5.4925 1

5 0 14 21.4802 6.4410 3

-5 0 -14 24.0697 6.8773 2

-14 5 0 19.9631 8.1499 2

0 -14 -5 24.5347 5.7190 1

5 0 -14 25.6665 6.7203 2

-14 -5 0 18.2174 7.1220 1

14 -5 0 30.0532 5.8629 1

0 -14 5 19.1739 6.6014 1

0 -1 -14 43.2475 7.2199 2

0 1 -14 47.6062 7.7792 2

0 -1 14 45.7303 6.7404 3

0 1 14 51.2614 6.7160 3

14 0 1 65.1908 6.6981 1

1 -14 0 49.9875 7.1877 1

-1 -14 0 63.0717 7.5741 1

14 0 -1 45.9755 6.1933 1

14 1 -1 7.26897 3.96948 1

14 -1 1 5.83583 3.97805 1

1 -1 14 2.26739 4.36708 3

-1 1 14 -1.9513 3.6920 3

-1 -1 -14 2.14826 4.44147 2

1 1 -14 -0.2281 4.4153 2

-1 -14 -1 6.92488 4.31705 1

1 -14 1 8.57975 5.26950 1

1 -14 -1 0.17160 4.44548 1

-1 -1 14 9.91994 4.43998 3

1 -1 -14 3.28883 4.81815 2

14 -1 -1 0.96421 3.76743 1

-1 1 -14 5.06434 5.21914 2

-1 -14 1 2.41073 4.43079 1

14 1 1 0.21510 3.80862 1

1 1 14 -2.5913 4.0529 3

-1 -14 -2 197.150 13.809 1

-2 -1 -14 189.133 14.868 2

2 1 -14 177.493 14.846 2

14 -2 1 211.468 13.057 1

-14 -2 -1 206.281 14.342 1

2 -1 14 194.949 13.723 3

1 -14 2 178.263 14.124 1

-2 1 14 186.309 13.091 3

-1 -14 2 188.596 14.137 1

-14 -2 1 214.518 14.645 1

-2 1 -14 181.312 15.336 2

2 -1 -14 182.642 14.684 2

14 -2 -1 234.244 13.448 1

-2 -1 14 176.699 12.862 3

2 1 14 186.682 13.592 3

1 -14 -2 210.177 14.180 1

-1 -14 -3 3.81173 4.48624 1

14 -3 1 1.84321 4.10291 1

1 -14 3 1.64198 5.02774 1

-14 -3 -1 6.31072 5.53109 1

3 1 -14 3.86505 4.78781 2

-3 -1 -14 2.19868 5.10441 2

-3 1 14 -4.6296 3.9907 3

3 -1 14 0.48948 4.50551 3

3 -1 -14 3.40340 4.54442 2

1 -14 -3 2.75094 4.61062 1

3 1 14 0.29668 4.70110 3

-3 1 -14 6.15962 5.34259 2

14 -3 -1 3.84329 4.30108 1

-3 -1 14 0.20365 3.79932 3

-14 -3 1 5.66915 5.24699 1

-1 -14 3 -1.0842 4.8706 1

-14 3 -1 -2.3258 6.0146 2

-14 4 1 37.9642 8.9840 2

4 -1 14 41.5199 7.4102 3

-14 -4 -1 44.5293 8.2149 1

-4 -1 -14 59.9424 8.6222 2

14 -4 1 63.7651 7.3272 1

-1 -14 -4 63.9407 7.6191 1

1 -14 4 44.7504 7.6035 1

4 1 -14 62.4532 8.4856 2

-4 -1 14 59.3135 7.1631 3

14 -4 -1 52.5951 6.9039 1

4 1 14 53.5725 7.7649 3

1 -14 -4 63.3568 7.7208 1

-4 1 -14 48.6074 8.4226 2

4 -1 -14 45.0126 7.7754 2

-14 -4 1 60.0697 8.6505 1

-14 4 -1 49.9233 9.2953 2

-1 -14 4 65.5350 8.2794 1

14 -5 1 14.3422 5.1495 1

-1 -14 -5 17.2166 5.1461 1

-5 -1 -14 19.2403 6.5857 2

-14 5 1 12.3268 7.5153 2

5 1 -14 18.3843 6.4467 2

-14 -5 -1 7.78413 6.14099 1

-14 -5 1 16.2677 7.2103 1

5 -1 -14 16.0129 5.9167 2

-5 -1 14 12.1038 4.9032 3

5 1 14 20.7413 6.3475 3

-5 1 -14 22.7796 7.0888 2

1 -14 -5 14.2663 5.3523 1

-14 5 -1 17.0203 7.5482 2

-1 -14 5 13.4810 6.0876 1

14 -5 -1 9.98390 4.92411 1

0 2 -14 0.27934 4.74655 2

14 0 -2 6.96432 4.32278 1

14 0 2 7.76584 4.26254 1

0 2 14 7.80507 4.74706 3

2 -14 0 8.62808 4.96344 1

-2 -14 0 3.34265 4.48029 1

0 -2 -14 9.45577 4.86624 2

0 -2 14 11.0081 4.9027 3

-2 -14 -1 175.535 13.160 1

1 2 -14 169.913 14.999 2

-14 -1 -2 196.734 13.816 1

1 -2 14 181.607 13.604 3

14 1 -2 204.330 12.966 1

2 -14 1 195.634 14.219 1

-1 2 14 176.133 12.870 3

-1 -2 -14 191.759 14.479 2

14 -1 2 209.560 12.748 1

-2 -14 1 188.644 13.688 1

-1 -2 14 178.895 13.027 3

-1 2 -14 180.437 15.358 2

1 2 14 185.753 13.115 3

14 -1 -2 189.065 12.755 1

2 -14 -1 209.920 14.273 1

1 -2 -14 202.357 14.635 2

2 -14 2 442.583 25.433 1

-2 -2 -14 454.108 26.697 2

14 -2 2 461.496 23.920 1

2 2 -14 437.247 27.326 2

2 -2 14 455.217 25.189 3

-2 2 14 422.528 23.834 3

-2 -14 -2 495.209 25.170 1

-14 -2 -2 441.440 25.195 1

-2 -2 14 467.339 24.344 3

2 -14 -2 443.805 25.166 1

2 2 14 444.324 24.480 3

-2 -14 2 465.999 25.307 1

2 -2 -14 442.176 26.404 2

-14 -2 2 454.785 25.459 1

14 -2 -2 468.004 24.140 1

-2 2 -14 409.452 27.482 2

-3 -2 -14 233.940 17.091 2

3 -2 14 233.268 16.178 3

14 -3 2 248.681 15.006 1

-14 -3 -2 236.604 16.558 1

3 2 -14 245.543 17.846 2

2 -14 3 265.026 16.847 1

3 -2 -14 237.970 16.581 2

-2 -14 3 241.935 15.965 1

14 -3 -2 246.320 15.120 1

-14 3 -2 222.480 17.749 2

-14 -3 2 247.557 16.551 1

-3 2 -14 259.341 18.475 2

2 -14 -3 249.455 15.877 1

-3 -2 14 215.299 14.633 3

3 2 14 205.446 15.201 3

2 -14 4 4.19800 5.52396 1

-14 -4 -2 8.21468 6.05437 1

-4 -2 -14 5.76173 5.32628 2

14 -4 2 8.22045 4.61674 1

-2 -14 -4 0.19001 4.31539 1

4 -2 14 5.51064 5.25245 3

-14 4 2 0.48882 6.69426 2

4 2 -14 5.96326 5.60617 2

14 -4 -2 3.47242 4.37408 1

-4 -2 14 2.40167 4.04532 3

2 -14 -4 3.57264 4.66601 1

-2 -14 4 7.16697 5.22328 1

-14 -4 2 6.42894 5.65598 1

-14 4 -2 -1.9709 6.4614 2

-4 2 -14 -0.0561 5.5941 2

4 -2 -14 0.32937 4.51660 2

4 2 14 -1.8519 4.8085 3

0 -3 -14 81.2028 8.8213 2

-3 -14 0 65.9946 7.9678 1

14 0 3 90.0547 7.9875 1

0 3 14 69.5516 7.8947 3

0 -3 14 73.3383 8.4035 3

14 0 -3 85.3395 7.8243 1

0 3 -14 73.3895 9.9602 2

3 -14 0 79.7618 9.1185 1

-14 -1 -3 1.77628 4.96301 1

3 -14 1 4.48243 5.07468 1

14 1 -3 -4.6007 3.6172 1

1 -3 14 0.31565 4.57372 3

1 3 -14 1.09585 5.16272 2

3 14 -1 5.16109 3.79357 3

14 -1 3 3.31695 4.14779 1

-3 -14 -1 0.99119 4.37155 1

-1 -3 -14 -0.6494 4.2713 2

-1 3 14 1.10189 4.25791 3

1 3 14 0.94678 4.27443 3

-3 -14 1 2.90920 4.76812 1

14 -1 -3 -1.0224 3.7300 1

-1 3 -14 2.67582 5.56781 2

1 -3 -14 2.19434 4.34220 2

-1 -3 14 -0.6456 4.2047 3

3 -14 -1 -2.6832 4.6286 1

3 14 -2 275.141 14.372 3

2 3 -14 225.331 17.793 2

-2 -3 -14 239.487 16.603 2

14 -2 3 243.450 14.725 1

3 -14 2 240.299 16.456 1

2 -3 14 240.139 16.213 3

-14 -2 -3 225.283 15.927 1

-3 -14 -2 209.519 15.388 1

-2 3 -14 230.427 17.949 2

-3 -14 2 212.801 15.477 1

14 -2 -3 240.200 14.710 1

2 3 14 238.468 15.418 3

-2 -3 14 228.843 15.050 3

2 -3 -14 240.837 16.333 2

3 -14 -2 238.937 16.145 1

-14 -3 -3 9.09897 5.91588 1

3 3 -14 3.65214 5.61472 2

3 -14 3 11.8583 5.8203 1

-3 -3 -14 6.51910 5.24294 2

3 14 -3 11.5861 4.0914 3

3 -3 14 8.81604 5.43282 3

14 -3 3 14.3927 4.8937 1

-3 -14 -3 16.1633 5.3182 1

-14 -3 3 11.8657 5.8525 1

3 3 14 3.04466 4.74917 3

-14 3 -3 4.88022 6.34961 2

-3 -14 3 8.99190 5.53382 1

3 -14 -3 11.0121 5.3151 1

-3 -3 14 15.2132 5.1441 3

3 -3 -14 7.97453 4.70048 2

-3 3 -14 9.87195 6.02197 2

14 -3 -3 12.1960 4.7946 1

-14 4 3 37.6475 8.9560 2

3 14 -4 58.7032 6.3441 3

3 -14 4 52.2300 8.1749 1

4 -3 14 62.8657 8.6884 3

-14 -4 -3 65.4676 8.8936 1

-3 -14 -4 49.8696 7.3268 1

4 3 -14 47.7438 8.4467 2

-4 -3 -14 59.2989 8.0270 2

3 -14 -4 45.5070 7.5417 1

-3 -14 4 71.5924 8.2182 1

-14 -4 3 31.8099 7.8608 1

14 -4 -3 66.4339 7.2750 1

4 3 14 66.2161 8.3196 3

-4 -3 14 64.8738 7.4947 3

-4 3 -14 43.2671 8.7690 2

4 -3 -14 59.1767 8.1786 2

-14 4 -3 53.1301 9.7291 2

4 14 0 3.31269 3.37097 3

4 -14 0 -4.6073 4.7571 1

0 -4 -14 -0.8730 4.1243 2

14 0 -4 -2.5813 3.4045 1

0 -4 14 1.41043 4.17446 3

-4 -14 0 -1.8146 4.3466 1

0 4 -14 5.96504 5.78651 2

0 4 14 -1.0462 4.2347 3

-4 -14 -1 47.9828 7.3011 1

-1 -4 -14 63.7320 7.8810 2

4 14 -1 50.9311 5.9965 3

4 -14 1 67.8044 8.5539 1

1 -4 14 61.3995 7.8877 3

1 4 -14 50.6490 8.9882 2

14 1 -4 66.6271 7.0137 1

-1 4 -14 55.7238 9.3764 2

14 -1 -4 58.4638 6.7832 1

-4 -14 1 51.8774 7.4424 1

4 -14 -1 63.4406 8.1903 1

4 14 1 60.7947 6.1378 3

-1 -4 14 45.0754 7.1818 3

1 -4 -14 52.5332 7.4470 2

1 4 14 66.4500 7.7247 3

2 -4 14 0.20504 4.50747 3

4 14 -2 2.54119 3.65096 3

-4 -14 -2 3.77017 4.67496 1

2 4 -14 -2.7877 5.1412 2

4 -14 2 -0.1237 4.7346 1

-2 -4 -14 11.2052 5.0537 2

14 -2 -4 6.53602 4.20281 1

-4 -14 2 4.20865 4.87196 1

2 -4 -14 0.42852 4.41853 2

2 4 14 0.61671 4.57110 3

-14 -2 4 4.75279 5.36064 1

-2 -4 14 -2.6904 4.1656 3

-2 4 -14 6.76307 6.19628 2

4 14 2 4.16706 3.55682 3

4 -14 -2 8.71383 5.20210 1

-3 -4 -14 70.0770 8.3435 2

4 -14 3 64.1698 8.5357 1

-4 -14 -3 59.9040 7.8100 1

4 14 -3 61.6235 6.4094 3

3 -4 14 49.0424 8.2093 3

3 4 -14 58.5201 9.0456 2

3 -4 -14 64.5088 7.9585 2

-4 -14 3 59.8345 7.8741 1

-3 4 -14 56.7996 9.6673 2

-14 -3 4 57.0543 8.6883 1

-3 -4 14 62.3940 7.6667 3

14 -3 -4 52.5542 6.8437 1

3 4 14 57.8367 7.8593 3

-14 3 -4 48.9552 8.7500 2

4 -14 -3 59.0924 8.0806 1

14 0 -5 25.3360 5.4173 1

5 14 0 32.0857 4.9239 3

0 5 -14 22.3549 7.4289 2

-5 -14 0 17.6844 5.4925 1

0 -5 -14 30.7756 5.9954 2

0 -5 14 11.1748 5.2446 3

5 -14 0 10.1931 6.0764 1

5 14 -1 17.9301 4.4171 3

-1 -5 -14 23.0985 5.6538 2

14 1 -5 21.1718 5.2112 1

-5 -14 -1 18.0325 5.6650 1

5 -14 1 8.34288 6.05036 1

1 5 -14 18.8503 7.0840 2

-5 -14 1 19.2659 5.7268 1

5 14 1 16.2704 4.4437 3

-1 5 -14 13.1360 7.2378 2

1 -5 -14 20.6101 5.6920 2

5 -14 -1 14.4041 6.0654 1

-1 -5 14 17.4259 5.6478 3

1 5 14 13.5142 5.7299 3

0 0 0 0.00 0.00 0

;

_shelx_hkl_checksum 67388
